# A new discrete dynamic model of ABA-induced stomatal closure predicts key feedback loops

**DOI:** 10.1371/journal.pbio.2003451

**Published:** 2017-09-22

**Authors:** Réka Albert, Biswa R. Acharya, Byeong Wook Jeon, Jorge G. T. Zañudo, Mengmeng Zhu, Karim Osman, Sarah M. Assmann

**Affiliations:** 1 Department of Physics, Pennsylvania State University, University Park, Pennsylvania, United States of America; 2 Biology Department, Pennsylvania State University, University Park, Pennsylvania, United States of America; University of California San Diego, United States of America

## Abstract

Stomata, microscopic pores in leaf surfaces through which water loss and carbon dioxide uptake occur, are closed in response to drought by the phytohormone abscisic acid (ABA). This process is vital for drought tolerance and has been the topic of extensive experimental investigation in the last decades. Although a core signaling chain has been elucidated consisting of ABA binding to receptors, which alleviates negative regulation by protein phosphatases 2C (PP2Cs) of the protein kinase OPEN STOMATA 1 (OST1) and ultimately results in activation of anion channels, osmotic water loss, and stomatal closure, over 70 additional components have been identified, yet their relationships with each other and the core components are poorly elucidated. We integrated and processed hundreds of disparate observations regarding ABA signal transduction responses underlying stomatal closure into a network of 84 nodes and 156 edges and, as a result, established those relationships, including identification of a 36-node, strongly connected (feedback-rich) component as well as its in- and out-components. The network’s domination by a feedback-rich component may reflect a general feature of rapid signaling events. We developed a discrete dynamic model of this network and elucidated the effects of ABA plus knockout or constitutive activity of 79 nodes on both the outcome of the system (closure) and the status of all internal nodes. The model, with more than 10^24^ system states, is far from fully determined by the available data, yet model results agree with existing experiments in 82 cases and disagree in only 17 cases, a validation rate of 75%. Our results reveal nodes that could be engineered to impact stomatal closure in a controlled fashion and also provide over 140 novel predictions for which experimental data are currently lacking. Noting the paucity of wet-bench data regarding combinatorial effects of ABA and internal node activation, we experimentally confirmed several predictions of the model with regard to reactive oxygen species, cytosolic Ca^2+^ (Ca^2+^_c_), and heterotrimeric G-protein signaling. We analyzed dynamics-determining positive and negative feedback loops, thereby elucidating the attractor (dynamic behavior) repertoire of the system and the groups of nodes that determine each attractor. Based on this analysis, we predict the likely presence of a previously unrecognized feedback mechanism dependent on Ca^2+^_c_. This mechanism would provide model agreement with 10 additional experimental observations, for a validation rate of 85%. Our research underscores the importance of feedback regulation in generating robust and adaptable biological responses. The high validation rate of our model illustrates the advantages of discrete dynamic modeling for complex, nonlinear systems common in biology.

## Author summary

Guard cells, located in pairs on the surface of plant leaves, circumscribe microscopic pores called stomata, through which plants take up gaseous carbon dioxide that will be fixed by photosynthesis into sugars. However, plants also inevitably lose water vapor to the atmosphere through open stomata. Under drought conditions, the plant hormone abscisic acid (ABA) causes volume changes in guard cells that result in stomatal closure, thereby restricting water loss. Given the paramount importance of drought tolerance for plant survival, it is important to understand the cellular mechanisms underlying guard cell response to ABA, and over 100 studies in the literature have addressed this topic. We synthesized this information into a network that contains 84 cellular components and 156 interactions between them and then applied logic-based analyses to predict how these components coordinately transduce the ABA signal. We identified several positive feedback loops and mutual inhibition loops that can lead to sustained activity of their constituent components in the presence, or absence, of ABA. Control of these loops, for example, by other stimuli present in the natural environment, may sensitize the system to ABA. We validated some of the novel predictions from our model with new experiments.

## Introduction

The epidermes of leaves and other aerial plant parts have natural openings known as stomata. Stomata are the entry and exit points where gas exchange, particularly CO_2_ uptake for photosynthesis and O_2_ and water vapor diffusion from the leaf interior to the atmosphere, takes place. Each stomate is surrounded by a pair of guard cells that modulate stomatal apertures in response to endogenous water status, to multiple phytohormones, and to many environmental signals such as light and CO_2_ [1–4]. Abscisic acid (ABA) is a key phytohormone that functions as an essential signal in plant responses to abiotic and biotic stress [5, 6]. Drought promotes ABA biosynthesis and accumulation in guard cells [7, 8]. ABA activates complex intracellular signaling cascades that ultimately open anion channels at the guard cell membrane [9], leading to anion efflux and thus cellular depolarization, which in turn drives K^+^ efflux through depolarization-activated outward K^+^ channels [10, 11]. Solute loss drives the osmotic efflux of water through aquaporins [12], resulting in guard cell deflation and stomatal closure [13].

An ultimate goal in modeling a complex system such as guard cell ABA signaling is to create a model in which the output from each element can be quantitatively predicted. This has been accomplished for the small number of “effector” nodes (i.e., ion transporters and channels) of the guard cell network, as provided in the OnGuard software [14]. However, for the intracellular signaling cascade that functions between ABA and effectors, regulation of the secondary messengers is indirect, and little information is available concerning quantitative relationships or response kinetics among the signaling components. In such situations, which are common in complex biological systems, information is simply insufficient to quantitatively parameterize a continuous (kinetic) model, and any attempt to do so would require an inordinate number of assumptions. Network modeling then offers the best way forward to synthesize the available information into a logical framework that is consistent with experimental data [15]. Signaling components (signaling proteins, enzymes, and small molecules) are represented as nodes, the regulatory relationships between them are represented as edges [16, 17], and discrete dynamic modeling is the preferred choice to study the dynamic behavior of the system [18, 19].

We previously constructed the first discrete model of the ABA signaling network that mediates stomatal closure; this model successfully reproduced many observed knockout phenotypes and predicted many new mutant phenotypes [16]. At the time, our 40-node Boolean model of guard cell ABA signaling was the largest system to have been studied by Boolean modeling with asynchronous update. More recently, we constructed a multilevel dynamic model of light-induced stomatal opening and of the effects of CO_2_ and ABA on this process, which, among many other predictions, led us to discover and experimentally address an open question concerning ABA inhibition of red light–induced stomatal opening [20]. In recent years, many new aspects of guard cell ABA signaling have been revealed including, at the molecular level, soluble ABA receptors [21, 22], anion channel genes such as slow anion channel-associated 1 (SLAC1) [23], and aquaporin genes [12]. Upon ABA perception, the soluble ABA receptors interact with and inhibit clade-A protein phosphatase 2Cs (PP2Cs): ABA-insensitive 1 (ABI1), ABA-insensitive 2 (ABI2), hypersensitive to ABA 1 (HAB1), and protein phosphatase 2CA (PP2CA); this interaction relieves inhibition of the serine-threonine kinase OPEN STOMATA 1 (OST1) [24]. Phosphorylation-based channel regulation mediated by OST1 as well as several other classes of protein kinases, including Ca^2+^-dependent kinases (CPKs) and mitogen-activated protein kinases (MPKs), then occurs [25–29]. ABA promotion of cytosolic Ca^2+^ (Ca^2+^_c_) increases, reactive oxygen species (ROS) production, pH increases, and vacuolar acidification also have been found to play positive roles in stomatal closure [30]. Actin reorganization [31] and microtubule depolymerization [32] observed in response to ABA highlight the importance of cytoskeletal rearrangements.

Here, we present a current signaling network and dynamic model for ABA-induced stomatal closure. We extracted information from the published literature and identified 84 signaling components. We employ asynchronous Boolean modeling to study dynamic behaviors of signaling components during signal propagation. We identify long-term states (attractors) and their corresponding precursor states in our dynamic model. A novel stable motif analysis provides a particularly compelling prediction regarding Ca^2+^_c_ regulation of PP2C activation. We also simulate node knockout (in the in silico presence of ABA) for all internal nodes and find that the results for over 50 signaling components agree with phenotypes observed previously by wet-bench experimentation. Moreover, we provide new experimental data that verify an unexpected model prediction regarding the impact of heterotrimeric Gα subunit (GPA1) knockout on ABA regulation of Ca^2+^_c_. We also comprehensively simulate node constitutive activity (in the in silico presence and absence of ABA) and experimentally validate 2 of the resultant predictions regarding ABA hypersensitivity conferred by constitutive activation of Ca^2+^_c_ or ROS.

## Results

### Construction and analysis of the signaling network of ABA-induced stomatal closure

**Fig 1:**
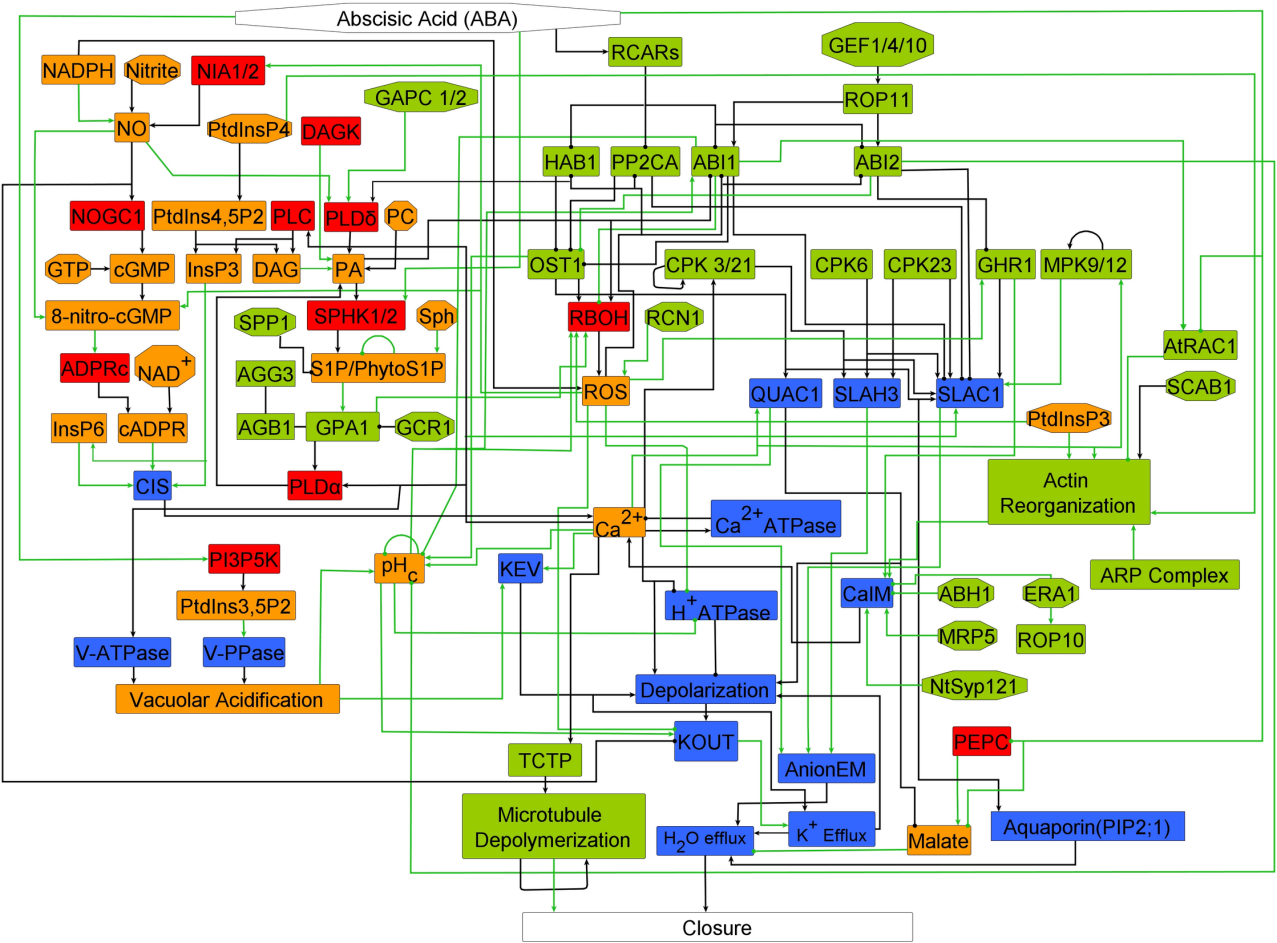
The network of abscisic acid (ABA)-induced stomatal closure. Edges that end in an arrowhead indicate positive interactions or regulatory relationships. Edges that end in a filled circle indicate negative interactions or regulatory relationships. Source nodes (nodes with no incoming edges) are represented by octagons; the rest are rectangles. Edges that represent direct interactions or regulatory relationships are indicated with black, and green edges represent indirect or inferred relationships. The color of the nodes represents their function, as follows: enzymes (red), signaling proteins (green), membrane-transport related nodes (blue), and secondary messengers and small molecules (orange). The full names of network components corresponding to each node label are indicated in S3 Table, and biological justification for the edges is provided in S1 Table, S2 Table, and S1 Text.

Through extensive literature analysis, we identified signaling components and evidence of interactions or causal effects between them from over 120 articles published through 2015, resulting in compilation of over 190 relationships described in the guard cell literature (S1 Table). We interpreted indirect causal relationships in a parsimonious way, e.g., by interpreting causal relationships involving 3 nodes as 2 pairwise relationships (see Materials and methods). The resultant network (Fig 1) has ABA as the primary signal and stomatal closure as the only output node. ABA induces stomatal closure mediated by the soluble pyrabactin resistance 1/pyrabactin resistance 1-like/regulatory component of ABA receptor (PYR1/PYL/RCAR) ABA receptors (RCARs node). The network also contains a few important ABA-regulated nodes, e.g., phosphoenolpyruvate carboxylase (PEPC) [33] and phosphatidylinositol 3-phosphate 5-kinase (PI3P5K) [30], that are not known to be regulated by these soluble receptors. Twenty-two nodes other than ABA are designated as source nodes because they are not known to be regulated by any other nodes in the network. The network also contains 60 intermediate nodes that are signaling proteins (e.g., protein phosphatases, protein kinases), metabolic enzymes (e.g., NADPH oxidases), secondary messengers and small molecules (e.g., Ca^2+^_c,_ cyclic GMP), cytoskeletal proteins (actins and microtubules), and effector proteins (e.g., anion and K^+^ channels and aquaporins). The network contains 156 edges (S2 Table). There are 103 direct edges (direct physical interactions or chemical reactions), 27 indirect edges, and 26 edges inferred during our interpretation of indirect evidence. S1 Text provides a complete biological description of the network, with the associated literature references that support it.

**Fig 2:**
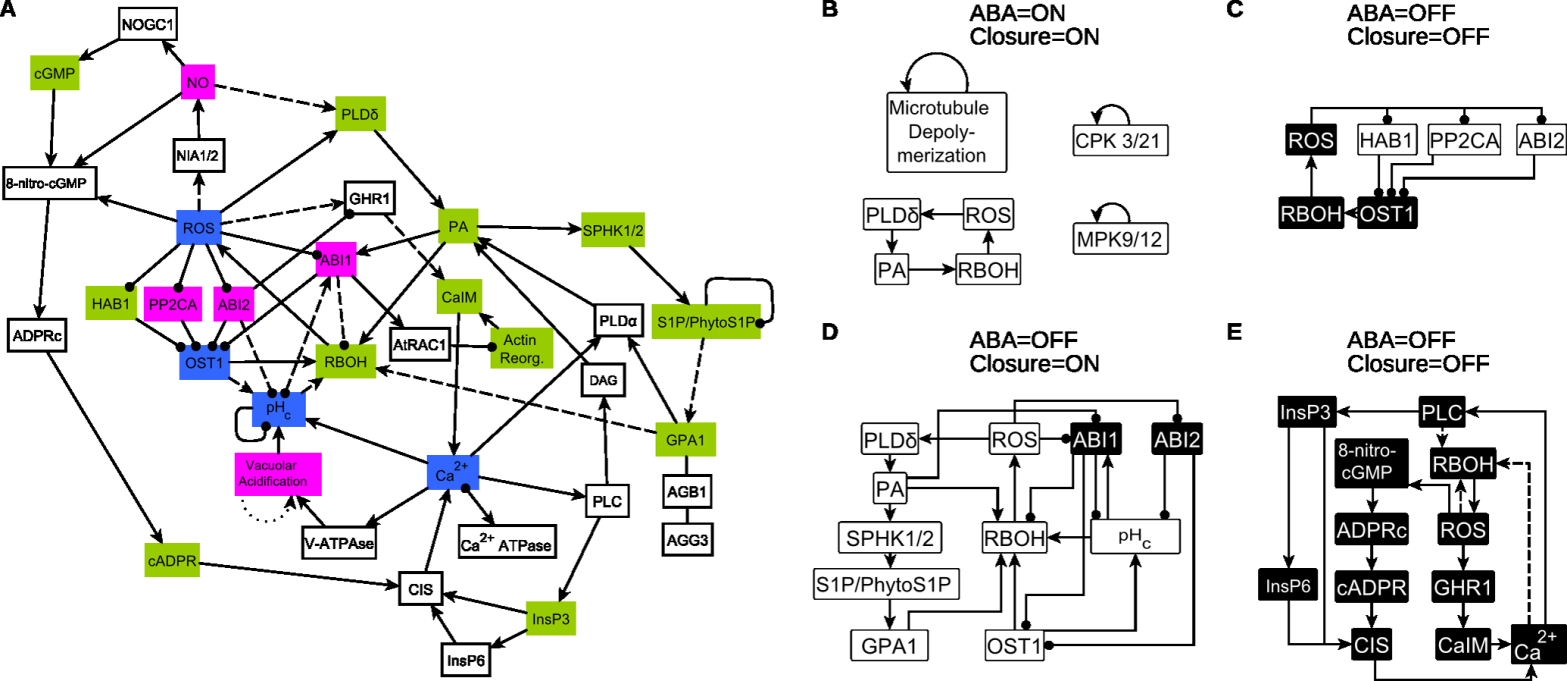
Feedbacks play a key role in the structural and dynamic properties of the abscisic acid (ABA)-induced stomatal closure network. A. The network’s strongly connected component (SCC) comprises almost half of the nodes and more than two-thirds of the edges; it contains both positive and negative feedback loops. Nodes with a light green background are affected by nodes in the in-component, nodes with a blue background regulate nodes of the out-component, and nodes with a pink background interact with both the in-component and the out-component. The dashed edges indicate edges inferred during our network construction process. Even if all dashed edges were removed, 26 nodes would remain in the SCC. The dotted edge indicates a positive self-regulation inferred during the construction of the dynamic model. The nodes that make up the in-component, the SCC, and the out-component are listed in S4 Table. B-D. Stable motifs determine the final outcome (attractor) of the network. The node background indicates the stabilized state of the node; white represents 1 (ON) and black represents 0 (OFF). All stable motifs that are not self-loops are subsets of the SCC. B. Stable motifs associated with closure in response to sustained ABA. C and E. Stable motifs associated with lack of closure in the absence of ABA. The stable motif in E has 3 variants that share all the nodes and all the solid edges. Each variant also includes 2 to 6 additional nodes and 4 to 8 additional edges, which form additional feedback(s) and can be summarized as the indirect relationships shown as dashed edges. D. Stable motif associated with closure in the absence of ABA. Stabilization of this motif requires that Vacuolar Acidification first stabilizes in the ON state; this does not happen in any of the trajectories that start from our assumed initial condition representative of open stomata.

To identify the general information propagation capacity of the network, we determine the network’s largest strongly connected component (SCC) (i.e., feedback-rich subnetwork) and the nodes that can reach or can be reached from it (Materials and methods). The ABA signal transduction network contains a surprisingly large SCC composed of 36 nodes (Fig 2A, S4 Table). This SCC contains multiple positive feedback loops, e.g., Ca^2+^_c_→PLC→InsP3→CIS→Ca^2+^_c_, which describes a mechanism of Ca^2+^_c_-induced Ca^2+^ release, and negative feedback loops, e.g., between Ca^2+^_c_ and the Ca^2+^ ATPase. Twenty-eight nodes are in the network’s in-component (i.e., they can reach the nodes of the SCC through paths), including 22 source nodes (all except CPK6; see S4 Table). Nineteen nodes are in the out-component (can be reached from the SCC), including nodes that comprise plasma membrane ion channels and fluxes and the output node, Closure. All the paths from ABA to Closure pass through the SCC.

We use 3 measures of each node’s signal-mediating role: in-degree (the number of incoming edges), out-degree (the number of outgoing edges), and betweenness centrality (the number of paths mediated by the node, see Materials and methods). The distribution of the nodes according to these 3 measures is shown in S5 Table. Aggregating all 3 measures, 5 well-represented categories of nodes can be identified (Table 1): source nodes, transducers, signal integrators, intermediate SCC members, and key nodes (namely, ABI1, Ca^2+^_c_, phosphatidic acid [PA], cytosolic pH [pH_c_], and ROS).

**Table 1:** Node centrality analysis identifies 5 categories of nodes in the ABA-induced stomatal closure network.

Category	Node count	Nodes
Source nodes other than ABA	22	ABH1, ARP Complex, CPK6, CPK23, DAGK, ERA1, GAPC1/2, GCR1, GEF 1/4/10, GTP, MRP5, Nitrite, NADPH, NAD^+^, NtSyp121, PC, PtdInsP3, PtdInsP4, RCN1, SCAB1, Sph, SPP1
Transducers	30	AGB1, AGG3, AtRAC1, Aquaporin (PIP2;1), AnionEM, Ca^2+^ ATPase, cGMP, CPK3/21, DAG, Depolarization, HAB1, H^+^ ATPase, InsP3, InsP6, KEV, K^+^ Efflux, Malate, MPK9/12, Microtubule Depolymerization, NOGC1, PEPC, PI3P5K, PtdIns(3,5)P2, PtdIns(4,5)P2, PP2CA, RCARs, ROP11, TCTP, V-ATPase, V-PPase
Integrators outside of the SCC	4	H_2_O Efflux, KOUT, SLAC1, SLAH3
Intermediate SCC members	15	8-nitro-cGMP, ABI2, ADPRc, cADPR, GHR1, GPA1, NO, OST1, NIA1/2, PLC, PLDα, PLDδ, QUAC1, SPHK1/2, Vacuolar Acidification
Integrators inside the SCC	5	Actin Reorganization, CaIM, CIS, RBOH, S1P/PhytoS1P
Key nodes	5	ABI1, Ca^2+^_c_, PA, pH_c_, ROS

The first column identifies the categories, the second column indicates the node count, and the third column gives the node list in each category. Source nodes other than ABA (first row) only have 1 or 2 out-going edges (likely because of the scarcity of knowledge about them) and have a betweenness centrality of zero (because they do not mediate any paths). Transducers (second row) have low in- and out-degree and low betweenness centrality; they may belong to any component of the network. Membership in the SCC tends to correlate with a high betweenness centrality. For example, 4 integrators in the out-component of the network have a low betweenness centrality, while the 5 integrators inside the SCC have a moderate to high betweenness centrality. Fifteen nodes of the SCC have moderate in- and out-degree and moderate-to-high betweenness centrality; we refer to these nodes as intermediate SCC members. The nodes in the last row are the most central nodes according to all 3 measures. We did not categorize the signal node ABA, the output node Closure, or the alternate sink node ROP10.

**Abbreviation:** SCC, strongly connected component. All other abbreviations are explained in S3 Table.

### Construction of the dynamic model

Boolean models are a rich, dynamic representation of a signaling system in which each node is characterized by 2 possible states: ON or 1 (interpreted as a higher-than-threshold abundance or activity), and OFF or 0 (lower-than-threshold abundance and activity). For example, the ON state of the OST1 kinase means that there is a sufficient abundance of OST1 proteins in the active, phosphorylated form, such that they can catalyze the phosphorylation of SLAC1 anion channels and other downstream targets. In our dynamic model, we implement the initial states of 54 signaling components as they are known or expected to be in guard cells of open stomata prior to ABA exposure, based on information in the literature, and initialize the remaining 26 randomly (see Materials and methods, S6 Table). The future state of each node is determined by the current state of its direct regulators (the sources of incoming edges) and is expressed as a Boolean regulatory function (see Materials and methods). In Table 2, we give the Boolean regulatory functions for nodes with 3 or more regulators, and associated references. A full list and explanation of the Boolean functions used is in S2 Text. There are 23 unregulated nodes that do not have regulatory functions; they will sustain the state they are initiated with.

**Table 2:** The Boolean regulatory function of each node expresses the combined effect of the node’s regulators in a logical form.

Node	Regulatory function	**References**
ABI1	ABI1* = not PA and (not RCARs or ROP11) and not ROS and pH_c_	[21, 34–38]
ABI2	ABI2* = (not RCARS or ROP11) and not ROS	[21, 39, 40]
OST1	OST1* = (not ABI1 and not HAB1) or (not PP2CA and not ABI2) or (not ABI1 and not ABI2) or (not HAB1 and not PP2CA) or (not HABI1 and not ABI2) or (not ABI1 and not PP2CA)	[22, 41–45]
RBOH	RBOH* = pH_c_ and not ABI1 and PtdInsP3 and OST1 and GPA1 and PA and RCN1	[25, 46–55]
NO	NO* = Nitrite and NIA1/2 and NADPH	[56]
8-nitro-cGMP	8-nitro-cGMP* = cGMP and ROS and NO	[57]
CIS	CIS* = InsP3 or InsP6 or cADPR	[58–60]
CaIM	CaIM* = Actin Reorganization or (NtSyp121 and GHR1 and MRP5) or not ABH1 or not ERA1	[61–65]
Ca^2+^_c_	Ca^2+^_c_* = (CIS or CaIM) and not Ca^2+^ ATPase	[16]
PLDδ	PLDδ* = NO or ROS and GAPC1/2	[66, 67]
PA	PA* = PC and (PLDδ or PLDα) or DAG and DAGK	[68, 69]
S1P/PhytoS1P	S1P/PhytoS1P* = SPHK1/2 and Sph and not SPP1	[70, 71]
Vacuolar Acidification	Vacuolar Acidification* = V-PPase or V-ATPase or Vacuolar Acidification	[30, 72–76]
pH_c_	pH_c_* = (OST1 and not ABI2 and not ABI1 or Ca^2+^_c_) and Vacuolar Acidification	[30, 77, 78]
H^+^ ATPase	H^+^ ATPase* = not pH_c_ and not Ca^2+^_c_ and not ROS	[79–81]
Actin Reorganization	Actin Reorganization* = (PtdInsP4 or PtdInsP3) and not AtRAC1 and ARP Complex and SCAB1	[31, 82–84]
SLAC1	SLAC1* = (CPK6 or CPK23 or CPK3/21) and MPK9/12 and OST1 and GHR1 and pH_c_ and not ABI1 and not PP2CA and not ABI2	[25–29, 63, 85–88]
SLAH3	SLAH3* = (CPK6 or CPK23) and CPK3/21 and not ABI1	[26, 89]
AnionEM	AnionEM* = SLAC1 or QUAC1 and SLAH3	[90, 91]
Malate	Malate* = PEPC and not ABA and not AnionEM	[16, 33, 92]
Depolarization	Depolarization* = (AnionEM or Ca^2+^_c_ or KEV) and (not H^+^ ATPase or not K^+^ efflux)	[16]
KOUT	KOUT* = (not NO or not ROS or pH_c_) and Depolarization	[93–95]
H_2_O Efflux	H_2_O Efflux* = (AnionEM and PIP2; 1 and K^+^ efflux) and not Malate	[12]

The regulatory functions of nodes with 3 or more regulators as well as the supporting references are included here. The node states are represented by the node names (with full names given in S3 Table). * is used to denote the future state of a node. The full list of regulatory functions for all nodes, with extensive justification for each, is given in S2 Text.

In Boolean network models, the simulation of successive events (e.g., successive activation of receptor proteins, downstream second messengers, and ion channels) is implemented by update schemes (see Materials and methods). We used a stochastic update algorithm in which the nodes are updated in a randomly selected order for 30 rounds (time steps). In each simulation, the system transitions through several states until it settles down in an attractor (e.g., a steady state or oscillation). In each setting, we used 2,500 replicate simulations, i.e., 2,500 “in silico stomata” and determined at each time step the percentage of simulations in which the node Closure is in state 1, which we will refer to as the percentage of closure.

### Analysis of the wild-type system

**Fig 3:**
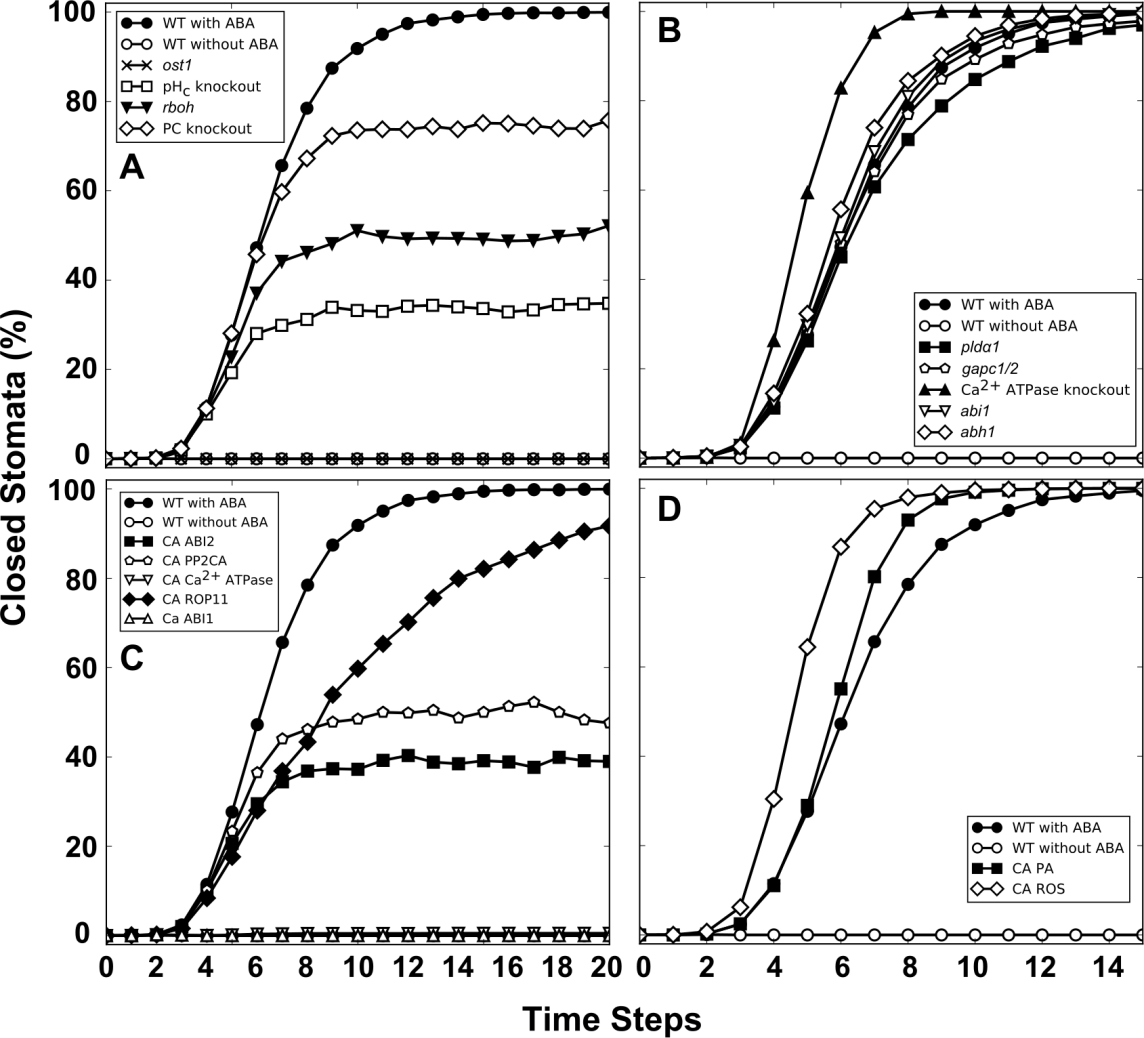
Node knockout (sustained OFF state) or constitutive activity (sustained ON state) can lead to a variety of divergences from the wild-type system’s response to abscisic acid (ABA), in agreement with experiments. The percentage of closed stomata in the wild-type system reaches 100% in the presence of ABA (filled circles) and stays at 0% in the absence of ABA (open circles). A. Simulated knockouts that lead to ABA insensitivity (*ost1*) or reduced sensitivity to ABA (*rboh*, cytosolic pH [pH_c_] clamp, phosphatidylcholine [PC] depletion). B. Examples of node knockouts that lead to ABA hyposensitivity (*pld*α, *gapc1/2*) or ABA hypersensitivity (*abi1*, *abh1*, Ca^2+^ ATPase knockout). C. Examples of constitutive node activation that lead to insensitivity to ABA (*abi1* dominant mutant, constitutively active Ca^2+^_c_ ATPase), reduced ABA sensitivity (constitutive activity of ABA-insensitive 2 [ABI2] or protein phosphatase 2CA [PP2CA]), or ABA hyposensitivity (ROP11 constitutive activity). D. Examples of constitutive node activity that lead to ABA hypersensitivity: supply of phosphatidic acid (PA) and supply of reactive oxygen species (ROS), which we experimentally assess and confirm. Fewer than 30 time steps are illustrated in all panels simply to better display the differences among curves. The numerical data can be found in S1 Data.

We started with simulation of the response of wild-type stomata following the sustained presence of ABA (Fig 3, filled circles). The percentage of closure is initially 0, corresponding to open stomata. After a time delay of approximately 4 time steps, during which the signal propagates through the network, the percentage of closure increases dramatically, reaching 100% by time step 20. This indicates that all 2,500 simulations, regardless of the random differences in the initial states of 26 nodes and of the random differences in the order of update of the nodes, reach a state that corresponds to stomatal closure. In the converse situation of sustained absence of ABA (Fig 3, open circles), the percentage of closure remains at zero, indicating that no simulations are able to reach closure in the absence of the ABA signal. These results indicate that we have captured the expected behavior of the wild-type system.

For a deeper analysis of the long-term states (attractors) reached by the system in the presence and absence of ABA, we performed stable motif analysis (Materials and methods). This analysis is based on finding feedback loops that stabilize in a fixed state and determine the system’s attractors [96]. In the sustained presence of ABA, there are 4 such stable motifs (Fig 2B): positive self-regulation of CPK3/21, MPK9/12, and Microtubule Depolymerization, and the positive feedback loop PA→RBOH→ROS→PLDδ→PA, each of which leads to a sustained ON state of the corresponding node(s). In the presence of ABA, any dynamic trajectory leads to the stabilization of each of the motifs of Fig 2B, which yields an attractor of the system (S6 Table) in which 70 nodes stabilize (most, but not all, in opposite states than their initial state). Ten nodes oscillate, driven by the negative feedback loop between Ca^2+^_c_ and Ca^2+^ ATPase; most of these nodes are well known to be regulated by and/or regulate Ca^2+^_c_ and indeed Ca^2+^_c_ is observed to oscillate in response to ABA [59, 60, 97]. The model-predicted attractor is in good agreement with the known response of the network elements to the sustained presence of ABA [1, 3, 98–101].

In the absence of ABA, there are 2 main stable motifs associated with the state of no stomatal closure. The first represents the OFF state of the positive feedback loop between OST1 and ROS combined with the ON state of 3 PP2Cs, e.g., ABI2, HAB1, and PP2CA, each of which is in a mutually inhibitory relationship with ROS (Fig 2C). Second, a family of 3 related stable motifs (Fig 2E) represents the OFF state of 2 interlocking positive feedback loops involving Ca^2+^ influx through the membrane (CaIM) and Ca^2+^ release from internal stores (CIS), respectively. Stabilization of the first motif leads to stabilization of the second. In addition, the self-regulation of 4 nodes, namely CPK3/21, MPK9/12, Microtubule Depolymerization, and Vacuolar Acidification, allows their stabilization in either the ON or the OFF state in the absence of ABA. As a consequence, there are 16 reachable final attractors (one for each combination of states of these 4 nodes) associated with the absence of stomatal closure (S6 Table and S7 Table), which nevertheless have identical states for all but 9 nodes. The stabilized state of many nodes is identical to their initial states, which correspond to open stomata, as expected.

In addition, there also exists a stable motif associated with closure (Fig 2D). In the absence of ABA, this motif does not stabilize in any of the trajectories that start from our initial condition corresponding to open stomata. Accordingly, our model predicts that there are no trajectories that would lead to closure of open stomata in the absence of ABA or other closing signals, consistent with biological reality.

### Simulated node knockout (KO) and constitutive activity (CA) in the presence of ABA

We performed a comprehensive analysis of the effects of single node knockout (sustained OFF state, see Materials and methods) and constitutive activity (sustained ON state) on ABA-induced stomatal closure. We observed 4 major types of responses: insensitivity, reduced sensitivity, hyposensitivity, and hypersensitivity to ABA (Materials and methods). Fig 3 illustrates a few representative cases. The model predicts that *ost1* knockout leads to insensitivity to ABA (Fig 3A), in agreement with experiments [25, 48]. Two example cases of reduced ABA sensitivity (*rboh* and pH_c_ clamp) are well supported experimentally [52]. A third, phosphatidylcholine (PC) depletion, is not amenable to experimental study, as PC is a fundamental component of the lipid bilayer. Fig 3B shows examples of node knockouts that lead to ABA hyposensitivity (*pld*α and *gapc1/2*), both supported by the literature [35, 67]. Of the 3 examples of ABA hypersensitivity shown (*abi1*, *abh1*, and Ca^2+^ ATPase knockout), the first 2 are documented cases of increased ABA sensitivity [44, 65], while knockout of Ca^2+^ pumps has not been experimentally evaluated. Fig 3C shows examples of constitutive node activity that lead to insensitivity to ABA (*abi1* dominant mutant, constitutively active Ca^2+^_c_ ATPase) or reduced ABA sensitivity (constitutive activity of ABI2 or PP2CA). Impaired ABA sensitivity of *abi1* and *abi2* dominant mutants was indeed observed [50, 102, 103]. The percentage of closure in the case of ROP11 constitutive activity approaches 100% closure by 30 time steps; thus, this is an example of ABA hyposensitivity, which also agrees with experiments [104]. Fig 3D shows examples of constitutive node activity that lead to ABA hypersensitivity: supply of PA, which agrees with experimental observations [35], or supply of ROS, which we experimentally assess and confirm.

Summarizing the results of individual knockout or constitutive activity of all 79 internal nodes in the presence of ABA (S8 Table), 30% lead to no appreciable difference from a wild-type response to ABA (i.e., stomatal closure), 30% lead to a more effective response, 14% lead to a somewhat reduced response, and 26% lead to a dramatically reduced response. Where genetic or pharmacological intervention experiments have been performed, comparison with these wet-bench results can serve to evaluate the model. Table 3 summarizes the relationship between the in silico results and the closest comparable experimental results. Agreement is obtained in 57 cases of 158 (shown in bold font) or 36% and there are 16 cases of discrepancy (10%, shown in italic font). In most discrepancies, the model is indicating close to wild-type response to ABA, while experiments found decreased (1b) or increased (1a) sensitivity to ABA. In the remaining 54% of cases, no experimental data are currently available; thus, the model’s result is a novel prediction (S8 Table).

**Table 3:** Agreement between experimental results and simulated outcomes in the presence of abscisic acid (ABA).

Response category	Total number of cases	Simulated interventions for which experimental results exist	CPC range
Equivalent to wild type	22	*SCAB1 CA (1b)* [83], *GEF1/4/10 KO (1a)* [40, 105], *SPP1 KO (1a)* [106]	23.93–24.08
Hypersensitivity	48	**SphK1/2 CA** [107, 108], **HAB1 KO** [44, 45], **RCARs CA** [109], **GCR1 KO** [110], **TCTP CA** [32], **S1P/PhytoS1P CA** [70, 111], **ABI1 KO** [43, 112], **OST1 CA** [25], **ABI2 KO** [43], **ERA1 KO** [113], **ABH1 KO** [65], **PA CA** [114], **PLD**α **CA** [114] [36]; *NIA1/2 KO (1b*) [56], *Microtubule Depolymerization CA (2)* [115, 116]	24.08–26.7
Close to wild type	25	**CPK6 KO** [86], **cGMP CA** [57]; *MRP5 KO (1b)* [64], *InsP3 KO (1b)* [59, 117], *pH*_*c*_ *CA (1a)* [78], *cGMP KO (1b)* [57], *V-ATPase KO (1b)* [30] [104], *NO KO (1b)* [56], *ROP11 KO (1a)* [34], *PP2CA KO (1a)* [118], *NOGC1 KO (1b)* [57]	23.92–24.07
Hyposensitivity	22	**ROP11 CA** [104], **GEF1/4/10 CA** [40, 105], **QUAC1 KO** [119, 120], **PLC KO** [59, 117], **PtdIns(4,5)P2 KO** [30, 121], **PtdInsP4 KO** [121], **PLD**α **KO** [36], **V-PPase KO** [30], **CIS KO** [122, 123], **PtdIns(3,5)P2 KO** [30], **cADPR KO** [124], **PI3P5K KO** [30], **GAPC KO** [67], **ADPRc KO** [124, 125]; *SLAH3 KO (2)* [89]	20.22–23.91
Reduced sensitivity	26	**Vacuolar Acidification KO** [30], **pH**_**c**_ **KO** [87], **ABI2 CA** [102], **H**^**+**^ **ATPase CA** [126], **CaIM KO** [127], **PP2CA CA** [118], **MPK9/12 KO** [29], **S1P/PhytoS1P KO** [70, 111], **SphK1/2 KO** [107, 108], **PtdInsP3 KO** [121], **GHR1 KO** [63], **SLAC1 KO** [23], **RCN1 KO** [128], **ROS KO** [46], **RBOH KO** [46], **PA KO** [114], **ARP complex KO** [31],**AtRAC1 CA** [82], **SCAB1 KO** [83], **PLDδ KO** [67]; *GPA1 KO (2)* [87]	5.9–21.02
Insensitivity	15	**Microtubule Depolymerization KO** [116], **K**^**+**^ **Efflux KO** [11], **KOUT KO** [11], **Aquaporin (PIP2;1) KO** [12], **OST1 KO** [24, 129], **RCARs KO** [42, 130], **ABI1 CA** [102], **Ca**^**2+**^_**c**_ **KO** [122, 123]	0.0–0.02

The interventions considered are a sustained OFF state (denoted KO), corresponding to knockout of a gene, disruption of a process, or subthreshold concentration of a molecule, and a sustained ON state (denoted CA), corresponding to constitutive activity of a protein, activation of a process, or external supply of a molecule. Simulated manipulations of the state of source nodes that do not cause any alterations of the initial condition (e.g., supply of a molecule that is already abundant) can be considered as replicates of the wild-type simulation and are denoted “equivalent to wild type.” Interventions that lead to CPC (see Materials and methods) within the wild-type equivalent range are denoted “close to wild type.” Hyposensitivity refers to reaching closure in all simulations but with a longer time line (more time steps) than the wild-type equivalent simulations, reflected in a CPC smaller than the lowest wild-type equivalent CPC (23.93). Hypersensitivity refers to closure in all simulations with a shorter time line (fewer time steps) than the wild-type equivalent simulations and a CPC larger than the largest wild-type CPC (24.08). Reduced sensitivity means that the percentage of closure stabilizes at a value less than 100%; insensitivity means that the percentage of closure stabilizes at 0%. The node names within each category are grouped according to the results of the comparison with the closest experimental information (reported in the indicated reference) and within each group are shown in increasing order of CPC. Genetic or pharmacological KO experiments are compared with the results of simulated KOs. Experimental overexpression or external supply are compared with the results of simulated CA. We note that overexpression of a protein may not correspond to its CA, especially if its activation involves posttranslational modification. Bold font indicates that the model result is consistent with the experimental data and italic font indicates that the model result is not consistent with experimental data. We categorize the cases of discrepancy between model and experiment into 2 types and indicate the category in parenthesis after the node name. In type 1, the model indicates a response equivalent/close to wild type while the experiments indicate significantly increased (1a) or decreased (1b) response compared to wild type. In type 2, the model indicates significant deviation from wild type (of the kind shown in the first column), and the experiments indicate wild-type response.

**Abbreviations:** CA, constitutive activity; CPC, cumulative percentage of closure; KO, knockout. All other abbreviations are explained in S3 Table.

Stable motif-based attractor analysis indicates that all the interventions that lead to a final closure probability of 100% (i.e. hyposensitivity, close to wild-type response, and hypersensitivity) lead to an attractor identical or very close to the attractor corresponding to wild-type ABA-induced closure (S6 Table). Any differences from this attractor are limited to the node that is knocked out or constitutively activated and up to a few nodes immediately regulated by it, but the stable motifs (shown in Fig 2B) are preserved. Interventions that lead to reduced ABA sensitivity lead to an attractor in which AnionEM, H_2_O efflux, and Closure oscillate, driven by oscillating Ca^2+^_c_ (see S9 Table for an example). There are 2 types of attractors corresponding to insensitivity to ABA: one type similar to the attractors expressing lack of closure in the absence of ABA (e.g., in case of RCARs knockout) and another type that preserves the stable motifs of Fig 2B but Closure is OFF because one of its required upstream regulators is OFF (S9 Table).

The model also can be used to predict how genetic or pharmacological knockout of an internal node affects other internal nodes. S10 Table shows agreement between the model result and experimental observation for 12 of these instances, supporting the internal consistency and predictive power of the model.

### Experimental validation of model predictions

**Fig 4:**
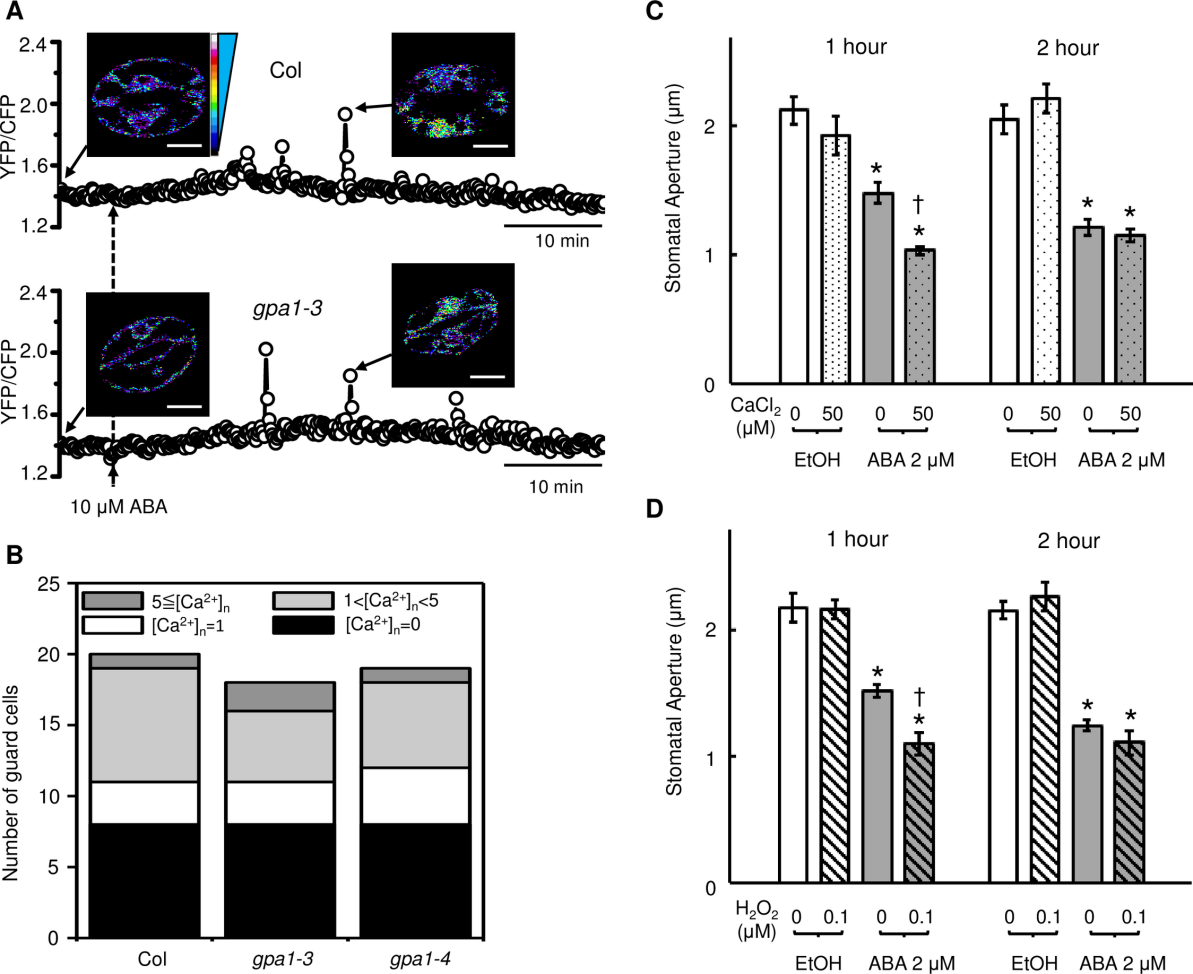
Experimental validation of model predictions. A,B. Wild-type ABA response of Ca^2+^_c_ oscillation in *gpa1* null mutants. A. Representative ABA-induced oscillations in Ca^2+^_c_ using Y3.6 fluorescent reporter. The fluorescence emission ratio of yellow fluorescent protein (YFP) to cyan fluorescent protein (CFP) (YFP/CFP) was calculated from YFP and CFP emission intensities. Pseudo-colored scale bar refers to both wild-type Columbia accession (Col) and *gpa1* data. B. The numbers of guard cells exhibiting different types of ABA-induced Ca^2+^_c_ oscillation in Col (*n* = 20), *gpa1-3* (*n* = 18), and *gpa1-4* (*n* = 19). C,D. ABA hypersensitivity in *Arabidopsis thaliana* stomatal closure upon external supply of Ca^2+^ or H_2_O_2_. Ca^2+^ or H_2_O_2_ alone does not induce closure at the low concentrations used here. C. CaCl_2_ (50 μM) accelerates ABA (2 μM)-induced stomatal closure in epidermal peels at 1 h. D. H_2_O_2_ (0.1 μM) accelerates ABA (2 μM)-induced stomatal closure in epidermal peels at 1 h. The asterisk above the sample indicates statistical significance (Student *t* test *p* < 0.05) between this sample and the same sample within the ethanol (EtOH) (solvent control) group; the dagger symbol indicates statistical significance (Student *t* test *p* < 0.05) between the 2 samples within the ABA treatment group. Data are mean ± SE of 3 independent replicates with 64 stomata per replicate. The numerical data (for B, C, and D) can be found in S1 Data.

The most valuable biological models provide new predictions that guide experimental research. One especially intriguing prediction of our model is that *GPA1* knockout should not significantly impact Ca^2+^_c_ oscillations. While *gpa1* mutants are known to be impaired in ABA activation of the ROS-activated plasma membrane Ca^2+^ channels [51], which our model captures, the simulated loss of GPA1 does not affect stretch-activated Ca^2+^ channels, nor does it affect Ca^2+^-induced CIS in our model. We tested this prediction by introducing the Yellow Cameleon 3.6 (YC 3.6) fluorescent Ca^2+^ reporter into *gpa1* guard cells and measuring Ca^2+^_c_ following ABA application. As is evident in Fig 4A and 4B, ABA does initiate wild-type Ca^2+^_c_ transients in *gpa1* guard cells, consistent with our dynamic model.

Given the paucity of experimental data on the effects of node constitutive activation in the presence of ABA, we decided to test 2 such predictions: constitutive activation of Ca^2+^_c_ by provision of external Ca^2+^ [131, 132] and constitutive activation of ROS by provision of H_2_O_2_. As shown in Fig 4C and 4D, in both cases, the experimental results support the model predictions: in agreement with our definition of ABA hypersensitivity, stomatal closure occurred more rapidly when the internal node was constitutively activated, and it reached the same final closure state. These data illustrate the predictive power of our model.

### The effects of constitutive activity or external supply of a node in the absence of ABA

**Fig 5:**
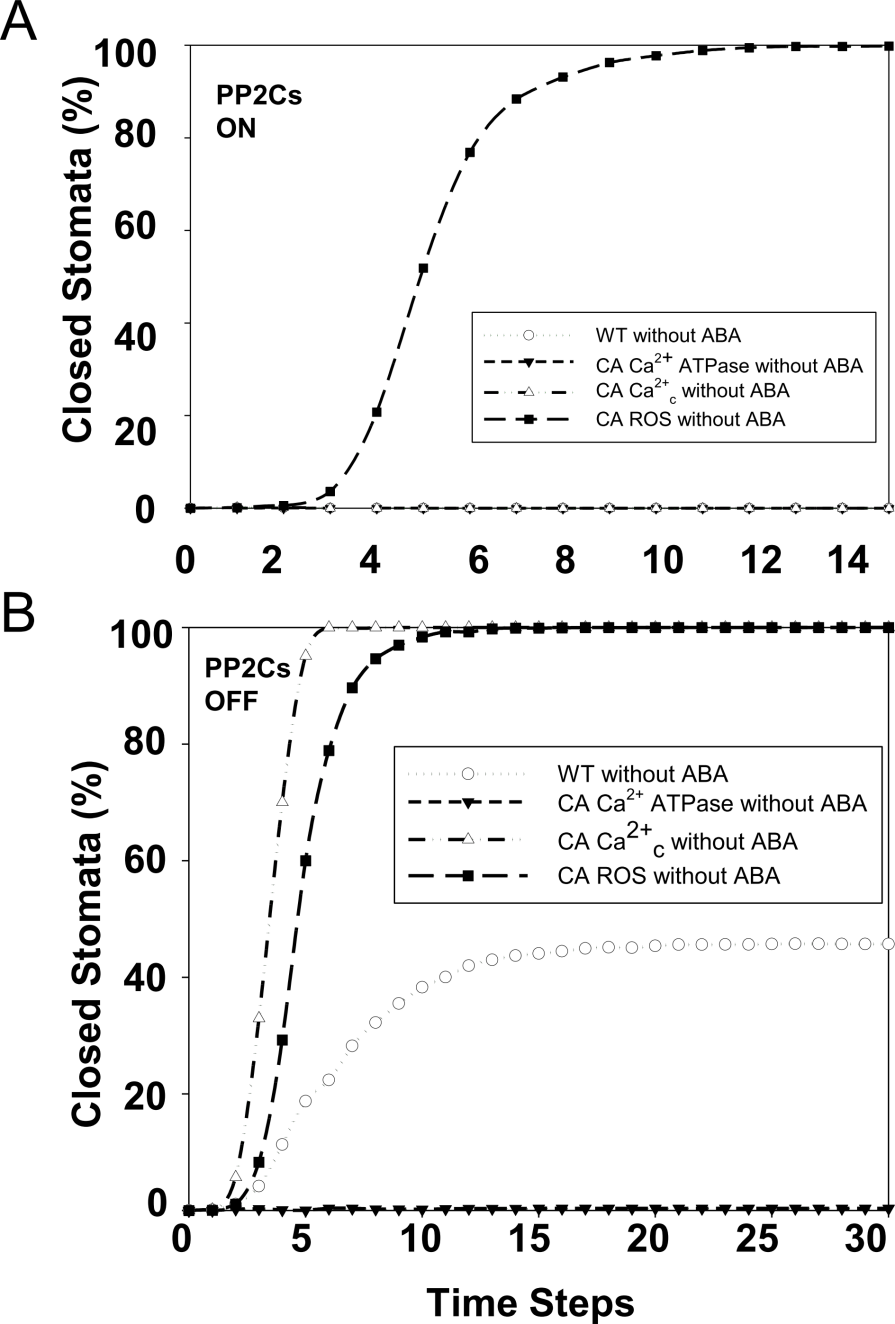
The system’s response to simulated constitutive activity or external supply of nodes in the absence of abscisic acid (ABA) depends on the initial activity of the protein phosphatases 2C (PP2Cs). A. The PP2Cs are assumed to be ON in the initial state. The wild-type system in the absence of ABA (open circles) shows lack of closure in all simulations. Constitutively high concentration of cytosolic Ca^2+^ (Ca^2+^_c_) or constitutive activity (CA) of Ca^2+^ ATPase similarly leads to lack of closure, but supply of reactive oxygen species (ROS) leads to a response similar to the response to ABA (Fig 3, closed circles). B. The PP2Cs are assumed to be OFF in the initial state. Now there is a nonzero probability of closure in the absence of ABA. Supply of ROS or sustained high Ca^2+^_c_ leads to a response similar to the response to ABA (Fig 3, closed circles), consistent with experimental observations. CA of the Ca^2+^ ATPase leads to the absence of closure in all simulations. The numerical data can be found in S1 Data.

We next considered effects of constitutive activation or external supply of a node in the absence of ABA. According to model predictions, the constitutive activity of any of 67 nodes does not lead to any predicted stomatal closure from the open state, while for 12 nodes, there is a small (7 cases) or significant (5 cases) closure response (Table 4, S11 Table). Only constitutive activity of ROS leads to a closure probability similar to the response to ABA (Fig 5A), consistent with experimental data [51, 63]. Comparing to experimental observations, there is agreement in 13 cases (16%) and disagreement in 11 cases. Notably, for the majority of cases (70%, see S11 Table), there are no comparable experiments, again highlighting the usefulness of modeling in hypothesis generation.

**Table 4:** Weaker agreement between experimental results and model outcomes upon simulated constitutive activity (or external supply) of nodes in the absence of abscisic acid (ABA).

Response category	Total number of cases	Cases that are consistent with experimental results	Cases that are inconsistent with experimental results	CPC range
Equivalent to wild type (signal-free)	20	**SCAB1** [83]	*Nitrite (1a)* [56]	0.0–0.002
Close to wild type	47	**AtRAC1** [82], **SphK1/2** [107], **PLD**α [36], **PP2CA** [118], **ROP11** [104], **ABI1** [102], **cGMP** [57], **ABI2** [102], **H**^**+**^ **ATPase** [133]	*S1P/ PhytoS1P (1a)* [70, 134], *pH*_*c*_ *increase (1a)* [78], *NO (1a)* [56], *cADPR (1a)* [124], *PA (1a)* [114], *8-nitro-cGMP (1a)* [57], *InsP3 (1a)* [135]	0.0–0.0024
Slightly increased response	7	**TCTP** [32], **Microtubule Depolymerization** [116]	*Ca*^*2+*^_*c*_ *(1a*) [136]	0.0028–0.03
Significantly increased response	5	**ROS** [46, 137]	*OST1 (2*) [25], *RCARs (2)* [42, 130]	5.8–25.2

The wild-type system in this case refers to an initial condition that represents open stomata in the absence of any closure signal, with initial node states summarized in S5 Table. Cases of constitutive activity of a source node that is ON in the initial condition can be considered as replicates of the wild-type simulation and are denoted “equivalent to wild type.” Node activations that lead to a CPC within the wild-type equivalent range (0–0.002) are denoted “close to wild type.” The “equivalent to wild type” and “close to wild type” categories lead to a final closure percentage of 0% and may include transient closure in a few simulations. “Slightly increased response” corresponds to a final closure percentage of 0%, with a higher degree of transient closure and a CPC larger than the highest wild-type equivalent CPC. The category “significantly increased response” indicates a nonzero final closure probability, including a 100% closure probability for RBOH or ROS constitutive activity. The nodes are in the order of increasing CPC in each category. Discrepancy type designations are as in Table 3 with the addition that the category “slightly increased response” is considered consistent with experimentally observed wild-type responses.

**Abbreviations:** CPC, cumulative percentage of closure. All other abbreviations are explained in S3 Table.

Our stable motif analysis explains the effectiveness of ROS in promoting stomatal closure in the absence of ABA: it can lead to the stabilization of the stable motif in Fig 2D. The model indicates that sustained activity of ROS, RBOH, RCARs, or OST1 can induce the stabilization of this stable motif in a fraction of the trajectories in the system, which then leads to stomatal closure in these trajectories. This result is seemingly at odds with the experimental observation that overexpression of OST1 and RCARs does not lead to increased stomatal closure in the absence of ABA [25, 109]. One reason for apparent inconsistency with experimental results is that overexpression of a protein may not correspond to its constitutive activation, e.g., if its activation involves posttranslational modification. Experimental observations also indicate more potential nodes whose constitutive activation is able to lead to closure than are predicted by our simulations, namely, the 9 nodes categorized as 1a in Table 4.

### Predicted destabilization of the PP2Cs

We investigated the basis for the above-noted discrepancy between model and experimental results and identified it in the effect of the PP2C family member protein phosphatases. Because, according to experimental observations, these PP2Cs are biochemically active in the absence of ABA [103], they lead to stabilization of the stable motif in Fig 2C in all the trajectories of the signal-free system and even in the vast majority of trajectories in the presence of internal drivers. Elevated PA or S1P can overcome this outcome only when combined with destabilization of the activity of multiple PP2Cs. To explicitly test in silico the importance of the initial state of the PP2Cs, we reanalyzed the model with a revised initial state, in which the 4 PP2Cs are inactive (OFF). We found that the stable motif in Fig 2D now has a chance of being stabilized, leading to an attractor (A17 in S7 Table) close to the attractor corresponding to ABA-induced closure (S6 Table). As indicated in Fig 5B, the simulated system with the 4 PP2Cs initialized as OFF but no other interventions (a situation that we call the baseline) reaches closure in around 44% of trajectories. The same result of nonzero percentage of closure is observed for constitutive activity of the majority of nodes (S12 Table).

Importantly, the model now predicts robust closure for 20 internal drivers. When combined with an initial down-regulation of the PP2C protein phosphatases, sustained Ca^2+^_c_ or sustained CaIM [136] leads to a closure response similar to the wild-type response to ABA (see Fig 5B), and external supply of S1P [70] now also leads to an increased frequency of closure, consistent with experimental results. There are no wet-bench experiments that manipulate the initial state of the PP2Cs, but if one uses the experiments that served as comparison in Table 4, agreement is now found for 10 of the 11 cases of italicized discrepancy (every case except supply of nitrite). The resolved nodes are shown with bold font in S12 Table. Importantly, the consistency with experiments of the boldface nodes in Table 4 is maintained.

Stable motif analysis of the simulated system in the absence of ABA with all 4 PP2Cs knocked out (S13 Table) indicates that there are 15 attractors: 14 attractors associated with lack of closure (with a similar node expression pattern as in S6 Table) as well as an attractor associated with closure (the same as in S7 Table). Importantly, the existence of this closure attractor is supported by the experimental observation that the *hab-1 abi-2 pp2ca-1* triple null mutant shows partial stomatal closure in the absence of ABA [45]. The shared observation of the attractor associated with closure for both initial inactivity of the 4 PP2Cs and for their knockout indicates that transient inactivity of the 4 inhibitors of closure can be made permanent by the feedbacks in the system (specifically, by the stable motif in Fig 2D).

The assumption that the PP2C phosphatases are present but initially inactive in open stomata has not been evaluated experimentally, and the prediction from this assumption of a significant degree of closure in the absence of ABA (open circles in Fig 5B) is not consistent with current knowledge regarding behavior of guard cells with intact PP2Cs. However, the strong effect of a merely transient (initial) down-regulation of the PP2Cs (S12 Table) suggests that alternative, weaker assumptions may also be explanatory. Reinspecting the stable motifs of Fig 2C and 2D, which correspond to 2 opposite outcomes in the absence of ABA, one can see that both involve mutual inhibition between, on the one hand, PP2Cs and, on the other hand, OST1, RBOH, and ROS. If ABI2, PP2CA, and HAB1 are ON, then OST1, RBOH, and ROS are OFF (Fig 2C). Conversely, destabilization of the activity of ABI1 and ABI2, coupled with the stabilization of the positive feedback loop PA→RBOH→ROS→PLDδ→PA, can lead to the stabilization of the stable motif in Fig 2D and thus to closure in the absence of ABA. We find that assuming initial inactivity of only ABI1 and ABI2 also resolves the inconsistencies with experimental observations of Table 4 and at the same time leads to a lower baseline probability of closure (S14 Table).

Because 7 of the nodes with type 1a discrepancies in Table 4 participate in paths that lead to Ca^2+^ release from stores (for example, Nitrite→NO→NOGC1→cGMP→8-nitro-cGMP→ADPRc→cADPR→CIS), an alternative specific hypothesis is that Ca^2+^_c_ increase leads to the inactivation of one or more PP2Cs. Such a mechanism would not affect the system’s responsiveness to ABA, because the PP2Cs are inactivated early by RCARs. It is also consistent with knockout of RCARs, leading to insensitivity to ABA, as our model indicates that the loss of RCARs leads to the absence of an increase in Ca^2+^_c_. We find that assuming that Ca^2+^_c_ increase leads to the inactivation of all 4 PP2Cs resolves 10 of the 11 inconsistencies with experimental observations of Table 4 (every case except supply of nitrite) and at the same time leads to a lower baseline probability of closure in the absence of ABA (S14 Table). The thus-augmented model’s experimental validation rate is increased to 85% (see Materials and methods). Consistent with our hypothesis is the observation that dominant negative *abi1* and *abi2* mutants fail to show stomatal closure induced by external Ca^2+^ application [138]. In fact, an early study on recombinant ABI1 observed inhibition of phosphatase activity by Ca^2+^ in vitro, but the result was interpreted to lack biological relevance, as inhibition was only observed at much higher Ca^2+^ concentrations than those found in living cells [139]. Our model suggests that ABI1 may be more sensitive to Ca^2+^ inhibition in vivo than in vitro, and it is intriguing to note that the ABI1 protein contains a putative Ca^2+^-binding EF hand motif [139].

## Discussion

Our construction of a network of ABA-induced stomatal closure based on the literature provides a means to comprehend the vast number of relationships reported for this complex system. In network construction, we employed causal network inference and binary transitive reduction [140] to facilitate identification of the most parsimonious paths between the more than 80 known signaling components of ABA-induced stomatal closure. We found that the system is strongly nonlinear. The network contains a 36-node SCC (Fig 2A) that includes 5 key nodes with high in- and out-degree (ABI1, Ca^2+^_c_, PA, pH_c_, and ROS), several nodes, particularly CaIM and RBOH, that serve as integrators of information, and several nodes with high out-degree, such as OST1 (Table 1). The integrators and PA receive information from the network’s in-component (which includes ABA), while Ca^2+^_c_, ROS, and pH_c_ relay information to the network’s out-component (which includes Closure), and ABI1 performs both functions (Fig 2A). Indeed, these nodes are known important mediators of ABA-induced stomatal closure [47, 52, 77], supporting the validity of our network reconstruction. The SCC serves as a core of information processing and mediator of all the paths between ABA and closure. Its strong nonlinearity emphasizes how simple epistatic reasoning may not always be valid for biological systems. The SCC contains numerous positive feedback loops, several of which constitute stable motifs. This inclusive relationship between the SCC (a property of the network) and the stable motifs (a property of the dynamic model) reflects the added insights contributed by dynamic modeling. The SCC identifies the interrelatedness of its 36 nodes; the stable motifs identify which interrelated sets of nodes will stabilize and drive the system’s outcome in the presence or absence of ABA.

Given the complexity of the guard cell ABA signaling network combined with lack of information on the quantitative kinetics of changes in internal node status, discrete dynamic modeling offers an optimal approach to provide insights and predictions regarding intracellular signaling mechanisms. The model provided here has been evaluated by new approaches: stable motif analysis, determination of the long-term states (attractors) of the system, and simulation of how stomatal apertures in the presence or absence of ABA are affected by in silico constitutive activation of each internal node. Our stable motif analysis indicates that the response to ABA is robust to variability in the timing of individual events and to variations in the initial state of 26 nodes, confirming a similar conclusion of the previous discrete dynamic model of ABA-induced closure [16, 141].

Our model successfully recapitulates well-characterized examples of impaired ABA sensitivity (Fig 3A and 3C) and increased ABA sensitivity (Fig 3B and 3D). In the cases with experimental documentation, 59 out of 76 simulations of individual node knockouts or constitutive activations in the presence of ABA (boldface nodes in Table 3 and 2 experiments reported here) and 13 of 24 simulations of constitutive activation of nodes in the absence of ABA (boldface nodes in Table 4) match experimental evidence. Moreover, for the limited number of cases for which experimental data are available, our model accurately captures the effect of knockout of an internal node on the status of a second internal node in the presence of ABA (S8 Table and the experiment reported here). Our model goes beyond the previous discrete dynamic model of ABA-induced closure [16] in a way that preserves the results in which the previous model agrees with experimental observations and improves the agreement of other results (S3 Text).

**Fig 6:**
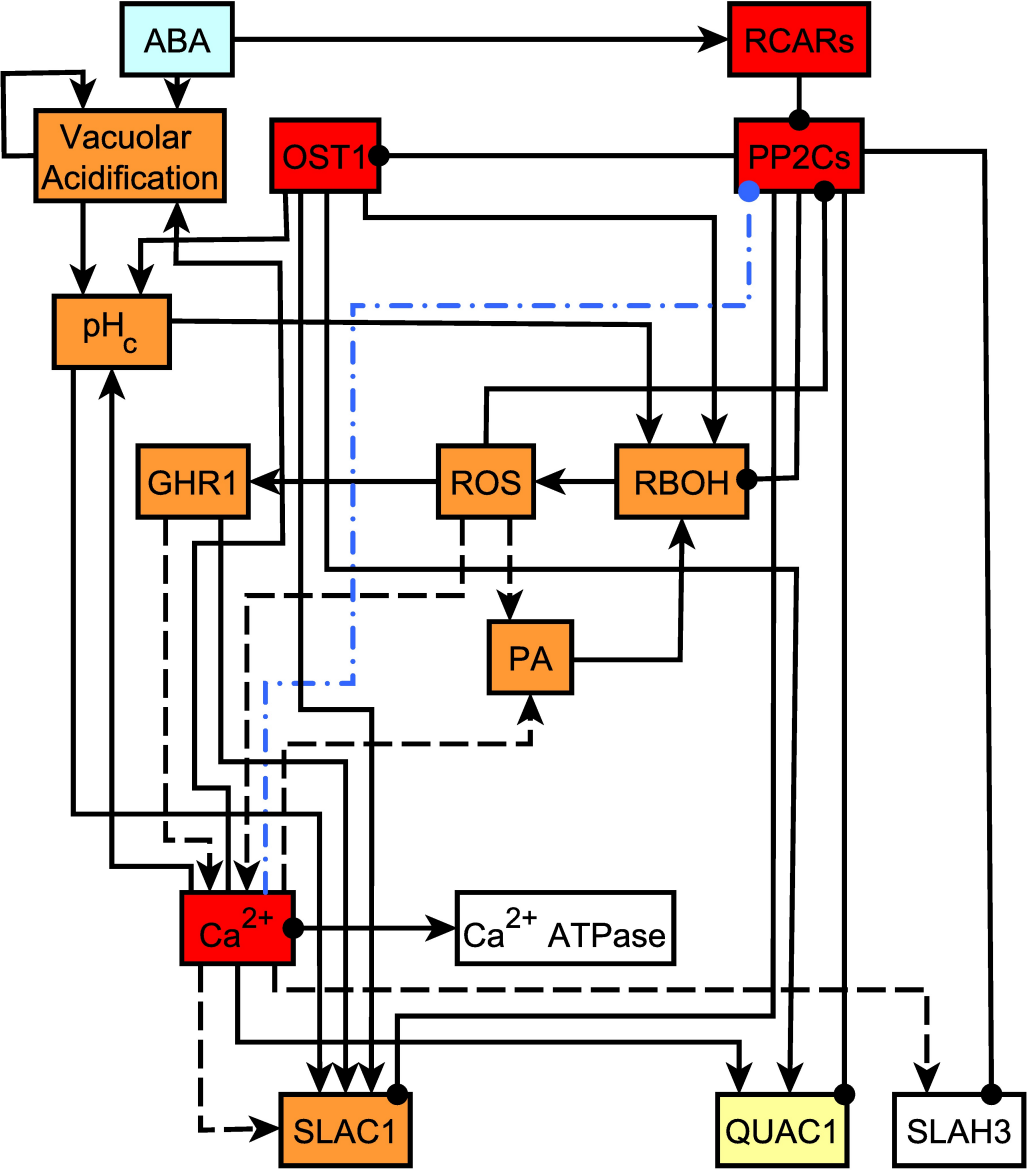
The subnetwork from abscisic acid (ABA) to the anion channels includes the core ABA signaling chain as well as the entire strongly connected component (SCC). As in Fig 1 and Fig 2, edges that end in an arrowhead signify activation and edges that end in a filled circle mean inhibition. For simplicity, certain linear chains, e.g., the Ca^2+^-dependent kinase (CPK)-mediated effect of Ca^2+^ on SLAC1 and SLAH3, have been compressed into single edges (shown with dashes). The 4 protein phosphatases 2C are merged into a single node (PP2Cs). ABA and RCARs are in the in-component, and SLAC1, QUAC1, and SLAH3 are in the out-component; the rest of the nodes are in the SCC. Nodes whose manipulation (knockout or, in the case of PP2Cs, constitutive activity) has been experimentally shown to cause decreased ABA sensitivity are marked with colored background (see Table 3). The colors indicate the response category for the simulated node manipulation: red means ABA insensitivity, orange marks reduced ABA sensitivity, and yellow means ABA hyposensitivity. The blue dash-dotted edge indicates our prediction that cytosolic Ca^2+^ inhibits the PP2Cs.

Our model recapitulates the core ABA signaling chain—ABA binding to receptors, which alleviates negative regulation by PP2Cs of the OST1 kinase, ultimately resulting in activation of anion channels—and places it in a broader context. Indeed, the subnetwork from ABA to the 3 anion channels, shown in Fig 6, is largely dependent on the ABA→RCARs—●PP2Cs—●OST1 chain (here—● refers to an inhibitory edge). This is consistent with the fact that RCARs or OST1 knockout or constitutive activation of the PP2Cs confers ABA insensitivity. This subnetwork also includes the whole SCC of the network (to which PP2Cs and OST1 belong), indicating the importance of feedbacks. Knockout of several other members of this subnetwork has also been observed to lead to decreased ABA sensitivity, indicated as red, orange, or yellow color in Fig 6. Notably, the process of vacuolar acidification, which, according to current knowledge, is independent of the core ABA signaling chain, is also a significant indirect contributor to anion channel activation. Furthermore, our prediction that Ca^2+^_c_ increase inhibits the PP2Cs (which yields an almost perfect model validation rate in the absence of ABA) suggests the existence of an additional feedback into the core ABA signaling chain.

It is important to note that, while our model is based on experimental evidence, this does not ipso facto guarantee the congruencies described above: (1) the evidence incorporated in the regulatory functions is at the level of specific edges (i.e., it only considers the most direct regulators of the target node) and so provides no control over pathway-level effects; (2) the network-level outcomes (the dynamic attractors) are emergent properties of the system; and (3) in construction of the regulatory functions, we found numerous instances of insufficiency of information and tested several alternatives before settling on the function that most faithfully recapitulated biological results.

Despite the excellent validation rate, some discrepancies with the literature remain. In some cases, this may be due to unresolved inconsistencies within the literature itself. For example, the calcium-dependent kinase CPK6 has been reported to be either active or inactive at resting Ca^2+^_c_ levels [26, 142]. Our model uses the assumption that CPK6 is always active, as is CPK23, while CPK3/21 is Ca^2+^ activated and then maintains its activity through autophosphorylation. Assuming that CPK6 and CPK23 are Ca^2+^ activated and thus follow the transient increase and decrease of Ca^2+^_c_ would yield the result that the anion channels’ activation and ultimately the stomatal aperture also increase and decrease with Ca^2+^_c._ This is not consistent with current knowledge. Making the assumption that the activity of all the Ca^2+^-activated CPKs is maintained through autophosphorylation (which is a known property of CPK kinases) would restore agreement and maintain all the current results of the model.

Another source of discrepancies between experimental and model results may have to do with cumulative percentage of closure (CPC) thresholds, although logically defined, imprecisely capturing the biological reality. For example, had the CPC range for “hyposensitivity” been extended to a slightly greater upper limit (23.97 instead of 23.92), 2 cases of type 1b error (MPK9/12 knockout and InsP3 knockout; Table 3) would have disappeared. Another reason for discrepancies is the fact that in a Boolean framework, either constitutive activity or constitutive inactivity of a source node must have the same meaning as the assumed wild-type unchanged state. In 2 cases of discrepancies regarding source nodes, namely, SCAB1 and GEF1/4/10, their knockout and their constitutive expression both were observed experimentally to lead to differential sensitivity to ABA [40, 83, 105]. This differential sensitivity would be captured by the model if it was assumed that ABA indirectly activates SCAB1 and indirectly inhibits GEF1/4/10. In general, the source nodes are promising candidates for experimental testing of whether they are in fact regulated by nodes of the ABA signaling network.

Incorporation of 3 states also would be able to separate the normal activity of a node from reduced activity due to an intervention and increased activity due to a different intervention. This increased resolution would capture differential sensitivity to the knockout or constitutive activation of a source node and would generally decrease the number of cases in which a simulated intervention leads to a close to wild-type response. While a Boolean regulatory function has a single activity threshold (between the OFF and ON state), a 3-state regulatory function involves 2 activity thresholds. The construction of such functions would require additional node-level, quantitative (dose-dependent) experimental information beyond what is currently available, but it is an important area for future work.

Many of the model’s predictions are unexpected, illustrating how models can aid human intuition. For example, impairment of ROS-activated Ca^2+^ channels in null mutants of GPA1 [51] would reasonably lead to the hypothesis that Ca^2+^_c_ response to ABA would be impaired in *gpa1* mutants; however, our model, which integrates all sources contributing to Ca^2+^_c_ elevation, made the counterintuitive prediction of wild-type Ca^2+^_c_ response, and this prediction was confirmed upon wet-bench assessment (Fig 4A and 4B). One particularly interesting model prediction is the existence of multiple states of the network (see S6 Table, S7 Table, S13 Table), all consistent with lack of closure in the absence of ABA. Such states may provide entry points for regulation by other stimuli to which multisensory guard cells also respond, including light, CO_2_ concentrations, and humidity. Indeed, attractor analysis of our previous model of light- and CO_2_-induced stomatal opening [20, 143] indicates the possibility of multiple long-term behaviors of nodes involved in K^+^ efflux in open stomata, consistent with the results reported here.

Our comprehensive analysis of the effect of node knockouts and constitutive activity in the presence of ABA allows the evaluation of implicit assumptions frequently encountered in the literature. Summarizing the results of Table 3 (augmented by our experiments reported here) in terms of whether an intervention (knockout or constitutive activation) yields an increased or decreased sensitivity to ABA yields several patterns (S15 Table). These patterns suggest that observing a differential ABA response for a node intervention (e.g., ABA insensitivity upon knockout) is not sufficient to infer (without testing) an opposite effect for the opposite intervention (e.g., to infer ABA hypersensitivity upon constitutive activation). Current results also show that simply observing state change of an internal node during ABA-induced closure is not sufficient to infer (without testing) a key role for this node in the process.

Our analysis also emphasizes the absence of data on the extent to which constitutive availability of a signaling metabolite or constitutive activation of a protein influences stomatal apertures either independently of or in combination with a closing signal such as ABA. A main reason is that in many cases, the genetic or pharmacological manipulation that would confer constitutive activation of an enzyme is simply unknown. Our model highlights that protein overexpression does not necessarily suffice to confer constitutive activity if the protein is posttranslationally regulated, e.g., by metabolites or other proteins. While it is well documented that experimental application of ROS, NO, or Ca^2+^ can each induce stomatal closure, the effects of activation of these internal nodes in combination with ABA exposure have received little attention. We tested 2 such instances (Ca^2+^_c_ and ROS, Fig 4C and 4D) and found agreement between simulated and experimental results. Our model makes explicit predictions for all such combinatorial effects (S8 Table) that can be tested in future experiments. Multiple stimuli are present in the natural environment; for example, both ABA and elevated CO_2_ induce ROS and NO production in guard cells [144]. Modeling of these and other combinatorial scenarios will provide information that ultimately can be applied to improve crop fitness [145] under the increasing threat of drought [146, 147] arising from elevated atmospheric concentrations of CO_2_ and other greenhouse gases. Finally, our approach illustrates the value of discrete dynamic modeling to identify both robustness and vulnerabilities of a complex, nonlinear system, which typifies many biological phenomena.

## Materials and methods

### Network construction

In the network, components of the ABA signal transduction pathway are represented as nodes and pairwise interactions are denoted by edges. Most edges in this network are directed to indicate the orientation of regulation or information transfer. The exceptions are the edges that represent binding among the subunits of the heterotrimeric G protein (GPA1, AGB1, and AGG1), which are undirected (symmetrical) and are considered bidirectional in the network analysis. A positive edge (terminating in an arrowhead) indicates positive regulation or information transfer from the starting node (regulator) to the end point of the edge (the target). A negative edge (terminating in a black circle) indicates the repression or inhibition of the target by the regulator.

Nodes in the ABA signal transduction network model originate from the known components reported in the literature through 2015. From over 120 published articles, we identified evidence of interactions or causal effects between signaling components (summarized in S1 Table). This information comes from 2 major types of experimental evidence. Protein–protein interactions discovered through bimolecular fluorescence complementation (BiFC) and/or yeast two-hybrid experiments; assays of phosphorylation status indicative of relationships between kinases, phosphatases, and their substrates; and assays wherein coexpression of an ion channel and an upstream regulator in *Xenopus* oocytes results in altered ion channel behavior indicate a direct interaction between 2 components. For example, the OST1 kinase activates SLAC1 anion channels by direct phosphorylation [148, 149]; accordingly, the network contains a direct positive regulatory edge from OST1 to SLAC1. By contrast, a genetic mutation or pharmacological treatment of a component that alters the status of a downstream component indicates that there is a causal relationship between the 2 components of the biological system, but because it does not assess physical interaction, it does not reveal whether the relationship is direct or indirect. We considered the existence of evidence of physical interaction (or lack thereof) to categorize these relationships as direct or indirect. For example, exogenous Ca^2+^ application indirectly leads to cytosolic alkalinization (pH_c_ increase); accordingly, the network includes an indirect positive regulatory edge between Ca^2+^_c_ and pH_c_.

To keep the network parsimonious, indirect causal relationships that could be explained by a path of direct edges (i.e., a sequence of direct regulatory relationships) were considered for elimination. Edge elimination was performed by employing a network reduction technique called binary transitive reduction implemented in the software NetSynthesis [140]. In each case, we consulted the biologically relevant literature to make sure that there is no evidence of a direct interaction between the participants in the causal relationship that was a candidate for elimination.

In some cases, the literature indicates the effect of genetic mutation or pharmacological treatment of a component on a process rather than on a target node, which results in a causal relationship among 3 nodes, e.g., “X promotes the process through which A induces B.” Following [16] and [140], we used the most parsimonious interpretation of this relationship. For example, if it is known that A activates X directly or indirectly, we inferred that X activates B. An example of a signed version of this inference is ABI1—● pH_c_, based on the evidence that (i) ABI1 inhibits ABA-induced pH_c_ increase [77] and (ii) ABA, via its binding to RCARs, leads to the inhibition of the phosphatase activity of ABI1 [21, 22]. Conversely, if it is known that X activates B through a direct interaction, we inferred that A activates X. An example of such an inference is ROS→NO, based on the evidence that (i) in *nia1/2* double knockout plants, ROS fails to induce NO production and (ii) NIA1/2 are the enzymes that catalyze the production of NO from NADPH [56].

The network is provided as a graphml file, readable into Cytoscape, in the GitHub repository https://github.com/krhyyme/ABA-Boolean-Network-Model.

### Network analysis

Network analysis was performed using the Python graph analysis library NetworkX [150]. Node degree quantifies the number of edges that belong to a particular node. The in-degree of a node is the number of edges oriented toward it. The out-degree is the number of edges that originate from it. Undirected edges are interpreted as bidirectional. Nodes with an in-degree of zero (no incoming edges) are called source nodes and nodes with an out-degree of zero (no outgoing edges) are called sink nodes.

A path in a network is a sequence of adjacent edges. The SCC of a network represents the subnetwork whose nodes are connected by paths in both directions. Thus, every pair of nodes A and B in an SCC has both an A→→B path and a B→→A path. The in-component of a network is the set of nodes that can reach the SCC through paths, and the out-component is the set of nodes that can be reached from the SCC through paths.

The betweenness centrality of a node *i* quantifies the node’s participation in directed paths that start and end at nodes other than *i*. We used the betweenness centrality measure based on shortest paths (paths with the smallest length). Specifically, the betweenness centrality of node *i* is $g(i)=\sum_{j\neq i\neq k}\frac{n_{jk}\;(i)}{n_{jk}}$, where *n*_*jk*_ is the number of shortest paths that start from node *j* and end in node *k*, both of which are different from node *i*, and *n*_*jk*_*(i)* is the number of shortest paths that start from node *j* and end in node *k* and contain node *i*.

### The initial state of each node in the dynamic model

In a Boolean model, each node is characterized by 2 possible states: ON or 1 (interpreted as a higher-than-threshold abundance or activity) and OFF or 0 (lower-than-threshold abundance and activity). For example, the ON state of the Ca^2+^_c_ node indicates that the concentration of Ca^2+^_c_ is sufficiently higher than the resting level, such that it can activate CPKs and other downstream targets. Biologically known initial states are implemented as ON or OFF. For example, secondary messengers known to be produced in response to ABA (e.g., ROS, NO) and signaling proteins known to be activated in response to ABA (e.g., RCARs, OST1) are assumed to be OFF in the initial state [1, 3, 98–101]. The PP2C protein phosphatases are assumed to be initially ON [103]. The initial states of the 26 nodes with no such information are randomly chosen. The initial state of each node is summarized in S6 Table.

### Construction of the regulatory function of each node

The future state of a node is determined by the current state of its directly upstream regulators and is expressed as a Boolean regulatory function. A node with a single direct regulator is characterized by one of 2 types of single variable Boolean function: identity, used for positive regulators and meaning that the target is adopting the state of the regulator, and negation, used for negative regulators and meaning that the target adopts the opposite state as the regulator. Negation is represented by the Boolean operator “not.” For nodes that have more than one direct regulator, the “or” operator is used if any of these regulators can independently activate the target; the “and” operator is used if all direct regulators are needed for activation. The choice of the most appropriate operator to use is determined by experimental evidence. If there is evidence that knockout of one regulator prevents a node from being activated, then the “and” rule is used to describe the set of regulators to which this behavior applies. For example, as ABA does not induce pH_c_ increase in *abi1-*dominant mutants, we include “and not ABI1” in the regulatory function of pH_c_. If it is shown that simultaneous absence of a pair or set of direct regulators is required to prevent activation, then the “or” rule is used. In cases where adequate information is not available, “or” can also be used as the presumptive rule [151]. Below, we indicate the regulatory function of PA as an example. In this function, the node states are represented by the node names and an asterisk is used to denote the future state of a node:

$${\mathrm{PA}}^{*}=\;\mathrm{PC}\;\mathrm{and}\;(\mathrm{PLD}\delta \;\mathrm{or}\;\mathrm{PLD}\alpha )\;\mathrm{or}\;\mathrm{DAG}\;\mathrm{and}\;\mathrm{DAGK}$$

PC is the substrate required by PLDα or PLDδ for PA production. DAG, a product of PLC activity, can be converted into PA by DAGK-mediated phosphorylation; thus, PC combined with either of the indicated PLDs or the combination of DAG and DAGK is sufficient for PA production and thus for the presence of PA after a time delay (PA*).

AGB1 and AGG3 are not included in the dynamic model, as there is no current evidence that they influence ABA-induced stomatal closure other than via GPA1, with which they share an undirected edge [152, 153]. ROP10 is also not included in the dynamic model: there is no current evidence that it influences ABA-induced closure; hence, it has no outgoing edges and therefore exerts no control on model outcome.

Generally, the regulatory function of a node contains the node itself if and only if there is a self-loop in the network. There are 3 exceptions to this rule in the model. For pH_c_ and S1P/PhytoS1P production, transient increases are observed following ABA exposure [47, 70] but there is no causal or mechanistic understanding of the negative feedback regulation; hence, the self-regulation is included in the network but not included in the regulatory functions. In the case of vacuolar acidification, we assume the maintenance of an already-initiated state; hence, the regulatory function contains the node itself.

### Implementation of node update in the dynamic simulations

Each model simulation starts from an initial condition (initial state of the network elements) corresponding to open stomata, specified in S6 Table and transitions through several states until it reaches a set of states in which it settles down, known as an attractor. An attractor can be a fixed point (steady state) or a set of states that repeat indefinitely (a complex attractor), such as an oscillation. We simulate successive events by a stochastic update algorithm. Update of a node’s state means that its regulatory function is evaluated and the function’s output is adopted as the new state of the node. Because signaling networks contain a diverse set of components that act at many different timescales, asynchronous update algorithms are most appropriate for modeling signal transduction pathways [154], particularly when, as in the present case, relative timescales of internal processes are largely unknown. We implemented the model using the Python software library BooleanNet [155]. We used the stochastic asynchronous update algorithm, in which the nodes are updated in a randomly selected order in each time step. Due to the stochasticity introduced by the update method and by the initial node states, we used 2,500 replicate simulations. This number of replicates ensures that the margin of error of the percentage of closure is less than 3%, with 95% confidence. In each simulation, we followed the evolution of the model for 30 time steps (rounds of update), which was sufficient to reach an attractor in each modeled scenario. The outcome of the model is summarized as the percentage of simulations in which the node Closure is in the state 1 at each time step, which we refer to as the percentage of closure. A sample script using the BooleanNet library and the list of Boolean regulatory functions in the form readable by the script are provided in the GitHub repository https://github.com/krhyyme/ABA-Boolean-Network-Model.

### Stable motif analysis

A stable motif is a special kind of SCC that can maintain a specific steady state of its constituent nodes regardless of the state of the rest of the network [96]. The stable motifs of a Boolean network determine its attractors: one can uniquely associate sequences of stable motifs (stabilized in the order given by the sequence) to each attractor. We use the method introduced in [96], which maps the identification of stable motifs into finding SCCs with certain identifiable properties in an expanded representation of the Boolean network (which includes the network’s Boolean functions as part of the network structure). A more detailed explanation of the criteria for identifying stable motifs and of the procedure for creating the expanded representation of the Boolean network can be found in [96]. A Java library that implements the stable motif analysis and attractor determination is available in the GitHub repository https://github.com/jgtz/StableMotifs.

### Simulated knockout and constitutive activity

We simulate knockout of a node by setting its state as OFF (0) in the initial condition and maintaining this state throughout the simulation. We simulate constitutive activity of a node by setting its state as ON (1) in the initial condition and maintaining this state throughout the simulation. Thus the knocked-out or constitutively active nodes are not updated.

### Definition of the response categories in case of simulated knockout or constitutive activity

In the most severe case of defect, the percentage of closure stays exactly or very close to 0% for the entire duration of the simulation, similar to the wild-type simulations in the absence of ABA. It can be concluded that knockouts in this category, e.g., *ost1*, lead to insensitivity to ABA. A less severe defect is indicated by a percentage of closure that stabilizes at a value between 0% and 100%; we call this category “reduced sensitivity” to ABA.

Following [16], we define the cumulative percentage of closure (CPC) as the summed fraction of simulations in which Closure = 1 over the 30 time steps. Thus, CPC ranges from 0 (if the percentage of closure were 0% at every time step) to 30 (if the percentage of closure were 100% at every time step). Comparison of the CPC corresponding to the knockout or constitutive activity of a node with the CPC of wild type can determine whether the knockout led to a decrease in sensitivity or, possibly, an increase. To determine whether a measured difference in CPC values is significant, especially in light of the fact that 2,500 simulations represent a sampling of the system’s total possible trajectories, we benchmark with the fact that certain cases of simulated knockout or constitutive activity are equivalent to a wild-type simulation. Specifically, simulated constitutive activity of any of 20 source (unregulated) nodes that are assumed to be ON in the model is equivalent with a wild-type simulation, as is simulated knockout of either of the 2 source nodes that are assumed to be OFF in the model (see Table 1 and S2 Text for the identity of these nodes). We use these 22 ensembles of 2,500 simulations to delineate the CPC range of wild-type equivalent simulations. Interventions that lead to a higher CPC value than the highest value in the wild-type equivalent range (24.08) are classified as ABA hypersensitive, and interventions that lead to eventual closure but with a lower CPC value than the lowest value in the wild-type equivalent range (23.93) are classified as ABA hyposensitive. Interventions that lead to a CPC value within the wild-type equivalent range are classified as close to wild type. In the absence of ABA and initial activity of the PP2C protein phosphatases, the threshold between close to wild type and slightly increased response is CPC = 0.002 (Table 4). In the absence of ABA and initial inactivity of the PP2C protein phosphatases, the threshold between slightly decreased response and close to wild type response is CPC = 9.66 and the threshold between close to wild type response and increased response is CPC = 10.62 (S12 Table).

### Experimental testing of model predictions

Wild-type *Arabidopsis* of the Columbia (Col) accession and previously described [156] *gpa1-3* and *gpa1-4* null mutants in the Col background were grown in a growth chamber with 8-h light/16-h dark cycles with a light intensity of 150 μmol/m^2^s and temperatures of 21°C during the light period and 19°C during the dark period. To measure Ca^2+^_c_ in response to ABA, epidermal peels from fully expanded leaves from 4-week-old T2 plants stably transformed with a YC3.6 Ca^2+^_c_ reporter construct [157] driven by the GC1 guard cell-specific promoter [158] were imaged using a Zeiss LSM510 confocal microscope as described [159]. Epidermal peels were mounted on a coverslip chamber with medical adhesive (Hollister, Libertyville, IL, USA) and incubated with 20 mM KCl, 50 μM CaCl_2_, 5 mM Mes-Tris, pH 6.15 under illumination (150 μmol/m^2^ s white light) for 3 hours. The coverslip chamber was placed on the stage of the confocal microscope. The YC3.6 fluorescence signal from individual guard cells was observed by a laser scanning confocal microscope (LSM 510 Meta; Carl Zeiss, Thornwoood, NY, USA) using a C-Apochromat 40X/1.2 W corr water immersion objective with the 458-nm line of the argon laser for excitation of YC3.60 and 484–505 nm and 526–536 nm for the detection of CFP and Venus (for FRET) of YC3.60, respectively. After 5 minutes, epidermal peels were treated with 10 μM ABA, then images were taken at 10-second intervals for 50 minutes. The ratios of Ca^2+^-dependent (FRET/CFP) fluorescence intensities were calculated using ImageJ software. Ca^2+^_c_ transients were defined as increases that were at least 0.1 above the baseline, following the method of Ye et al. [160].

To measure the effects of Ca^2+^ and ROS provision on ABA-induced stomatal closure, we modified a previously established protocol [161]. Before initiation of the light cycle, fully expanded leaves from 4–5-week-old plants were excised. Abaxial epidermes were peeled off using forceps and incubated in solution (20 mM KCl, 5 mM MES/KOH, pH 6.15) under white light (175 ± 25 μmol m^-2^ sec^-1^) for 3 h to induce stomatal opening. Different treatments, i.e., solvent control ethanol (0.1%), ABA (final concentration at 2 μM), CaCl_2_ (final concentration at 50 μM), and H_2_O_2_ (final concentration at 0.1 μM) were then added to the solutions, as indicated. The abaxial epidermes were imaged at the indicated time points (1 h and 2 h) by light microscopy (Nikon Diaphot 300) and attached camera (Nikon E990). Stomatal apertures were measured by analysis of the digital images using ImageJ (National Institutes of Health, USA). Experiments were performed blinded and each experiment was repeated 3 times, with 64 stomatal apertures measured for each treatment.

### Calculation of the model’s validation rate

In the 97 cases with prior experimental documentation of the effect of a node’s knockout or constitutive activation on the closure response (Table 3 and Table 4), 57 out of 73 simulations of individual node knockouts or constitutive activations in the presence of ABA (boldface nodes in Table 3) and 13 of 24 simulations of constitutive activation of nodes in the absence of ABA (boldface nodes in Table 4) match experimental evidence. Our model accurately captures the effect of knockout of an internal node on the status of a second internal node in the presence of ABA in all 12 extant experiments reported in S8 Table. Thus, the model’s validation rate with prior results is 82/109 = 0.75. The 3 new experiments reported here increase both the cases of agreement and the denominator by 3. The hypothesis that Ca^2+^ increase leads to the inactivation of the four PP2Cs yields 10 new cases of agreement. Thus, the augmented model’s validation rate is 95/112 = 0.85.

## Supporting information

S1 Data Data supporting Fig 3A–3D, Fig 4B–4D and Fig 5A and 5B. (XLSX)

S1 Table Summary of interactions and regulatory relationships collected from more than 120 articles in the literature. (DOCX)

S2 Table List of edges in the ABA-induced closure network. (DOCX)

S3 Table The full names of the abbreviated node names in the ABA-induced closure network. (DOCX)

S4 Table Composition of the in-component, strongly connected component, and out-component of the network. (DOCX)

S5 Table Distribution of the nodes of the ABA signal transduction network according to 3 centrality measures: in-degree, out-degree, and betweenness centrality. (DOCX)

S6 Table Summary of the long-term dynamics (attractors) of network nodes in the sustained presence or absence of ABA compared to the assumed initial condition of open stomata. (DOCX)

S7 Table The system’s 17 attractors in the absence of ABA. (DOCX)

S8 Table Full list of the effect of simulated node manipulations (knockout or constitutive activity) in the presence of ABA and comparison with the closest experimental results. (DOCX)

S9 Table The attractors corresponding to simulated *rboh* knockout and disrupted K^+^ efflux, respectively, in the presence of ABA. (DOCX)

S10 Table Twelve representative cases of consistency between the experimentally observed and simulated effect of an internal node’s knockout on a second internal node in the presence of ABA. (DOCX)

S11 Table Full list of the effect of simulated constitutive activity (or external supply) of nodes in the absence of ABA and comparison with the closest experimental results. (DOCX)

S12 Table Initial inactivity of the PP2C protein phosphatases (HAB1, PP2C, ABI1, and ABI2) significantly affects the simulated response to constitutive activity (or external supply) of nodes in the absence of ABA. (DOCX)

S13 Table The system’s 15 attractors in the absence of ABA if assuming that the PP2C protein phosphatases are initially inactive. (DOCX)

S14 Table Comprehensive summary of the effect of node activations in the absence of ABA in 2 alternative scenarios: initial inactivity of ABI1 and ABI2 and assuming that Ca^2+^_c_ can inhibit all 4 PP2C protein phosphatases. (DOCX)

S15 Table The effect of node knockouts and node constitutive activity in the presence of ABA falls into several patterns shared by multiple nodes. (DOCX)

S1 Text Complete biological description of the ABA-induced stomatal closure network. (DOCX)

S2 Text Detailed justification of the sustained state or Boolean regulatory function of each node in the model. (DOCX)

S3 Text Comparison of the predicted effect of node knockout in the presence of ABA in the Li et al. 2006 [16] article and this work. (DOCX)

## Abbreviations

ABA: abscisic acid
ABI1: ABA-insensitive 1
ABI2: ABA-insensitive 2
CA: constitutive activity
Ca^2+^_c_: cytosolic Ca^2+^
CaIM: Ca^2+^_c_ influx through the membrane
CIS: Ca^2+^_c_ release from internal stores
CPC: cumulative percentage of closure
CPK: Ca^2+^-dependent kinase
CFP: cyan fluorescent protein
Col: Columbia accession
EtOH: ethanol
GPA1: Gα subunit
HAB1: hypersensitive to ABA 1
KO: knockout
MPK: mitogen activated protein kinase
OST1: OPEN STOMATA 1
PA: phosphatidic acid
PC: phosphatidylcholine
PEPC: phosphoenolpyruvate carboxylase
pH_c_: cytosolic pH
PI3P5K: phosphatidylinositol 3-phosphate 5-kinase
PP2C: protein phosphatase 2C
PP2CA: protein phosphatase 2CA
PYR1: pyrabactin resistance 1
PYL: pyrabactin resistance 1-like
RCAR: regulatory component of ABA receptor
ROS: reactive oxygen species
SCC: strongly connected component
SLAC1: slow anion channel-associated 1
YC 3.6: Yellow Cameleon 3.6
YFP: yellow fluorescent protein
