# Supplementary material for: A new discrete dynamic model of ABA-induced stomatal closure predicts key feedback loops
Source: PLoS Biol. 2017 Sep 22;15(9):e2003451. doi: 10.1371/journal.pbio.2003451 (PMC5627951; doi:10.1371/journal.pbio.2003451)
Supplement: S1 Table — (DOCX) [file pbio.2003451.s002.docx]

**S1 Table. Summary of interactions and regulatory relationships collected from more than 120 articles in the literature.**

The entries are grouped into three categories: 1. Pairwise interactions and regulatory relationships represented as edges in our network, 2. Pairwise regulatory relationships represented as paths in our network, and 3. Node to process regulatory relationships represented as (potentially multiple) paths in our network. The first column indicates the regulator node, the second column indicates the target node or target process, the third column indicates the relationship category, the fourth column indicates the effect of the interaction, i.e. whether the relationship is positive (“promotes”) or negative (“inhibits”), the fifth column indicates the interaction type (direct or not). We use the species shorthand notations A.t. (*Arabidopsis thaliana*), V.f. (*Vicia faba* L.), C.c. (*Commelina communis*). X. (Xenopus oocytes), N. (*Nicotiana*), V.r. (*Vigna radiata*), a.c.l. (animal cell lines).

| **Node A** | **Node/**  **Process B** | **Cat.** | **Int. effect** | **Int. type** | **Spe-cies** | **Ref** | **Comments/notes** |
| --- | --- | --- | --- | --- | --- | --- | --- |
| 8-Nitro-cGMP | ADPRC | 1 | Promotes | Not direct | A.t. | [1] | 8-nitro-cGMP indirectly activates ADPRc. |
| ABA | RCARs | 1 | Promotes | Direct | A.t. | [2-5] | RCARs are soluble ABA receptors. These proteins directly bind ABA. RCARs also mediate ABA-mediated stomatal closure. |
| ABA | PEPC | 1 | Inhibits | Not direct | V.f | [6] | ABA inhibits PEP carboxylase (PEPC) activity in guard cells. |
| ABA | PI3P5K | 1 | Promotes | Not direct | A.t. | [7] | ABA indirectly promotes PI3P5K activity. |
| ABA | SPHK1/2 | 1 | Promotes | Not direct | A.t. | [8-11] | SPHK1 and SPHK2 are sphingosine kinases. Guard cell sphingosine kinase activity is stimulated by ABA in Arabidopsis. |
| ABA | AtRAC1 | 1 | Inhibits | Not direct | A.t. | [12] | ABA treatment causes inactivation of AtRAC1 and promotion of actin reorganization in guard cells. |
| ABA | Malate | 1 | Inhibits | Not direct | C.c. | [13] | ABA negatively regulates malate concentration by inducing malate breakdown. |
| ABH1 | CaIM | 1 | Inhibits | Not direct | A.t. | [14] | The *abh1* mutant shows greater increases in cytosolic Ca^2+^ in response to ABA compared to the wild type. We parsimoniously interpret this as ABH1 inhibiting the Ca^2+^ permeable channels. |
| ABI | AtRAC1 | 1 | Promotes | Not direct | A.t. | [12] | Both ABI and AtRAC1 are negative regulators of stomatal closure. The dominant negative form of AtRAC1 was able to recover stomatal closure in *abi1-1*(dominant negative mutant). We assume that ABI1 promotes AtRAC1. |
| ABI | SLAH3 | 1 | Inhibits | Direct | A.t. | [15] | ABI1 inhibits CPK-mediated activation of SLAH3. |
| ABI1 | SLAC1 | 1 | Inhibits | Direct | A.t., X. | [16] | ABI1 physically interacts with SLAC1; ABI1 inhibits SLAC1 by dephosphorylation. |
| ABI1 | OST1 | 1 | Inhibits | Direct | A.t. | [3, 17, 18] | ABI1 physically interacts with OST1 and inhibits its kinase activity. |
| ABI2 | OST1 | 1 | Inhibits | Direct | A.t. | [18] | ABI2 physically interacts with OST1 and inhibits its kinase activity. |
| HAB1 | OST1 | 1 | Inhibits | Direct | A.t. | [18] | HAB1 physically interacts with OST1 and inhibits its kinase activity. |
| ABI2 | GHR1 | 1 | Inhibits | Direct | A.t.,  X. | [19] | ABI2 physically interacts with GHR1.  GHRI-mediated activation of SLAC1 is inhibited by ABI2 but not by ABI1 in Xenopus oocytes. |
| ABI2 | SLAC1 | 1 | Inhibits |  | X. | [16, 20] | ABI2 inhibits SLAC1 activity. |
| ABI2 | SLAC1 | 1 | Inhibits | Direct | A.t. | [20] | ABI2 inhibits CPK-mediated activation of SLAC1. |
| Actin reor-ganization | CaIM | 1 | Promotes | Not direct | V.f | [21] | Actin reorganization promotes stretch-activated Ca^2+^ channels. |
| ADPRc | cADPR | 1 | Promotes | Direct |  |  | Enzyme and product relationship |
| AGB1 | AGG3 | 1 | Binds | Direct | A.t. | [22] | AGB1 physically interacts with AGG3 in yeast (Y2H) and in plant BiFC). AGB1 and AGG3 function as a heterodimer. |
| AnionEM | Depolarization | 1 | Promotes | Direct | V.f | [23] | Anion efflux across the plasma membrane promotes plasma membrane depolarization. |
| AnionEM | Malate | 1 | Inhibits | Direct | A.t. | [24, 25] | Anion efflux (AnionEM) negatively regulates intracellular malate concentration by releasing malate from the cytosol. |
| AnionEM | H_2_O Efflux | 1 | Promotes | Not direct | A.t. |  | The efflux of anions is required for H_2_O efflux. |
| ARP2/3 complex | Actin reorganiza-tion | 1 | Promotes | Direct | A.t. | [26, 27] | ARP2 (Actin Related Protein C2) encodes the ARPC2 subunit of the ARP2/3 complex. Purified, active Arp2/3 complex binds to the sites of existing actin filaments and nucleates new ‘daughter’ filaments. The *arp2* mutant is deficient in ABA- and CaCl_2_-induced stomatal closure. This mutant does not show actin reorganization in response to ABA in guard cells. Upon addition of cytochalasin D (which induces depolymerization of actin filaments) *arp2* mutant guard cells show similar ABA-mediated stomatal response as wild type. |
| AtMPK9 (MPK9/12 node) | AtMPK9 | 1 | Promotes | Direct | A.t. | [28] | AtMPK9 maintains its activity through auto-phosphorylation. |
| AtRAC1 | Actin reorganiza-tion | 1 | Inhibits | Not direct | A.t. | [12] | Expression of a dominant-positive mutant of AtRAC1 inhibits ABA-induced actin reorganization whereas expression of a dominant-negative mutant of AtRAC1 promotes actin reorganization in the absence of ABA. |
| AtSPP1 | S1P | 1 | Inhibits | Direct | A.t. | [29] | SPP1, a long-chain base 1-phosphatase, has been implicated as a negative regulator of S1P accumulation in plants. |
| Ca^2+^ | pH_c_ | 1 | Promotes | Not direct | A.t. | [30] | Exogenous Ca^2+^ induces cytosolic alkalization. We incorporate this by a positive edge from Ca^2+^_c_ to pH_c_. |
| Ca^2+^ | TCTP | 1 | Promotes | Direct | A.t. | [31] | Ca^2+^ promotes the interaction between AtTCTP (TCTP node) and microtubules. We assume that Ca^2+^ promotes the activity of TCTP. |
| Ca^2+^ | Ca^2+^ ATPase | 1 | Promotes | Direct | A.t. | [32] | Ca^2+^ promotes Ca^2+^ ATPase activity. |
| Ca^2+^ | KEV | 1 | Promotes | Not direct | V.f | [33] | Calcium induces K^+^ release through K^+^-permeable channels in the tonoplast. |
| Ca^2+^ | H^+^ ATPase | 1 | Inhibits | Direct | V.f | [34] | The H^+^ ATPase is inhibited by cytosolic calcium concentration increase. |
| Ca^2+^ | Depolarization | 1 | Promotes | Direct | A.t. | [35] | Ca­­_c_^2+^ influx across the plasma membrane promotes plasma membrane depolarization. |
| Ca^2+^ | CPK3 and CPK21 (CPK3/21 node) | 1 | Promotes | Direct | A.t. | [36] | Cytosolic calcium activates CPK3 and CPK21. |
| Ca^2+^ | PLDα1 (PLDα node) | 1 | Promotes | Direct | A.t. | [37] | Ca^2+^_c_ is required for activation of PLDα1 and also promotes translocation of PLDα1 to the plasma membrane and tonoplast where lipid substrates are available for the enzyme. |
| Ca^2+^ | PLC | 1 | Promotes | Direct | A.t. | [38] | Ca^2+^ is required for PLC activity. |
| Ca^2+^ | V-ATPase | 1 | Promotes | Not Direct | A.t. | [39] | Ca^2+^_c_ has been implicated as a positive regulator of the V-ATPase. |
| Ca^2+^ ATPase | Ca^2+^ | 1 | Inhibits | Direct | A.t. | [32] | The Ca­­^2+^ ATPase pumps Ca­­^2+^ from the cytosol to the apoplast. |
| cADPR | CIS | 1 | Promotes | Not Direct |  | [40, 41] | cADPR is an important signaling molecule leading to calcium release from internal stores. |
| CaIM | Ca^2+^_c_ | 1 | Promotes | Direct |  | [42-45] | CaIM (Ca­­^2+^ Influx across the plasma Membrane) causes increase of cytosolic Ca^2+^. |
| cGMP | 8-nitro-cGMP | 1 | Promotes | Direct | A.t. | [1] | cGMP is the substrate for 8-nitro-cGMP synthesis. |
| CIS | Ca^2+^_c_ | 1 | Promotes | Direct | V.f, C.c. | [46, 47] | CIS causes increase of cytosolic Ca^2+^. |
| CPK21 | SLAC1 | 1 | Promotes | Direct | X., A.t. | [20] | CPK21 directly interacts with SLAC1 and stimulates its activity in Arabidopsis and in Xenopus oocytes. |
| CPK23 | SLAC1 | 1 | Promotes | Direct | X.,  A.t. | [20] | CPK23 directly interacts with SLAC1 and stimulates its activity in Arabidopsis and in Xenopus oocytes. |
| CPK23 | SLAH3 | 1 | Promotes | Direct | X. | [15, 36] | CPK23 interacts with SLAH3. CPK23 promotes SLAH3 activity. |
| CPK3/21 | SLAC1 | 1 | Promotes | Direct | A.t., X. | [20, 36, 48] | Both CPK3 and CPK21 physically interact with SLAC1 and activate SLAC1 activity. |
| CPK3/21 | SLAH3 | 1 | Promotes | Direct | X. | [15, 36] | CPK3 and CPK21 interact with SLAH3. |
| CPK3/21 | CPK3/21 | 1 | Promotes | Direct | A.t. | [49] | CPK3 and other CPKs maintain their activity by auto-phosphorylation. |
| CPK6 | SLAC1 | 1 | Promotes | Direct | X.,  A.t. | [36, 48, 50] | CPK6 physically interact with SLAC1 and activates SLAC1 activity |
| CPK6 | SLAH3 | 1 | Promotes | Direct | X. | [36] | CPK6 interacts with SLAH3. CPK6 promotes SLAH3 activity. |
| DAG | PA | 1 | Promotes | Direct |  | [51] | DAG can be converted into PA by DAGK-mediated phosphorylation. |
| DAGK | PA | 1 | Promotes | Direct |  | [51] | DAG can be converted into PA by DAGK-mediated phosphorylation. |
| Depolarization | KOUT | 1 | Promotes | Direct | A.t. | [52] | K^+^ efflux through outwardly rectifying K^+^ channels requires membrane depolarization. |
| ERA1 | ROP10 | 1 | Promotes | Not direct | A.t. | [53] | Localization of ROP10 to the plasma membrane is required ROP10 function. Functional ERA1 is required for plasma membrane localization of ROP10 indicating that ERA1 positively affects ROP10 function. |
| ERA1 | CaIM | 1 | Inhibits | Not direct | A.t. | [54] | At low ABA concentrations, greater increases in cytosolic Ca^2+^ and stomatal closure activation were observed in the *era1-2* loss-of-function mutant in comparison to the wild type. These observations suggest that ERA1 inhibits cytosolic Ca^2+^ influx. |
| GAPC1 and GAPC2 (GAPC1/2) | PLDδ | 1 | Promotes | Direct | A.t. | [55] | GAPC1 and GAPC2 interact with PLDδ which in turn increases enzyme activity of PLDδ. |
| GCR1 | GPA1 | 1 | Inhibits | Direct | A.t. | [56] | GCR1 interacts with GPA1. Loss-of-function *gcr1* mutants show hypersensitivity in S1P induced stomatal responses, in contrast *gpa1* loss-of-function mutants show insensitivity. These findings suggesting that GCR1 is a negative regulator of GPA1. |
| GEF1, GEF4, GEF10 (GEF1/4/10 node) | ROP11 | 1 | Promotes | Direct | A.t. | [57, 58] | GEF1, GEF4, and GEF10 interact with ROP11.  Guanine nucleotide exchange factors (GEFs) regulate the function of ROPs. *gef1 ge4* double mutant and *gef1/4/10* triple mutants are hypersensitive to ABA-mediated stomatal responses. The *gef* double and triple mutants do not show different aperture size in the absence of exogenous ABA. |
| GHR1 | SLAC1 | 1 | Promotes | Direct | A.t., X. | [19] | GHR1 physically interacts with SLAC1 and phosphorylates SLAC1. Upon coexpreesion in Xenopus oocytes, GHR1 physically interacts with SLAC1 and phosphorylates SLAC1, which in turn activates SLAC1. *ghr1* mutation impairs the ABA- and ROS activation of anion channels. |
| GPA1 | AGB1 | 1 | Binds | Direct, undirected | A.t. | [59] | GPA1 interacts with AGB1, which facilitates the formation of the heterotrimeric G-protein complex. |
| GPA1 | PLDα1 | 1 | Promotes | Direct | A.t. | [60] | The GTP bound form of GPA1 is required for PLDα to be active. |
| GTP | cGMP | 1 | Promotes | Direct | A.t. | [61] | GTP is the substrate for cGMP production. |
| H^+^ ATPase | Depolarization | 1 | Inhibits | Direct | A.t. | [62, 63] | H^+^ ATPase activity negatively regulates plasma membrane depolarization. |
| InsP3 | CIS | 1 | Promotes | Not direct | C. c. | [46] | InsP3 can release calcium from internal stores. |
| InsP3 | InsP6 | 1 | Promotes | Direct |  | [64] | Triple phosphorylation of InsP_3_ can yield InsP_6_. |
| InsP6 | CIS | 1 | Promotes | Not direct | V.f | [47] | InsP6 can release calcium from internal stores. |
| K^+^ efflux | H_2_O efflux | 1 | Promotes | Not direct | A.t. | [52] | K^+^ efflux is required for H_2_O efflux. |
| K^+^ efflux | Depolarization | 1 | Inhibits | Direct | A.t. | [52] | K^+^ efflux across the membrane negatively regulates plasma membrane depolarization. |
| KEV | K^+^ efflux | 1 | Promotes | Direct | V.f | [33] | Sustained efflux of K^+^ from the guard cell requires K^+^ efflux from the vacuole to the cytosol. |
| KEV | Depolarization | 1 | Promotes | Direct | V.f | [33] | Release of K^+^ from the vacuole promotes plasma membrane depolarization. |
| KOUT | K^+^ Efflux | 1 | Promotes | Direct | A.t. | [52] | Outwardly rectifying K^+^ channels (KOUT) promote K^+^ efflux from the cytosol to the apoplast. |
| Malate | H_2_O efflux | 1 | Inhibits | Not direct | C.c. | [13] | Malate is an osmoticum that inhibits H_2_O efflux by decreasing water potential in the cell. |
| Microtubule depolymeri-zation | Stomatal closure | 1 | Promotes | Not direct | A.t. | [65] | Microtubule depolymerization is essential for stomatal closure. |
| Microtubule depolymeri-zation | Microtubule depolymeriza-tion | 1 | Promotes | Direct |  | [66] | Once started, microtubule depolymerization continues for a sustained period; a process termed catastrophe. |
| NAD^+^ | cADPR | 1 | Promotes | Direct | A.t. | [67] | NAD^+^ is a coenzyme that is required for cADPR production |
| NADPH | ROS (H_2_O_2_) | 1 | Promotes | Direct |  |  | NADPH is a coenzyme that is required for ROS (H_2_O_2_) production by RBOH (NADPH oxidase). |
| NADPH | NO | 1 | Promotes | Direct | A.t. | [68] | NADPH is a coenzyme that is required for NO production by NIA1/2 |
| NIA1/2 | NO | 1 | Promotes | Direct | A.t. | [68] | NIA1 and NIA2 enzymes are required for NO production. |
| Nitrite | NO | 1 | Promotes | Direct | A.t. | [68] | Nitrite is a substrate required for NO production by NIA1 and NIA2 enzymes |
| NO | NOGC1 | 1 | Promotes | Direct | A.t. | [61] | NO binds to NOGC1 and promotes the enzyme activity of NOGC1, which in turn accelerates the production of cGMP. |
| NO | 8-Nitro-cGMP | 1 | Promotes | Not direct | A.t. | [1] | Application of NO (NO donors: NOC5 and SNAP) increases production of 8-nitro-cGMP in guard cells. Application of NO scavenger (cPTIO) reduces ABA-and NO-mediated production of 8-nitro-cGMP. Application of ODQ (GC inhibitor) causes reduction of NO-mediated production of 8-nitro-cGMP. But, simultaneous application of NOC5 (NO donor) and ODQ (GC inhibitor) and cGMP, guard cells show higher level of 8-nitro-cGMP production.These findings suggest that NO promotes the biosynthesis of 8-nitro-cGMP. |
| NO | KOUT | 1 | Inhibits | Direct | V.f | [69] | Outwardly rectifying K^+^ channels are inhibited by NO |
| NOGC1 | cGMP | 1 | Promotes | Direct | A.t. | [61] | AtNOGC1 is a guanylate cyclase that binds to NO. NOGC1 is responsible for the production of cGMP. |
| NtSyp121-Sp2  (SNARE protein) | CaIM | 1 | Inhibits | Not Direct | N. | [70] | SNAREs, soluble NSF (N-ethylmaleimide-sensitive factor) attachment protein receptors, are membrane trafficking proteins that play important role in vesicle fusion and membrane trafficking.  Expression of NtSyp121-Sp2 fragment inhibits gating (opening) of Ca^2+^ permeable channels.  These findings implicate that SNARE proteins are positive regulators in Ca^2+^ channel gating. |
| OST1 | SLAC1 | 1 | Promotes | Direct | A.t. | [16, 71] | SLAC1 slow anion channel 1) is required for ABA-mediated stomatal closure. OST1 physically interacts with SLAC1. OST1 activates SLAC1 by phosphorylation. |
| OST1 | QUAC1 | 1 | Promotes | Direct | A.t., X. | [25, 72, 73] | QUAC1 (AtALMT12) encodes a rapid anion channel. OST1 physically interacts with QUAC1. OST1 interaction causes promotion of QUAC1 activity.  QUAC1 (AtALMT12) is required for ABA and calcium-mediated stomatal responses |
| OST1 | RbohD/F (RBOH) | 1 | Promotes | Direct | A.t., a.c.l. | [45, 74-76] | OST1 physically interacts with both RbohD/F. OST1 kinase phosphorylates NADPH oxidase subunits RbohD/F; OST1 promotes ROS production; RbohD and RbohF are required for ABA-mediated ROS production |
| OST1 | PIP2;1 | 1 | Promotes | Direct | A.t. | [77] | OST1-mediated phosphorylation activates aquaporin Plasma membrane Intrinsic Protein 2;1 (PIP2;1) in guard cells in response to ABA. |
| PA | ABI1 | 1 | Inhibits | Direct | A.t. | [78, 79] | PA interacts with ABI1, which in turn decreases the phosphatase activity of ABI1. PA also regulates ABI1 by sequestering it to the plasma membrane. |
| PA | SPHK1 and SPHK2 (SPHK1/2) | 1 | Promotes | Direct | A.t. | [11] | PA interacts with SPHK1 and SPHK2. PA promotes the binding of substrate to SPHKs that in turn accelerates the activity of SPHKs. |
| PA | RBOH | 1 | Promotes | Direct | A.t. | [80] | PA binds to RbohD/F. Binding of PA activates NADPH oxidase activity of RBOH. |
| PC | PA | 1 | Promotes | Direct | A.t. | [37] | PC is a substrate for PA production by both PLDα and PLDδ. |
| PEPC | Malate | 1 | Promotes | Not direct | V.f | [6] | PEPC is an upstream enzyme of malate biosynthesis. |
| pH_c_ increase | KOUT | 1 | Promotes | Not Direct | V.f | [81] | Outwardly rectifying K^+^ channels are activated by cytosolic pH increase. |
| pH_c_ increase | H^+^ ATPase | 1 | Inhibits | Not direct | N. | [82] | The H^+^ ATPase is inhibited by cytosolic H^+^ concentration decrease. |
| pH_c_ increase | ABI1 | 1 | Promotes | Direct | A.t. | [83] | pH increase activates enzyme activity of ABI1. |
| pH_c_ increase | pH_c_ | 1 | Inhibits | Not direct | A.t., pea | [62, 84, 85] | Time course data indicate that in response to ABA, in guard cells, pH_c_ value increases quickly and then decreases gradually, subsequently stabilizing above the resting level. No signaling components have been identified. |
| PI3P5K | PtdIns(3,5)P2 | 1 | Promotes | Direct | A.t. | [7] | The enzyme PI35PK produces PtdIns(3,5)P2. |
| PIP2;1  (Aquaporin) | H_2_O Efflux | 1 | Promotes | Direct | A.t. | [77] | Aquaporin (PIP2;1) facilitates water efflux during ABA induced stomatal closure. |
| PLC | DAG | 1 | Promotes | Direct |  |  | PLC (enzyme) uses PIP2 (substrate) for production of DAG. |
| PLC | InsP3 | 1 | Promotes | Direct |  |  | PLC (enzyme) uses substrate PIP2 for production of InsP3. |
| PLC | PtdIns(4,5)P2 (PI4P5P2) | 1 | Promotes | Direct | A.t. | [86] | PI45P_2_ (PtdIns(4,5)P2) can be cleaved into inositol 1,4,5-trisphosphate (IP_3_ ) and diacylglycerol by phospholipase C |
| PLDα | PA | 1 | Promotes | Direct | A.t. | [79] | PA is a product of PLDα. |
| PLDδ | PA | 1 | Promotes | Direct | A.t. | [87] | PA is a product of PLDδ. |
| PP2CA | SLAC1 | 1 | Inhibits | Direct | A.t. | [88] | PP2CA means protein phosphatase 2C (also known as AHG3 (ABA Hypersensitive to Germination3). PP2CA physically interacts with SLAC1. Upon coexpression in Xenopus oocytes, PP2CA inhibits the activity of SLAC1. A PP2CA phosphatase-dead mutant does not inhibit SLAC1 activity. |
| PP2CA | OST1 | 1 | Inhibits | Direct | A.t. | [88] | PP2CA physically interacts with OST1. Upon physical interaction, PP2CA forms a complex with OST1 that in turn blocks the activity of OST1. |
| PtdIns(3,5)P2 | V-PPase | 1 | Promotes | Not direct | V.r. | [7] | PtdIns(3,5)P_2_ binds to the V-PPase (vacuolar pyrophosphatase) and activates it. |
| PtdIns(3,5)P2 | V-PPase | 1 | Promotes | Direct | A.t.,  V.f. | [7] | The functional significance of the interaction between PtdIns(3,5)P_2_ and V-PPase not determined yet. The authors speculate that upon interaction PtdIns(3,5)P_2_ may activate the V-PPase. Our network includes this as the PtdIns(3,5)P2→V-PPase edge. |
| PtdIns(4,5)P2 | DAG | 1 | Promotes | Direct |  |  | PLC (enzyme) uses PtdIns(4,5)P_2_ (substrate) for production of DAG. |
| PtdIns(4,5)P2 | InsP3 | 1 | Promotes | Direct |  |  | PLC (enzyme) uses substrate PtdIns(4,5)P2 for production of InsP3. |
| PtdInsP3 and PtdInsP4 | Actin reorganization | 1 | Promotes | Not direct | C.c. | [89] | (PtdInsP3: phosphatidylinositol 3-phosphate)  (PtdInsP4: phosphatidylinositol 4-phosphate)  LY29402 (inhibitor of PtdInsP3 biosynthesis) and wortmanin (inhibitor of PtdInsP4 biosynthesis) inhibit ABA-induced actin reorganization in guard cells. PtdInsP3 and PtdInsP4 have been implicated as negative regulators of actin binding proteins (ABPs). It is unknown whether InsP3 and InsP4 bind to actin. |
| PtdInsP4 | PtdIns(4,5)P2 | 1 | Promotes | Direct |  | [86] | Precursor-product relationship. |
| QUAC1 | AnionEM | 1 | Promotes | Direct | A.t. | [24, 25] | QUAC1 facilitates efflux of malate anions. |
| RBOH | ROS | 1 | Promotes | Direct | A.t. | [90] | RBOH (D and F) enzymes are responsible for production of ROS. |
| RCARs | ABI1/ABI2/HAB1 | 1 | Inhibits | Direct | A.t. | [2, 3, 5] | Upon ABA binding, RCARs interact with ABI1/ABI2/HAB1 that in turn causes inhibition of their phosphatase activity. |
| RCARs | PP2CA | 1 | Inhibits | Direct | A.t. | [91] | In an *in vitro* study, it has been shown that soluble ABA receptors RCARs (PYR1, PYL1, PYL2, PYL4, PYL5, PYL6, PYL8) inhibit the phosphatase activity of PP2CA in the presence of ABA. RCARs are alternatively known as PYR/PYL. |
| ROP11 | ABI2 | 1 | Promotes | Direct | A.t. | [57] | ROP11 physically interacts with ABI2. The physical interaction between ROP11 and ABI2 promotes phosphatase activity of ABI2, which in turn inhibits OST1 kinase activity. |
| ROP11 | ABI1 | 1 | Promotes | Direct | A.t. | [92, 93] | ROP11 physically interacts with ABI1. ROP11 protects the phosphatase activity of ABI1 by interfering with the inhibitory effect of the ABA receptor RCAR1. |
| ROS | 8-Nitro-cGMP | 1 | Promotes | Not direct | A.t. | [1] | Suppression of ROS production by application of different reagents (reducing agent, DTT; superoxide scavenger, Trion; and H_2_O_2_ scavenger, catalase) reduces production of 8-nitro-cGMP.  The *abi1-1* mutant (dominant negative), which does not show ROS production in response to ABA, also does not induce production of 8-nitro-cGMP in response to ABA. These findings suggest that ROS are required for the production of 8-nitro-cGMP. |
| ROS | KOUT | 1 | Inhibits | Not direct | V.f | [94] | Outwardly rectifying K^+^ channels are inhibited by ROS. |
| ROS | H^+^ ATPase | 1 | Inhibits | Not direct | V.f | [95] | The H^+^ ATPase is inhibited by reactive oxygen species. |
| ROS | ABI1 | 1 | Inhibits | Direct | A.t. | [96] | ROS inhibit ABI1 activity. |
| ROS | HAB1 | 1 | Inhibits | Direct | A.t. | [97] | ROS oxidize H_2_O_2_-sensitive thiols and inhibit HAB1’s catalytic activity. |
| ROS | ABI2 | 1 | Inhibits | Direct | A.t. | [98] | ABI2 is negatively regulated by ROS. |
| S1P/PhytoS1P | S1P/PhytoS1P | 1 | Inhibits | Not direct | A.t.and pea | [8, 62, 84] | Time course data indicate that in response to ABA, S1P accumulation in guard cells increases quickly and then decreases gradually, subsequently stabilizing above the resting level. This biological phenomenon has been implicated as negative feedback regulation of S1P/PhytoS1P. |
| SCAB1 | Actin reorganization | 1 | Promotes | Direct | A.t. | [99] | SCAB1 (stomatal closure related actin binding protein1) binds actin filaments. *scab1* mutant shows delayed stomatal closure-associated with slower actin reorganization in response to ABA.  *scab1* mutant shows reduced stomatal movements in response to ABA, ROS and CaCl_2_. |
| SLAC1 | AnionEM | 1 | Promotes | Direct | A.t. | [16, 24] | SLAC1 facilitates efflux of chloride and nitrate anions. |
| SLAH3 | AnionEM | 1 | Promotes | Direct | A.t. | [15, 24] | SLAH3 facilitates efflux of nitrate anions. |
| Sph | S1P/phytoS1P | 1 | Promotes | Direct | A.t. | [8] | Sph is a substrate needed for S1P production. |
| SPHK1/2 | S1P  /phytoS1P | 1 | Promotes | Direct | A.t. | [11, 100] | SPHK1 and SPHK2 (SPHK1/2) enzymes are responsible for S1P production. |
| TCTP | Microtubule depolymeriza-tion | 1 | Promotes | Direct | A.t. | [31] | Interaction between AtTCTP and microtubules promotes microtubule depolymerization. |
| Vacuolar acidification | pH_c_ | 1 | Promotes | Direct | V.f. | [7] | By employing a pharmacological approach, the authors showed that vacuolar acidification is necessary for cytoplasmic alkalization (pH_c_ increase).  Inhibition of ABA-induced cytosolic alkalization by butyrate (a weak acid) application causes suppression of vacuolar acidification in response to ABA.  These above findings suggest that vacuolar acidification and cytosolic alkalization are interdependent during ABA-mediated stomatal closure. |
| Vacuolar acidification | KEV | 1 | Promotes | Not Direct | V.f | [33] | Vacuolar acidification induces K^+^ efflux from the vacuole. |
| V-ATPase  and | Vacuolar acidification | 1 | Promotes | Direct | Yeast,  A.t.,  V.f. | [7, 101, 102] | The functioning of vacuolar pumps is essential for acidification of vacuole. In yeast, the vacuolar proton ATPase (V-ATPase) proton pump plays an important role in vacuolar acidification. Loss of vacuolar pump (V-ATPase) activity delays stomatal closure in response to ABA. |
| V-PPase  (vacuolar pump) | Vacuolar acidification | 1 | Promotes | Direct | A.t.,  V.f. | [7] | Proton pumping vacuolar pyrophosphatase (V-PPase) uses energy of PPi hydrolysis to acidify the vacuole. Loss of vacuolar pump (V-PPase) activity delays stomatal closure in response to ABA. |
| 8-Nitro-cGMP | Stomatal closure | 2 | Promotes | Not Direct | A.t. | [1] | 8-Nitro-cGMP promotes stomatal closure in a dose-dependent manner but 8-bromo-cGMP does not promote stomatal closure. One path in our network that incorporates this effect is  8-nitro-CGMP→ADPRc→cADPR→CIS→Ca^2+^_c_ →Depolarization→KOUT→K^+^ efflux →H_2_O efflux →Closure |
| ABA | Actin reorganization | 2 | Promotes | Not direct | A.t. | [12] | ABA treatment promotes actin reorganization in guard cells. Our network incorporates this as the  ABA--\|AtRAC1--\| Actin reorganization path. |
| ABA | 8-Nitro-cGMP | 2 | Promotes | Not direct | A.t. | [1] | Application of ABA induces the production of 8-nitro-cGMP in guard cells. One path that incorporates this in our network is ABA→ RCARs --\| ABI1 --\|OST1→RBOH  →ROS→NIA1/2→NO→8-nitro-CGMP |
| ABA | ROS (H_2_O_2_) | 2 | Promotes | Not direct | A.t. | [103] | ABA induces the production of ROS (H_2_O_2_) in guard cells. One path that incorporates this in our network is ABA→ RCARs --\| ABI1 --\|OST1→RBOH →ROS |
| ABA | NO | 2 | Promotes | Not direct | A.t., pea | [104, 105] | ABA promotes the production of NO in guard cells. One path that incorporates this in our network is ABA→ RCARs --\| ABI1 --\|OST1→RBOH →ROS→NIA1/2→NO |
| ABA | K^+^ ion release from vacuole (KEV) | 2 | Promotes | Not direct | C.c. | [106, 107] | ABA promotes transient stimulation of tonoplast K^+^ efflux. One path in our network that explains this is ABA→ RCARs --\| ABI1 --\| OST1→RBOH→ROS →GHR1→ CaIM→Ca^2+^_c_ →KEV |
| ABA | pH_c_ increase | 2 | Promotes | Not direct | pea | [[86](#_ENREF_86)] | ABA induces cytosolic alkalization (pH_c_ increase). One path that incorporates this is ABA→PI3P5K→PtdIns(3,5)P2→V-PPase→Vacuolar acidification→ pH_c_ |
| ABA | H^+^ ATPase | 2 | Inhibits | Not direct | V.f | [95] | ABA inhibits the activity of the H^+^ ATPase. One path that incorporates this is ABA→ RCARs --\| ABI1 --\|OST1  →RBOH →ROS--\| H^+^ ATPase |
| ABA | Microtubule Depolymerization | 2 | Promotes | Not Direct | A.t.,  V.f. | [108] | Application of ABA (10 µM) causes reduction of resolved microtubule structures. One path that incorporates this is ABA→ RCARs --\| ABI1 --\| OST1→RBOH→ROS→  NIA1/2→NO→ NOGC1→cGMP→8- nitroCGMP→  ADPRc →cADPR→CIS→Ca^2+^_c_ →TCTP →Microtubule depolymerization. |
| ABA | Vacuolar acidification | 2 | Promotes | Not direct | A.t.,  V.f. | [7] | The vacuolar lumen is acidified during ABA-mediated stomatal closure. Loss of vacuolar pump activities delays stomatal closure in response to ABA, suggesting the relevance of lumen acidification. Our network includes several paths from ABA to vacuolar acidification, e.g. ABA→PI3P5K→ PtdIns(3,5)P2→V-PPase→Vacuolar acidification |
| Ca^2+^_c_ | Stomatal closure | 2 | Promotes | Not direct | C.c. | [109] | Increasing concentration of Ca^2+^_c_ induces stomatal closure. The network incorporates this by several paths, e.g. Ca^2+^_c_→ MPK9/12→ SLAC1→AnionEM→ H_2_O Efflux→closure |
| Ca^2+^_c_ | Slow anion channels (SLAC1) | 2 | Promotes | Not direct | A.t. | [48, 71, 110, 111] | Increase of Ca^2+^_c_ increases the activity of Slow anion channels. One path that incorporates this in our network is Ca^2+^_c_→ MPK9/12→ SLAC1. |
| NO | Closure | 2 | Promotes | Not direct | V.f. | [112] | NO promotes stomatal closure. One path that incorporates this is ABA→ RCARs --\| ABI1 --\| OST1→RBOH→ROS→NIA1/2→NO |
| NO | cGMP | 2 | Promotes | Not direct | A.t. | [113] | NO promotes cGMP production in Arabidopsis seedlings. This is incorporated in the NO→NOGC1→cGMP path. |
| NOGC1 | 8-nitro-cGMP | 2 | Promotes | Not direct | A.t. | [1] | Guard cells of *nogc1* mutant show stomatal closure in response to 8-nitro-cGMP. Guard cells of *nogc1* mutant do not show stomatal closure in response to ABA. These findings suggest that 8-nitro-cGMP is downstream of NOGC1 in guard cell ABA signaling. Our network incorporates this evidence as the  NOGC1→cGMP→8-nitro-cGMP path. |
| NtSyp121-Sp2 | Ca^2+^ transient | 2 | Inhibits | Not Direct | N. | [70] | Expression of NtSyp121-Sp2 fragment inhibits the induction of intracellular Ca^2+^ transient. This suggests that NtSyp121 promotes Ca^2+^ increase. Our network incorporates this effect as the NtSyp121→CaIM→Ca^2+^_c_ path. |
| OST1 | CaIM  (Ca^2+^ channels) | 2 | Promotes | Not direct | A.t. | [45] | *ost1* mutants are insensitive to ABA-activation of Ca^2+^ channels but plants overexpressing OST1 are hypersensitive to ABA-activation of Ca^2+^ channels. It is unknown whether OST1 interacts with Ca^2+^ channels.  Our network incorporates this evidence as the OST1→RBOH→ROS→GHR1→CaIM path. |
| pH_c_  increase | Vacuolar acidification | 2 | promotes | Direct | V.f. | [7] | Inhibition of ABA-induced cytosolic alkalization by butyrate (a weak acid) application causes suppression of vacuolar acidification in response to ABA. Our network incorporates the dependence of vacuolar acidification on cytosolic alkalization as the path  pH_c_ Increase→RBOH→ROS →GHR1→ CaIM→Ca^2+^_c_→ V-ATPase→Vacuolar acidification. |
| PP2CA | Stomatal closure | 2 | Inhibits | Not direct | A.t. | [114] | PP2CA encodes the protein phosphatase 2CA (also known as AHG3 (ABA Hypersensitive to Germination3). Overexpression of PP2CA causes ABA insensitivity in ABA induced stomatal closure whereas loss-of-function mutants are hypersensitive in ABA-mediated stomatal closure. One path that incorporates this relationship is PP2CA--\| SLAC1→AnionEM→ H_2_O Efflux→closure. |
| ROS  (H_2_O_2_) | NO | 2 | Promotes | Not direct | A.t. | [105] | ABA-induced NO generation is dependent on ABA-induced ROS (H_2_O_2_) production. This is incorporated in the path ROS→NIA1/2→NO. |
| ROS (H_2_O_2_) | Microtubule Depolymerization | 2 | Promotes | Not Direct | A.t.,  V.f. | [108] | Application of ROS (H_2_O_2_) causes reduction of resolved microtubule structures. This is incorporated in the network by the ROS→NIA1/2→NO→ NOGC1→cGMP→8-nitroCGMP→ADPRc→cADPR→CIS→Ca^2+^_c_ →TCTP →Microtubule depolymerization path. |
| ROS (H_2_O_2_) | Stomatal closure | 2 | Promotes | Not Direct | A.t.,V.f. | [19, 115, 116] | Application of ROS promotes stomatal closure. The network incorporates this by several paths, e.g.  ROS→GHR1→SLAC1→AnionEM→ H_2_O Efflux→closure |
| S1P | Stomatal closure | 2 | Promotes | Not direct | A.t., C.c | [8, 117] | Application of S1P induces stomatal closure. The network incorporates this by several paths, e.g. S1P/PhytoS1P →GPA1→RBOH→ROS→GHR1→SLAC1→AnionEM→ H_2_O Efflux→closure. |
| ABI1 | ABA induced pH_c_ increase | 3 | Inhibits | Not direct | A.t. | [30] | ABA does not induce cytosolic alkalization in *abi1-*dominant mutant. We assume that ABI1 inhibits pH_c_ increase. |
| ABI1 | ABA activation of RBOH | 3 | Inhibits | Not direct | A.t. | [118] | Guard cells of the *abi1-1* mutant (dominant negative) do not show ABA induced ROS production. We assume that ABI1 inhibits RBOH. |
| ABI1 | SLAC1 | 3 | Inhibits | Direct | A.t. | [20] | CPK21 and CPK23 mediated activation of SLAC1 is suppressed by ABI1.  This is incorporated as separate positive edges from CPK3/21 and CPK23 to SLAC1, and an inhibitory edge from ABI1 to SLAC1. The dependency among the edges is incorporated in the dynamic model through the regulatory function of SLAC1. |
| ABI1 | CPK3 and CPK6 activation of SLAC1 | 3 | Inhibits | Direct | A.t., X. | [36, 50] | ABI1 inhibits CPK3/6-mediated activation of SLAC1. Our network incorporates this as ABI1 inhibiting SLAC1. The dependency among the edges is incorporated in the regulatory function of SLAC1. |
| ABI2 | ABA induced pH_c_ increase | 3 | Inhibits | Not direct | A.t. | [30] | ABA does not induce cytosolic alkalization in *abi2-1* dominant mutant. We assume that ABI2 inhibits pH_c_ increase. |
| ABI2 | CPK6 and CPK23  activation of SLAC1 | 3 | Inhibits | Direct | A.t., X. | [20, 50] | ABI2 inhibits CPK6/23 mediated activation of SLAC1. Our network incorporates this as ABI2 inhibiting SLAC1. The dependency among the edges is incorporated in the regulatory function of SLAC1. |
| AtSPP1 | ABA-induced stomatal closure | 3 | Inhibits | Not direct | A.t. | [29] | Long-chain base 1-phosphates (LCBP) are sphingolipid metabolites. LCBPs are synthesized by LCB kinase and dephosphorylated by LCBP phosphatase and degraded by LCBP lyase.  AtSPP1 (Arabidopsis thaliana sphingoid phosphate phosphatase 1) is an LCBP phosphatase.  Guard cells of *atspp1* mutant show slightly enhanced stomatal closure compared to wild type in response to ABA. Our network incorporates this by negative paths between SPP1 and closure, e.g. SPP1--\|S1P/PhytoS1P →GPA1→RBOH→ROS→GHR1→SLAC1→AnionEM→ H_2_O Efflux→closure. |
| AtTCTP (TCTP node) | Ca^2+^-mediated stomatal closure | 3 | Promotes | Not Direct | A.t. | [31] | TCTP encodes a translationally controlled tumor protein (known as P23 in human) and belongs to a family of calcium- and tubulin-binding proteins. Plants overexpressing AtTCTP show faster Ca^2+^-mediated stomatal closure. Our network incorporates this as the TCTP →Microtubule depolymerization→Closure path. |
| AtTCTP (TCTP node) | ABA-mediated stomatal closure | 3 | Promotes | Not Direct | A.t. | [31] | TCTP encodes a translationally controlled tumor protein (known as P23 in human) and belongs to a family of calcium- and tubulin-binding proteins. Plants overexpressing AtTCTP show faster ABA-mediated stomatal closure. Our network incorporates this as the TCTP →Microtubule depolymerization→Closure path. |
| Ca^2+^ | S1P activation of PLDα | 3 | Promotes | Not direct | A.t. | [100] | Phyto-S1P does not activate PLDα1 directly *in vitro*. As S1P caused an increase in Ca^2+^ in response to ABA, this effect may be mediated by an increase in cytoplasmic Ca^2+^ which promotes PLDα1 translocation to the plasma membrane and tonoplast.Ca^2+^ is a key factor required for PLDα1 activity. As S1P, Ca^2+^_c_  and PLDα are part of the strongly connected component of the network, there are paths between them in both directions. |
| Ca^2+^ | 8-nitro-cGMP-mediated stomatal closure | 3 | Promotes | Not Direct | A.t. | [1] | Application of cell-permeating Ca^2+^ chelator (BAPTA-AM) inhibits 8-nitro-cGMP-mediated stomatal closure. Similarly, application of BAPTA-AM inhibits NOC5- (NO donor) mediated stomatal closure. These findings suggest that Ca^2+^ is a required signaling component in 8-nitro-cGMP-mediated stomatal closure. Indeed, Ca^2+^_c_ is a mediator of all the paths from 8-nitro-CGMP to closure in our network. |
| Ca^2+^ | ABA induction of KEV | 3 | Promotes | Not direct | C.c. | [107, 119] | Above-threshold level of Ca^2+^_c_ is required for ABA activation of KEV and K^+^ ion release from vacuole. Inhibition of Ca^2+^ influx through Ca^2+^-permeable channels and Ca^2+^ release from internal stores blocks K^+^ release from vacuoles. Ca^2+^_c_ mediates many paths between ABA and KEV in our network, e.g. ABA→ RCARs --\| ABI1 --\| OST1→RBOH→ROS →GHR1→ CaIM→Ca^2+^_c_ →KEV |
| Ca^2+^ | ABA-induced NO production | 3 | Promotes | Not direct | Pea | [104] | Application of EGTA (a calcium chelator) restricts ABA-induced NO production and stomatal closure. One path that incorporates the effect of Ca^2+^_c_ is ABA→ RCARs --\| ABI1→AtRAC1--\| Actin reorganization→CaIM→Ca^2+^_c_ →pH_c_ →RBOH→ROS→NIA1/2→NO |
| cADPR | 8-nitro-cGMP-induced stomatal closure | 3 | Promotes | Unknown | A.t. | [1] | Cyclic adenosine-5’-diphosphate-ribose (cADPR) is a second messenger that modulates intracellular Ca^2+^ levels. Application of an antagonist of cADPR production (nicotinamide or 8-bromo-cADPR) inhibits 8-nitro-cGMP-mediated stomatal closure. These two inhibitors also inhibit NO-mediated stomatal closure. One path that incorporates these relationships NO→ NOGC1→cGMP→8-nitroCGMP→ADPRc→cADPR→CIS→Ca^2+^_c_→QUAC1→AnionEM →H_2_O efflux →Closure |
| CIS | ABA-induced stomatal closure | 3 | Promotes | Not direct | A.t. | [120] | Pharmacological inhibition of the cADP-ribose/ryanodine receptor or the IP_3_ receptor negatively affects ABA-induced stomatal closure. As both receptors are likely to control the release of Ca^2+^ from intracellular stores, this finding suggests that intracellular Ca^2+^ stores participate in guard cell ABA signaling.. One path that incorporates the involvement of CIS in ABA induced closure is ABA→ RCARs --\| ABI1 --\| OST1→RBOH→ROS→NIA1/2  →NO→ NOGC1→cGMP→8- nitroCGMP→ADPRc→ cADPR→CIS→Ca^2+^_c_ →QUAC1→AnionEM →H_2_O efflux →Closure |
| GAPC1, GAPC2 | ROS activation of PLDδ | 3 | Promotes | Direct | A.t. | [55] | Upon interaction with ROS, GAPC1/2 (Glyceraldehyde-3-phosphate dehydrogenases 1 and 2) interact with PLDδ and transduce the ROS (H_2_O_2_)-signal. |
| GHR1 | ROS activation of CaIM  (Ca^2+^ channels) | 3 | Promotes | Not direct | A.t. | [19] | *ghr1* mutation impairs ABA- and ROS (H_2_O_2_)-activation of Ca^2+^ channels in Arabidopsis thaliana. It is unknown whether GHR1 physically interacts with Ca^2+^ channels. We assume that ROS activates GHR1 and GHR1 activates CaIM. |
| GHR1 | Activation of SLAC1 by ROS (H_2_O_2_) | 3 | Promotes | Not Direct | A.t. | [19] | GHR1 is a positive regulator of ABA- and ROS (H_2_O_2_)-mediated stomatal closure. *ghr1* mutation impairs ABA-, ROS mediated activation of SLAC1, Ca^2+^ channels and promotion of stomatal closure.  No direct interaction between ROS and GHR1 has been shown. Taken together with the fact that GHR1 interacts with and activates SLAC1, we infer that ROS indirectly promotes GHR1. |
| GPA1 | ABA activation of RBOH | 3 | Promotes | Not direct | A.t. | [116] | RbohD and RbohF (RBOH ) are vital components for ABA-induced ROS production. Guard cells of gpa1 mutant plants do not show significant ROS production in response to ABA. We assume that GPA1 is a positive regulator of RBOH. |
| GPA1 | S1P – induced closure | 3 | Promotes | Not direct | A.t. | [8] | GPA1 is required for S1P-mediated stomatal response. We assume that S1P functions through GPA1, incorporated by an S1P/phytoS1P→GPA1 edge. |
| GPA1 | ABA-induced CaIM | 3 | Promotes | Not direct | A.t. | [116] | Guard cells of gpa1 mutant plants do not show ABA-activation of Ca^2+^-permeable channels suggesting that GPA1 is a positive regulator or mediator of ABA activation of Ca^2+^-permeable channels in guard cells. One path that incorporates this is ABA→SPHK1/2→S1P/phytoS1P→GPA1→  RBOH→ROS→GHR1→CaIM |
| MPK9/12 | ROS-mediated stomatal closure | 3 | Promotes | Not direct | A.t. | [121] | *mpk9 mpk12* double mutants are impaired in ROS-mediated stomatal responses. No direct interaction between MPK9/12 and ROS has been shown. Our network incorporates several paths between ROS and Ca^2+^_c,_ followed by Ca^2+^_c_→MPK9/12 →SLAC1, and several paths between SLAC1 and closure. |
| MPK9/12 | Ca^2+^ induced activation of SLAC1 | 3 | Promotes | Not direct | A.t. | [121] | MAP kinases MPK9 and MPK12 play important roles in ABA and Ca^2+^ activation of S-type anion channels. Direct interaction between MPK9/12 and anion channels has not been shown. We assume that Ca^2+^ promotes MPK9/12 and MPK9/12 promotes SLAC1. |
| MRP5  (ATP binding cassette (ABC) protein) | ABA activation of CaIM | 3 | Promotes | Not direct | A.t. | [122] | Guard cells of *mrp5* mutant do not show ABA activation of Ca^2+^- channels.  *mrp5* loss-of-function mutant plants show partially impaired ABA-induced stomatal closure. We assume that MRP5 promotes the activation of CaIM. |
| MRP5 | Ca^2+^ activation of slow (S-type) anion channel | 3 | promotes | Not direct | A.t. | [122] | MRP5 encodes an ATP binding cassette (ABC) protein. *mrp5* mutant shows less pronounced Ca^2+^ activation of anion currents. *mrp5* mutant also does not show ABA activation of S-type anion currents. We assume that MRP5 promotes CaIM. One path that incorporates the role of MRP5 in the activation of SLAC1 is this is MRP5→Ca^2+^_c_→MPK9/12→SLAC1 |
| NIA1 and NIA2 (NIA1/2) | ROS induced NO production | 3 | Promotes | Not direct | A.t. | [105] | In the *NIA1/2* double mutant (loss-of-function), *nia1* *nia2*, ROS fails to induce NO production. This finding suggests that NIA1 and NIA2 are positively regulated by ROS.  ROS (H_2_O_2_) does not enhance nitrate reductase activity *in vitro*. This suggests that ROS (H_2_O_2_) does not interact directly with NIA1/NIA2. Taken together with the fact that NIA1 and NIA2 are the enzymes responsible for NO production, we infer that ROS promotes the activity of NIA1/2. |
| NO | NtSyp121-Sp2-mediated inhibition of ABA-induced stomatal closure | 3 | Inhibits | Not Direct | N. | [70] | Application of NO donor, SNAP, elevates the intracellular Ca^2+^ level and partially blocks the inhibitory effect of NtSyp121-Sp2 fragment on ABA-induced stomatal closure. This finding indicates that expression of NtSyp121-Sp2 fragment inhibits stomatal closure by modulating Ca^2+^. Our network incorporates this in the NtSyp121→CaIM→Ca^2+^_c_ path. |
| NOGC1 | ABA-induced stomatal closure | 3 | Promotes | Direct | A.t. | [1] | Guard cells of the *nogc1* mutant do not show ABA-induced stomatal closure, indicating that NOGC1 is required for ABA-mediated stomatal closure. Our network incorporates several paths from ABA to closure that pass through NOGC1, e.g. ABA→ RCARs --\| ABI1 --\|OST1→RBOH→  ROS→NIA1/2→NO→ NOGC1→cGMP→8- nitroCGMP →ADPRc→cADPR→CIS→Ca^2+^_c_ →Depolarization→  KOUT→K^+^ efflux →H_2_O efflux →Closure |
| NOGC1 | NO-induced stomatal closure | 3 | Promotes | direct | A.t. | [1] | Guard cells of the *nogc1* mutant do not show NO-induced stomatal closure, indicating that NOGC1 is required for NO-induced stomatal closure. One path in our network that incorporates this is NO→ NOGC1→cGMP→8-nitro- CGMP→ADPRc→cADPR→CIS→Ca^2+^_c_→Depolarization→KOUT→K^+^ efflux →H_2_O efflux →Closure |
| NtSyp121-Sp2 fragment (SNARE protein) | ABA-induced stomatal closure | 3 | Inhibits | Not Direct | N. | [70] | SNAREs, soluble NSF (N-ethylmaleimide-sensitive factor) attachment protein receptors, are membrane trafficking proteins that play important roles in vesicle fusion and membrane trafficking.  Expression of an inhibitory (dominant negative) form of the SNARE NtSyp121 inhibits ABA-induced stomatal closure. This finding suggests that SNARE proteins play positive regulatory role in ABA-mediated stomatal closure. One path in our network that incorporates this effect is NtSyp121→CaIM→Ca^2+^_c_ →Depolarization →KOUT→K^+^ efflux →H_2_O efflux →Closure |
| OST1 | ABA induced pH_c_ increase | 3 | Promotes | Not direct | A.t. | [30] | ABA does not induce cytosolic alkalization in the *ost1-2* mutant. We assume that OST1 promotes pH_c_ increase. |
| OST2/AHA1  H^+^-ATPase | ABA-promotion of stomatal closure | 3 | Inhibits | Not direct | A.t. | [63] | OST2 encodes the plasma membrane bound H^+^-ATPase AHA1. Constitutive activation of this gene impairs ABA promotion of stomatal closure. OST2/AHA1 is expressed in guard cells. The network incorporates this finding by including negative paths between the H^+^-ATPase and closure, e.g. H^+^-ATPase--\|Depolarization→KOUT→K^+^ Efflux →H_2_O Efflux→closure. |
| pH_c_ increase | ABA activation of RBOH | 3 | Promotes | Not Direct | A.t. | [85] | ABA-induced cytosolic alkalization (pH_c_ increase) is necessary for ROS production. We assume that pH_c_ increase promotes RBOH. |
| pH_c_ increase | ABA activation of SLAC1 | 3 | Promotes | Not Direct | A.t. | [123] | Clamping cytosolic pH inhibits ABA activation of slow anion channel activity. We assume that pH_c_ increase promotes the activation of SLAC1. |
| pH_c_ increase | ABA-promotion of NO production | 3 | Promotes | Not direct | Pea | [104] | Guard cell pH rises after 6 min of ABA application, peaking at 18 min. NO production starts after 9 min of ABA application and peaks at 18 min. Application of butyrate (a weak acid) reduces cytosolic pH, decreases NO production and prevents stomatal closure in response to ABA. In contrast, application of methylamine (a weak base) enhances cytosolic alkalization and promotes stomatal closure in response to ABA. Our network incorporates several paths from ABA to NO that are mediated by pH_c_, e.g. ABA→ RCARs --\| ABI1 --\| OST1→pH_c_ →RBOH →ROS→NIA1/2→NO |
| PLC | NO-mediated stomatal closure | 3 | Promotes | Not direct | V.f. | [112] | By employing a pharmacological approach, the authors showed that addition of U73122 (PLC inhibitor) inhibits NO induced stomatal closure. This finding suggests that PLC activity is required for NO-mediated stomatal closure. PLC and NO are part of the strongly connected component of the network, so they are connected by paths in both directions. |
| PLC | NO-mediated PA production | 3 | Promotes | Not direct | V.f. | [112] | PLC hydrolyses PtdIns(4,5)P_2_ into IP_3_ and DAG. Subsequently, DAG can be phosphorylated to PA by DAG kinase (DAGK). PA production increases in response to NO treatment in Vicia. Addition of PLC inhibitor (U73122) causes reduced production of PA in response to NO. This finding suggests that PLC is involved NO-induced PA production. Our network incorporates this as the NO→ NOGC1→cGMP→8- nitroCGMP→ ADPRc→cADPR→CIS→Ca^2+^_c_ →PLC→DAG→PA path. |
| PLDδ | NO induced stomatal closure | 3 | Promotes | Not direct | A.t. | [124] | PLDδ knockout disrupts NO induced closure. We assume that NO promotes PLDδ. |
| PLDδ | ROS  induced stomatal closure | 3 | Promotes | Not direct | A.t. | [55, 124] | PLDδ knockout disrupts ROS induced closure. Taken together with the effect of ROS on GAPC1/2's interaction with PLDδ, we assume that ROS promotes PLDδ |
| PP2CA | CPK6-mediated activation of SLAC1 activity | 3 | Inhibits | Direct | A.t.,  X. | [50] | PP2CA inhibits CPK6-mediated activation of SLAC1 activity in oocytes. PP2CA physically interacts with SLAC1. Our network incorporates this as PP2CA inhibiting SLAC1. The dependency among the edges is incorporated in the regulatory function of SLAC1. |
| PtdIns(3,5)P2 | ABA-induced vacuolar acidification | 3 | Promotes | Not direct | A.t. ,  V.f. | [7] | By employing pharmacological approaches the authors have shown that inhibition of PtdIns(3,5)P_2_ biosynthesis causes reduced ABA induction of vacuolar pH. The network incorporates this relationship as the path ABA→PI3P5K→PtdIns(3,5)P2→V-PPase→Vacuolar acidification. |
| PtdIns(3,5)P2 | ABA-induced stomatal closure | 3 | Promotes | Not direct | A.t.  V.f. | [7] | PtdIns3P 5-kinase (PI3P5K) is the enzyme responsible for production of PtdIns(3,5)P_2_ from phosphatidylinositol 3-phosphate.  Pharmacological inhibition of PtdIns(3,5)P_2_ biosynthesis causes delayed stomatal closure in response to ABA. In addition, loss-of-function mutants of PI3P5Ks show delayed ABA-induced stomatal closure. One path that incorporates this relationship is ABA→PI3P5K→PtdIns(3,5)P_2_→V-PPase→Vacuolar acidification→KEV →K^+^ efflux →H_2_O efflux →Closure |
| PtdInsP3 | ABA induced ROS production | 3 | Promotes | Not direct | V.f.  C.c. | [89, 125, 126] | PI3K inhibitors (which deplete PtdInsP3) inhibit the increase of cytosolic calcium and production of ROS in response to ABA in guard cells.  In plants, PtdInsP3 direct regulation of NADPH oxidases (AtrbohD/F) has not been studied. In neutrophils, the NADPH oxidase complex, which consists of many components, is responsible for ROS generation, and is activated by the binding of PtdInsP3to one of the components. We assume that PtdInsP3 activates RBOH. |
| QUAC1 | Ca^2+^ induced closure | 3 | Promotes | Not direct | A.t. | [73] | The QUAC1 loss-of-function mutant shows hyposensitivity to reduction of Ca^2+^-induced stomatal closure. We assume that Ca^2+^ promotes QUAC1. |
| RBOH | ABA induced NO production | 3 | Promotes | Not direct | A.t. | [105] | ABA-induced NO production and stomatal closure are impaired in *atrbohd /f* double mutants. RBOH is a mediator of all the paths between ABA and NO, e.g. ABA→ RCARs --\| ABI1 --\| OST1→RBOH→ROS→NIA1/2→NO |
| RCN1 | ABA induced ROS production | 3 | Promotes | Not direct | A.t. | [90, 127] | RCN1 is required for full ABA-mediated stomatal closure and ROS production. We assume that RCN1 promotes ROS. |
| S1P | ABA-induced Ca^2+^ increase | 3 | Promotes | Not direct | C.c | [128] | S1P causes an increase in Ca^2+^ in response to ABA. As both S1P and Ca^2+^_c_ are part of the strongly connected component of the network, there are paths between them in both directions. |
| SLAC1 | 8-nitro-cGMP -induced stomatal closure | 3 | Promotes | Not direct | A.t. | [1] | Guard cells of *slac1* mutant do not show 8-nitro-cGMP-induced stomatal closure. In our network there are several paths between 8-nitro-cGMP and closure that are mediated by SLAC1, for example 8-nitro-cGMP→ADPRc→  cADPR→CIS→ Ca^2+^_c_→MPK9/12→SLAC1→  PIP2;1→ H_2_O efflux →Closure |
| SPHK1 and SPHK2 | ABA-induced stomatal closure | 3 | Promotes | Not direct | A.t. | [9, 10] | *sphk1* and *sphk2* mutants are deficient in ABA-mediated stomatal closure. One path that incorporates this relationship is ABA→SPHK1/2→ S1P/PhytoS1P  →GPA1→RBOH→ROS→GHR1→SLAC1→AnionEM→ H_2_O Efflux→closure |
| SPHK1/2 | ABA activation of PLDα | 3 | Promotes | Not direct | A.t. | [11] | ABA activation of PLDα1 is attenuated in *sphk* mutants, as is PA production. This relationship is incorporated in the path ABA→SPHK1/2→S1P/Phyto S1P →GPA1→PLDα |

References

1. Joudoi T, Shichiri Y, Kamizono N, Akaike T, Sawa T, Yoshitake J, et al. Nitrated cyclic GMP modulates guard cell signaling in Arabidopsis. Plant Cell. 2013;25(2):558-71. Epub 2013/02/12. doi: 10.1105/tpc.112.105049. PubMed PMID: 23396828; PubMed Central PMCID: PMC3608778.

2. Park SY, Fung P, Nishimura N, Jensen DR, Fujii H, Zhao Y, et al. Abscisic acid inhibits type 2C protein phosphatases via the PYR/PYL family of START proteins. Science. 2009;324(5930):1068-71. Epub 2009/05/02. doi: 10.1126/science.1173041. PubMed PMID: 19407142; PubMed Central PMCID: PMC2827199.

3. Nishimura N, Sarkeshik A, Nito K, Park SY, Wang A, Carvalho PC, et al. PYR/PYL/RCAR family members are major in-vivo ABI1 protein phosphatase 2C-interacting proteins in Arabidopsis. Plant J. 2010;61(2):290-9. Epub 2009/10/31. doi: 10.1111/j.1365-313X.2009.04054.x. PubMed PMID: 19874541; PubMed Central PMCID: PMC2807913.

4. Gonzalez-Guzman M, Pizzio GA, Antoni R, Vera-Sirera F, Merilo E, Bassel GW, et al. Arabidopsis PYR/PYL/RCAR receptors play a major role in quantitative regulation of stomatal aperture and transcriptional response to abscisic acid. Plant Cell. 2012;24(6):2483-96. Epub 2012/06/29. doi: 10.1105/tpc.112.098574. PubMed PMID: 22739828; PubMed Central PMCID: PMCPMC3406898.

5. Ma Y, Szostkiewicz I, Korte A, Moes D, Yang Y, Christmann A, et al. Regulators of PP2C phosphatase activity function as abscisic acid sensors. Science. 2009;324(5930):1064-8. Epub 2009/05/02. doi: 10.1126/science.1172408. PubMed PMID: 19407143.

6. Du Z, Aghoram K, Outlaw WH, Jr. In vivo phosphorylation of phosphoenolpyruvate carboxylase in guard cells of Vicia faba L. is enhanced by fusicoccin and suppressed by abscisic acid. Arch Biochem Biophys. 1997;337(2):345-50. Epub 1997/01/15. PubMed PMID: 9016832.

7. Bak G, Lee EJ, Lee Y, Kato M, Segami S, Sze H, et al. Rapid structural changes and acidification of guard cell vacuoles during stomatal closure require phosphatidylinositol 3,5-bisphosphate. Plant Cell. 2013;25(6):2202-16. Epub 2013/06/13. doi: 10.1105/tpc.113.110411. PubMed PMID: 23757398; PubMed Central PMCID: PMCPMC3723621.

8. Coursol S, Fan LM, Le Stunff H, Spiegel S, Gilroy S, Assmann SM. Sphingolipid signalling in Arabidopsis guard cells involves heterotrimeric G proteins. Nature. 2003;423(6940):651-4. Epub 2003/06/06. doi: 10.1038/nature01643. PubMed PMID: 12789341.

9. Guo L, Mishra G, Markham JE, Li M, Tawfall A, Welti R, et al. Connections between sphingosine kinase and phospholipase D in the abscisic acid signaling pathway in Arabidopsis. J Biol Chem. 2012;287(11):8286-96. Epub 2012/01/26. doi: 10.1074/jbc.M111.274274. PubMed PMID: 22275366; PubMed Central PMCID: PMCPMC3318714.

10. Worrall D, Liang YK, Alvarez S, Holroyd GH, Spiegel S, Panagopulos M, et al. Involvement of sphingosine kinase in plant cell signalling. Plant J. 2008;56(1):64-72. Epub 2008/06/19. doi: 10.1111/j.1365-313X.2008.03579.x. PubMed PMID: 18557834; PubMed Central PMCID: PMC2752831.

11. Guo L, Mishra G, Taylor K, Wang X. Phosphatidic acid binds and stimulates Arabidopsis sphingosine kinases. J Biol Chem. 2011;286(15):13336-45. Epub 2011/02/19. doi: 10.1074/jbc.M110.190892. PubMed PMID: 21330371; PubMed Central PMCID: PMC3075680.

12. Lemichez E, Wu Y, Sanchez JP, Mettouchi A, Mathur J, Chua NH. Inactivation of AtRac1 by abscisic acid is essential for stomatal closure. Genes Dev. 2001;15(14):1808-16. Epub 2001/07/19. doi: 10.1101/gad.900401. PubMed PMID: 11459830; PubMed Central PMCID: PMC312738.

13. Dittrich P, Raschke K. Malate metabolism in isolated epidermis of Commelina communis L. in relation to stomatal functioning. Planta. 1977;134(1):77-81. Epub 1977/01/01. doi: 10.1007/BF00390098. PubMed PMID: 24419583.

14. Hugouvieux V, Kwak JM, Schroeder JI. An mRNA cap binding protein, ABH1, modulates early abscisic acid signal transduction in Arabidopsis. Cell. 2001;106(4):477-87. Epub 2001/08/30. PubMed PMID: 11525733.

15. Geiger D, Maierhofer T, Al-Rasheid KA, Scherzer S, Mumm P, Liese A, et al. Stomatal closure by fast abscisic acid signaling is mediated by the guard cell anion channel SLAH3 and the receptor RCAR1. Sci Signal. 2011;4(173):ra32. Epub 2011/05/19. doi: 10.1126/scisignal.2001346. PubMed PMID: 21586729.

16. Geiger D, Scherzer S, Mumm P, Stange A, Marten I, Bauer H, et al. Activity of guard cell anion channel SLAC1 is controlled by drought-stress signaling kinase-phosphatase pair. Proc Natl Acad Sci U S A. 2009;106(50):21425-30. Epub 2009/12/04. doi: 10.1073/pnas.0912021106. PubMed PMID: 19955405; PubMed Central PMCID: PMC2795561.

17. Umezawa T, Sugiyama N, Mizoguchi M, Hayashi S, Myouga F, Yamaguchi-Shinozaki K, et al. Type 2C protein phosphatases directly regulate abscisic acid-activated protein kinases in Arabidopsis. Proc Natl Acad Sci U S A. 2009;106(41):17588-93. doi: 10.1073/pnas.0907095106. PubMed PMID: 19805022; PubMed Central PMCID: PMC2754379.

18. Vlad F, Rubio S, Rodrigues A, Sirichandra C, Belin C, Robert N, et al. Protein phosphatases 2C regulate the activation of the Snf1-related kinase OST1 by abscisic acid in Arabidopsis. Plant Cell. 2009;21(10):3170-84. doi: 10.1105/tpc.109.069179. PubMed PMID: 19855047; PubMed Central PMCID: PMC2782292.

19. Hua D, Wang C, He J, Liao H, Duan Y, Zhu Z, et al. A plasma membrane receptor kinase, GHR1, mediates abscisic acid- and hydrogen peroxide-regulated stomatal movement in Arabidopsis. Plant Cell. 2012;24(6):2546-61. Epub 2012/06/26. doi: 10.1105/tpc.112.100107. PubMed PMID: 22730405; PubMed Central PMCID: PMC3406912.

20. Geiger D, Scherzer S, Mumm P, Marten I, Ache P, Matschi S, et al. Guard cell anion channel SLAC1 is regulated by CDPK protein kinases with distinct Ca2+ affinities. Proc Natl Acad Sci U S A. 2010;107(17):8023-8. Epub 2010/04/14. doi: 10.1073/pnas.0912030107. PubMed PMID: 20385816; PubMed Central PMCID: PMC2867891.

21. Zhang W, Fan LM, Wu WH. Osmo-sensitive and stretch-activated calcium-permeable channels in Vicia faba guard cells are regulated by actin dynamics. Plant Physiol. 2007;143(3):1140-51. Epub 2007/01/30. doi: 10.1104/pp.106.091405. PubMed PMID: 17259289; PubMed Central PMCID: PMC1820927.

22. Chakravorty D, Trusov Y, Zhang W, Acharya BR, Sheahan MB, McCurdy DW, et al. An atypical heterotrimeric G-protein gamma-subunit is involved in guard cell K^+^-channel regulation and morphological development in Arabidopsis thaliana. Plant J. 2011;67(5):840-51. Epub 2011/05/18. doi: 10.1111/j.1365-313X.2011.04638.x. PubMed PMID: 21575088.

23. Levchenko V, Konrad KR, Dietrich P, Roelfsema MR, Hedrich R. Cytosolic abscisic acid activates guard cell anion channels without preceding Ca2+ signals. Proc Natl Acad Sci U S A. 2005;102(11):4203-8. Epub 2005/03/09. doi: 10.1073/pnas.0500146102. PubMed PMID: 15753314; PubMed Central PMCID: PMC554796.

24. Hedrich R. Ion channels in plants. Physiol Rev. 2012;92(4):1777-811. Epub 2012/10/18. doi: 10.1152/physrev.00038.2011. PubMed PMID: 23073631.

25. Meyer S, Mumm P, Imes D, Endler A, Weder B, Al-Rasheid KA, et al. AtALMT12 represents an R-type anion channel required for stomatal movement in Arabidopsis guard cells. Plant J. 2010;63(6):1054-62. Epub 2010/07/16. doi: 10.1111/j.1365-313X.2010.04302.x. PubMed PMID: 20626656.

26. Jiang K, Sorefan K, Deeks MJ, Bevan MW, Hussey PJ, Hetherington AM. The ARP2/3 complex mediates guard cell actin reorganization and stomatal movement in Arabidopsis. Plant Cell. 2012;24(5):2031-40. Epub 2012/05/10. doi: 10.1105/tpc.112.096263. PubMed PMID: 22570440; PubMed Central PMCID: PMC3442585.

27. Szymanski DB. Breaking the WAVE complex: the point of Arabidopsis trichomes. Curr Opin Plant Biol. 2005;8(1):103-12. doi: 10.1016/j.pbi.2004.11.004. PubMed PMID: 15653407.

28. Nagy SK, Darula Z, Kallai BM, Bogre L, Banhegyi G, Medzihradszky KF, et al. Activation of AtMPK9 through autophosphorylation that makes it independent of the canonical MAPK cascades. Biochem J. 2015;467(1):167-75. Epub 2015/02/04. doi: 10.1042/BJ20141176. PubMed PMID: 25646663.

29. Nakagawa N, Kato M, Takahashi Y, Shimazaki K, Tamura K, Tokuji Y, et al. Degradation of long-chain base 1-phosphate (LCBP) in Arabidopsis: functional characterization of LCBP phosphatase involved in the dehydration stress response. J Plant Res. 2012;125(3):439-49. Epub 2011/09/13. doi: 10.1007/s10265-011-0451-9. PubMed PMID: 21910031.

30. Islam MM, Hossain MA, Jannat R, Munemasa S, Nakamura Y, Mori IC, et al. Cytosolic alkalization and cytosolic calcium oscillation in Arabidopsis guard cells response to ABA and MeJA. Plant Cell Physiol. 2010;51(10):1721-30. Epub 2010/08/27. doi: 10.1093/pcp/pcq131. PubMed PMID: 20739306.

31. Kim YM, Han YJ, Hwang OJ, Lee SS, Shin AY, Kim SY, et al. Overexpression of Arabidopsis translationally controlled tumor protein gene AtTCTP enhances drought tolerance with rapid ABA-induced stomatal closure. Mol Cells. 2012;33(6):617-26. Epub 2012/05/23. doi: 10.1007/s10059-012-0080-8. PubMed PMID: 22610367; PubMed Central PMCID: PMC3887759.

32. Sanders D, Pelloux J, Brownlee C, Harper JF. Calcium at the crossroads of signaling. Plant Cell. 2002;14 Suppl:S401-17. PubMed PMID: 12045291; PubMed Central PMCID: PMC151269.

33. Ward JM, Schroeder JI. Calcium-Activated K+ Channels and Calcium-Induced Calcium Release by Slow Vacuolar Ion Channels in Guard Cell Vacuoles Implicated in the Control of Stomatal Closure. Plant Cell. 1994;6(5):669-83. doi: 10.1105/tpc.6.5.669. PubMed PMID: 12244253; PubMed Central PMCID: PMC160467.

34. Kinoshita T, Nishimura M, Shimazaki K. Cytosolic Concentration of Ca2+ Regulates the Plasma Membrane H+-ATPase in Guard Cells of Fava Bean. Plant Cell. 1995;7(8):1333-42. doi: 10.1105/tpc.7.8.1333. PubMed PMID: 12242406; PubMed Central PMCID: PMC160955.

35. Pei ZM, Baizabal-Aguirre VM, Allen GJ, Schroeder JI. A transient outward-rectifying K+ channel current down-regulated by cytosolic Ca2+ in Arabidopsis thaliana guard cells. Proc Natl Acad Sci U S A. 1998;95(11):6548-53. PubMed PMID: 9601004; PubMed Central PMCID: PMC27872.

36. Scherzer S, Maierhofer T, Al-Rasheid KA, Geiger D, Hedrich R. Multiple calcium-dependent kinases modulate ABA-activated guard cell anion channels. Mol Plant. 2012;5(6):1409-12. Epub 2012/08/31. doi: 10.1093/mp/sss084. PubMed PMID: 22933711.

37. Pappan KL, Wang X. Assaying different types of plant phospholipase D activities in vitro. Methods Mol Biol. 2013;1009:205-17. doi: 10.1007/978-1-62703-401-2_19. PubMed PMID: 23681536.

38. Otterhag L, Sommarin M, Pical C. N-terminal EF-hand-like domain is required for phosphoinositide-specific phospholipase C activity in Arabidopsis thaliana. FEBS Lett. 2001;497(2-3):165-70. Epub 2001/05/30. doi: S0014-5793(01)02453-X [pii]. PubMed PMID: 11377433.

39. Tang RJ, Liu H, Yang Y, Yang L, Gao XS, Garcia VJ, et al. Tonoplast calcium sensors CBL2 and CBL3 control plant growth and ion homeostasis through regulating V-ATPase activity in Arabidopsis. Cell Res. 2012;22(12):1650-65. doi: 10.1038/cr.2012.161. PubMed PMID: 23184060; PubMed Central PMCID: PMC3515760.

40. Guse AH. Cyclic ADP-ribose: a novel Ca2+-mobilising second messenger. Cell Signal. 1999;11(5):309-16. Epub 1999/06/22. PubMed PMID: 10376802.

41. Leckie CP, McAinsh MR, Allen GJ, Sanders D, Hetherington AM. Abscisic acid-induced stomatal closure mediated by cyclic ADP-ribose. Proc Natl Acad Sci U S A. 1998;95(26):15837-42. Epub 1998/12/23. PubMed PMID: 9861057; PubMed Central PMCID: PMC28131.

42. Schroeder JI, Hagiwara S. Repetitive increases in cytosolic Ca2+ of guard cells by abscisic acid activation of nonselective Ca2+ permeable channels. Proc Natl Acad Sci U S A. 1990;87(23):9305-9. PubMed PMID: 2174559; PubMed Central PMCID: PMC55153.

43. Gilroy S, Fricker MD, Read ND, Trewavas AJ. Role of Calcium in Signal Transduction of Commelina Guard Cells. Plant Cell. 1991;3(4):333-44. Epub 1991/04/01. doi: 10.1105/tpc.3.4.333. PubMed PMID: 12324599; PubMed Central PMCID: PMC160004.

44. Grabov A, Blatt MR. Membrane voltage initiates Ca2+ waves and potentiates Ca2+ increases with abscisic acid in stomatal guard cells. Proc Natl Acad Sci U S A. 1998;95(8):4778-83. Epub 1998/04/29. PubMed PMID: 9539815; PubMed Central PMCID: PMC22567.

45. Acharya BR, Jeon BW, Zhang W, Assmann SM. Open Stomata 1 (OST1) is limiting in abscisic acid responses of Arabidopsis guard cells. New Phytol. 2013;200(4):1049-63. Epub 2013/09/17. doi: 10.1111/nph.12469. PubMed PMID: 24033256.

46. Staxen I, Pical C, Montgomery LT, Gray JE, Hetherington AM, McAinsh MR. Abscisic acid induces oscillations in guard-cell cytosolic free calcium that involve phosphoinositide-specific phospholipase C. Proc Natl Acad Sci U S A. 1999;96(4):1779-84. PubMed PMID: 9990101; PubMed Central PMCID: PMC15593.

47. Lemtiri-Chlieh F, MacRobbie EA, Webb AA, Manison NF, Brownlee C, Skepper JN, et al. Inositol hexakisphosphate mobilizes an endomembrane store of calcium in guard cells. Proc Natl Acad Sci U S A. 2003;100(17):10091-5. Epub 2003/08/13. doi: 10.1073/pnas.1133289100. PubMed PMID: 12913129; PubMed Central PMCID: PMC187775.

48. Mori IC, Murata Y, Yang Y, Munemasa S, Wang YF, Andreoli S, et al. CDPKs CPK6 and CPK3 function in ABA regulation of guard cell S-type anion- and Ca(2+)-permeable channels and stomatal closure. PLoS Biol. 2006;4(10):e327. Epub 2006/10/13. doi: 10.1371/journal.pbio.0040327. PubMed PMID: 17032064; PubMed Central PMCID: PMC1592316.

49. Swatek KN, Wilson RS, Ahsan N, Tritz RL, Thelen JJ. Multisite phosphorylation of 14-3-3 proteins by calcium-dependent protein kinases. Biochem J. 2014;459(1):15-25. Epub 2014/01/21. doi: 10.1042/BJ20130035. PubMed PMID: 24438037; PubMed Central PMCID: PMC4127189.

50. Brandt B, Brodsky DE, Xue S, Negi J, Iba K, Kangasjarvi J, et al. Reconstitution of abscisic acid activation of SLAC1 anion channel by CPK6 and OST1 kinases and branched ABI1 PP2C phosphatase action. Proc Natl Acad Sci U S A. 2012;109(26):10593-8. Epub 2012/06/13. doi: 10.1073/pnas.1116590109. PubMed PMID: 22689970; PubMed Central PMCID: PMC3387046.

51. Munnik T, Irvine RF, Musgrave A. Phospholipid signalling in plants. Biochim Biophys Acta. 1998;1389(3):222-72. PubMed PMID: 9512651.

52. Hosy E, Vavasseur A, Mouline K, Dreyer I, Gaymard F, Poree F, et al. The Arabidopsis outward K+ channel GORK is involved in regulation of stomatal movements and plant transpiration. Proc Natl Acad Sci U S A. 2003;100(9):5549-54. doi: 10.1073/pnas.0733970100. PubMed PMID: 12671068; PubMed Central PMCID: PMC154382.

53. Zheng ZL, Nafisi M, Tam A, Li H, Crowell DN, Chary SN, et al. Plasma membrane-associated ROP10 small GTPase is a specific negative regulator of abscisic acid responses in Arabidopsis. Plant Cell. 2002;14(11):2787-97. PubMed PMID: 12417701; PubMed Central PMCID: PMC152727.

54. Allen GJ, Murata Y, Chu SP, Nafisi M, Schroeder JI. Hypersensitivity of abscisic acid-induced cytosolic calcium increases in the Arabidopsis farnesyltransferase mutant era1-2. Plant Cell. 2002;14(7):1649-62. Epub 2002/07/18. PubMed PMID: 12119381; PubMed Central PMCID: PMC150713.

55. Guo L, Devaiah SP, Narasimhan R, Pan X, Zhang Y, Zhang W, et al. Cytosolic glyceraldehyde-3-phosphate dehydrogenases interact with phospholipase Ddelta to transduce hydrogen peroxide signals in the Arabidopsis response to stress. Plant Cell. 2012;24(5):2200-12. Epub 2012/05/17. doi: 10.1105/tpc.111.094946. PubMed PMID: 22589465; PubMed Central PMCID: PMC3442596.

56. Pandey S, Assmann SM. The Arabidopsis putative G protein-coupled receptor GCR1 interacts with the G protein alpha subunit GPA1 and regulates abscisic acid signaling. Plant Cell. 2004;16(6):1616-32. doi: 10.1105/tpc.020321. PubMed PMID: 15155892; PubMed Central PMCID: PMC490050.

57. Yu F, Qian L, Nibau C, Duan Q, Kita D, Levasseur K, et al. FERONIA receptor kinase pathway suppresses abscisic acid signaling in Arabidopsis by activating ABI2 phosphatase. Proc Natl Acad Sci U S A. 2012;109(36):14693-8. Epub 2012/08/22. doi: 10.1073/pnas.1212547109. PubMed PMID: 22908257; PubMed Central PMCID: PMC3437822.

58. Li Z, Liu D. ROPGEF1 and ROPGEF4 are functional regulators of ROP11 GTPase in ABA-mediated stomatal closure in Arabidopsis. FEBS Lett. 2012;586(9):1253-8. Epub 2012/04/17. doi: 10.1016/j.febslet.2012.03.040. PubMed PMID: 22500990.

59. Gookin TE, Assmann SM. Significant reduction of BiFC non-specific assembly facilitates in planta assessment of heterotrimeric G-protein interactors. Plant J. 2014;80(3):553-67. Epub 2014/09/05. doi: 10.1111/tpj.12639. PubMed PMID: 25187041; PubMed Central PMCID: PMC4260091.

60. Zhao J, Wang X. Arabidopsis phospholipase Dalpha1 interacts with the heterotrimeric G-protein alpha-subunit through a motif analogous to the DRY motif in G-protein-coupled receptors. J Biol Chem. 2004;279(3):1794-800. doi: 10.1074/jbc.M309529200. PubMed PMID: 14594812.

61. Mulaudzi T, Ludidi N, Ruzvidzo O, Morse M, Hendricks N, Iwuoha E, et al. Identification of a novel Arabidopsis thaliana nitric oxide-binding molecule with guanylate cyclase activity in vitro. FEBS Lett. 2011;585(17):2693-7. Epub 2011/08/09. doi: 10.1016/j.febslet.2011.07.023. PubMed PMID: 21820435.

62. Li S, Assmann SM, Albert R. Predicting essential components of signal transduction networks: a dynamic model of guard cell abscisic acid signaling. PLoS Biol. 2006;4(10):e312. Epub 2006/09/14. doi: 10.1371/journal.pbio.0040312. PubMed PMID: 16968132; PubMed Central PMCID: PMC1564158.

63. Merlot S, Leonhardt N, Fenzi F, Valon C, Costa M, Piette L, et al. Constitutive activation of a plasma membrane H(+)-ATPase prevents abscisic acid-mediated stomatal closure. EMBO J. 2007;26(13):3216-26. Epub 2007/06/09. doi: 10.1038/sj.emboj.7601750. PubMed PMID: 17557075; PubMed Central PMCID: PMC1914098.

64. Boss WF, Im YJ. Phosphoinositide signaling. Annu Rev Plant Biol. 2012;63:409-29. Epub 2012/03/13. doi: 10.1146/annurev-arplant-042110-103840. PubMed PMID: 22404474.

65. Jiang Y, Wu K, Lin F, Qu Y, Liu X, Zhang Q. Phosphatidic acid integrates calcium signaling and microtubule dynamics into regulating ABA-induced stomatal closure in Arabidopsis. Planta. 2014;239(3):565-75. doi: 10.1007/s00425-013-1999-5. PubMed PMID: 24271006.

66. Gardner MK, Zanic M, Howard J. Microtubule catastrophe and rescue. Curr Opin Cell Biol. 2013;25(1):14-22. Epub 2012/10/25. doi: 10.1016/j.ceb.2012.09.006. PubMed PMID: 23092753; PubMed Central PMCID: PMC3556214.

67. Hunt L, Lerner F, Ziegler M. NAD - new roles in signalling and gene regulation in plants. New Phytol. 2004;163(1):31-44. doi: 10.1111/j.1469-8137.2004.01087.x. PubMed PMID: WOS:000221977600005.

68. Desikan R, Griffiths R, Hancock J, Neill S. A new role for an old enzyme: nitrate reductase-mediated nitric oxide generation is required for abscisic acid-induced stomatal closure in Arabidopsis thaliana. Proc Natl Acad Sci U S A. 2002;99(25):16314-8. Epub 2002/11/26. doi: 10.1073/pnas.252461999. PubMed PMID: 12446847; PubMed Central PMCID: PMC138608.

69. Sokolovski S, Blatt MR. Nitric oxide block of outward-rectifying K+ channels indicates direct control by protein nitrosylation in guard cells. Plant Physiol. 2004;136(4):4275-84. doi: 10.1104/pp.104.050344. PubMed PMID: 15563619; PubMed Central PMCID: PMC535857.

70. Sokolovski S, Hills A, Gay RA, Blatt MR. Functional interaction of the SNARE protein NtSyp121 in Ca2+ channel gating, Ca2+ transients and ABA signalling of stomatal guard cells. Mol Plant. 2008;1(2):347-58. Epub 2008/03/01. doi: 10.1093/mp/ssm029. PubMed PMID: 19825544.

71. Vahisalu T, Kollist H, Wang YF, Nishimura N, Chan WY, Valerio G, et al. SLAC1 is required for plant guard cell S-type anion channel function in stomatal signalling. Nature. 2008;452(7186):487-91. Epub 2008/02/29. doi: 10.1038/nature06608. PubMed PMID: 18305484; PubMed Central PMCID: PMC2858982.

72. Imes D, Mumm P, Bohm J, Al-Rasheid KA, Marten I, Geiger D, et al. Open stomata 1 (OST1) kinase controls R-type anion channel QUAC1 in Arabidopsis guard cells. Plant J. 2013;74(3):372-82. Epub 2013/03/05. doi: 10.1111/tpj.12133. PubMed PMID: 23452338.

73. Sasaki T, Mori IC, Furuichi T, Munemasa S, Toyooka K, Matsuoka K, et al. Closing plant stomata requires a homolog of an aluminum-activated malate transporter. Plant Cell Physiol. 2010;51(3):354-65. Epub 2010/02/16. doi: 10.1093/pcp/pcq016. PubMed PMID: 20154005; PubMed Central PMCID: PMC2835873.

74. Sirichandra C, Gu D, Hu HC, Davanture M, Lee S, Djaoui M, et al. Phosphorylation of the Arabidopsis AtrbohF NADPH oxidase by OST1 protein kinase. FEBS Lett. 2009;583(18):2982-6. Epub 2009/09/01. doi: 10.1016/j.febslet.2009.08.033. PubMed PMID: 19716822.

75. Ogasawara Y, Kaya H, Hiraoka G, Yumoto F, Kimura S, Kadota Y, et al. Synergistic activation of the Arabidopsis NADPH oxidase AtrbohD by Ca2+ and phosphorylation. J Biol Chem. 2008;283(14):8885-92. Epub 2008/01/26. doi: 10.1074/jbc.M708106200. PubMed PMID: 18218618.

76. Kimura S, Kaya H, Kawarazaki T, Hiraoka G, Senzaki E, Michikawa M, et al. Protein phosphorylation is a prerequisite for the Ca2+-dependent activation of Arabidopsis NADPH oxidases and may function as a trigger for the positive feedback regulation of Ca2+ and reactive oxygen species. Biochim Biophys Acta. 2012;1823(2):398-405. Epub 2011/10/18. doi: 10.1016/j.bbamcr.2011.09.011. PubMed PMID: 22001402.

77. Grondin A, Rodrigues O, Verdoucq L, Merlot S, Leonhardt N, Maurel C. Aquaporins Contribute to ABA-Triggered Stomatal Closure through OST1-Mediated Phosphorylation. Plant Cell. 2015;27(7):1945-54. Epub 2015/07/15. doi: 10.1105/tpc.15.00421. PubMed PMID: 26163575; PubMed Central PMCID: PMC4531361.

78. Mishra G, Zhang W, Deng F, Zhao J, Wang X. A bifurcating pathway directs abscisic acid effects on stomatal closure and opening in Arabidopsis. Science. 2006;312(5771):264-6. Epub 2006/04/15. doi: 10.1126/science.1123769. PubMed PMID: 16614222.

79. Zhang W, Qin C, Zhao J, Wang X. Phospholipase D alpha 1-derived phosphatidic acid interacts with ABI1 phosphatase 2C and regulates abscisic acid signaling. Proc Natl Acad Sci U S A. 2004;101(25):9508-13. Epub 2004/06/16. doi: 10.1073/pnas.0402112101. PubMed PMID: 15197253; PubMed Central PMCID: PMC439007.

80. Zhang Y, Zhu H, Zhang Q, Li M, Yan M, Wang R, et al. Phospholipase dalpha1 and phosphatidic acid regulate NADPH oxidase activity and production of reactive oxygen species in ABA-mediated stomatal closure in Arabidopsis. Plant Cell. 2009;21(8):2357-77. Epub 2009/08/20. doi: 10.1105/tpc.108.062992. PubMed PMID: 19690149; PubMed Central PMCID: PMC2751945.

81. Miedema H, Assmann SM. A membrane-delimited effect of internal pH on the K+ outward rectifier of Vicia faba guard cells. J Membr Biol. 1996;154(3):227-37. PubMed PMID: 8952952.

82. Luo H, Morsomme P, Boutry M. The two major types of plant plasma membrane H+-ATPases show different enzymatic properties and confer differential pH sensitivity of yeast growth. Plant Physiol. 1999;119(2):627-34. PubMed PMID: 9952459; PubMed Central PMCID: PMC32140.

83. Leube MP, Grill E, Amrhein N. ABI1 of Arabidopsis is a protein serine/threonine phosphatase highly regulated by the proton and magnesium ion concentration. FEBS Lett. 1998;424(1-2):100-4. Epub 1998/04/16. PubMed PMID: 9537523.

84. Puli MR, Rajsheel P, Aswani V, Agurla S, Kuchitsu K, Raghavendra AS. Stomatal closure induced by phytosphingosine-1-phosphate and sphingosine-1-phosphate depends on nitric oxide and pH of guard cells in Pisum sativum. Planta. 2016;244(4):831-41. Epub 2016/05/29. doi: 10.1007/s00425-016-2545-z. PubMed PMID: 27233507.

85. Suhita D, Raghavendra AS, Kwak JM, Vavasseur A. Cytoplasmic alkalization precedes reactive oxygen species production during methyl jasmonate- and abscisic acid-induced stomatal closure. Plant Physiol. 2004;134(4):1536-45. doi: 10.1104/pp.103.032250. PubMed PMID: 15064385; PubMed Central PMCID: PMC419829.

86. Jung JY, Kim YW, Kwak JM, Hwang JU, Young J, Schroeder JI, et al. Phosphatidylinositol 3- and 4-phosphate are required for normal stomatal movements. Plant Cell. 2002;14(10):2399-412. Epub 2002/10/09. PubMed PMID: 12368494; PubMed Central PMCID: PMC151225.

87. Uraji M, Katagiri T, Okuma E, Ye W, Hossain MA, Masuda C, et al. Cooperative function of PLDdelta and PLDalpha1 in abscisic acid-induced stomatal closure in Arabidopsis. Plant Physiol. 2012;159(1):450-60. Epub 2012/03/07. doi: 10.1104/pp.112.195578. PubMed PMID: 22392280; PubMed Central PMCID: PMC3375977.

88. Lee SC, Lan W, Buchanan BB, Luan S. A protein kinase-phosphatase pair interacts with an ion channel to regulate ABA signaling in plant guard cells. Proc Natl Acad Sci U S A. 2009;106(50):21419-24. Epub 2009/12/04. doi: 10.1073/pnas.0910601106. PubMed PMID: 19955427; PubMed Central PMCID: PMC2795491.

89. Choi Y, Lee Y, Jeon BW, Staiger CJ, Lee Y. Phosphatidylinositol 3- and 4-phosphate modulate actin filament reorganization in guard cells of day flower. Plant Cell Environ. 2008;31(3):366-77. Epub 2007/12/20. doi: 10.1111/j.1365-3040.2007.01769.x. PubMed PMID: 18088331.

90. Kwak JM, Moon JH, Murata Y, Kuchitsu K, Leonhardt N, DeLong A, et al. Disruption of a guard cell-expressed protein phosphatase 2A regulatory subunit, RCN1, confers abscisic acid insensitivity in Arabidopsis. Plant Cell. 2002;14(11):2849-61. Epub 2002/11/06. PubMed PMID: 12417706; PubMed Central PMCID: PMC152732.

91. Antoni R, Gonzalez-Guzman M, Rodriguez L, Rodrigues A, Pizzio GA, Rodriguez PL. Selective inhibition of clade A phosphatases type 2C by PYR/PYL/RCAR abscisic acid receptors. Plant Physiol. 2012;158(2):970-80. Epub 2011/12/27. doi: 10.1104/pp.111.188623. PubMed PMID: 22198272; PubMed Central PMCID: PMC3271782.

92. Li Z, Gao X, Chinnusamy V, Bressan R, Wang ZX, Zhu JK, et al. ROP11 GTPase negatively regulates ABA signaling by protecting ABI1 phosphatase activity from inhibition by the ABA receptor RCAR1/PYL9 in Arabidopsis. J Integr Plant Biol. 2012;54(3):180-8. Epub 2012/01/19. doi: 10.1111/j.1744-7909.2012.01101.x. PubMed PMID: 22251383; PubMed Central PMCID: PMC3586988.

93. Li Z, Kang J, Sui N, Liu D. ROP11 GTPase is a negative regulator of multiple ABA responses in Arabidopsis. J Integr Plant Biol. 2012;54(3):169-79. Epub 2012/01/12. doi: 10.1111/j.1744-7909.2012.01100.x. PubMed PMID: 22233300.

94. Kohler B, Hills A, Blatt MR. Control of guard cell ion channels by hydrogen peroxide and abscisic acid indicates their action through alternate signaling pathways. Plant Physiol. 2003;131(2):385-8. doi: 10.1104/pp.016014. PubMed PMID: 12586862; PubMed Central PMCID: PMC1540280.

95. Zhang X, Wang H, Takemiya A, Song CP, Kinoshita T, Shimazaki K. Inhibition of blue light-dependent H+ pumping by abscisic acid through hydrogen peroxide-induced dephosphorylation of the plasma membrane H+-ATPase in guard cell protoplasts. Plant Physiol. 2004;136(4):4150-8. doi: 10.1104/pp.104.046573. PubMed PMID: 15563626; PubMed Central PMCID: PMC535845.

96. Meinhard M, Grill E. Hydrogen peroxide is a regulator of ABI1, a protein phosphatase 2C from Arabidopsis. FEBS Lett. 2001;508(3):443-6. PubMed PMID: 11728469.

97. Sridharamurthy M, Kovach A, Zhao Y, Zhu JK, Xu HE, Swaminathan K, et al. H2O2 inhibits ABA-signaling protein phosphatase HAB1. PLoS One. 2014;9(12):e113643. doi: 10.1371/journal.pone.0113643. PubMed PMID: 25460914; PubMed Central PMCID: PMC4252038.

98. Meinhard M, Rodriguez PL, Grill E. The sensitivity of ABI2 to hydrogen peroxide links the abscisic acid-response regulator to redox signalling. Planta. 2002;214(5):775-82. doi: 10.1007/s00425-001-0675-3. PubMed PMID: 11882947.

99. Zhao Y, Zhao S, Mao T, Qu X, Cao W, Zhang L, et al. The plant-specific actin binding protein SCAB1 stabilizes actin filaments and regulates stomatal movement in Arabidopsis. Plant Cell. 2011;23(6):2314-30. Epub 2011/07/02. doi: 10.1105/tpc.111.086546. PubMed PMID: 21719691; PubMed Central PMCID: PMC3160031.

100. Guo L, Wang X. Crosstalk between Phospholipase D and Sphingosine Kinase in Plant Stress Signaling. Front Plant Sci. 2012;3:51. Epub 2012/05/29. doi: 10.3389/fpls.2012.00051. PubMed PMID: 22639650; PubMed Central PMCID: PMC3355621.

101. Gary JD, Wurmser AE, Bonangelino CJ, Weisman LS, Emr SD. Fab1p is essential for PtdIns(3)P 5-kinase activity and the maintenance of vacuolar size and membrane homeostasis. J Cell Biol. 1998;143(1):65-79. Epub 1998/10/08. PubMed PMID: 9763421; PubMed Central PMCID: PMC2132800.

102. Baars TL, Petri S, Peters C, Mayer A. Role of the V-ATPase in regulation of the vacuolar fission-fusion equilibrium. Mol Biol Cell. 2007;18(10):3873-82. Epub 2007/07/27. doi: 10.1091/mbc.E07-03-0205. PubMed PMID: 17652457; PubMed Central PMCID: PMC1995711.

103. Pei ZM, Murata Y, Benning G, Thomine S, Klusener B, Allen GJ, et al. Calcium channels activated by hydrogen peroxide mediate abscisic acid signalling in guard cells. Nature. 2000;406(6797):731-4. doi: 10.1038/35021067. PubMed PMID: 10963598.

104. Gonugunta VK, Srivastava N, Puli MR, Raghavendra AS. Nitric oxide production occurs after cytosolic alkalinization during stomatal closure induced by abscisic acid. Plant Cell Environ. 2008;31(11):1717-24. Epub 2008/08/30. doi: 10.1111/j.1365-3040.2008.01872.x. PubMed PMID: 18721267.

105. Bright J, Desikan R, Hancock JT, Weir IS, Neill SJ. ABA-induced NO generation and stomatal closure in Arabidopsis are dependent on H2O2 synthesis. Plant J. 2006;45(1):113-22. Epub 2005/12/22. doi: 10.1111/j.1365-313X.2005.02615.x. PubMed PMID: 16367958.

106. MacRobbie EA, Kurup S. Signalling mechanisms in the regulation of vacuolar ion release in guard cells. New Phytol. 2007;175(4):630-40. Epub 2007/08/11. doi: 10.1111/j.1469-8137.2007.02131.x. PubMed PMID: 17688580.

107. MacRobbie EA. ABA activates multiple Ca(2+) fluxes in stomatal guard cells, triggering vacuolar K(+)(Rb(+)) release. Proc Natl Acad Sci U S A. 2000;97(22):12361-8. Epub 2000/10/12. doi: 10.1073/pnas.220417197. PubMed PMID: 11027317; PubMed Central PMCID: PMC17347.

108. Eisinger W, Ehrhardt D, Briggs W. Microtubules are essential for guard-cell function in Vicia and Arabidopsis. Mol Plant. 2012;5(3):601-10. Epub 2012/03/10. doi: 10.1093/mp/sss002. PubMed PMID: 22402260.

109. Gilroy S, Read ND, Trewavas AJ. Elevation of cytoplasmic calcium by caged calcium or caged inositol triphosphate initiates stomatal closure. Nature. 1990;346(6286):769-71. Epub 1990/08/23. doi: 10.1038/346769a0. PubMed PMID: 2388697.

110. Chen ZH, Hills A, Lim CK, Blatt MR. Dynamic regulation of guard cell anion channels by cytosolic free Ca2+ concentration and protein phosphorylation. Plant J. 2010;61(5):816-25. Epub 2009/12/18. doi: 10.1111/j.1365-313X.2009.04108.x. PubMed PMID: 20015065.

111. Siegel RS, Xue S, Murata Y, Yang Y, Nishimura N, Wang A, et al. Calcium elevation-dependent and attenuated resting calcium-dependent abscisic acid induction of stomatal closure and abscisic acid-induced enhancement of calcium sensitivities of S-type anion and inward-rectifying K channels in Arabidopsis guard cells. Plant J. 2009;59(2):207-20. Epub 2009/03/24. doi: 10.1111/j.1365-313X.2009.03872.x. PubMed PMID: 19302418; PubMed Central PMCID: PMC2827207.

112. Distefano AM, Garcia-Mata C, Lamattina L, Laxalt AM. Nitric oxide-induced phosphatidic acid accumulation: a role for phospholipases C and D in stomatal closure. Plant Cell Environ. 2008;31(2):187-94. Epub 2007/11/13. doi: 10.1111/j.1365-3040.2007.01756.x. PubMed PMID: 17996010.

113. Dubovskaya LV, Bakakina YS, Kolesneva EV, Sodel DL, McAinsh MR, Hetherington AM, et al. cGMP-dependent ABA-induced stomatal closure in the ABA-insensitive Arabidopsis mutant abi1-1. New Phytol. 2011;191(1):57-69. Epub 2011/03/05. doi: 10.1111/j.1469-8137.2011.03661.x. PubMed PMID: 21371039.

114. Kuhn JM, Boisson-Dernier A, Dizon MB, Maktabi MH, Schroeder JI. The protein phosphatase AtPP2CA negatively regulates abscisic acid signal transduction in Arabidopsis, and effects of abh1 on AtPP2CA mRNA. Plant Physiol. 2006;140(1):127-39. Epub 2005/12/20. doi: 10.1104/pp.105.070318. PubMed PMID: 16361522; PubMed Central PMCID: PMC1326037.

115. Zhang X, Zhang L, Dong F, Gao J, Galbraith DW, Song CP. Hydrogen peroxide is involved in abscisic acid-induced stomatal closure in Vicia faba. Plant Physiol. 2001;126(4):1438-48. PubMed PMID: 11500543; PubMed Central PMCID: PMC117144.

116. Zhang W, Jeon BW, Assmann SM. Heterotrimeric G-protein regulation of ROS signalling and calcium currents in Arabidopsis guard cells. J Exp Bot. 2011;62(7):2371-9. Epub 2011/01/26. doi: 10.1093/jxb/erq424. PubMed PMID: 21262908.

117. Ng CKY, Carr K, McAinsh MR, Powell B, Hetherington AM. Drought-induced guard cell signal transduction involves sphingosine-1-phosphate. Nature. 2001;410(6828):596-9. doi: Doi 10.1038/35069092. PubMed PMID: WOS:000167859300051.

118. Murata Y, Pei ZM, Mori IC, Schroeder J. Abscisic acid activation of plasma membrane Ca^2+^ channels in guard cells requires cytosolic NAD(P)H and is differentially disrupted upstream and downstream of reactive oxygen species production in abi1-1 and abi2-1 protein phosphatase 2C mutants. Plant Cell. 2001;13(11):2513-23. PubMed PMID: 11701885; PubMed Central PMCID: PMC139468.

119. Macrobbie EA. Signalling in guard cells and regulation of ion channel activity. J Exp Bot. 1997;48 Spec No:515-28. doi: 10.1093/jxb/48.Special_Issue.515. PubMed PMID: 21245228.

120. Meimoun P, Vidal G, Bohrer AS, Lehner A, Tran D, Briand J, et al. Intracellular Ca^2+^ stores could participate to abscisic acid-induced depolarization and stomatal closure in *Arabidopsis thaliana*. Plant Signal Behav. 2009;4(9):830-5. Epub 2009/10/23. doi: 9396 [pii]. PubMed PMID: 19847112; PubMed Central PMCID: PMC2802785.

121. Jammes F, Song C, Shin D, Munemasa S, Takeda K, Gu D, et al. MAP kinases MPK9 and MPK12 are preferentially expressed in guard cells and positively regulate ROS-mediated ABA signaling. Proc Natl Acad Sci U S A. 2009;106(48):20520-5. Epub 2009/11/17. doi: 10.1073/pnas.0907205106. PubMed PMID: 19910530; PubMed Central PMCID: PMCPMC2776606.

122. Suh SJ, Wang YF, Frelet A, Leonhardt N, Klein M, Forestier C, et al. The ATP binding cassette transporter AtMRP5 modulates anion and calcium channel activities in Arabidopsis guard cells. J Biol Chem. 2007;282(3):1916-24. Epub 2006/11/14. doi: 10.1074/jbc.M607926200. PubMed PMID: 17098742.

123. Wang XQ, Ullah H, Jones AM, Assmann SM. G protein regulation of ion channels and abscisic acid signaling in Arabidopsis guard cells. Science. 2001;292(5524):2070-2. Epub 2001/06/16. doi: 10.1126/science.1059046. PubMed PMID: 11408655.

124. Distefano AM, Scuffi D, Garcia-Mata C, Lamattina L, Laxalt AM. Phospholipase Ddelta is involved in nitric oxide-induced stomatal closure. Planta. 2012;236(6):1899-907. Epub 2012/08/31. doi: 10.1007/s00425-012-1745-4. PubMed PMID: 22932846.

125. Park KY, Jung JY, Park J, Hwang JU, Kim YW, Hwang I, et al. A role for phosphatidylinositol 3-phosphate in abscisic acid-induced reactive oxygen species generation in guard cells. Plant Physiol. 2003;132(1):92-8. Epub 2003/05/15. doi: 10.1104/pp.102.016964. PubMed PMID: 12746515; PubMed Central PMCID: PMC166955.

126. Ellson CD, Gobert-Gosse S, Anderson KE, Davidson K, Erdjument-Bromage H, Tempst P, et al. PtdIns(3)P regulates the neutrophil oxidase complex by binding to the PX domain of p40(phox). Nat Cell Biol. 2001;3(7):679-82. Epub 2001/07/04. doi: 10.1038/35083076. PubMed PMID: 11433301.

127. Saito N, Munemasa S, Nakamura Y, Shimoishi Y, Mori IC, Murata Y. Roles of RCN1, regulatory A subunit of protein phosphatase 2A, in methyl jasmonate signaling and signal crosstalk between methyl jasmonate and abscisic acid. Plant Cell Physiol. 2008;49(9):1396-401. Epub 2008/07/25. doi: 10.1093/pcp/pcn106. PubMed PMID: 18650210.

128. Ng CK, Carr K, McAinsh MR, Powell B, Hetherington AM. Drought-induced guard cell signal transduction involves sphingosine-1-phosphate. Nature. 2001;410(6828):596-9. Epub 2001/03/30. doi: 10.1038/35069092. PubMed PMID: 11279499.
