# Supplementary material for: A new discrete dynamic model of ABA-induced stomatal closure predicts key feedback loops
Source: PLoS Biol. 2017 Sep 22;15(9):e2003451. doi: 10.1371/journal.pbio.2003451 (PMC5627951; doi:10.1371/journal.pbio.2003451)
Supplement: S2 Table — (DOCX) [file pbio.2003451.s003.docx]

**S2 Table. List of edges in the ABA induced closure network.**

| **Start Node** | **End Node** | **Edge sign** | **Direct, indirect or inferred** | **Reference** |
| --- | --- | --- | --- | --- |
| 8-nitro-cGMP | ADPRc | Positive | Indirect | [1] |
| ABH1 | CaIM | Negative | Inferred | [2] |
| ABI1 | pH_c_ | Negative | Inferred | [3] |
| ABI1 | RBOH | Negative | Inferred | [4] |
| ABI1 | SLAC1 | Negative | Direct | [5-7] |
| ABI1 | AtRAC1 | Positive | Inferred | [8] |
| ABI2 | GHR1 | Negative | Direct | [9] |
| ABI2 | pH_c_ | Negative | Inferred | [3] |
| ADPRc | cADPR | Positive | Direct |  |
| AGB1 | AGG3 | No sign, no direction | Direct | [10, 11] |
| AGG3 | AGB1 | No sign, no direction | Direct | [10, 11] |
| ARP Complex | Actin reorganization | Positive | Direct | [12, 13] |
| ABA | PEPC | Negative | Indirect | [14] |
| ABA | RCARs | Positive | Direct | [15, 16] |
| ABA | PI3P5K | Positive | Indirect | [17] |
| ABA | SPHK1/2 | Positive | Indirect | [18, 19] |
| Actin Reorganization | CaIM | Positive | Indirect | [20] |
| AnionEM | H_2_O Efflux | Positive | Indirect |  |
| Aquaporin (PIP2;1) | H_2_O Efflux | Positive | Direct | [21] |
| AtRAC1 | Actin Reorganization | Negative | Indirect | [8] |
| CIS | Ca^2+^_c_ | Positive | Direct | [22, 23] |
| CPK23 | SLAH3 | Positive | Direct | [24, 25] |
| CPK23 | SLAC1 | Positive | Direct | [26] |
| CPK3/21 | SLAH3 | Positive | Direct | [24, 25] |
| CPK3/21 | CPK3/21 | Positive | Direct | [27] |
| ABI1 | OST1 | Negative | Direct | [7, 28, 29] |
| CPK 6 | SLAH3 | Positive | Direct | [25] |
| CPK 6 | SLAC1 | Positive | Direct | [6] |
| Ca^2+^_c_ | MPK9/12 | Positive | Inferred | [30] |
| Ca^2+^_c_ | Ca^2+^ ATPase | Positive | Direct | [31] |
| Ca^2+^_c_ | KEV | Positive | Indirect | [32] |
| Ca^2+^_c_ | QUAC1 | Positive | Inferred | [33] |
| Ca^2+^_c_ | H^+^ ATPase | Negative | Direct | [34] |
| Ca^2+^_c_ | Depolarization | Positive | Direct | [35] |
| Ca^2+^_c_ | pH_c_ | Positive | Indirect | [3] |
| Ca^2+^_c_ | CPK 3/21 | Positive | Direct | [25] |
| Ca^2+^_c_ | PLDα | Positive | Direct | [18] |
| Ca^2+^_c_ | PLC | Positive | Direct | [36] |
| Ca^2+^_c_ | TCTP | Positive | Direct | [37] |
| Ca^2+^ ATPase | Ca^2+^_c_ | Negative | Direct | [31] |
| CaIM | Ca^2+^_c_ | Positive | Direct | [38] |
| Depolarization | KOUT | Positive | Direct | [39] |
| ERA1 | CaIM | Negative | Inferred | [40] |
| GAPC1/2 | PLDδ | Positive | Direct | [41] |
| GCR1 | GPA1 | Negative | Direct | [42] |
| GEF1/4/10 | ROP11 | Positive | Direct | [43] |
| GHR1 | SLAC1 | Positive | Direct | [9] |
| GHR1 | CaIM | Positive | Inferred | [9] |
| GPA1 | AGB1 | No sign, no direction | Direct | [11] |
| GPA1 | RBOH | Positive | Inferred | [44] |
| GPA1 | PLDα | Positive | Direct | [45] |
| GTP | cGMP | Positive | Direct | [46] |
| H^+^ ATPase | Depolarization | Negative | Direct | [47] |
| H_2_O Efflux | Closure | Positive | Direct |  |
| HAB1 | OST1 | Negative | Direct | [28, 29] |
| InsP3 | CIS | Positive | Indirect | [22] |
| InsP6 | CIS | Positive | Indirect | [23] |
| K^+^ Efflux | H_2_O Efflux | Positive | Indirect | [39] |
| K^+^ efflux | Depolarization | Negative | Direct | [39] |
| KEV | K^+^ Efflux | Positive | Direct | [32] |
| KEV | Depolarization | Positive | Direct | [32] |
| KOUT | K^+^ Efflux | Positive | Direct | [39] |
| MPK9/12 | SLAC1 | Positive | Inferred | [30] |
| MPK9/12 | MPK9/12 | Positive | Direct | [48] |
| MRP5 | CaIM | Positive | Inferred | [49] |
| Malate | H_2_O Efflux | Negative | Indirect | [50] |
| Microtubule Depolymerization | Closure | Positive | Indirect | [51] |
| Microtubule  Depolymerization | Microtubule  Depolymerization | Positive | Direct | [52] |
| NAD^+^ | cADPR | Positive | Direct | [53] |
| NADPH | ROS | Positive | Direct |  |
| NADPH | NO | Positive | Direct | [54] |
| NIA1/2 | NO | Positive | Direct | [54] |
| NO | NOGC1 | Positive | Direct | [46] |
| NO | KOUT | Negative | Direct | [55] |
| NO | PLDδ | Positive | Inferred | [56] |
| NO | 8-nitro-cGMP | Positive | Indirect | [1] |
| NOGC1 | cGMP | Positive | Direct | [46] |
| Nitrite | NO | Positive | Direct | [54] |
| NtSyp121 | CaIM | Positive | Indirect | [57] |
| OST1 | pH_c_ | Positive | Inferred | [3] |
| OST1 | QUAC1 | Positive | Direct | [58] |
| OST1 | RBOH | Positive | Direct | [59, 60] |
| PA | ABI1 | Negative | Direct | [61] |
| PA | RBOH | Positive | Direct | [62] |
| PA | SPHK1/2 | Positive | Direct | [63] |
| PC | PA | Positive | Direct | [64] |
| PEPC | Malate | Positive | Indirect | [14] |
| pH_c_ | pH_c_ | Negative | Inferred | [65-67] |
| PI3P5K | PtdIns(3,5)P2 | Positive | Direct | [17] |
| PtdIns(4,5)P2 | DAG | Positive | Direct |  |
| PtdIns(4,5)P2 | InsP3 | Positive | Direct |  |
| PLC | DAG | Positive | Direct |  |
| PLC | InsP3 | Positive | Direct |  |
| PLDα | PA | Positive | Direct | [61] |
| PLDδ | PA | Positive | Direct | [68] |
| PP2CA | OST1 | Negative | Direct | [5] |
| PP2CA | SLAC1 | Negative | Direct | [5] |
| PtdIns(3,5)P2 | V-PPase | Positive | Direct | [17] |
| PtdInsP3 | Actin | Positive | Inferred | [69] |
| PtdInsP3 | RBOH | Positive | Inferred | [69] |
| PtdInsP4 | Actin | Positive | Inferred | [70] |
| PtdInsP4 | PIP2 | Positive | Direct | [71] |
| QUAC1 | AnionEM | Positive | Direct | [72] |
| RBOH | ROS | Positive | Direct | [73] |
| RCARs | HAB1 | Negative | Direct | [74] |
| RCARs | ABI1 | Negative | Direct | [15, 16, 75] |
| RCARs | ABI2 | Negative | Direct | [15, 16] |
| RCARs | PP2CA | Negative | Direct | [76] |
| RCN1 | ROS | Positive | Inferred | [77] |
| ROP11 | ABI1 | Positive | Direct | [78] |
| ROP11 | ABI2 | Positive | Direct | [43] |
| ROS | KOUT | Negative | Indirect | [79] |
| ROS | H^+^ ATPase | Negative | Indirect | [80] |
| ROS | GHR1 | Positive | Inferred | [9] |
| ROS | ABI1 | Negative | Direct | [81] |
| ROS | NIA1/2 | Positive | Inferred | [82] |
| ROS | 8-nitro-cGMP | Positive | Indirect | [1] |
| S1P / PhytoS1P | S1P /PhytoS1P | Negative | Inferred | [19, 66, 67] |
| S1P / PhytoS1P | GPA1 | Positive | Inferred | [19] |
| SCAB1 | Actin Depolymerization | Positive | Direct | [83] |
| SLAC1 | AnionEM | Positive | Direct | [7, 84] |
| AnionEM | Depolarization | Positive | Direct | [85] |
| AnionEM | Malate | Negative | Direct | [72, 84] |
| SLAH3 | AnionEM | Positive | Direct | [24, 84] |
| SPHK1/2 | S1P/phytoS1P | Positive | Direct | [63] |
| TCTP | Microtubule Depolymerization | Positive | Direct | [37] |
| V-PPase | Vacuolar Acidification | Positive | Direct | [17] |
| Vacuolar Acidification | pH_c_ | Positive | Direct | [17] |
| Vacuolar Acidification | KEV | Positive | Indirect | [32] |
| cADPR | CIS | Positive | Indirect | [86, 87] |
| cGMP | 8-nitro-cGMP | Positive | Direct | [1] |
| pH_c_ | KOUT | Positive | Indirect | [88] |
| pH_c_ | H^+^ ATPase | Negative | Indirect | [89] |
| pH_c_ | RBOH | Positive | Inferred | [65] |
| pH_c_ | SLAC1 | Positive | Inferred | [90] |
| pH_c_ | ABI1 | Positive | Direct | [91] |
| ROS | HAB1 | Negative | Direct | [92] |
| ROS | PP2CA | Negative | Direct |  |
| Ca^2+^_c_ | V-ATPase | Positive | Indirect | [93] |
| V-ATPAse | Vacuolar Acidification | Positive | Direct | [17] |
| ROS | ABI2 | Negative | Direct | [94] |
| InsP3 | InsP6 | Positive | Direct | [95] |
| ROS | PLDδ | Positive | Inferred | [41] |
| DAGK | PA | Positive | Direct | [96] |
| DAG | PA | Positive | Direct | [96] |
| Sph | S1P/PhytoS1P | Positive | Direct | [19] |
| SPP1 | S1P/PhytoS1P | Negative | Direct | [97] |
| ABA | AtRAC1 | Negative | Indirect | [8] |
| ABI2 | OST1 | Negative | Direct | [29] |
| CPK3/21 | SLAC1 | Positive | Direct | [26] |
| OST1 | SLAC1 | Positive | Direct | [7] |
| ABI2 | SLAC1 | Negative | Direct | [26] |
| ABI1 | SLAH3 | Negative | Direct | [24] |
| ABA | Malate | Negative | Indirect | [50] |
| OST1 | Aquaporin (PIP2;1) | Positive | Direct | [21] |
| ERA1 | ROP10 | Positive | Direct | [98] |

References

1. Joudoi T, Shichiri Y, Kamizono N, Akaike T, Sawa T, Yoshitake J, et al. Nitrated cyclic GMP modulates guard cell signaling in Arabidopsis. Plant Cell. 2013;25(2):558-71. Epub 2013/02/12. doi: 10.1105/tpc.112.105049. PubMed PMID: 23396828; PubMed Central PMCID: PMC3608778.

2. Hugouvieux V, Kwak JM, Schroeder JI. An mRNA cap binding protein, ABH1, modulates early abscisic acid signal transduction in Arabidopsis. Cell. 2001;106(4):477-87. Epub 2001/08/30. PubMed PMID: 11525733.

3. Islam MM, Hossain MA, Jannat R, Munemasa S, Nakamura Y, Mori IC, et al. Cytosolic alkalization and cytosolic calcium oscillation in Arabidopsis guard cells response to ABA and MeJA. Plant Cell Physiol. 2010;51(10):1721-30. Epub 2010/08/27. doi: 10.1093/pcp/pcq131. PubMed PMID: 20739306.

4. Murata Y, Pei ZM, Mori IC, Schroeder J. Abscisic acid activation of plasma membrane Ca(2+) channels in guard cells requires cytosolic NAD(P)H and is differentially disrupted upstream and downstream of reactive oxygen species production in abi1-1 and abi2-1 protein phosphatase 2C mutants. Plant Cell. 2001;13(11):2513-23. PubMed PMID: 11701885; PubMed Central PMCID: PMC139468.

5. Lee SC, Lan W, Buchanan BB, Luan S. A protein kinase-phosphatase pair interacts with an ion channel to regulate ABA signaling in plant guard cells. Proc Natl Acad Sci U S A. 2009;106(50):21419-24. Epub 2009/12/04. doi: 10.1073/pnas.0910601106. PubMed PMID: 19955427; PubMed Central PMCID: PMC2795491.

6. Brandt B, Brodsky DE, Xue S, Negi J, Iba K, Kangasjarvi J, et al. Reconstitution of abscisic acid activation of SLAC1 anion channel by CPK6 and OST1 kinases and branched ABI1 PP2C phosphatase action. Proc Natl Acad Sci U S A. 2012;109(26):10593-8. Epub 2012/06/13. doi: 10.1073/pnas.1116590109. PubMed PMID: 22689970; PubMed Central PMCID: PMC3387046.

7. Geiger D, Scherzer S, Mumm P, Stange A, Marten I, Bauer H, et al. Activity of guard cell anion channel SLAC1 is controlled by drought-stress signaling kinase-phosphatase pair. Proc Natl Acad Sci U S A. 2009;106(50):21425-30. Epub 2009/12/04. doi: 10.1073/pnas.0912021106. PubMed PMID: 19955405; PubMed Central PMCID: PMC2795561.

8. Lemichez E, Wu Y, Sanchez JP, Mettouchi A, Mathur J, Chua NH. Inactivation of AtRac1 by abscisic acid is essential for stomatal closure. Genes Dev. 2001;15(14):1808-16. Epub 2001/07/19. doi: 10.1101/gad.900401. PubMed PMID: 11459830; PubMed Central PMCID: PMC312738.

9. Hua D, Wang C, He J, Liao H, Duan Y, Zhu Z, et al. A plasma membrane receptor kinase, GHR1, mediates abscisic acid- and hydrogen peroxide-regulated stomatal movement in Arabidopsis. Plant Cell. 2012;24(6):2546-61. Epub 2012/06/26. doi: 10.1105/tpc.112.100107. PubMed PMID: 22730405; PubMed Central PMCID: PMC3406912.

10. Chakravorty D, Trusov Y, Zhang W, Acharya BR, Sheahan MB, McCurdy DW, et al. An atypical heterotrimeric G-protein gamma-subunit is involved in guard cell K^+^-channel regulation and morphological development in Arabidopsis thaliana. Plant J. 2011;67(5):840-51. Epub 2011/05/18. doi: 10.1111/j.1365-313X.2011.04638.x. PubMed PMID: 21575088.

11. Gookin TE, Assmann SM. Significant reduction of BiFC non-specific assembly facilitates in planta assessment of heterotrimeric G-protein interactors. Plant J. 2014;80(3):553-67. Epub 2014/09/05. doi: 10.1111/tpj.12639. PubMed PMID: 25187041; PubMed Central PMCID: PMCPMC4260091.

12. Jiang K, Sorefan K, Deeks MJ, Bevan MW, Hussey PJ, Hetherington AM. The ARP2/3 complex mediates guard cell actin reorganization and stomatal movement in Arabidopsis. Plant Cell. 2012;24(5):2031-40. Epub 2012/05/10. doi: 10.1105/tpc.112.096263. PubMed PMID: 22570440; PubMed Central PMCID: PMC3442585.

13. Szymanski DB. Breaking the WAVE complex: the point of Arabidopsis trichomes. Curr Opin Plant Biol. 2005;8(1):103-12. doi: 10.1016/j.pbi.2004.11.004. PubMed PMID: 15653407.

14. Du Z, Aghoram K, Outlaw WH, Jr. In vivo phosphorylation of phosphoenolpyruvate carboxylase in guard cells of Vicia faba L. is enhanced by fusicoccin and suppressed by abscisic acid. Arch Biochem Biophys. 1997;337(2):345-50. Epub 1997/01/15. PubMed PMID: 9016832.

15. Ma Y, Szostkiewicz I, Korte A, Moes D, Yang Y, Christmann A, et al. Regulators of PP2C phosphatase activity function as abscisic acid sensors. Science. 2009;324(5930):1064-8. Epub 2009/05/02. doi: 10.1126/science.1172408. PubMed PMID: 19407143.

16. Park SY, Fung P, Nishimura N, Jensen DR, Fujii H, Zhao Y, et al. Abscisic acid inhibits type 2C protein phosphatases via the PYR/PYL family of START proteins. Science. 2009;324(5930):1068-71. Epub 2009/05/02. doi: 10.1126/science.1173041. PubMed PMID: 19407142; PubMed Central PMCID: PMC2827199.

17. Bak G, Lee EJ, Lee Y, Kato M, Segami S, Sze H, et al. Rapid structural changes and acidification of guard cell vacuoles during stomatal closure require phosphatidylinositol 3,5-bisphosphate. Plant Cell. 2013;25(6):2202-16. Epub 2013/06/13. doi: 10.1105/tpc.113.110411. PubMed PMID: 23757398; PubMed Central PMCID: PMC3723621.

18. Guo L, Wang X. Crosstalk between Phospholipase D and Sphingosine Kinase in Plant Stress Signaling. Front Plant Sci. 2012;3:51. Epub 2012/05/29. doi: 10.3389/fpls.2012.00051. PubMed PMID: 22639650; PubMed Central PMCID: PMC3355621.

19. Coursol S, Fan LM, Le Stunff H, Spiegel S, Gilroy S, Assmann SM. Sphingolipid signalling in Arabidopsis guard cells involves heterotrimeric G proteins. Nature. 2003;423(6940):651-4. Epub 2003/06/06. doi: 10.1038/nature01643. PubMed PMID: 12789341.

20. Zhang W, Fan LM, Wu WH. Osmo-sensitive and stretch-activated calcium-permeable channels in Vicia faba guard cells are regulated by actin dynamics. Plant Physiol. 2007;143(3):1140-51. Epub 2007/01/30. doi: 10.1104/pp.106.091405. PubMed PMID: 17259289; PubMed Central PMCID: PMC1820927.

21. Grondin A, Rodrigues O, Verdoucq L, Merlot S, Leonhardt N, Maurel C. Aquaporins Contribute to ABA-Triggered Stomatal Closure through OST1-Mediated Phosphorylation. Plant Cell. 2015;27(7):1945-54. Epub 2015/07/15. doi: 10.1105/tpc.15.00421. PubMed PMID: 26163575; PubMed Central PMCID: PMC4531361.

22. Staxen I, Pical C, Montgomery LT, Gray JE, Hetherington AM, McAinsh MR. Abscisic acid induces oscillations in guard-cell cytosolic free calcium that involve phosphoinositide-specific phospholipase C. Proc Natl Acad Sci U S A. 1999;96(4):1779-84. PubMed PMID: 9990101; PubMed Central PMCID: PMC15593.

23. Lemtiri-Chlieh F, MacRobbie EA, Webb AA, Manison NF, Brownlee C, Skepper JN, et al. Inositol hexakisphosphate mobilizes an endomembrane store of calcium in guard cells. Proc Natl Acad Sci U S A. 2003;100(17):10091-5. Epub 2003/08/13. doi: 10.1073/pnas.1133289100. PubMed PMID: 12913129; PubMed Central PMCID: PMC187775.

24. Geiger D, Maierhofer T, Al-Rasheid KA, Scherzer S, Mumm P, Liese A, et al. Stomatal closure by fast abscisic acid signaling is mediated by the guard cell anion channel SLAH3 and the receptor RCAR1. Sci Signal. 2011;4(173):ra32. Epub 2011/05/19. doi: 10.1126/scisignal.2001346. PubMed PMID: 21586729.

25. Scherzer S, Maierhofer T, Al-Rasheid KA, Geiger D, Hedrich R. Multiple calcium-dependent kinases modulate ABA-activated guard cell anion channels. Mol Plant. 2012;5(6):1409-12. Epub 2012/08/31. doi: 10.1093/mp/sss084. PubMed PMID: 22933711.

26. Geiger D, Scherzer S, Mumm P, Marten I, Ache P, Matschi S, et al. Guard cell anion channel SLAC1 is regulated by CDPK protein kinases with distinct Ca2+ affinities. Proc Natl Acad Sci U S A. 2010;107(17):8023-8. Epub 2010/04/14. doi: 10.1073/pnas.0912030107. PubMed PMID: 20385816; PubMed Central PMCID: PMC2867891.

27. Swatek KN, Wilson RS, Ahsan N, Tritz RL, Thelen JJ. Multisite phosphorylation of 14-3-3 proteins by calcium-dependent protein kinases. Biochem J. 2014;459(1):15-25. Epub 2014/01/21. doi: 10.1042/BJ20130035. PubMed PMID: 24438037; PubMed Central PMCID: PMC4127189.

28. Umezawa T, Sugiyama N, Mizoguchi M, Hayashi S, Myouga F, Yamaguchi-Shinozaki K, et al. Type 2C protein phosphatases directly regulate abscisic acid-activated protein kinases in Arabidopsis. Proc Natl Acad Sci U S A. 2009;106(41):17588-93. doi: 10.1073/pnas.0907095106. PubMed PMID: 19805022; PubMed Central PMCID: PMC2754379.

29. Vlad F, Rubio S, Rodrigues A, Sirichandra C, Belin C, Robert N, et al. Protein phosphatases 2C regulate the activation of the Snf1-related kinase OST1 by abscisic acid in Arabidopsis. Plant Cell. 2009;21(10):3170-84. doi: 10.1105/tpc.109.069179. PubMed PMID: 19855047; PubMed Central PMCID: PMCPMC2782292.

30. Jammes F, Song C, Shin D, Munemasa S, Takeda K, Gu D, et al. MAP kinases MPK9 and MPK12 are preferentially expressed in guard cells and positively regulate ROS-mediated ABA signaling. Proc Natl Acad Sci U S A. 2009;106(48):20520-5. Epub 2009/11/17. doi: 10.1073/pnas.0907205106. PubMed PMID: 19910530; PubMed Central PMCID: PMC2776606.

31. Sanders D, Pelloux J, Brownlee C, Harper JF. Calcium at the crossroads of signaling. Plant Cell. 2002;14 Suppl:S401-17. PubMed PMID: 12045291; PubMed Central PMCID: PMC151269.

32. Ward JM, Schroeder JI. Calcium-Activated K+ Channels and Calcium-Induced Calcium Release by Slow Vacuolar Ion Channels in Guard Cell Vacuoles Implicated in the Control of Stomatal Closure. Plant Cell. 1994;6(5):669-83. doi: 10.1105/tpc.6.5.669. PubMed PMID: 12244253; PubMed Central PMCID: PMC160467.

33. Sasaki T, Mori IC, Furuichi T, Munemasa S, Toyooka K, Matsuoka K, et al. Closing plant stomata requires a homolog of an aluminum-activated malate transporter. Plant Cell Physiol. 2010;51(3):354-65. Epub 2010/02/16. doi: 10.1093/pcp/pcq016. PubMed PMID: 20154005; PubMed Central PMCID: PMC2835873.

34. Kinoshita T, Nishimura M, Shimazaki K. Cytosolic Concentration of Ca2+ Regulates the Plasma Membrane H+-ATPase in Guard Cells of Fava Bean. Plant Cell. 1995;7(8):1333-42. doi: 10.1105/tpc.7.8.1333. PubMed PMID: 12242406; PubMed Central PMCID: PMC160955.

35. Pei ZM, Baizabal-Aguirre VM, Allen GJ, Schroeder JI. A transient outward-rectifying K+ channel current down-regulated by cytosolic Ca2+ in Arabidopsis thaliana guard cells. Proc Natl Acad Sci U S A. 1998;95(11):6548-53. PubMed PMID: 9601004; PubMed Central PMCID: PMC27872.

36. Otterhag L, Sommarin M, Pical C. N-terminal EF-hand-like domain is required for phosphoinositide-specific phospholipase C activity in Arabidopsis thaliana. FEBS Lett. 2001;497(2-3):165-70. Epub 2001/05/30. doi: S0014-5793(01)02453-X [pii]. PubMed PMID: 11377433.

37. Kim YM, Han YJ, Hwang OJ, Lee SS, Shin AY, Kim SY, et al. Overexpression of Arabidopsis translationally controlled tumor protein gene AtTCTP enhances drought tolerance with rapid ABA-induced stomatal closure. Mol Cells. 2012;33(6):617-26. Epub 2012/05/23. doi: 10.1007/s10059-012-0080-8. PubMed PMID: 22610367; PubMed Central PMCID: PMCPMC3887759.

38. Schroeder JI, Hagiwara S. Repetitive increases in cytosolic Ca^2+^ of guard cells by abscisic acid activation of nonselective Ca2+ permeable channels. Proc Natl Acad Sci U S A. 1990;87(23):9305-9. PubMed PMID: 2174559; PubMed Central PMCID: PMC55153.

39. Hosy E, Vavasseur A, Mouline K, Dreyer I, Gaymard F, Poree F, et al. The Arabidopsis outward K+ channel GORK is involved in regulation of stomatal movements and plant transpiration. Proc Natl Acad Sci U S A. 2003;100(9):5549-54. doi: 10.1073/pnas.0733970100. PubMed PMID: 12671068; PubMed Central PMCID: PMC154382.

40. Allen GJ, Murata Y, Chu SP, Nafisi M, Schroeder JI. Hypersensitivity of abscisic acid-induced cytosolic calcium increases in the Arabidopsis farnesyltransferase mutant era1-2. Plant Cell. 2002;14(7):1649-62. Epub 2002/07/18. PubMed PMID: 12119381; PubMed Central PMCID: PMC150713.

41. Guo L, Devaiah SP, Narasimhan R, Pan X, Zhang Y, Zhang W, et al. Cytosolic glyceraldehyde-3-phosphate dehydrogenases interact with phospholipase Ddelta to transduce hydrogen peroxide signals in the Arabidopsis response to stress. Plant Cell. 2012;24(5):2200-12. Epub 2012/05/17. doi: 10.1105/tpc.111.094946. PubMed PMID: 22589465; PubMed Central PMCID: PMC3442596.

42. Pandey S, Assmann SM. The Arabidopsis putative G protein-coupled receptor GCR1 interacts with the G protein alpha subunit GPA1 and regulates abscisic acid signaling. Plant Cell. 2004;16(6):1616-32. doi: 10.1105/tpc.020321. PubMed PMID: 15155892; PubMed Central PMCID: PMC490050.

43. Yu F, Qian L, Nibau C, Duan Q, Kita D, Levasseur K, et al. FERONIA receptor kinase pathway suppresses abscisic acid signaling in Arabidopsis by activating ABI2 phosphatase. Proc Natl Acad Sci U S A. 2012;109(36):14693-8. Epub 2012/08/22. doi: 10.1073/pnas.1212547109. PubMed PMID: 22908257; PubMed Central PMCID: PMC3437822.

44. Zhang W, Jeon BW, Assmann SM. Heterotrimeric G-protein regulation of ROS signalling and calcium currents in Arabidopsis guard cells. J Exp Bot. 2011;62(7):2371-9. Epub 2011/01/26. doi: 10.1093/jxb/erq424. PubMed PMID: 21262908.

45. Zhao J, Wang X. Arabidopsis phospholipase Dalpha1 interacts with the heterotrimeric G-protein alpha-subunit through a motif analogous to the DRY motif in G-protein-coupled receptors. J Biol Chem. 2004;279(3):1794-800. doi: 10.1074/jbc.M309529200. PubMed PMID: 14594812.

46. Mulaudzi T, Ludidi N, Ruzvidzo O, Morse M, Hendricks N, Iwuoha E, et al. Identification of a novel Arabidopsis thaliana nitric oxide-binding molecule with guanylate cyclase activity in vitro. FEBS Lett. 2011;585(17):2693-7. Epub 2011/08/09. doi: 10.1016/j.febslet.2011.07.023. PubMed PMID: 21820435.

47. Merlot S, Leonhardt N, Fenzi F, Valon C, Costa M, Piette L, et al. Constitutive activation of a plasma membrane H(+)-ATPase prevents abscisic acid-mediated stomatal closure. EMBO J. 2007;26(13):3216-26. Epub 2007/06/09. doi: 10.1038/sj.emboj.7601750. PubMed PMID: 17557075; PubMed Central PMCID: PMC1914098.

48. Nagy SK, Darula Z, Kallai BM, Bogre L, Banhegyi G, Medzihradszky KF, et al. Activation of AtMPK9 through autophosphorylation that makes it independent of the canonical MAPK cascades. Biochem J. 2015;467(1):167-75. Epub 2015/02/04. doi: 10.1042/BJ20141176. PubMed PMID: 25646663.

49. Suh SJ, Wang YF, Frelet A, Leonhardt N, Klein M, Forestier C, et al. The ATP binding cassette transporter AtMRP5 modulates anion and calcium channel activities in Arabidopsis guard cells. J Biol Chem. 2007;282(3):1916-24. Epub 2006/11/14. doi: 10.1074/jbc.M607926200. PubMed PMID: 17098742.

50. Dittrich P, Raschke K. Malate metabolism in isolated epidermis of Commelina communis L. in relation to stomatal functioning. Planta. 1977;134(1):77-81. Epub 1977/01/01. doi: 10.1007/BF00390098. PubMed PMID: 24419583.

51. Jiang Y, Wu K, Lin F, Qu Y, Liu X, Zhang Q. Phosphatidic acid integrates calcium signaling and microtubule dynamics into regulating ABA-induced stomatal closure in Arabidopsis. Planta. 2014;239(3):565-75. doi: 10.1007/s00425-013-1999-5. PubMed PMID: 24271006.

52. Gardner MK, Zanic M, Howard J. Microtubule catastrophe and rescue. Curr Opin Cell Biol. 2013;25(1):14-22. Epub 2012/10/25. doi: 10.1016/j.ceb.2012.09.006. PubMed PMID: 23092753; PubMed Central PMCID: PMC3556214.

53. Hunt L, Lerner F, Ziegler M. NAD - new roles in signalling and gene regulation in plants. New Phytol. 2004;163(1):31-44. doi: 10.1111/j.1469-8137.2004.01087.x. PubMed PMID: WOS:000221977600005.

54. Desikan R, Griffiths R, Hancock J, Neill S. A new role for an old enzyme: nitrate reductase-mediated nitric oxide generation is required for abscisic acid-induced stomatal closure in Arabidopsis thaliana. Proc Natl Acad Sci U S A. 2002;99(25):16314-8. Epub 2002/11/26. doi: 10.1073/pnas.252461999. PubMed PMID: 12446847; PubMed Central PMCID: PMC138608.

55. Sokolovski S, Blatt MR. Nitric oxide block of outward-rectifying K+ channels indicates direct control by protein nitrosylation in guard cells. Plant Physiol. 2004;136(4):4275-84. doi: 10.1104/pp.104.050344. PubMed PMID: 15563619; PubMed Central PMCID: PMC535857.

56. Distefano AM, Scuffi D, Garcia-Mata C, Lamattina L, Laxalt AM. Phospholipase Ddelta is involved in nitric oxide-induced stomatal closure. Planta. 2012;236(6):1899-907. Epub 2012/08/31. doi: 10.1007/s00425-012-1745-4. PubMed PMID: 22932846.

57. Sokolovski S, Hills A, Gay RA, Blatt MR. Functional interaction of the SNARE protein NtSyp121 in Ca2+ channel gating, Ca2+ transients and ABA signalling of stomatal guard cells. Mol Plant. 2008;1(2):347-58. Epub 2008/03/01. doi: 10.1093/mp/ssm029. PubMed PMID: 19825544.

58. Imes D, Mumm P, Bohm J, Al-Rasheid KA, Marten I, Geiger D, et al. Open stomata 1 (OST1) kinase controls R-type anion channel QUAC1 in Arabidopsis guard cells. Plant J. 2013;74(3):372-82. Epub 2013/03/05. doi: 10.1111/tpj.12133. PubMed PMID: 23452338.

59. Sirichandra C, Gu D, Hu HC, Davanture M, Lee S, Djaoui M, et al. Phosphorylation of the Arabidopsis AtrbohF NADPH oxidase by OST1 protein kinase. FEBS Lett. 2009;583(18):2982-6. Epub 2009/09/01. doi: 10.1016/j.febslet.2009.08.033. PubMed PMID: 19716822.

60. Acharya BR, Jeon BW, Zhang W, Assmann SM. Open Stomata 1 (OST1) is limiting in abscisic acid responses of Arabidopsis guard cells. New Phytol. 2013;200(4):1049-63. Epub 2013/09/17. doi: 10.1111/nph.12469. PubMed PMID: 24033256.

61. Zhang W, Qin C, Zhao J, Wang X. Phospholipase D alpha 1-derived phosphatidic acid interacts with ABI1 phosphatase 2C and regulates abscisic acid signaling. Proc Natl Acad Sci U S A. 2004;101(25):9508-13. Epub 2004/06/16. doi: 10.1073/pnas.0402112101. PubMed PMID: 15197253; PubMed Central PMCID: PMC439007.

62. Zhang Y, Zhu H, Zhang Q, Li M, Yan M, Wang R, et al. Phospholipase dalpha1 and phosphatidic acid regulate NADPH oxidase activity and production of reactive oxygen species in ABA-mediated stomatal closure in Arabidopsis. Plant Cell. 2009;21(8):2357-77. Epub 2009/08/20. doi: 10.1105/tpc.108.062992. PubMed PMID: 19690149; PubMed Central PMCID: PMC2751945.

63. Guo L, Mishra G, Taylor K, Wang X. Phosphatidic acid binds and stimulates Arabidopsis sphingosine kinases. J Biol Chem. 2011;286(15):13336-45. Epub 2011/02/19. doi: 10.1074/jbc.M110.190892. PubMed PMID: 21330371; PubMed Central PMCID: PMC3075680.

64. Pappan KL, Wang X. Assaying different types of plant phospholipase D activities in vitro. Methods Mol Biol. 2013;1009:205-17. doi: 10.1007/978-1-62703-401-2_19. PubMed PMID: 23681536.

65. Suhita D, Raghavendra AS, Kwak JM, Vavasseur A. Cytoplasmic alkalization precedes reactive oxygen species production during methyl jasmonate- and abscisic acid-induced stomatal closure. Plant Physiol. 2004;134(4):1536-45. doi: 10.1104/pp.103.032250. PubMed PMID: 15064385; PubMed Central PMCID: PMC419829.

66. Li S, Assmann SM, Albert R. Predicting essential components of signal transduction networks: a dynamic model of guard cell abscisic acid signaling. PLoS Biol. 2006;4(10):e312. Epub 2006/09/14. doi: 10.1371/journal.pbio.0040312. PubMed PMID: 16968132; PubMed Central PMCID: PMC1564158.

67. Puli MR, Rajsheel P, Aswani V, Agurla S, Kuchitsu K, Raghavendra AS. Stomatal closure induced by phytosphingosine-1-phosphate and sphingosine-1-phosphate depends on nitric oxide and pH of guard cells in Pisum sativum. Planta. 2016;244(4):831-41. Epub 2016/05/29. doi: 10.1007/s00425-016-2545-z. PubMed PMID: 27233507.

68. Uraji M, Katagiri T, Okuma E, Ye W, Hossain MA, Masuda C, et al. Cooperative function of PLDdelta and PLDalpha1 in abscisic acid-induced stomatal closure in Arabidopsis. Plant Physiol. 2012;159(1):450-60. Epub 2012/03/07. doi: 10.1104/pp.112.195578. PubMed PMID: 22392280; PubMed Central PMCID: PMC3375977.

69. Choi Y, Lee Y, Jeon BW, Staiger CJ, Lee Y. Phosphatidylinositol 3- and 4-phosphate modulate actin filament reorganization in guard cells of day flower. Plant Cell Environ. 2008;31(3):366-77. Epub 2007/12/20. doi: 10.1111/j.1365-3040.2007.01769.x. PubMed PMID: 18088331.

70. Park KY, Jung JY, Park J, Hwang JU, Kim YW, Hwang I, et al. A role for phosphatidylinositol 3-phosphate in abscisic acid-induced reactive oxygen species generation in guard cells. Plant Physiol. 2003;132(1):92-8. Epub 2003/05/15. doi: 10.1104/pp.102.016964. PubMed PMID: 12746515; PubMed Central PMCID: PMC166955.

71. Jung JY, Kim YW, Kwak JM, Hwang JU, Young J, Schroeder JI, et al. Phosphatidylinositol 3- and 4-phosphate are required for normal stomatal movements. Plant Cell. 2002;14(10):2399-412. Epub 2002/10/09. PubMed PMID: 12368494; PubMed Central PMCID: PMC151225.

72. Meyer S, Mumm P, Imes D, Endler A, Weder B, Al-Rasheid KA, et al. AtALMT12 represents an R-type anion channel required for stomatal movement in Arabidopsis guard cells. Plant J. 2010;63(6):1054-62. Epub 2010/07/16. doi: 10.1111/j.1365-313X.2010.04302.x. PubMed PMID: 20626656.

73. Kwak JM, Mori IC, Pei ZM, Leonhardt N, Torres MA, Dangl JL, et al. NADPH oxidase AtrbohD and AtrbohF genes function in ROS-dependent ABA signaling in Arabidopsis. EMBO J. 2003;22(11):2623-33. Epub 2003/05/30. doi: 10.1093/emboj/cdg277. PubMed PMID: 12773379; PubMed Central PMCID: PMC156772.

74. Santiago J, Rodrigues A, Saez A, Rubio S, Antoni R, Dupeux F, et al. Modulation of drought resistance by the abscisic acid receptor PYL5 through inhibition of clade A PP2Cs. Plant J. 2009;60(4):575-88. doi: 10.1111/j.1365-313X.2009.03981.x. PubMed PMID: 19624469.

75. Nishimura N, Sarkeshik A, Nito K, Park SY, Wang A, Carvalho PC, et al. PYR/PYL/RCAR family members are major in-vivo ABI1 protein phosphatase 2C-interacting proteins in Arabidopsis. Plant J. 2010;61(2):290-9. Epub 2009/10/31. doi: 10.1111/j.1365-313X.2009.04054.x. PubMed PMID: 19874541; PubMed Central PMCID: PMC2807913.

76. Antoni R, Gonzalez-Guzman M, Rodriguez L, Rodrigues A, Pizzio GA, Rodriguez PL. Selective inhibition of clade A phosphatases type 2C by PYR/PYL/RCAR abscisic acid receptors. Plant Physiol. 2012;158(2):970-80. Epub 2011/12/27. doi: 10.1104/pp.111.188623. PubMed PMID: 22198272; PubMed Central PMCID: PMC3271782.

77. Saito N, Munemasa S, Nakamura Y, Shimoishi Y, Mori IC, Murata Y. Roles of RCN1, regulatory A subunit of protein phosphatase 2A, in methyl jasmonate signaling and signal crosstalk between methyl jasmonate and abscisic acid. Plant Cell Physiol. 2008;49(9):1396-401. Epub 2008/07/25. doi: 10.1093/pcp/pcn106. PubMed PMID: 18650210.

78. Li Z, Gao X, Chinnusamy V, Bressan R, Wang ZX, Zhu JK, et al. ROP11 GTPase negatively regulates ABA signaling by protecting ABI1 phosphatase activity from inhibition by the ABA receptor RCAR1/PYL9 in Arabidopsis. J Integr Plant Biol. 2012;54(3):180-8. Epub 2012/01/19. doi: 10.1111/j.1744-7909.2012.01101.x. PubMed PMID: 22251383; PubMed Central PMCID: PMC3586988.

79. Kohler B, Hills A, Blatt MR. Control of guard cell ion channels by hydrogen peroxide and abscisic acid indicates their action through alternate signaling pathways. Plant Physiol. 2003;131(2):385-8. doi: 10.1104/pp.016014. PubMed PMID: 12586862; PubMed Central PMCID: PMC1540280.

80. Zhang X, Wang H, Takemiya A, Song CP, Kinoshita T, Shimazaki K. Inhibition of blue light-dependent H+ pumping by abscisic acid through hydrogen peroxide-induced dephosphorylation of the plasma membrane H+-ATPase in guard cell protoplasts. Plant Physiol. 2004;136(4):4150-8. doi: 10.1104/pp.104.046573. PubMed PMID: 15563626; PubMed Central PMCID: PMC535845.

81. Meinhard M, Grill E. Hydrogen peroxide is a regulator of ABI1, a protein phosphatase 2C from Arabidopsis. FEBS Lett. 2001;508(3):443-6. PubMed PMID: 11728469.

82. Bright J, Desikan R, Hancock JT, Weir IS, Neill SJ. ABA-induced NO generation and stomatal closure in Arabidopsis are dependent on H2O2 synthesis. Plant J. 2006;45(1):113-22. Epub 2005/12/22. doi: 10.1111/j.1365-313X.2005.02615.x. PubMed PMID: 16367958.

83. Zhao Y, Zhao S, Mao T, Qu X, Cao W, Zhang L, et al. The plant-specific actin binding protein SCAB1 stabilizes actin filaments and regulates stomatal movement in Arabidopsis. Plant Cell. 2011;23(6):2314-30. Epub 2011/07/02. doi: 10.1105/tpc.111.086546. PubMed PMID: 21719691; PubMed Central PMCID: PMC3160031.

84. Hedrich R. Ion channels in plants. Physiol Rev. 2012;92(4):1777-811. Epub 2012/10/18. doi: 10.1152/physrev.00038.2011. PubMed PMID: 23073631.

85. Levchenko V, Konrad KR, Dietrich P, Roelfsema MR, Hedrich R. Cytosolic abscisic acid activates guard cell anion channels without preceding Ca2+ signals. Proc Natl Acad Sci U S A. 2005;102(11):4203-8. Epub 2005/03/09. doi: 10.1073/pnas.0500146102. PubMed PMID: 15753314; PubMed Central PMCID: PMC554796.

86. Guse AH. Cyclic ADP-ribose: a novel Ca2+-mobilising second messenger. Cell Signal. 1999;11(5):309-16. Epub 1999/06/22. PubMed PMID: 10376802.

87. Leckie CP, McAinsh MR, Allen GJ, Sanders D, Hetherington AM. Abscisic acid-induced stomatal closure mediated by cyclic ADP-ribose. Proc Natl Acad Sci U S A. 1998;95(26):15837-42. Epub 1998/12/23. PubMed PMID: 9861057; PubMed Central PMCID: PMC28131.

88. Miedema H, Assmann SM. A membrane-delimited effect of internal pH on the K+ outward rectifier of Vicia faba guard cells. J Membr Biol. 1996;154(3):227-37. PubMed PMID: 8952952.

89. Luo H, Morsomme P, Boutry M. The two major types of plant plasma membrane H+-ATPases show different enzymatic properties and confer differential pH sensitivity of yeast growth. Plant Physiol. 1999;119(2):627-34. PubMed PMID: 9952459; PubMed Central PMCID: PMC32140.

90. Wang XQ, Ullah H, Jones AM, Assmann SM. G protein regulation of ion channels and abscisic acid signaling in Arabidopsis guard cells. Science. 2001;292(5524):2070-2. Epub 2001/06/16. doi: 10.1126/science.1059046. PubMed PMID: 11408655.

91. Leube MP, Grill E, Amrhein N. ABI1 of Arabidopsis is a protein serine/threonine phosphatase highly regulated by the proton and magnesium ion concentration. FEBS Lett. 1998;424(1-2):100-4. Epub 1998/04/16. PubMed PMID: 9537523.

92. Sridharamurthy M, Kovach A, Zhao Y, Zhu JK, Xu HE, Swaminathan K, et al. H2O2 inhibits ABA-signaling protein phosphatase HAB1. PLoS One. 2014;9(12):e113643. doi: 10.1371/journal.pone.0113643. PubMed PMID: 25460914; PubMed Central PMCID: PMC4252038.

93. Tang RJ, Liu H, Yang Y, Yang L, Gao XS, Garcia VJ, et al. Tonoplast calcium sensors CBL2 and CBL3 control plant growth and ion homeostasis through regulating V-ATPase activity in Arabidopsis. Cell Res. 2012;22(12):1650-65. doi: 10.1038/cr.2012.161. PubMed PMID: 23184060; PubMed Central PMCID: PMC3515760.

94. Meinhard M, Rodriguez PL, Grill E. The sensitivity of ABI2 to hydrogen peroxide links the abscisic acid-response regulator to redox signalling. Planta. 2002;214(5):775-82. doi: 10.1007/s00425-001-0675-3. PubMed PMID: 11882947.

95. Boss WF, Im YJ. Phosphoinositide signaling. Annu Rev Plant Biol. 2012;63:409-29. Epub 2012/03/13. doi: 10.1146/annurev-arplant-042110-103840. PubMed PMID: 22404474.

96. Munnik T, Irvine RF, Musgrave A. Phospholipid signalling in plants. Biochim Biophys Acta. 1998;1389(3):222-72. PubMed PMID: 9512651.

97. Nakagawa N, Kato M, Takahashi Y, Shimazaki K, Tamura K, Tokuji Y, et al. Degradation of long-chain base 1-phosphate (LCBP) in Arabidopsis: functional characterization of LCBP phosphatase involved in the dehydration stress response. J Plant Res. 2012;125(3):439-49. Epub 2011/09/13. doi: 10.1007/s10265-011-0451-9. PubMed PMID: 21910031.

98. Zheng ZL, Nafisi M, Tam A, Li H, Crowell DN, Chary SN, et al. Plasma membrane-associated ROP10 small GTPase is a specific negative regulator of abscisic acid responses in Arabidopsis. Plant Cell. 2002;14(11):2787-97. PubMed PMID: 12417701; PubMed Central PMCID: PMC152727.
