# Supplementary material for: A new discrete dynamic model of ABA-induced stomatal closure predicts key feedback loops
Source: PLoS Biol. 2017 Sep 22;15(9):e2003451. doi: 10.1371/journal.pbio.2003451 (PMC5627951; doi:10.1371/journal.pbio.2003451)
Supplement: S3 Table — (DOCX) [file pbio.2003451.s004.docx]

**S3 Table. The full name of the abbreviated node names in the ABA induced closure network.**

| **Node name in the network** | **Full name** |
| --- | --- |
| 8-nitro-cGMP | 8-nitro-Cyclic guanosine monophosphate |
| ABA | Abscisic acid |
| ABH1 | ABA hypersensitive |
| ABI1 | ABA (abscisic acid)-insensitive 1 |
| ABI2 | ABA (abscisic acid)-insensitive 2 |
| Actin Reorganization | Actin reorganization |
| ADPRc | ADP (adenosine diphosphate)-ribosyl cyclase |
| AGB1 | Arabidopsis G-protein beta subunit1 |
| AGG3 | Arabidopsis G protein gamma subunit 3 |
| AnionEM | Anion efflux through the plasma membrane |
| ARP Complex | Actin related protein complex |
| AtRAC1 | small GTPase RAC1 |
| Ca^2+^_c_ | Cytosolic calcium |
| Ca^2+^ ATPase | Ca^2+^ ATPases and Ca^2+/^H^+^ antiporters responsible for Ca^2+^ efflux from the cytosol |
| cADPR | cyclic ADP-ribose |
| CaIM | Ca^2+^ influx across the plasma membrane |
| cGMP | Cyclic guanosine monophosphate |
| CIS | Ca^2+^ influx to the cytosol from intracellular stores |
| Closure | Stomatal closure |
| CPK23 | Calcium-dependent protein kinase 23 |
| CPK3/21 | Calcium-dependent protein kinases 3 and 21 |
| CPK6 | Calcium-dependent protein kinase 6 |
| DAG | Diacylglycerol |
| DAGK | Diacylglycerol kinase |
| Depolarization | Plasma membrane depolarization |
| ERA1 | Enhanced Response to Abscisic acid1 |
| GAPC1/2 | Glyceraldehyde-3-phosphate dehydrogenase subunits 1 and 2 |
| GCR1 | putative G protein–coupled receptor |
| GEF1/4/10 | Guanine exchange factors 1, 4 and 10 |
| GHR1 | Guard cell hydrogen peroxide resistant 1 |
| GPA1 | Heterotrimeric G protein α subunit 1 |
| GTP | Guanosine 5'-triphosphate |
| H^+^ ATPase | H^+^ ATPase at the plasma membrane |
| H_2_O Efflux | water efflux through the plasma membrane |
| HAB1 | Hypersensitive to ABA 1 |
| InsP3 | Inositol-1,4,5 trisphosphate |
| InsP6 | Inositol hexakisphosphate |
| K^+^ Efflux | K^+^ efflux through the plasma membrane |
|  |  |
| KEV | K^+^ efflux from the vacuole to the cytosol |
| KOUT | K^+^ efflux through slowly activating outwardly-rectifying K^+^ channels through the plasma membrane |
| Malate | Malate |
| Microtubule Depolymerization | Microtubule depolymerization |
| MPK9/12 | Mitogen-activated protein kinases 9 and 12 |
| MRP5 | Multidrug Resistance-associated Protein 5 |
| NAD^+^ | Nicotinamide adenine dinucleotide |
| NADPH | Nicotinamide adenine dinucleotide phosphate |
| NIA1/2 | Nitrate reductase 1/2 |
| Nitrite | Nitrite |
| NO | Nitric Oxide |
| NOGC1 | Nitric Oxide dependent Guanylate Cyclase |
| NtSyp121 | Tobacco syntaxin-like SNARE (soluble N-ethylmaleimide-sensitive factor) attachment protein receptors |
| OST1 | protein kinase *O*PEN *ST*OMATA 1 |
| PA | Phosphatidic acid |
| PC | Phosphatidyl Choline |
| PEPC | Phosphoenolpyruvate carboxylase |
| pHc | Increase of the cytosolic pH level |
| PI3P5K | Phosphatidylinositol 3-phosphate 5-kinase |
| PIP2;1 | Plasma membrane intrinsic protein 2;1 (Aquaporin) |
| PLC | Phospholipase C |
| PLDα | Phospholipase D α1 |
| PLDδ | Phospholipase D δ |
| PP2CA | Protein Phosphatase 2CA |
| PtdIns(3,5)P2 | Phosphatidylinositol 3,5-bisphosphate |
| PtdIns(4,5)P2 | Phosphatidylinositol 4,5-bisphosphate |
| PtdInsP3 | Phosphatidylinositol 3-phosphate |
| PtdInsP4 | Phosphatidylinositol 4-phosphate |
| QUAC1 | QUickly activating Anion Channel1 |
| RBOH | NADPH oxidases AtRBOH D and F |
| RCARs | Regulatory Components of ABA Receptor |
| RCN1 | Protein phosphatase 2A |
| ROP10 | Small GTPase ROP10 |
| ROP11 | Small GTPase ROP11 |
| ROS | Reactive oxygen species |
| S1P / PhytoS1P | sphingosine-1-phosphate/ phyto sphingosine-1-phosphate |
| SCAB1 | Stomatal Closure-related Actin Binding protein |
| SLAC1 | Slow Anion Channel- associated 1 |
| SLAH3 | SLAC1 Homologue 3 |
| Sph | Sphingosine |
| SPHK1/2 | Sphingosine Kinases 1 and 2 |
| SPP1 | Sphingoid Phosphate Phosphatase 1 |
| TCTP | Translationally controlled tumor protein |
| V-ATPAse | Vacuolar proton ATPase |
| Vacuolar Acidification | Vacuolar Acidification |
| V-PPase | vacuolar proton pyrophosphatase |
