# Supplementary material for: A new discrete dynamic model of ABA-induced stomatal closure predicts key feedback loops
Source: PLoS Biol. 2017 Sep 22;15(9):e2003451. doi: 10.1371/journal.pbio.2003451 (PMC5627951; doi:10.1371/journal.pbio.2003451)
Supplement: S4 Table — (DOCX) [file pbio.2003451.s005.docx]

**S4 Table.** **Composition of the in-component, strongly connected component, and out-component of the network**

The strongly connected component is a generalized feedback loop, the in-component is the set of nodes that can reach the strongly connected component through paths, and the out-component is the set of nodes that can be reached from the strongly connected component. ABA is in the in-component and Closure is in the out-component. All the paths from ABA to Closure pass through the strongly connected component, and begin in one of two ways: via RCARs, which inhibit the PP2C protein phosphatases, or via a path that includes PI3P5K, PtdIns(3,5)P2 and V-PPase. Four nodes do not meet the criteria for inclusion in any of these three components: PEPC, which is only regulated by ABA, ROP10, which is only regulated by ERA1, and the source nodes CPK6 and CPK23 , which are active at resting Ca^2+^_c_ concentrations [1, 2], unlike other Ca^2+^-activated CPKs.

| **Component** | **Node count** | **Nodes** |
| --- | --- | --- |
| In - component | 27 | ABA, ABH1, ARP complex, DAGK, ERA1, GCR1, GAPC1/2, GEF1/4/10, GTP, MRP5, NAD^+^, NADPH, Nitrite, NtSyp121, RCARs, ROP11, PC, PI3P5K, PtdIns(3,5)P2, PtdIns(4,5)P2, PtdInsP3, PtdInsP4, RCN1, SCAB1, Sph, SPP1, V-PPase |
| Strongly connected component | 36 | 8-nitro-cGMP, ABI1, ABI2, Actin Reorganization, ADPRc, AGB1, AGG3, AtRAC1, Ca^2+^_c_, Ca^2+^ ATPase, cADPR, CaIM, cGMP, CIS, DAG, GPA1, GHR1, HAB1, InsP3, InsP6, NIA1/2, NO, NOGC1, OST1, PA, pH_c_, PLC, PLDα, PLDδ, PP2CA, RBOH, ROS, S1P / PhytoS1P, SPHK1/2, Vacuolar Acidification, V-ATPase |
| Out- component | 18 | AnionEM, Aquaporin (PIP2;1), Closure, CPK3/21, Depolarization, H_2_O Efflux, H^+^ ATPase, K^+^ Efflux, KEV, KOUT, Malate, MPK 9/12, Microtubule Depolymerization, QUAC1, TCTP, SLAH3, SLAC1 |

1. Geiger D, Scherzer S, Mumm P, Marten I, Ache P, Matschi S, et al. Guard cell anion channel SLAC1 is regulated by CDPK protein kinases with distinct Ca2+ affinities. Proc Natl Acad Sci U S A. 2010;107(17):8023-8. Epub 2010/04/14. doi: 10.1073/pnas.0912030107. PubMed PMID: 20385816; PubMed Central PMCID: PMC2867891.

2. Scherzer S, Maierhofer T, Al-Rasheid KA, Geiger D, Hedrich R. Multiple calcium-dependent kinases modulate ABA-activated guard cell anion channels. Mol Plant. 2012;5(6):1409-12. Epub 2012/08/31. doi: 10.1093/mp/sss084. PubMed PMID: 22933711.
