# Supplementary material for: A new discrete dynamic model of ABA-induced stomatal closure predicts key feedback loops
Source: PLoS Biol. 2017 Sep 22;15(9):e2003451. doi: 10.1371/journal.pbio.2003451 (PMC5627951; doi:10.1371/journal.pbio.2003451)
Supplement: S5 Table — (DOCX) [file pbio.2003451.s006.docx]

**S5 Table.** **Distribution of the nodes of the ABA signal transduction network according to three centrality measures: in-degree, out-degree and betweenness centrality.**

The first column specifies the respective centrality values observed in the network. The second column indicates the number of nodes that have the corresponding centrality value, while the third column lists the nodes in each centrality category. The average in- and out-degree is 1.8, thus the average degree is 3.6. Most nodes in the network possess low in-degree, low out-degree and/or low betweenness centrality. Key mediators of ABA-induced stomatal closure (RBOH/ROS, Ca^2+^_c_, pH_c_) exhibit high in- or out-degree and high betweenness centrality. Although the network incorporates the soluble ABA receptor family as the node RCARs, several regulatory effects of ABA, represented as edges pointing to PI3P5K, SphK1/2, PEPC, or AtRAC1, are not currently linked to RCARs.

| **In-Degree** | **Node count** | **Nodes** |
| --- | --- | --- |
| 0 | 23 | ABA, ABH1, ARP Complex, CPK 6, CPK 23, DAGK, ERA1, GAPC1/2, GCR1, GEF 1/4/10, GTP, MRP5, Nitrite, NADPH, NAD^+^, NtSyp121, PC, PtdInsP3, PtdInsP4, RCN1, SCAB1, Sph, SPP1 |
| 1 | 17 | AGG3, Aquaporin(PIP2;1), Ca^2+^ ATPase, InsP6, NIA1/2, NOGC1, PEPC, RCARs, PI3P5K, PtdIns(3,5)P2, PtdIns(4,5)P2, PLC, ROP10, ROP11, TCTP, V-ATPase, V-PPase |
| 2 | 21 | ADPRc, AGB1, AtRAC1, cADPR, cGMP, Closure, CPK 3/21, DAG, GHR1, HAB1, InsP3, KEV, K^+^ Efflux, Microtubule Depolymerization, MPK9/12, QUAC1, PLDα, PP2CA, ROS, SPHK1/2, Vacuolar Acidification |
| 3 | 13 | 8-nitro-cGMP, ABI2, AnionEM, Ca^2+^_c_, CIS, Depolarization, GPA1, H^+^ ATPase, Malate, NO, OST1, PLDδ, S1P / PhytoS1P |
| 4 | 3 | H_2_O Efflux, KOUT, SLAH3 |
| 5 | 4 | ABI1, Actin Reorganization, PA, pH_c_ |
| 7 | 2 | RBOH, CaIM |
| 10 | 1 | SLAC1 |
| **Out-Degree** | **Node count** | **Nodes** |
| 0 | 2 | Closure, ROP10 |
| 1 | 51 | 8-nitro-cGMP, ABH1, Actin Reorganization, ADPRc, AGG3, AnionEM, Aquaporin(PIP2;1), ARP Complex, AtRAC1, Ca^2+^ ATPase, cADPR, CaIM, cGMP, CIS, DAG, DAGK, Depolarization, GAPC1/2, GCR1, GEF 1/4/10, GTP, H^+^ ATPase, H_2_O Efflux, HAB1, InsP6, K^+^ Efflux, KOUT, Malate, MRP5, NAD^+^, NIA1/2, Nitrite, NOGC1, NtSyp121, PC, PEPC, PI3P5K, PLDα, PLDδ, RCN1, PtdIns(3,5)P2, RBOH, SCAB1, SLAC1, SLAH3, Sph, SPHK1/2, SPP1, TCTP, V-ATPase, V-PPase |
| 2 | 17 | AGB1, CPK23, ERA1, GHR1, InsP3, KEV, Microtubule Depolymerization, MPK 9/12, NADPH, PLC, PtdIns(4,5)P2, PtdInsP4, PtdInsP3, PP2CA, ROP11, S1P / PhytoS1P, Vacuolar Acidification |
| 3 | 5 | CPK 6, CPK3/21, GPA1, PA, QUAC1 |
| 4 | 4 | ABA, ABI2, NO, RCARs |
| 5 | 3 | ABI1, OST1, pH_c_ |
| 10 | 1 | ROS |
| 11 | 1 | Ca^2+^_c_ |
| **Betweenness Centrality Interval** | **Node count** | **Nodes** |
| [0, 0.01) | 53 | ABA, AGB1, ABH1, AGG3, AnionEM, Aquaporin (PIP2;1), ARP Complex, Ca^2+^ ATPase, Closure, CPK6, CPK 3/21, CPK 23, DAGK, Depolarization, ERA1, GAPC1/2, GCR1, GEF1/4/10, GTP, HAB1, H^+^ ATPase, H_2_O Efflux, InsP6, K^+^ Efflux, KEV, KOUT, Malate, MAPK 9/12, Microtubule Depolymerization, MRP5, NAD^+^, NADPH, Nitrite, NOGC1, NtSyp121, PEPC, PC, PI3P5K, PP2CA, PtdIns(3,5)P2, PtdIns(4,5)P2, ROP10, ROP11, SCAB1, PtdInsP3, PtdInsP4, RCARs, RCN1, Sph, SLAC1, SLAH3, SPP1, V-ATPase |
| [0.01, 0.02) | 6 | ABI2, cGMP, DAG, InsP3, TCTP, V-PPase |
| [0.02, 0.03) | 6 | AtRAC1, NIA1/2, PLC, PLDα, PLDδ, QUAC1 |
| [0.03, 0.04) | 4 | NO, OST1, SPHK1/2, Vacuolar Acidification |
| [0.04, 0.05) | 4 | 8-nitro-cGMP, Actin Reorganization, GHR1, S1P / PhytoS1P |
| [0.05, 0.06) | 4 | ABI1, ADPRc, cADPR, GPA1 |
| [0.07, 0.08) | 1 | CIS |
| [0.09, 0.1) | 1 | PA |
| [0.1, 0.11) | 1 | pH_c_ |
| [0.14, 0.15) | 1 | CaIM |
| [0.2, 0.21) | 1 | RBOH, ROS |
| [0.23, 0.24) | 1 | Ca^2+^_c_ |
