# Supplementary material for: A new discrete dynamic model of ABA-induced stomatal closure predicts key feedback loops
Source: PLoS Biol. 2017 Sep 22;15(9):e2003451. doi: 10.1371/journal.pbio.2003451 (PMC5627951; doi:10.1371/journal.pbio.2003451)
Supplement: S6 Table — (DOCX) [file pbio.2003451.s007.docx]

**S6 Table. Summary of the long-term dynamics (attractors) of network nodes in the sustained presence or absence of ABA, compared to the assumed initial condition of open stomata.**

Unregulated (source) nodes for which there is evidence of their sufficient abundance are initiated as ON and will stay ON during each simulation. Unregulated (source) nodes for which the evidence points to insufficient abundance are initiated as OFF and will stay OFF. Nodes in these two categories are counted as “+20” and “+2”, respectively, in the long-term dynamics categories, and are not explicitly indicated in the corresponding node lists. Nodes that are known to be sufficiently expressed/active in open stomata are initiated in the ON state. Nodes that are known to be insufficiently expressed or inactive in open stomata are initiated in the OFF state. The remaining 26 nodes are initiated in a randomly selected state. Orange font indicates the nodes whose stabilized state in the presence of ABA is the opposite of their initial state in open stomata, in agreement with experimental observations. The nodes whose stabilized state in the absence of ABA is the same as their initial state are indicated with light green font. The nine nodes in the last row of the table include four self-regulated nodes (CPK3/21, MPK9/12 and Microtubule Depolymerization and Vacuolar Acidification) that can stabilize either ON or OFF. In addition, depolarization, KOUT and K^+^ efflux oscillate if Vacuolar Acidification and KEV are ON (due to the negative feedback loop depolarization→KOUT→K+ efflux--| depolarization) and are OFF if Vacuolar Acidification and KEV are OFF. Finally, SLAH3 can stabilize ON if CPK3/21 is ON.

| **Initial or fixed state** | **Node count** | **Nodes** |
| --- | --- | --- |
| Stays ON (1) | 20 | ABH1, ARP Complex, CPK 6, CPK 23, DAGK, ERA1, GAPC1/2, GCR1, GTP, MRP5, NAD^+^, NADPH, Nitrite, NtSyp121, PC, PtdInsP3, PtdInsP4, RCN1, SCAB1, Sph |
| Stays OFF (0) | 2 | GEF 1/4/10, SPP1 |
| Initiated as ON (1) | 6 | ABI1, ABI2, HAB1, H^+^ ATPase, Malate, PP2CA |
| Initiated as OFF (0) | 26 | Actin Reorganization, Closure, CaIM, Ca^2+^_c_, Ca^2+^ ATPase, CIS, cGMP, CPK 3/21, GPA1, H_2_O Efflux, InsP3, K^+^ Efflux, MAPK9/12, Microtubule Depolymerization, NIA1/2, NO, NOGC1, OST1, PA, pH_c_, PLDα, RBOH, ROS, S1P / PhytoS1P, RCARs, Vacuolar Acidification |
| Initiated randomly | 26 | 8-nitro-cGMP, ADPRc, AnionEM, Aquaporin (PIP2;1), AtRAC1, cADPR, DAG, Depolarization, GHR1, InsP6, KEV, KOUT, PEPC, PI3P5K, PIP2, PLC, PLDδ, PtdIns(35)P2, QUAC1, ROP11, SPHK1/2, SLAC1, SLAH3, TCTP, V-ATPase, V-PPase |
| **Long-term dynamics in the presence of ABA** | **Node count** | **Nodes** |
| Stabilized in the ON state | 39+20 | 8-nitro-cGMP, Actin reorganization, ADPRc, AnionEM, Aquaporin(PIP2;1), cADPR, CaIM, CIS, Closure, cGMP, CPK3/21, Depolarization, GHR1, GPA1, H_2_O Efflux, K^+^ Efflux, KEV, KOUT, MPK 9/12, Microtubule Depolymerization, NIA1/2, NO, NOGC1, OST1, pH_c_, PI3P5K, PA, PLDδ, PtdIns(3,5)P2, PtdIns(4,5)P2, RBOH, RCARs, ROS, S1P/PhytoS1P, SPHK1/2, SLAC1, SLAH3, Vacuolar Acidification, V-PPase |
| Stabilized in the OFF state | 9+2 | ABI1, ABI2, AtRAC1, HAB1, H^+^ ATPase, Malate, PEPC, PP2CA, ROP11 |
| Oscillating | 10 | Ca^2+^_c_, Ca^2+^ ATPase, DAG, TCTP, InsP3, InsP6, PLC, PLDα, QUAC1, V-ATPase |
| **Long-term dynamics in the absence of ABA** | **Node count** | **Nodes** |
| Stabilized in the ON state | 9+20 | ABI2, AtRAC1, GHR1, H^+^ ATPase, HAB1, Malate, PEPC, PIP2, PP2CA |
| Stabilized in the OFF state | 40+2 | 8-nitro-cGMP, ABI1, Actin Reorganization, ADPRc, AnionEM, Aquaporin(PIP2;1), Ca^2+^, cADPR, CaIM, Ca^2+^ ATPase, cGMP, CIS, Closure, DAG, GHR1, GPA1, H_2_O Efflux, InsP3, InsP6, NIA1/2, NO, NOGC1, OST1, QUAC1, RBOH, ROS, pH_c_, PA, PI3P5K, PLC, PLDα, PLDδ, PtdIns(3,5)P2, RCARs, ROP11, SLAC1, S1P / PhytoS1P, TCTP, V-ATPase, V-PPase |
| Stabilized in either the ON or OFF state | 6 | CPK3/21, MPK9/12, KEV, Microtubule Depolymerization, SLAH3, Vacuolar Acidification |
| Stabilized in the OFF state or oscillates | 3 | Depolarization, KOUT, K^+^ Efflux |
