# Supplementary material for: A new discrete dynamic model of ABA-induced stomatal closure predicts key feedback loops
Source: PLoS Biol. 2017 Sep 22;15(9):e2003451. doi: 10.1371/journal.pbio.2003451 (PMC5627951; doi:10.1371/journal.pbio.2003451)
Supplement: S8 Table — (DOCX) [file pbio.2003451.s009.docx]

**S8 Table.** **Full list of the effect of simulated manipulations (KO or CA) in the presence of ABA and comparison with the closest experimental results.**

Green indicates that the model result is consistent with the experimental data, red indicates that the model result is not consistent with experimental data and purple indicates that no comparable experimental data are available. The response categories and notations for the deviations are as in Table 3.

| **Response category** | **Number of cases** | **Identity of the node and its relevant manipulation** | **CPC range** |
| --- | --- | --- | --- |
| Equivalent to wild type | 22 | GTP CA, Sph CA, SCAB1 CA (1b), GEF1/4/10 KO (1a) , CPK6 CA, GAPC CA, Nitrite CA, GCR1 CA, MRP5 CA, RCN1 CA, NAD^+^  CA, PtdInsP3 CA, SPP1 KO (1a), PtdInsP4 CA, PC CA, CPK23 CA, NADPH CA, ARP complex CA, ABH1 CA, NtSyp121 CA, ERA1 CA, DAGK CA | 23.93 – 24.08 |
| Hyper-sensitivity | 48 | Vacuolar Acidification CA, SphK1/2 CA, GPA1 CA, PI3P5K CA, NIA1/2 KO (1b), 8-nitro-cGMP CA, HAB1 KO, RCARs CA, H^+^ ATPase KO, V-ATPase CA, GCR1 KO, PtdIns(3,5)P2 CA, Depolarization CA, TCTP CA, S1P/PhytoS1P CA, Microtubule Depolymerization CA (2), V-PPase CA , ABI1 KO , OST1 CA, ABI2 KO , K^+^ Efflux CA, AtRAC1 KO , SLAH3 CA, ADPRc CA, cADPR CA, InsP3 CA , ERA1 KO, Actin Reorganization CA, InsP6 CA, ABH1 KO, CIS CA, PA CA, CaIM CA, NIA1/2 CA, DAG CA, NO CA, PLDδ CA, PLDα CA, PLC CA , QUAC1 CA, Ca^2+^ ATPase KO, RBOH CA, GHR1 CA, ROS CA , SLAC1 CA , AnionEM CA, H_2_O Efflux CA,  Ca^2+^_c_ CA | 24.08 – 26.7 |
| Close to wild type | 25 | NtSyp121 KO, CPK3/21 CA, MRP5 KO (1b), InsP3 KO (1b), pH_c_ CA (1a), HAB1 CA, cGMP KO (1b), PEPC KO, V-ATPase KO (1b), CPK6 KO, CPK23 KO, NOGC1 CA, Aquaporin(PIP2;1) CA, Malate KO, NO KO (1b), ROP11 KO (1a), KEV CA, cGMP CA, PEPC CA, PtdIns(4,5)P2 CA, Nitrite KO, GTP KO, KOUT CA, PP2CA KO (1a), NOGC1 KO (1b) | 23.92 – 24.07 |
| Hypo-sensitivity | 22 | ROP11 CA, GEF1/4/10 CA, QUAC1 KO, CPK3/21 KO, SLAH3 KO (2), PLC KO, PtdIns(4,5)P2 KO, PtdInsP4 KO, DAG KO, DAGK KO, PLDα KO, V-PPase KO, CIS KO, PtdIns(3,5)P2 KO, cADPR KO, PI3P5K KO, GAPC KO, NAD^+^ KO, ADPRc KO, MPK9/12 CA, InsP6 KO, 8-nitro-cGMP KO | 20.22 – 23.91 |
| Reduced sensitivity | 26 | Vacuolar Acidification KO, pH_c_ KO, ABI2 CA, H^+^ ATPase CA, CaIM KO, GPA1 KO (2), MPK9/12 KO, PP2CA CA, S1P/PhytoS1P KO, SphK1/2 KO, PtdInsP3 KO, GHR1 KO, SLAC1 KO, RCN1 KO, Sph KO, ROS KO, PA KO, RBOH KO, SPP1 CA, NADPH KO, ARP complex KO, AtRAC1 CA, Actin Reorganization KO, SCAB1 KO, PC KO, PLDδ KO | 5.9 – 21.02 |
| Insensitivity | 15 | Malate CA, H_2_O Efflux KO, Microtubule Depolymerization KO, TCTP KO, K^+^ Efflux KO, KEV KO, AnionEM KO, KOUT KO, Aquaporin(PIP2;1) KO, OST1 KO, Depolarization KO, RCARs KO, ABI1 CA, Ca^2+^ ATPase CA, Ca^2+^ _c_ KO | 0.0 – 0.02 |
