# Supplementary material for: A new discrete dynamic model of ABA-induced stomatal closure predicts key feedback loops
Source: PLoS Biol. 2017 Sep 22;15(9):e2003451. doi: 10.1371/journal.pbio.2003451 (PMC5627951; doi:10.1371/journal.pbio.2003451)
Supplement: S9 Table — (DOCX) [file pbio.2003451.s010.docx]

**S9 Table.** **The attractors corresponding to simulated *rboh* knockout, and disrupted K^+^ efflux, respectively, in the presence of ABA.**

As in S6 Table, source nodes, whose fixed ON or OFF states are unaffected by the considered disruptions, are counted as “+20” and “+2”, respectively, in the long term dynamics categories and are not explicitly indicated in the corresponding node lists. Nodes whose stabilized state or behavior is the same as their state in the wild type attractor in the presence of ABA (S6 Table) are shown in green font. The unaffected nodes include several nodes of the strongly connected component (OST1, pH_c_, SPHK, S1P/phytoS1P) and of the out-component.

| **Long-term dynamics in the *rboh* knockout** | **Number of nodes** | **Nodes** |
| --- | --- | --- |
| Stabilized in the ON state | 23+20 | Actin Reorganization, Aquaporin(PIP2;1), CaIM, CPK3/21, Depolarization, GPA1, K^+^ Efflux, KEV, KOUT, MPK 9/12, Microtubule Depolymerization, OST1, pH_c_, PI3P5K, PtdIns(3,5)P2, PtdIns(4,5)P2, RCARs, S1P/PhytoS1P, SPHK1/2, SLAC1, SLAH3, Vacuolar Acidification, V-PPase |
| Stabilized in the OFF state | 20+2 | 8-nitro-cGMP, ABI1, ABI2, ADPRc, AtRAC1, cADPR, cGMP, GHR1, HAB1, H^+^ ATPase, Malate, NIA1/2, NO, NOGC1, PEPC, PLDδ, PP2CA, RBOH, ROP11, ROS |
| Oscillating | 15 | AnionEM, Ca^2+^_c_, Ca^2+^ ATPase, CIS, Closure, DAG, H_2_O Efflux, InsP3, InsP6, PLC, PLDα, PA, QUAC1, TCTP, V-ATPase |
| **Long-term dynamics in case of disruption of K^+^ efflux** | **Number of nodes** | **Nodes** |
| Stabilized in the ON state | 37+20 | 8-nitro-cGMP, ABA, Actin Reorganization, ADPRc, AnionEM, Aquaporin(PIP2;1), cADPR, CaIM, CIS, cGMP, CPK3/21, Depolarization, GHR1, GPA1, KEV, KOUT, MPK 9/12, Microtubule Depolymerization, NIA1/2, NO, NOGC1, OST1, pH_c_, PI3P5K, PA, PLDδ, PtdIns(3,5)P2, PIP2, RBOH, RCARs, ROS, S1P/PhytoS1P, SPHK1/2, SLAC1, SLAH3, Vacuolar Acidification, V-PPase |
| Stabilized in the OFF state | 12+2 | ABI1, ABI2, AtRAC1, Closure, H_2_O Efflux, HAB1, H^+^ ATPase, K^+^ Efflux, Malate, PEPC, PP2CA, ROP11 |
| Oscillating | 10 | Ca^2+^_c_, Ca^2+^ ATPase, DAG, TCTP, InsP3, InsP6, PLC, PLDα, QUAC1, V-ATPase |
