# Supplementary material for: A new discrete dynamic model of ABA-induced stomatal closure predicts key feedback loops
Source: PLoS Biol. 2017 Sep 22;15(9):e2003451. doi: 10.1371/journal.pbio.2003451 (PMC5627951; doi:10.1371/journal.pbio.2003451)
Supplement: S10 Table — (DOCX) [file pbio.2003451.s011.docx]

**S10 Table. Twelve representative cases of consistency between experimentally observed (third column) and simulated (last column) effect of an internal node’s knockout on a second internal node in the presence of ABA.**

In the majority of the cases the knockout of the first node causes the inactivation or disruption of the second node due to the logical structure of the model (i.e. due to the totality of the regulatory functions), irrespective of the initial state of the system or of timing. In a few cases the second node retains the possibility of activation for certain trajectories, and in a single case the second node activates but with a slower timescale than in the wild type system. The model can also be used to predict lack of effect of a node’s knockout. For example, knockout of one of the 18 nodes in the out-component of the network will not affect the nodes of the in-component or of the SCC, as there are no paths that connect back from the out-component to the SCC or to the in-component, thus perturbation cannot propagate backwards. For similar reasons of independence due to lack of paths, *ost1*, *rboh,* or *gpa1* knockout would not affect S1P production, Vacuolar Acidification, Actin Reorganization or malate concentration. Furthermore, the model can be used to distinguish between minor and major effects.

| **First node** | **Type of knockout** | **Experimentally observed effect on the second node** | **Ref.** | **Model result** |
| --- | --- | --- | --- | --- |
| RCARs | Gene knockout | ROS production disrupted | [[1](#_ENREF_1)] | ROS = 0 for the considered initial condition |
| RCARs | Gene knockout | NO production disrupted | [[1](#_ENREF_1)] | ROS = 0 for the considered initial condition |
| OST1 | Gene knockout | ROS production disrupted | [[1-3](#_ENREF_1)] | ROS = 0 |
| OST1 | Gene knockout | NO production disrupted | [[1](#_ENREF_1)] | NO = 0 |
| OST1 | Gene knockout | pH_c_ increase impaired | [[1](#_ENREF_1)] | pH_c_ increase oscillates with an average activity of 0.5 instead of stabilizing at 1 |
| OST1 | Gene knockout | Hyposensitivity of ROS-activated CaIM (CaIM_ROS_) | [[3](#_ENREF_3)] | CaIM_ROS_= 0 |
| GPA1 | Gene knockout | ROS production disrupted | [[4](#_ENREF_4)] | ROS = 0 |
| GPA1 | Gene knockout | Hyposensitivity of CaIM_ROS_ | [[4](#_ENREF_4)] | CaIM_ROS_ = 0 |
| PI3P5K | Pharmacological inhibition | Reduced vacuolar Acidification | [[5](#_ENREF_5)] | Vacuolar Acidification reaches 1 in 17 steps instead of 4 |
| RBOH | Gene knockout | Reduced NO production | [[6](#_ENREF_6)] | NO = 0 |
| RBOH | Gene knockout | Impaired CaIM_ROS_ | [[7](#_ENREF_7)] | CaIM_ROS_ = 0 |
| ABI1 | Dominant mutant | Impaired ROS production | [[8](#_ENREF_8), [9](#_ENREF_9)] | ROS=0 |
| ABI1 | Dominant mutant | Impaired CaIM_ROS_ | [[9](#_ENREF_9)] | CaIM_ROS_ = 0 |
| PtdInsP3 | Pharmacological inhibition | Impaired ROS production | [[10](#_ENREF_10)] | ROS=0 |
| pH_c_ increase | Pharmacological inhibition (pH clamp) | Hyposensitivity of ROS production | [[11](#_ENREF_11)] | ROS=0 |
| PLDα | Gene knockout | Impaired ROS production | [[12](#_ENREF_12)] | ROS reaches 1 in 28 steps instead of 15 |
| PLDα | Gene knockout | Impaired NO production | [[12](#_ENREF_12)] | NO reaches 1 in 29 steps instead of 17 |

1. Yin Y, Adachi Y, Ye W, Hayashi M, Nakamura Y, Kinoshita T, et al. Difference in abscisic acid perception mechanisms between closure induction and opening inhibition of stomata. Plant Physiol. 2013;163(2):600-10. doi: 10.1104/pp.113.223826. PubMed PMID: 23946352; PubMed Central PMCID: PMC3793041.

2. Mustilli AC, Merlot S, Vavasseur A, Fenzi F, Giraudat J. Arabidopsis OST1 protein kinase mediates the regulation of stomatal aperture by abscisic acid and acts upstream of reactive oxygen species production. Plant Cell. 2002;14(12):3089-99. Epub 2002/12/07. PubMed PMID: 12468729; PubMed Central PMCID: PMC151204.

3. Acharya BR, Jeon BW, Zhang W, Assmann SM. Open Stomata 1 (OST1) is limiting in abscisic acid responses of Arabidopsis guard cells. New Phytol. 2013;200(4):1049-63. doi: 10.1111/nph.12469. PubMed PMID: 24033256.

4. Zhang W, Jeon BW, Assmann SM. Heterotrimeric G-protein regulation of ROS signalling and calcium currents in Arabidopsis guard cells. J Exp Bot. 2011;62(7):2371-9. Epub 2011/01/26. doi: 10.1093/jxb/erq424. PubMed PMID: 21262908.

5. Bak G, Lee EJ, Lee Y, Kato M, Segami S, Sze H, et al. Rapid Structural Changes and Acidification of Guard Cell Vacuoles during Stomatal Closure Require Phosphatidylinositol 3,5-Bisphosphate. Plant Cell. 2013. Epub 2013/06/13. doi: 10.1105/tpc.113.110411. PubMed PMID: 23757398.

6. Bright J, Desikan R, Hancock JT, Weir IS, Neill SJ. ABA-induced NO generation and stomatal closure in Arabidopsis are dependent on H2O2 synthesis. Plant J. 2006;45(1):113-22. Epub 2005/12/22. doi:10.1111/j.1365-313X.2005.02615.x. PubMed PMID: 16367958.

7. Kwak JM, Mori IC, Pei ZM, Leonhardt N, Torres MA, Dangl JL, et al. NADPH oxidase AtrbohD and AtrbohF genes function in ROS-dependent ABA signaling in Arabidopsis. EMBO J. 2003;22(11):2623-33. Epub 2003/05/30. doi: 10.1093/emboj/cdg277. PubMed PMID: 12773379; PubMed Central PMCID: PMC156772.

8. Joudoi T, Shichiri Y, Kamizono N, Akaike T, Sawa T, Yoshitake J, et al. Nitrated cyclic GMP modulates guard cell signaling in Arabidopsis. Plant Cell. 2013;25(2):558-71. Epub 2013/02/12. doi: 10.1105/tpc.112.105049. PubMed PMID: 23396828; PubMed Central PMCID: PMC3608778.

9. Murata Y, Pei ZM, Mori IC, Schroeder J. Abscisic acid activation of plasma membrane Ca(2+) channels in guard cells requires cytosolic NAD(P)H and is differentially disrupted upstream and downstream of reactive oxygen species production in abi1-1 and abi2-1 protein phosphatase 2C mutants. Plant Cell. 2001;13(11):2513-23. PubMed PMID: 11701885; PubMed Central PMCID: PMC139468.

10. Park KY, Jung JY, Park J, Hwang JU, Kim YW, Hwang I, et al. A role for phosphatidylinositol 3-phosphate in abscisic acid-induced reactive oxygen species generation in guard cells. Plant Physiol. 2003;132(1):92-8. Epub 2003/05/15. doi: 10.1104/pp.102.016964. PubMed PMID: 12746515; PubMed Central PMCID: PMC166955.

11. Suhita D, Raghavendra AS, Kwak JM, Vavasseur A. Cytoplasmic alkalization precedes reactive oxygen species production during methyl jasmonate- and abscisic acid-induced stomatal closure. Plant Physiol. 2004;134(4):1536-45. doi: 10.1104/pp.103.032250. PubMed PMID: 15064385; PubMed Central PMCID: PMC419829.

12. Zhang Y, Zhu H, Zhang Q, Li M, Yan M, Wang R, et al. Phospholipase dalpha1 and phosphatidic acid regulate NADPH oxidase activity and production of reactive oxygen species in ABA-mediated stomatal closure in Arabidopsis. Plant Cell. 2009;21(8):2357-77. Epub 2009/08/20. doi: 10.1105/tpc.108.062992. PubMed PMID: 19690149; PubMed Central PMCID: PMC2751945.
