# Supplementary material for: A new discrete dynamic model of ABA-induced stomatal closure predicts key feedback loops
Source: PLoS Biol. 2017 Sep 22;15(9):e2003451. doi: 10.1371/journal.pbio.2003451 (PMC5627951; doi:10.1371/journal.pbio.2003451)
Supplement: S11 Table — (DOCX) [file pbio.2003451.s012.docx]

**S11 Table. Full list of the effects of simulated constitutive activity (or external supply) of nodes in the absence of ABA and comparison with the closest experimental results.**

The wild type system in this case refers to an initial condition that represents open stomata in the absence of any closure signal, and initial node states as summarized in S5 Table. Green indicates that the model result is consistent with the experimental data, red indicates that the model result is not consistent with experimental data and purple indicates that no comparable experimental data are available. The response categories and deviation types are as in Table 4.

| **Response category** | **Number of cases** | **Identity of the node that is constitutively active** | **CPC range** |
| --- | --- | --- | --- |
| Equivalent to wild type (signal free) | 20 | ERA1, SCAB1, Nitrite (1a), CPK6, PC, PtdInsP4, NAD^+^, ARP complex, ABH1, CPK23 , GAPC, PtdInsP3, RCN1, NtSyp121, DAGK, GTP, MRP5, Sph, GCR1, NADPH | 0.0 – 0.002 |
| Close to wild type | 47 | Malate, Depolarization, PEPC, S1P/ PhytoS1P (1a), SPP1, MPK9/12, V-PPase, CaIM, PI3P5K, AtRAC1, ADPRc, Ca^2+^ ATPase, PLC, SphK12, HAB1, PLDα, NIA1/2, CPK3/21, V-ATPase, GPA1, pH_c_ (1a), KOUT, SLAC1, PP2CA, QUAC1, ROP11, ABI1, SLAH3, cGMP, ABI2, PtdIns(4,5)P2, Actin Reorganization, NOGC1, InsP6, KEV, NO (1a), cADPR (1a), PtdIns(3,5)P2, GHR1, PA (1a), DAG, 8-nitro-cGMP (1a), Vacuolar Acidification, InsP3 (1a), AnionEM , GEF1/4/10, H^+^ ATPase | 0.0 – 0.0024 |
| Slightly increased response | 7 | TCTP, Microtubule Depolymerization, PLDδ, CIS, Ca^2+^_c_ (1a), K^+^ Efflux, Aquaporin(PIP2;1) | 0.0028 – 0.03 |
| Significantly increased response | 5 | OST1 (2), RCARs (2), H_2_O Efflux, RBOH, ROS | 5.8- 25.2 |
