# Supplementary material for: A new discrete dynamic model of ABA-induced stomatal closure predicts key feedback loops
Source: PLoS Biol. 2017 Sep 22;15(9):e2003451. doi: 10.1371/journal.pbio.2003451 (PMC5627951; doi:10.1371/journal.pbio.2003451)
Supplement: S12 Table — (DOCX) [file pbio.2003451.s013.docx]

**S12 Table.** **Initial inactivity of the PP2C protein phosphatases (HAB1, PP2C, ABI1, ABI2) significantly affects the simulated response to constitutive activity (or external supply) of nodes in the absence of ABA.**

We refer to the time-course of the percentage of closure in the absence of any node interventions as the baseline. Cases of constitutive activity of a source node that is ON in the initial condition (i.e. S6 Table, except with the PP2C protein phosphatases are OFF) are denoted “Equivalent to baseline”. Node activations that lead to a CPC within the equivalent to baseline range (9.6-10.7) are denoted “Close to baseline”. Around 44% of the simulations lead to closure in these two categories of responses. In the cases of dramatically decreased response compared to baseline the percentage of closure stabilizes at or near 0%, while in the single case of decreased response the percentage of closure reaches 22%. In the increased response category the percentage of closure is between 45% and 70%, while in the cases of significantly increased response the percentage of closure is 90% or more. The nodes are in the order of increasing CPC in each category. Nodes that change categories in this table (i.e., assuming initial inactivity of the PP2Cs) compared to Table 4 (i.e., assuming initial activity of the PP2Cs) are indicated by colored font. Nodes whose constitutive activity yields a decreased response compared to Table 4 are indicated with orange font. Nodes whose constitutive activity yields an increased response compared to Table 4 are indicated with green font. There are no wet bench experiments that manipulate the initial state of the PP2Cs, but if one uses the experiments that served as comparison in Table 4, agreement is found in 10 of the 11 cases of discrepancy (all but supply of Nitrite); these nodes are shown in bold. The consistency with experiments of the 13 nodes shown with boldface in Table 4 is maintained, or, in the case of H^+^ ATPase, strengthened.

| **Response category** | **Number of cases in this category** | **Identity of the node that is constitutively activated** | **CPC range** |
| --- | --- | --- | --- |
| Equivalent to baseline | 20 | MRP5, Nitrite, ABH1, DAGK, GTP, NtSyp121, GAPC, RCN1, NAD ^+^, PtdInsP4 , GCR1, Sph, CPK6, PtdInsP3 , ARP complex, SCAB1, NADPH, PC , ERA1, CPK23 | 9.66 – 10.62 |
| Dramatically decreased response | 3 | Malate, Ca^2+^ ATPase, SPP1 | 0.0 – 1.61 |
| Decreased response | 1 | H^+^ ATPase | 5.13 |
| Close to baseline | 24 | NOGC1, **RCARs,** AtRAC1, Microtubule Depolymerization, ABI1, SLAH3, KOUT, Aquaporin(PIP2;1), PtdIns(4,5)P2 , HAB1, MPK9/12, PP2CA, CPK3/21, GEF1/4/10, Depolarization, PEPC,  K^+^ Efflux, ABI2, ROP11, TCTP, cGMP, PtdIns(3,5)P2, **OST1,** V -ATPase | 9.6 – 10.7 |
| Increased response | 11 | KEV, V-PPase, PI3P5K, Vacuolar Acidification, **pH_c_** , SphK1/2, **S1P/phytoS1P**, GPA1, QUAC1, SLAC1, AnionEM | 10.7 – 19 |
| Significantly increased response | 20 | NIA1/2, **8-nitro-cGMP, NO,** ADPRc, Actin Reorganization, InsP6, **InsP3,** **cADPR, PA,** PLDα, H_2_O Efflux, DAG, PLDδ, CaIM, CIS, PLC, RBOH, ROS, GHR1, **Ca^2+^ _c_** | 20.5- 27 |
