# Supplementary material for: A new discrete dynamic model of ABA-induced stomatal closure predicts key feedback loops
Source: PLoS Biol. 2017 Sep 22;15(9):e2003451. doi: 10.1371/journal.pbio.2003451 (PMC5627951; doi:10.1371/journal.pbio.2003451)
Supplement: S14 Table — (DOCX) [file pbio.2003451.s015.docx]

**S14 Table.** **Two alternative scenarios also recapitulate experimental observations of internal drivers of closure.**

ABA is absent in both scenarios. Scenario I involves modifying the initial condition (summarized in S6 Table) to include initial inactivity of ABI1 and ABI2. In scenario II, we assume that Ca^2+^_c_ can inhibit all four PP2C protein phosphatases; the initial condition (with all four PP2Cs ON) is the same as in S6 Table. We refer to the time-course of the percentage of closure in the absence of any node interventions as the baseline and categorize the node activations by their effect compared to this baseline. The baseline of the two scenarios is slightly different, but comparable. Cases of constitutive activity of a source node that is ON in the initial condition are denoted “Equivalent to baseline”. Node activations that lead to a CPC within the equivalent to baseline range (6.1-6.8 in scenario I and 5.6-6.1 in scenario II) are denoted “Close to baseline”. In the equivalent to or close to baseline responses around 26% (for scenario I) or 30% (for scenario II) of the simulations lead to stabilized closure. In the cases of dramatically decreased response the percentage of closure stabilizes at or near 0%, while in the cases of significantly increased response the percentage of closure is 90% or more. The nodes are in the order of increasing CPC according to scenario I; the order in scenario II is very similar. PP2CA is the only node that shifts categories: constitutive activity of PP2CA leads to a decreased response in scenario II and a close to baseline response in scenario I. As is the case in S12 Table, if one uses the experiments that served as comparison in Table 4, both scenario I and scenario II restores agreement in 10 of the 11 cases of discrepancy (all but supply of Nitrite). The consistency with experiments of the 13 nodes shown with boldface in Table 4 is maintained, or, in the case of H^+^ ATPase, strengthened.

| **Response category** | **Identity of the node that is constitutively active** | **CPC range** | |
| --- | --- | --- | --- |
|  |  | **I. ABIs OFF** | **II. Ca^2+^_c_ inhibits PP2Cs** |
| Equivalent to baseline | MRP5, Nitrite, ABH1, DAGK, GTP, NtSyp121, GAPC, RCN1, NAD ^+^, PtdInsP4, GCR1, Sph, CPK6, PtdInsP3 , ARP complex, SCAB1, NADPH, PC , ERA1, CPK23 | 5.6-6.1 | 6.1- 6.8 |
| Dramatically decreased response | Malate, Ca^2+^ ATPase , SPP1, ABI1, ABI2 | 0.0-1.22 | 0.0-0.93 |
| Decreased response | H^+^ ATPase, (PP2CA) | 3.05 | 2.6-3.5 |
| Close to baseline | HAB1, Depolarization, NOGC1, RCARs, AtRAC1, Microtubule Depolymerization, SLAH3, KOUT, Aquaporin(PIP2;1), PtdIns(4,5)P2 , MPK9/12, PK3/21, GEF1/4/10, PEPC, ROP11, TCTP, cGMP, PtdIns(3,5)P2, V –ATPase, KEV, K^+^ Efflux, V-PPase, PI3P5K, Vacuolar Acidification, OST1 | 5.4-6.4 | 5.9-7.1 |
| Increased response | pH_c_ , SphK1/2, S1P/phytoS1P, GPA1, QUAC1, NIA1/2, NO, PA, PLDα, DAG, PLDδ, SLAC1, AnionEM | 8.8-13.3 | 7.5-12.4 |
| Significantly increased response | 8-nitro-cGMP, ADPRc, Actin Reorganization, InsP3, InsP6, cADPR, GHR1, CaIM, CIS, H_2_O Efflux, PLC, RBOH, ROS, Ca^2+^ _c_ | 15.7-26.5 | 16.7-26.5 |
