# Supplementary material for: A new discrete dynamic model of ABA-induced stomatal closure predicts key feedback loops
Source: PLoS Biol. 2017 Sep 22;15(9):e2003451. doi: 10.1371/journal.pbio.2003451 (PMC5627951; doi:10.1371/journal.pbio.2003451)
Supplement: S15 Table — (DOCX) [file pbio.2003451.s016.docx]

**S15 Table.** **The effect of node knockouts and node constitutive activity in the presence of ABA falls into several patterns shared by multiple nodes.**

Dash means the absence of experimental data. Relevant literature citations for the “yes” entries in “Experiment” can be found in Table 3. The model predicts in 41 cases (rows 1-6 and 15-17) that the effect of simulated inactivation of the node is the opposite of the effect of simulated constitutive activity of the node. Seven cases are supported by experimental observation of both interventions (rows 1 and 15). For example, simulated constitutive activity of OST1 leads to hypersensitivity to ABA, while its simulated knockout leads to insensitivity to ABA, both consistent with the results of experiments [1, 2]. In 18 cases one of the interventions is supported and the other has not been studied experimentally (rows 2, 3 and 16), and in nine cases neither intervention was studied experimentally (rows 5 and 17). In the patterns of rows 7-14 and 18-21 (for a total of 41 cases) an opposite effect of the two interventions is contradicted by the model, experiments, or both (i.e. there is at least one “no” entry). Orange font indicates 25 nodes whose stabilized state in the presence of ABA is the opposite of their initial state in open stomata (first identified in S6 Table). For eleven of these the effect of node inactivation is the opposite of the effect of node constitutive activity according to the model and at least one experiment. However, there is at least one “no” entry for 10 nodes that reverse their state during ABA-induced closure. For example, cGMP is produced in response to ABA; its depletion causes a decreased sensitivity to ABA, but external application of cGMP does not cause ABA hypersensitivity [3].

| Entry | Positive regulators of ABA induced closure | | | | |
| --- | --- | --- | --- | --- | --- |
|  | Nodes | Decreased stomatal closure response to ABA in case of the node’s knockout? | | Increased stomatal closure response to ABA in case of the node’s constitutive activity? | |
|  |  | Experiment | Simulation | Experiment | Simulation |
| 1 | Ca^2+^_c_, OST1, PA, PLDα, RCARs, ROS, SPHK1/2, S1P/PhytoS1P | yes | yes | yes | yes |
| 2 | ADPRc, cADPR, CIS, GHR1, K^+^ efflux, KOUT, PI3P5K, PLC, PLDδ, PtdIns(3,5)P2, QUAC1, RBOH, SLAC1, Vacuolar Acidification, V-PPase | yes | yes | - | yes |
| 3 | TCTP | - | yes | yes | yes |
| 4 | Actin Reorganization, AnionEM, CaIM, DAG, H_2_O efflux, InsP6, Depolarization, 8-nitro-cGMP | - | yes | - | yes |
| 5 | Microtubule Depolymerization | yes | yes | no | yes |
| 6 | GPA1, SLAH3 | no | yes | - | yes |
| 7 | Aquaporin (PIP2;1), ARP complex, CPK3/21, GAPC, MPK9/12, PtdInsP3, PtdInsP4, PtdIns(4,5)P2, RCN1 | yes | yes | - | no |
| 8 | pH_c_, SCAB1 | yes | yes | yes | no |
| 9 | DAGK, KEV, NAD^+^_,_ NADPH, PC, Sph | - | yes | - | no |
| 10 | InsP3, NIA1/2, NO | yes | no | - | yes |
| 11 | MRP5, NOGC1,V-ATPase | yes | no | - | no |
| 12 | cGMP | yes | no | no | no |
| 13 | CPK23, GTP, Nitrite, NtSyp121, PEPC | - | no | - | no |
| 14  12 | CPK6 | no | no | - | no |
|  | Negative regulators of ABA induced closure | | | | |
|  | Nodes | Increased response to ABA in case of the node’s knockout? | | Decreased response to ABA in case of the node’s constitutive activity? | |
|  |  | Experiment | Simulation | Experiment | Simulation |
| 15 | ABI1, ABI2 | yes | yes | yes | yes |
| 16 | AtRAC1, H^+^ ATPase | - | yes | yes | yes |
| 17 | Ca^2+^ ATPase | - | yes | - | yes |
| 18 | ABH1, ERA1, GCR1, HAB1 | yes | yes | - | no |
| 19 | GEF1/4/10, PP2CA, ROP11 | yes | no | yes | yes |
| 20 | SPP1 | yes | no | - | yes |
| 21 | malate | - | no | - | yes |

1. Mustilli AC, Merlot S, Vavasseur A, Fenzi F, Giraudat J. Arabidopsis OST1 protein kinase mediates the regulation of stomatal aperture by abscisic acid and acts upstream of reactive oxygen species production. Plant Cell. 2002;14(12):3089-99. Epub 2002/12/07. PubMed PMID: 12468729; PubMed Central PMCID: PMC151204.

2. Acharya BR, Jeon BW, Zhang W, Assmann SM. Open Stomata 1 (OST1) is limiting in abscisic acid responses of Arabidopsis guard cells. New Phytol. 2013;200(4):1049-63. doi: 10.1111/nph.12469. PubMed PMID: 24033256.

3. Joudoi T, Shichiri Y, Kamizono N, Akaike T, Sawa T, Yoshitake J, et al. Nitrated cyclic GMP modulates guard cell signaling in Arabidopsis. Plant Cell. 2013;25(2):558-71. Epub 2013/02/12. doi: 10.1105/tpc.112.105049. PubMed PMID: 23396828; PubMed Central PMCID: PMC3608778.
