# Supplementary material for: A new discrete dynamic model of ABA-induced stomatal closure predicts key feedback loops
Source: PLoS Biol. 2017 Sep 22;15(9):e2003451. doi: 10.1371/journal.pbio.2003451 (PMC5627951; doi:10.1371/journal.pbio.2003451)
Supplement: S1 Text — (DOCX) [file pbio.2003451.s017.docx]

**S1 Text. Complete biological description of the ABA-induced stomatal closure network.**

The ABA signal is perceived by ABA receptors, RCARs/PYR/PYLs [1, 2]. Upon binding to ABA, RCARs interact with and inhibit class A protein phosphatase type 2Cs ABI1, ABI2, HAB1, and PP2CA, which in turn causes activation of OST1 kinase [1-4], a key positive regulator of guard cell ABA signaling. The PP2C protein phosphatases are biochemically active in the absence of ABA and maintain OST1 inhibition via dephosphorylation [2-4]. ABI1 and ABI2 are both positively regulated by ROP11 (a small G protein) [5, 6] which is positively regulated by Guanine Exchange Factors GEF1/4/10 [5, 7]. Upon activation, OST1 activates anion channels SLAC1 [8, 9] and QUAC1 [10], conduits for anion efflux from guard cells [10-14]. OST1 also activates aquaporin PIP2;1 [15], which mediates water efflux from guard cells. OST1 also activates NADPH oxidases RbohD/F which produce ROS [9, 16, 17].

ROS that are generated during ABA signal propagation function as one of the major secondary messengers in GC ABA signaling. The NADPH oxidases RbohD and RbohF (RBOH) are the primary enzymes responsible for ROS production in guard cells [18]. In addition to OST1 [9, 19], GPA1 [20], PA [21], PtdInsP3 [22, 23], pH_c_ increase [24] and RCN1 [25] are required for ROS production and therefore are assumed to be activators of RBOH in the network, whereas ABI1 functions as negative regulator of RBOH [26] as indicated by the respective incoming edges from these nodes to RBOH. The GHR1 receptor-like-kinase (RLK) is activated by ROS (specifically H_2_O_2_), while ABI2 acts as a negative regulator of GHR1 [27]. Reactive oxygen species (ROS) inhibit the protein phosphatases ABI1, ABI2 and HAB1 [28-30] and inhibit the H^+^ ATPase [31].

ROS positively regulate NIA1 and NIA2 (NIA1/2), the enzymes responsible for NO production [32]. Upon accumulation, NO activates NOGC1 [33] which leads to production of cGMP, a substrate for the generation of 8-Nitro-cGMP. The network indicates two positive incoming edges, from ROS and from NO, to 8-Nitro-cGMP, as both ROS and NO are required for generation of 8-Nitro-cGMP [34]. 8-Nitro-cGMP is an activator of ADPRc [34], the enzyme responsible for production of cADPR, a signaling molecule that triggers release of Ca^2+^ from internal stores (CIS) during guard cell ABA signaling [35]. In addition to cADPR, InsP3 and InsP6 play positive regulatory roles in Ca^2+^_c_ increase via promoting release of Ca^2+^ from internal stores as shown by their respective incoming positive edges to CIS [35-37].

In response to ABA, Ca^2+^_c_ levels also increase in guard cells by influx of Ca^2+^ across the plasma membrane (CaIM). Influx of Ca^2+^ (CaIM) in response to ABA in guard cells is positively regulated by actin reorganization [38, 39], NtSyp121 [40], GHR1 [27], and MRP5 [41], and is negatively regulated by ABH1 [42] and ERA1 [43]; all of which are implemented as direct incoming edges to CaIM. The actin reorganization that contributes to CaIM is in turn regulated by other processes. The ARP complex is involved in actin nucleation [44, 45] and STOMATAL CLOSURE-RELATED ACTIN BINDING PROTEIN1 (SCAB1) stabilizes actin filaments [46]. The network includes incoming edges from ARP complex and SCAB1 to the actin reorganization node as both are involved in the actin reorganization processes [44-46]. PtdInsP4 and PtdInsP3 are both implicated as positive regulators of ABA-induced actin reorganization, as pharmacological inhibition of biosynthesis of these two phosphoinositides negatively affects actin reorganization [47]. The network incorporates the most parsimonious explanation of this observation as positive edges from PtdInsP3 and PtdInsP4 to actin reorganization. The incoming negative edge to the actin reorganization node from AtRAC1, a small G-protein, reflects the experimental observations that expression of a dominant-positive AtRAC1 mutant inhibits ABA-induced actin reorganization whereas expression of a dominant-negative AtRAC1 mutant promotes actin reorganization in the absence of ABA [48]. Only active AtRAC1 can bind to P-21activated kinase (PAK). By employing GST-PAK70–106 pulldown assays it has been reported that ABA inactivates AtRAC1 [49], whereas ABI1 has been implicated as a positive regulator of AtRAC1 [48], both of which are incorporated in the network as green (indirect) edges.

Increase of Ca^2+^_c_ activates the calcium-dependent protein kinases CPK3 and CPK21 [50, 51], whose activity is then sustained by autophosphorylation[52]. Ca^2+^ binding also promotes interaction between TCTP (a calcium and tubulin binding protein) and microtubules, which leads to microtubule depolymerization, an essential process for ABA-induced stomatal closure, as indicated by the Ca^2+^_c_ →TCTP→ Microtubule depolymerization→ closure path [53]. Guard cells of the *mpk9 mpk12* double mutant do not show activation of slow anion channels in response to Ca^2+^, which suggests that both MPK9 and MPK12 participate in calcium activation of slow anion channel in guard cells [54], parsimoniously represented as the path Ca^2+^_c_ →MPK9/12 →SLAC1. Conversely, an increase in Ca^2+^_c_ inhibits the H^+^ ATPase [55]. Increase of cytosolic Ca^2+^ also corresponds to an increase in substrate availability and thus activation of the Ca^2+^ ATPase [56] that in turn pumps Ca^2+^ from cytosol to the apoplast which reduces the level of cytosolic Ca^2+^, contributing to oscillations in Ca^2+^_c_ [56].

Several lipid metabolites, produced by a variety of enzymatic pathways, play signaling roles during ABA-induced stomatal closure. Phosphatidic acid, PA, is produced in guard cells in response to ABA [57]. PA binds to ABI1 and inhibits its enzyme activity, while PA binding to RBOH activates its enzyme activity [21, 57, 58]. Both PLDα1 (node PLDα) and PLDδ produce PA [21, 58, 59]. PA can also be produced by DAGK-mediated phosphorylation of DAG, a product of PLC [60, 61]. Active GPA1 plays an essential role for PLDα1 to remain in the active state as indicated by a positive edge from GPA1 to PLDα [62, 63]. Ca^2+^_c_ is required for activation of PLDα1 and also promotes translocation of PLDα1 to the plasma membrane and tonoplast where lipid substrates are available for the enzyme [64]. In addition, ROS promotes interaction of GAPC1/2 with PLDδ, which in turn activates PLDδ [65]; this is represented as separate edges from ROS and GAPC1/2 to PLDδ. PLDδ can also be activated by NO [61]. PLC requires Ca^2+^ for its activity [66]. InsP_3_ and DAG are produced by PLCs using PIP2 as substrate; PIP2 in turn is produced from its precursor PtdInsP4 [67]. InsP_6_ can be produced from InsP_3_ [68]. All of these are implemented in the network as direct substrate and product relationships.

ABA stimulates sphingosine kinase activity [69], presumably by the sphingosine kinases, SPHK1 and SPHK2 (SPHK1/2), leading to S1P production. PA also activates both SPHKs by physical interaction [70]. The network indicates a positive edge from S1P to the G protein α subunit GPA1, as it has been shown that S1P promotion of stomatal closure is impaired in *gpa1* null mutants [71]. GCR1 has been implicated by mutant analysis as a negative regulator of GPA1, and so GCR1 is shown as an inhibitory edge of GPA1 [72]. SPP1, a long-chain base 1-phosphatase (LCBP), is a negative regulator of S1P accumulation in plants [73].

ABA promotes an increase in cytosolic pH in guard cells [24, 74]. OST1 functions as a positive regulator of cytosolic alkalization in guard cells whereas both ABI1 and ABI2 function as negative regulators [75]. Ca^2+^_c_ also functions as a positive regulator of pH_c_ increase in guard cells [75]. Increase of cytosolic pH in response to ABA is one of the positive regulators of SLAC1 [76] and also positively regulates ABI1 [77]. Increase of pH_c_ is presumed to negatively regulate the H^+^ ATPase, owing to the decreased concentration of H^+^, the transported ion, and based on observed lower rates of ATP hydrolysis at alkaline pH [78]. ABA-induced vacuolar acidification is required for maintenance of ABA-induced cytosolic alkalization as shown by a positive edge from the former to the latter node in the network [79]. In addition to ABA-induced cytosolic pH increase, ABA-induced vacuolar acidification is required for K^+^ efflux from the vacuole (KEV) and so is depicted as a positive edge into KEV [79]. PI3P5K is indirectly activated by ABA [79] and is responsible for production of PtdIns(3,5)P2 [79]. PtdIns (3,5)P2 activates a V-PPase that in turn promotes vacuolar acidification [79]. Ca^2+^_c_ has been implicated as a positive regulator of the V-ATPase, a proton pump that also mediates vacuolar acidification [80]. Both the V-PPase and V-ATPase contribute to vacuolar acidification and are shown as independent positive edges [79].

Anion efflux across the plasma membrane (Anion EM) is primarily regulated by two slow anion channels, SLAC1 and SLAH3, and by a rapid anion channel (QUAC1) [13] which is implemented as three positive edges from these three anion channels to the Anion EM node. These anion channels are regulated by multiple signaling proteins and second messengers. OST1 [8, 9], GHR1 [27], and multiple CPKs [50, 51, 81] activate SLAC1 by phosphorylation. Guard cells of the *mpk9 mpk12* double mutant do not show activation of slow anion channels in response to ABA and Ca^2+^, indicating that MPK9 and MPK12 are also positive regulators of SLAC1, although no physical interaction between MPK9/12 and slow anion channels has been reported [54]. A positive edge from cytosolic pH (pH_c_) to SLAC1 indicates the former node is also a positive regulator of slow anion channels [76]. In contrast, the phosphatases ABI1, ABI2 and PP2CA are negative regulators of SLAC1 activation mediated by the kinases OST1, CPK23, CPK21 and CPK6 [8, 50, 81, 82]. CPK6, CPK23, CPK3, and CPK 21 activate SLAH3 by physical interaction [51, 83, 84] and so we presume that SLAH3 functions in anion efflux during stomatal closure, although the single SLAH3 knockout has no phenotype in ABA-induced stomatal closure [83], perhaps because of functional redundancy with SLAC1. ABI1 has been implicated as a negative regulator of SLAH3 [83]. Upon physical interaction, OST1 activates QUAC1 [10]. Calcium also has been implicated as an upstream positive regulator of QUAC1 [11]. PEPC is an enzyme of malate biosynthesis. A negative edge from malate to H_2_O efflux in the network reflects that accumulation of malate negatively affects ABA-induced stomatal closure due to its osmotic effect on water retention. ABA indirectly inhibits PEPC activity in guard cells [85] and promotes reduction of malate content by disposal via release, gluconeogenesis or consumption in the TCA cycle [86]. Malate levels in guard cells also decrease due to efflux from the cytosol to the apoplast, a component of AnionEM [10, 13].

Anion efflux from the cytosol causes membrane depolarization that in turn drives K^+^ efflux from cytosol to the apoplast which is dependent on K^+^ efflux from the vacuole (KEV) and is mediated by outwardly rectifying K^+^ channels (KOUT) that are activated by depolarization [87]. Both Ca^2+^ and vacuolar acidification promote K^+^ efflux from the vacuole to cytosol [87]). An incoming positive edge from the node Depolarization to the KOUT channels node in the network indicates the essential role of membrane depolarization in activation of the voltage-gated KOUT channels that mediate K^+^ efflux [88]. The activity of the KOUT channels is also promoted by cytosolic pH increase [89] and inhibited by ROS [90] and NO [91], as shown by respective positive and negative edges.

Aquaporins, specifically Plasma Membrane Intrinsic Protein 2;1 (PIP2;1) are connected with the H_2_O efflux node in the Network as the former node mediates H_2_O efflux from the guard cell . OST1 activates PIP2;1 by phosphorylation [15]). Loss of organic and inorganic anions and K^+^ leads to osmotically-driven efflux of H_2_O via aquaporins (e.g. PIP2;1), which in turn leads to stomatal closure by guard cell deflation. The network contains a positive edge from the microtubule depolymerization node to the closure node, since stabilization of microtubules inhibits ABA-induced stomatal closer [92].

References

1. Ma Y, Szostkiewicz I, Korte A, Moes D, Yang Y, Christmann A, et al. Regulators of PP2C phosphatase activity function as abscisic acid sensors. Science. 2009;324(5930):1064-8. Epub 2009/05/02. doi: 10.1126/science.1172408. PubMed PMID: 19407143.

2. Park SY, Fung P, Nishimura N, Jensen DR, Fujii H, Zhao Y, et al. Abscisic acid inhibits type 2C protein phosphatases via the PYR/PYL family of START proteins. Science. 2009;324(5930):1068-71. Epub 2009/05/02. doi: 10.1126/science.1173041. PubMed PMID: 19407142; PubMed Central PMCID: PMC2827199.

3. Nishimura N, Sarkeshik A, Nito K, Park SY, Wang A, Carvalho PC, et al. PYR/PYL/RCAR family members are major in-vivo ABI1 protein phosphatase 2C-interacting proteins in Arabidopsis. Plant J. 2010;61(2):290-9. Epub 2009/10/31. doi: 10.1111/j.1365-313X.2009.04054.x. PubMed PMID: 19874541; PubMed Central PMCID: PMC2807913.

4. Umezawa T, Sugiyama N, Mizoguchi M, Hayashi S, Myouga F, Yamaguchi-Shinozaki K, et al. Type 2C protein phosphatases directly regulate abscisic acid-activated protein kinases in Arabidopsis. Proc Natl Acad Sci U S A. 2009;106(41):17588-93. doi: 10.1073/pnas.0907095106. PubMed PMID: 19805022; PubMed Central PMCID: PMC2754379.

5. Yu F, Qian L, Nibau C, Duan Q, Kita D, Levasseur K, et al. FERONIA receptor kinase pathway suppresses abscisic acid signaling in Arabidopsis by activating ABI2 phosphatase. Proc Natl Acad Sci U S A. 2012;109(36):14693-8. Epub 2012/08/22. doi: 10.1073/pnas.1212547109. PubMed PMID: 22908257; PubMed Central PMCID: PMC3437822.

6. Li Z, Gao X, Chinnusamy V, Bressan R, Wang ZX, Zhu JK, et al. ROP11 GTPase negatively regulates ABA signaling by protecting ABI1 phosphatase activity from inhibition by the ABA receptor RCAR1/PYL9 in Arabidopsis. J Integr Plant Biol. 2012;54(3):180-8. Epub 2012/01/19. doi: 10.1111/j.1744-7909.2012.01101.x. PubMed PMID: 22251383; PubMed Central PMCID: PMC3586988.

7. Li Z, Kang J, Sui N, Liu D. ROP11 GTPase is a negative regulator of multiple ABA responses in Arabidopsis. J Integr Plant Biol. 2012;54(3):169-79. Epub 2012/01/12. doi: 10.1111/j.1744-7909.2012.01100.x. PubMed PMID: 22233300.

8. Geiger D, Scherzer S, Mumm P, Stange A, Marten I, Bauer H, et al. Activity of guard cell anion channel SLAC1 is controlled by drought-stress signaling kinase-phosphatase pair. Proc Natl Acad Sci U S A. 2009;106(50):21425-30. Epub 2009/12/04. doi: 10.1073/pnas.0912021106. PubMed PMID: 19955405; PubMed Central PMCID: PMC2795561.

9. Acharya BR, Jeon BW, Zhang W, Assmann SM. Open Stomata 1 (OST1) is limiting in abscisic acid responses of Arabidopsis guard cells. New Phytol. 2013;200(4):1049-63. Epub 2013/09/17. doi: 10.1111/nph.12469. PubMed PMID: 24033256.

10. Imes D, Mumm P, Bohm J, Al-Rasheid KA, Marten I, Geiger D, et al. Open stomata 1 (OST1) kinase controls R-type anion channel QUAC1 in Arabidopsis guard cells. Plant J. 2013;74(3):372-82. Epub 2013/03/05. doi: 10.1111/tpj.12133. PubMed PMID: 23452338.

11. Sasaki T, Mori IC, Furuichi T, Munemasa S, Toyooka K, Matsuoka K, et al. Closing plant stomata requires a homolog of an aluminum-activated malate transporter. Plant Cell Physiol. 2010;51(3):354-65. Epub 2010/02/16. doi: 10.1093/pcp/pcq016. PubMed PMID: 20154005; PubMed Central PMCID: PMC2835873.

12. Negi J, Matsuda O, Nagasawa T, Oba Y, Takahashi H, Kawai-Yamada M, et al. CO2 regulator SLAC1 and its homologues are essential for anion homeostasis in plant cells. Nature. 2008;452(7186):483-6. doi: 10.1038/nature06720. PubMed PMID: 18305482.

13. Hedrich R. Ion channels in plants. Physiol Rev. 2012;92(4):1777-811. Epub 2012/10/18. doi: 10.1152/physrev.00038.2011. PubMed PMID: 23073631.

14. Vahisalu T, Kollist H, Wang YF, Nishimura N, Chan WY, Valerio G, et al. SLAC1 is required for plant guard cell S-type anion channel function in stomatal signalling. Nature. 2008;452(7186):487-91. Epub 2008/02/29. doi: 10.1038/nature06608. PubMed PMID: 18305484; PubMed Central PMCID: PMC2858982.

15. Grondin A, Rodrigues O, Verdoucq L, Merlot S, Leonhardt N, Maurel C. Aquaporins Contribute to ABA-Triggered Stomatal Closure through OST1-Mediated Phosphorylation. Plant Cell. 2015;27(7):1945-54. Epub 2015/07/15. doi: 10.1105/tpc.15.00421. PubMed PMID: 26163575; PubMed Central PMCID: PMC4531361.

16. Kwak JM, Moon JH, Murata Y, Kuchitsu K, Leonhardt N, DeLong A, et al. Disruption of a guard cell-expressed protein phosphatase 2A regulatory subunit, RCN1, confers abscisic acid insensitivity in Arabidopsis. Plant Cell. 2002;14(11):2849-61. Epub 2002/11/06. PubMed PMID: 12417706; PubMed Central PMCID: PMC152732.

17. Mustilli AC, Merlot S, Vavasseur A, Fenzi F, Giraudat J. Arabidopsis OST1 protein kinase mediates the regulation of stomatal aperture by abscisic acid and acts upstream of reactive oxygen species production. Plant Cell. 2002;14(12):3089-99. Epub 2002/12/07. PubMed PMID: 12468729; PubMed Central PMCID: PMC151204.

18. Kwak JM, Mori IC, Pei ZM, Leonhardt N, Torres MA, Dangl JL, et al. NADPH oxidase AtrbohD and AtrbohF genes function in ROS-dependent ABA signaling in Arabidopsis. EMBO J. 2003;22(11):2623-33. Epub 2003/05/30. doi: 10.1093/emboj/cdg277. PubMed PMID: 12773379; PubMed Central PMCID: PMC156772.

19. Sirichandra C, Gu D, Hu HC, Davanture M, Lee S, Djaoui M, et al. Phosphorylation of the Arabidopsis AtrbohF NADPH oxidase by OST1 protein kinase. FEBS Lett. 2009;583(18):2982-6. Epub 2009/09/01. doi: 10.1016/j.febslet.2009.08.033. PubMed PMID: 19716822.

20. Zhang W, Jeon BW, Assmann SM. Heterotrimeric G-protein regulation of ROS signalling and calcium currents in Arabidopsis guard cells. J Exp Bot. 2011;62(7):2371-9. Epub 2011/01/26. doi: 10.1093/jxb/erq424. PubMed PMID: 21262908.

21. Zhang Y, Zhu H, Zhang Q, Li M, Yan M, Wang R, et al. Phospholipase dalpha1 and phosphatidic acid regulate NADPH oxidase activity and production of reactive oxygen species in ABA-mediated stomatal closure in Arabidopsis. Plant Cell. 2009;21(8):2357-77. Epub 2009/08/20. doi: 10.1105/tpc.108.062992. PubMed PMID: 19690149; PubMed Central PMCID: PMC2751945.

22. Park KY, Jung JY, Park J, Hwang JU, Kim YW, Hwang I, et al. A role for phosphatidylinositol 3-phosphate in abscisic acid-induced reactive oxygen species generation in guard cells. Plant Physiol. 2003;132(1):92-8. Epub 2003/05/15. doi: 10.1104/pp.102.016964. PubMed PMID: 12746515; PubMed Central PMCID: PMC166955.

23. Ellson CD, Gobert-Gosse S, Anderson KE, Davidson K, Erdjument-Bromage H, Tempst P, et al. PtdIns(3)P regulates the neutrophil oxidase complex by binding to the PX domain of p40(phox). Nat Cell Biol. 2001;3(7):679-82. Epub 2001/07/04. doi: 10.1038/35083076. PubMed PMID: 11433301.

24. Suhita D, Raghavendra AS, Kwak JM, Vavasseur A. Cytoplasmic alkalization precedes reactive oxygen species production during methyl jasmonate- and abscisic acid-induced stomatal closure. Plant Physiol. 2004;134(4):1536-45. doi: 10.1104/pp.103.032250. PubMed PMID: 15064385; PubMed Central PMCID: PMC419829.

25. Saito N, Munemasa S, Nakamura Y, Shimoishi Y, Mori IC, Murata Y. Roles of RCN1, regulatory A subunit of protein phosphatase 2A, in methyl jasmonate signaling and signal crosstalk between methyl jasmonate and abscisic acid. Plant Cell Physiol. 2008;49(9):1396-401. Epub 2008/07/25. doi: 10.1093/pcp/pcn106. PubMed PMID: 18650210.

26. Murata Y, Pei ZM, Mori IC, Schroeder J. Abscisic acid activation of plasma membrane Ca(2+) channels in guard cells requires cytosolic NAD(P)H and is differentially disrupted upstream and downstream of reactive oxygen species production in abi1-1 and abi2-1 protein phosphatase 2C mutants. Plant Cell. 2001;13(11):2513-23. PubMed PMID: 11701885; PubMed Central PMCID: PMC139468.

27. Hua D, Wang C, He J, Liao H, Duan Y, Zhu Z, et al. A plasma membrane receptor kinase, GHR1, mediates abscisic acid- and hydrogen peroxide-regulated stomatal movement in Arabidopsis. Plant Cell. 2012;24(6):2546-61. Epub 2012/06/26. doi: 10.1105/tpc.112.100107. PubMed PMID: 22730405; PubMed Central PMCID: PMC3406912.

28. Sridharamurthy M, Kovach A, Zhao Y, Zhu JK, Xu HE, Swaminathan K, et al. H2O2 inhibits ABA-signaling protein phosphatase HAB1. PLoS One. 2014;9(12):e113643. doi: 10.1371/journal.pone.0113643. PubMed PMID: 25460914; PubMed Central PMCID: PMC4252038.

29. Meinhard M, Grill E. Hydrogen peroxide is a regulator of ABI1, a protein phosphatase 2C from Arabidopsis. FEBS Lett. 2001;508(3):443-6. PubMed PMID: 11728469.

30. Meinhard M, Rodriguez PL, Grill E. The sensitivity of ABI2 to hydrogen peroxide links the abscisic acid-response regulator to redox signalling. Planta. 2002;214(5):775-82. doi: 10.1007/s00425-001-0675-3. PubMed PMID: 11882947.

31. Zhang X, Wang H, Takemiya A, Song CP, Kinoshita T, Shimazaki K. Inhibition of blue light-dependent H+ pumping by abscisic acid through hydrogen peroxide-induced dephosphorylation of the plasma membrane H+-ATPase in guard cell protoplasts. Plant Physiol. 2004;136(4):4150-8. doi: 10.1104/pp.104.046573. PubMed PMID: 15563626; PubMed Central PMCID: PMC535845.

32. Bright J, Desikan R, Hancock JT, Weir IS, Neill SJ. ABA-induced NO generation and stomatal closure in Arabidopsis are dependent on H2O2 synthesis. Plant J. 2006;45(1):113-22. Epub 2005/12/22. doi: 10.1111/j.1365-313X.2005.02615.x. PubMed PMID: 16367958.

33. Mulaudzi T, Ludidi N, Ruzvidzo O, Morse M, Hendricks N, Iwuoha E, et al. Identification of a novel Arabidopsis thaliana nitric oxide-binding molecule with guanylate cyclase activity in vitro. FEBS Lett. 2011;585(17):2693-7. Epub 2011/08/09. doi: 10.1016/j.febslet.2011.07.023. PubMed PMID: 21820435.

34. Joudoi T, Shichiri Y, Kamizono N, Akaike T, Sawa T, Yoshitake J, et al. Nitrated cyclic GMP modulates guard cell signaling in Arabidopsis. Plant Cell. 2013;25(2):558-71. Epub 2013/02/12. doi: 10.1105/tpc.112.105049. PubMed PMID: 23396828; PubMed Central PMCID: PMCPMC3608778.

35. Guse AH. Cyclic ADP-ribose: a novel Ca2+-mobilising second messenger. Cell Signal. 1999;11(5):309-16. Epub 1999/06/22. PubMed PMID: 10376802.

36. Staxen I, Pical C, Montgomery LT, Gray JE, Hetherington AM, McAinsh MR. Abscisic acid induces oscillations in guard-cell cytosolic free calcium that involve phosphoinositide-specific phospholipase C. Proc Natl Acad Sci U S A. 1999;96(4):1779-84. PubMed PMID: 9990101; PubMed Central PMCID: PMC15593.

37. Lemtiri-Chlieh F, MacRobbie EA, Webb AA, Manison NF, Brownlee C, Skepper JN, et al. Inositol hexakisphosphate mobilizes an endomembrane store of calcium in guard cells. Proc Natl Acad Sci U S A. 2003;100(17):10091-5. Epub 2003/08/13. doi: 10.1073/pnas.1133289100. PubMed PMID: 12913129; PubMed Central PMCID: PMC187775.

38. Hwang JU, Lee Y. Abscisic acid-induced actin reorganization in guard cells of dayflower is mediated by cytosolic calcium levels and by protein kinase and protein phosphatase activities. Plant Physiol. 2001;125(4):2120-8. Epub 2001/04/12. PubMed PMID: 11299391; PubMed Central PMCID: PMC88867.

39. Zhao SS, Jiang YX, Zhao Y, Huang SJ, Yuan M, Zhao YX, et al. CASEIN KINASE1-LIKE PROTEIN2 Regulates Actin Filament Stability and Stomatal Closure via Phosphorylation of Actin Depolymerizing Factor. Plant Cell. 2016;28(6):1422-39. PubMed PMID: WOS:000380689400018.

40. Sokolovski S, Hills A, Gay RA, Blatt MR. Functional interaction of the SNARE protein NtSyp121 in Ca2+ channel gating, Ca2+ transients and ABA signalling of stomatal guard cells. Mol Plant. 2008;1(2):347-58. Epub 2008/03/01. doi: 10.1093/mp/ssm029. PubMed PMID: 19825544.

41. Suh SJ, Wang YF, Frelet A, Leonhardt N, Klein M, Forestier C, et al. The ATP binding cassette transporter AtMRP5 modulates anion and calcium channel activities in Arabidopsis guard cells. J Biol Chem. 2007;282(3):1916-24. Epub 2006/11/14. doi: 10.1074/jbc.M607926200. PubMed PMID: 17098742.

42. Hugouvieux V, Kwak JM, Schroeder JI. An mRNA cap binding protein, ABH1, modulates early abscisic acid signal transduction in Arabidopsis. Cell. 2001;106(4):477-87. Epub 2001/08/30. PubMed PMID: 11525733.

43. Allen GJ, Murata Y, Chu SP, Nafisi M, Schroeder JI. Hypersensitivity of abscisic acid-induced cytosolic calcium increases in the Arabidopsis farnesyltransferase mutant era1-2. Plant Cell. 2002;14(7):1649-62. Epub 2002/07/18. PubMed PMID: 12119381; PubMed Central PMCID: PMC150713.

44. Jiang K, Sorefan K, Deeks MJ, Bevan MW, Hussey PJ, Hetherington AM. The ARP2/3 complex mediates guard cell actin reorganization and stomatal movement in Arabidopsis. Plant Cell. 2012;24(5):2031-40. Epub 2012/05/10. doi: 10.1105/tpc.112.096263. PubMed PMID: 22570440; PubMed Central PMCID: PMCPMC3442585.

45. Daugherty KM, Goode BL. Functional surfaces on the p35/ARPC2 subunit of Arp2/3 complex required for cell growth, actin nucleation, and endocytosis. J Biol Chem. 2008;283(24):16950-9. Epub 2008/04/03. doi: 10.1074/jbc.M800783200. PubMed PMID: 18381280; PubMed Central PMCID: PMCPMC2423265.

46. Zhao Y, Zhao S, Mao T, Qu X, Cao W, Zhang L, et al. The plant-specific actin binding protein SCAB1 stabilizes actin filaments and regulates stomatal movement in Arabidopsis. Plant Cell. 2011;23(6):2314-30. Epub 2011/07/02. doi: 10.1105/tpc.111.086546. PubMed PMID: 21719691; PubMed Central PMCID: PMC3160031.

47. Choi Y, Lee Y, Jeon BW, Staiger CJ, Lee Y. Phosphatidylinositol 3- and 4-phosphate modulate actin filament reorganization in guard cells of day flower. Plant Cell Environ. 2008;31(3):366-77. Epub 2007/12/20. doi: 10.1111/j.1365-3040.2007.01769.x. PubMed PMID: 18088331.

48. Lemichez E, Wu Y, Sanchez JP, Mettouchi A, Mathur J, Chua NH. Inactivation of AtRac1 by abscisic acid is essential for stomatal closure. Genes Dev. 2001;15(14):1808-16. Epub 2001/07/19. doi: 10.1101/gad.900401. PubMed PMID: 11459830; PubMed Central PMCID: PMC312738.

49. Manser E, Loo TH, Koh CG, Zhao ZS, Chen XQ, Tan L, et al. PAK kinases are directly coupled to the PIX family of nucleotide exchange factors. Mol Cell. 1998;1(2):183-92. PubMed PMID: 9659915.

50. Geiger D, Scherzer S, Mumm P, Marten I, Ache P, Matschi S, et al. Guard cell anion channel SLAC1 is regulated by CDPK protein kinases with distinct Ca2+ affinities. Proc Natl Acad Sci U S A. 2010;107(17):8023-8. Epub 2010/04/14. doi: 10.1073/pnas.0912030107. PubMed PMID: 20385816; PubMed Central PMCID: PMC2867891.

51. Scherzer S, Maierhofer T, Al-Rasheid KA, Geiger D, Hedrich R. Multiple calcium-dependent kinases modulate ABA-activated guard cell anion channels. Mol Plant. 2012;5(6):1409-12. Epub 2012/08/31. doi: 10.1093/mp/sss084. PubMed PMID: 22933711.

52. Swatek KN, Wilson RS, Ahsan N, Tritz RL, Thelen JJ. Multisite phosphorylation of 14-3-3 proteins by calcium-dependent protein kinases. Biochem J. 2014;459(1):15-25. Epub 2014/01/21. doi: 10.1042/BJ20130035. PubMed PMID: 24438037; PubMed Central PMCID: PMC4127189.

53. Kim YM, Han YJ, Hwang OJ, Lee SS, Shin AY, Kim SY, et al. Overexpression of Arabidopsis translationally controlled tumor protein gene AtTCTP enhances drought tolerance with rapid ABA-induced stomatal closure. Mol Cells. 2012;33(6):617-26. Epub 2012/05/23. doi: 10.1007/s10059-012-0080-8. PubMed PMID: 22610367; PubMed Central PMCID: PMC3887759.

54. Jammes F, Song C, Shin D, Munemasa S, Takeda K, Gu D, et al. MAP kinases MPK9 and MPK12 are preferentially expressed in guard cells and positively regulate ROS-mediated ABA signaling. Proc Natl Acad Sci U S A. 2009;106(48):20520-5. Epub 2009/11/17. doi: 10.1073/pnas.0907205106. PubMed PMID: 19910530; PubMed Central PMCID: PMC2776606.

55. Kinoshita T, Nishimura M, Shimazaki K. Cytosolic Concentration of Ca2+ Regulates the Plasma Membrane H+-ATPase in Guard Cells of Fava Bean. Plant Cell. 1995;7(8):1333-42. doi: 10.1105/tpc.7.8.1333. PubMed PMID: 12242406; PubMed Central PMCID: PMC160955.

56. Sanders D, Pelloux J, Brownlee C, Harper JF. Calcium at the crossroads of signaling. Plant Cell. 2002;14 Suppl:S401-17. PubMed PMID: 12045291; PubMed Central PMCID: PMC151269.

57. Jacob T, Ritchie S, Assmann SM, Gilroy S. Abscisic acid signal transduction in guard cells is mediated by phospholipase D activity. Proc Natl Acad Sci U S A. 1999;96(21):12192-7. Epub 1999/10/16. PubMed PMID: 10518598; PubMed Central PMCID: PMC18434.

58. Zhang W, Qin C, Zhao J, Wang X. Phospholipase D alpha 1-derived phosphatidic acid interacts with ABI1 phosphatase 2C and regulates abscisic acid signaling. Proc Natl Acad Sci U S A. 2004;101(25):9508-13. Epub 2004/06/16. doi: 10.1073/pnas.0402112101. PubMed PMID: 15197253; PubMed Central PMCID: PMC439007.

59. Uraji M, Katagiri T, Okuma E, Ye W, Hossain MA, Masuda C, et al. Cooperative function of PLDdelta and PLDalpha1 in abscisic acid-induced stomatal closure in Arabidopsis. Plant Physiol. 2012;159(1):450-60. Epub 2012/03/07. doi: 10.1104/pp.112.195578. PubMed PMID: 22392280; PubMed Central PMCID: PMC3375977.

60. Munnik T, Irvine RF, Musgrave A. Phospholipid signalling in plants. Biochim Biophys Acta. 1998;1389(3):222-72. PubMed PMID: 9512651.

61. Distefano AM, Garcia-Mata C, Lamattina L, Laxalt AM. Nitric oxide-induced phosphatidic acid accumulation: a role for phospholipases C and D in stomatal closure. Plant Cell Environ. 2008;31(2):187-94. Epub 2007/11/13. doi: 10.1111/j.1365-3040.2007.01756.x. PubMed PMID: 17996010.

62. Zhao J, Wang X. Arabidopsis phospholipase Dalpha1 interacts with the heterotrimeric G-protein alpha-subunit through a motif analogous to the DRY motif in G-protein-coupled receptors. J Biol Chem. 2004;279(3):1794-800. doi: 10.1074/jbc.M309529200. PubMed PMID: 14594812.

63. Roy Choudhury S, Pandey S. The role of PLDalpha1 in providing specificity to signal-response coupling by heterotrimeric G-protein components in Arabidopsis. Plant J. 2016;86(1):50-61. doi: 10.1111/tpj.13151. PubMed PMID: 26935351.

64. Guo L, Wang X. Crosstalk between Phospholipase D and Sphingosine Kinase in Plant Stress Signaling. Front Plant Sci. 2012;3:51. Epub 2012/05/29. doi: 10.3389/fpls.2012.00051. PubMed PMID: 22639650; PubMed Central PMCID: PMC3355621.

65. Guo L, Devaiah SP, Narasimhan R, Pan X, Zhang Y, Zhang W, et al. Cytosolic glyceraldehyde-3-phosphate dehydrogenases interact with phospholipase Ddelta to transduce hydrogen peroxide signals in the Arabidopsis response to stress. Plant Cell. 2012;24(5):2200-12. Epub 2012/05/17. doi: 10.1105/tpc.111.094946. PubMed PMID: 22589465; PubMed Central PMCID: PMC3442596.

66. Otterhag L, Sommarin M, Pical C. N-terminal EF-hand-like domain is required for phosphoinositide-specific phospholipase C activity in Arabidopsis thaliana. FEBS Lett. 2001;497(2-3):165-70. Epub 2001/05/30. doi: S0014-5793(01)02453-X [pii]. PubMed PMID: 11377433.

67. Jung JY, Kim YW, Kwak JM, Hwang JU, Young J, Schroeder JI, et al. Phosphatidylinositol 3- and 4-phosphate are required for normal stomatal movements. Plant Cell. 2002;14(10):2399-412. Epub 2002/10/09. PubMed PMID: 12368494; PubMed Central PMCID: PMC151225.

68. Boss WF, Im YJ. Phosphoinositide signaling. Annu Rev Plant Biol. 2012;63:409-29. Epub 2012/03/13. doi: 10.1146/annurev-arplant-042110-103840. PubMed PMID: 22404474.

69. Lee Y, Choi YB, Suh S, Lee J, Assmann SM, Joe CO, et al. Abscisic Acid-Induced Phosphoinositide Turnover in Guard Cell Protoplasts of Vicia faba. Plant Physiol. 1996;110(3):987-96. PubMed PMID: 12226236.

70. Guo L, Mishra G, Taylor K, Wang X. Phosphatidic acid binds and stimulates Arabidopsis sphingosine kinases. J Biol Chem. 2011;286(15):13336-45. Epub 2011/02/19. doi: 10.1074/jbc.M110.190892. PubMed PMID: 21330371; PubMed Central PMCID: PMC3075680.

71. Coursol S, Fan LM, Le Stunff H, Spiegel S, Gilroy S, Assmann SM. Sphingolipid signalling in Arabidopsis guard cells involves heterotrimeric G proteins. Nature. 2003;423(6940):651-4. Epub 2003/06/06. doi: 10.1038/nature01643. PubMed PMID: 12789341.

72. Pandey S, Assmann SM. The Arabidopsis putative G protein-coupled receptor GCR1 interacts with the G protein alpha subunit GPA1 and regulates abscisic acid signaling. Plant Cell. 2004;16(6):1616-32. doi: 10.1105/tpc.020321. PubMed PMID: 15155892; PubMed Central PMCID: PMC490050.

73. Nakagawa N, Kato M, Takahashi Y, Shimazaki K, Tamura K, Tokuji Y, et al. Degradation of long-chain base 1-phosphate (LCBP) in Arabidopsis: functional characterization of LCBP phosphatase involved in the dehydration stress response. J Plant Res. 2012;125(3):439-49. Epub 2011/09/13. doi: 10.1007/s10265-011-0451-9. PubMed PMID: 21910031.

74. Irving HR, Gehring CA, Parish RW. Changes in cytosolic pH and calcium of guard cells precede stomatal movements. Proc Natl Acad Sci U S A. 1992;89(5):1790-4. PubMed PMID: 11607281.

75. Islam MM, Hossain MA, Jannat R, Munemasa S, Nakamura Y, Mori IC, et al. Cytosolic alkalization and cytosolic calcium oscillation in Arabidopsis guard cells response to ABA and MeJA. Plant Cell Physiol. 2010;51(10):1721-30. Epub 2010/08/27. doi: 10.1093/pcp/pcq131. PubMed PMID: 20739306.

76. Wang XQ, Ullah H, Jones AM, Assmann SM. G protein regulation of ion channels and abscisic acid signaling in Arabidopsis guard cells. Science. 2001;292(5524):2070-2. Epub 2001/06/16. doi: 10.1126/science.1059046. PubMed PMID: 11408655.

77. Leube MP, Grill E, Amrhein N. ABI1 of Arabidopsis is a protein serine/threonine phosphatase highly regulated by the proton and magnesium ion concentration. FEBS Lett. 1998;424(1-2):100-4. Epub 1998/04/16. PubMed PMID: 9537523.

78. Luo H, Morsomme P, Boutry M. The two major types of plant plasma membrane H+-ATPases show different enzymatic properties and confer differential pH sensitivity of yeast growth. Plant Physiol. 1999;119(2):627-34. PubMed PMID: 9952459; PubMed Central PMCID: PMC32140.

79. Bak G, Lee EJ, Lee Y, Kato M, Segami S, Sze H, et al. Rapid structural changes and acidification of guard cell vacuoles during stomatal closure require phosphatidylinositol 3,5-bisphosphate. Plant Cell. 2013;25(6):2202-16. Epub 2013/06/13. doi: 10.1105/tpc.113.110411. PubMed PMID: 23757398; PubMed Central PMCID: PMCPMC3723621.

80. Tang RJ, Liu H, Yang Y, Yang L, Gao XS, Garcia VJ, et al. Tonoplast calcium sensors CBL2 and CBL3 control plant growth and ion homeostasis through regulating V-ATPase activity in Arabidopsis. Cell Res. 2012;22(12):1650-65. doi: 10.1038/cr.2012.161. PubMed PMID: 23184060; PubMed Central PMCID: PMC3515760.

81. Brandt B, Brodsky DE, Xue S, Negi J, Iba K, Kangasjarvi J, et al. Reconstitution of abscisic acid activation of SLAC1 anion channel by CPK6 and OST1 kinases and branched ABI1 PP2C phosphatase action. Proc Natl Acad Sci U S A. 2012;109(26):10593-8. Epub 2012/06/13. doi: 10.1073/pnas.1116590109. PubMed PMID: 22689970; PubMed Central PMCID: PMC3387046.

82. Lee SC, Lan W, Buchanan BB, Luan S. A protein kinase-phosphatase pair interacts with an ion channel to regulate ABA signaling in plant guard cells. Proc Natl Acad Sci U S A. 2009;106(50):21419-24. Epub 2009/12/04. doi: 10.1073/pnas.0910601106. PubMed PMID: 19955427; PubMed Central PMCID: PMC2795491.

83. Geiger D, Maierhofer T, Al-Rasheid KA, Scherzer S, Mumm P, Liese A, et al. Stomatal closure by fast abscisic acid signaling is mediated by the guard cell anion channel SLAH3 and the receptor RCAR1. Sci Signal. 2011;4(173):ra32. Epub 2011/05/19. doi: 10.1126/scisignal.2001346. PubMed PMID: 21586729.

84. Demir F, Horntrich C, Blachutzik JO, Scherzer S, Reinders Y, Kierszniowska S, et al. Arabidopsis nanodomain-delimited ABA signaling pathway regulates the anion channel SLAH3. Proc Natl Acad Sci U S A. 2013;110(20):8296-301. Epub 2013/05/01. doi: 10.1073/pnas.1211667110. PubMed PMID: 23630285; PubMed Central PMCID: PMC3657796.

85. Du Z, Aghoram K, Outlaw WH, Jr. In vivo phosphorylation of phosphoenolpyruvate carboxylase in guard cells of Vicia faba L. is enhanced by fusicoccin and suppressed by abscisic acid. Arch Biochem Biophys. 1997;337(2):345-50. Epub 1997/01/15. PubMed PMID: 9016832.

86. Dittrich P, Raschke K. Malate metabolism in isolated epidermis of Commelina communis L. in relation to stomatal functioning. Planta. 1977;134(1):77-81. Epub 1977/01/01. doi: 10.1007/BF00390098. PubMed PMID: 24419583.

87. Ward JM, Schroeder JI. Calcium-Activated K+ Channels and Calcium-Induced Calcium Release by Slow Vacuolar Ion Channels in Guard Cell Vacuoles Implicated in the Control of Stomatal Closure. Plant Cell. 1994;6(5):669-83. doi: 10.1105/tpc.6.5.669. PubMed PMID: 12244253; PubMed Central PMCID: PMC160467.

88. Hosy E, Vavasseur A, Mouline K, Dreyer I, Gaymard F, Poree F, et al. The Arabidopsis outward K+ channel GORK is involved in regulation of stomatal movements and plant transpiration. Proc Natl Acad Sci U S A. 2003;100(9):5549-54. doi: 10.1073/pnas.0733970100. PubMed PMID: 12671068; PubMed Central PMCID: PMC154382.

89. Miedema H, Assmann SM. A membrane-delimited effect of internal pH on the K+ outward rectifier of Vicia faba guard cells. J Membr Biol. 1996;154(3):227-37. PubMed PMID: 8952952.

90. Kohler B, Hills A, Blatt MR. Control of guard cell ion channels by hydrogen peroxide and abscisic acid indicates their action through alternate signaling pathways. Plant Physiol. 2003;131(2):385-8. doi: 10.1104/pp.016014. PubMed PMID: 12586862; PubMed Central PMCID: PMC1540280.

91. Sokolovski S, Blatt MR. Nitric oxide block of outward-rectifying K+ channels indicates direct control by protein nitrosylation in guard cells. Plant Physiol. 2004;136(4):4275-84. doi: 10.1104/pp.104.050344. PubMed PMID: 15563619; PubMed Central PMCID: PMC535857.

92. Jiang Y, Wu K, Lin F, Qu Y, Liu X, Zhang Q. Phosphatidic acid integrates calcium signaling and microtubule dynamics into regulating ABA-induced stomatal closure in Arabidopsis. Planta. 2014;239(3):565-75. doi: 10.1007/s00425-013-1999-5. PubMed PMID: 24271006.
