# Supplementary material for: A new discrete dynamic model of ABA-induced stomatal closure predicts key feedback loops
Source: PLoS Biol. 2017 Sep 22;15(9):e2003451. doi: 10.1371/journal.pbio.2003451 (PMC5627951; doi:10.1371/journal.pbio.2003451)
Supplement: S2 Text — (DOCX) [file pbio.2003451.s018.docx]

**S2 Text. Detailed justification of the sustained state or Boolean regulatory function of each node in the model.**

***Source nodes, not regulated by any other node of the network***

***Source nodes assumed to be ON***

**ABA**

ABA is a phytohormone, the primary signal for the model, which plays a vital positive regulatory role for stomatal closure by propagating the message through ABA receptors via signaling cascades, which in turn trigger the change in osmotic potential of stomatal guard cells in response to drought, leading to guard cell volume loss and stomatal closure. Once activated, this node is assumed to be in a sustained ON state to model the sustained presence of the signal [1, 2].

**ABH1**

*ABH1* encodes an mRNA binding protein in Arabidopsis. The *abh1* (*AB*A *h*ypersensitive) loss-of-function mutant shows greater increases in cytosolic calcium and stomatal closure activation in response to ABA as compared to wild type. We assume that the wild type state of ABH1 is ON [3]. Because upstream regulators of ABH1 have not been identified, it is modeled as a source node.

**ARP Complex**

*arp2 (hsr3)* loss-of-function mutant exhibits reduced sensitivity to ABA and CaCl_2_ induced stomatal closure [4]. ARP2 is part of a protein complex involved in actin reorganization. In this particular case, the behavior of one subunit is assumed to describe the behavior of the protein complex. We assume that the wild type state of the ARP complex is ON.

**CPK6**

ABI1, ABI2, and PP2CA have been implicated as negative regulators of CPK6-mediated phosphorylation and thus activation of slow anion channels [5, 6]. A recent report indicates that PP2Cs (which function as negative regulators in guard cell ABA signaling) do not downregulate CPK6 kinase activity directly [7]. Thus we do not include ABI1, ABI2 and PP2CA as direct negative regulators of CPK6 in our model. CPK6 activity shows no or weak dependence on Ca^2+^_c_ [6]. As CPK6 is active at resting Ca^2+^_c_ levels, we assume that CPK6 is always on.

**CPK23**

ABI1 and ABI2 phosphatases have been implicated as negative regulators of CPK23-mediated activation of slow anion channels [8]. A recent report indicates that PP2Cs (which function as negative regulators in guard cell ABA signaling) do not downregulate CPK23 directly [7]. Thus we do not include ABI1 and ABI2 as direct negative regulators of CPK23 in our model. CPK23 activity shows little or weak dependence on Ca^2+^ [6, 8]. Hence, we assume that CPK23 is always on.

**DAGK**

DAGK is the enzyme that uses DAG as substrate for production of PA. We assume that DAGK is normally ON.

**ERA1**

ERA1 encodes a farnesyltransferase beta subunit. At low ABA concentrations, greater increases in cytosolic calcium and enhanced stomatal closure were observed in the *era1-2* knockout mutant in comparison to the wild type [9]. This mutant also shows hypersensitivity in ABA activation of slow anion channels [10]. We assume that the normal (wild type) state of ERA1 is ON.

**GAPC1/2**

GAPC1 and GAPC2 (glyceraldehyde-3-phosphate dehydrogenases) can interact with PLDδ [11]. We assume that these GAPCs are normally ON.

**GCR1**

GCR1 is a negative regulator of the sphingosine-1-phosphate (S1P) signal in guard cells and it can interact with GPA1. The *gcr1* loss-of-function mutant shows stronger ABA response than the wild type at moderate ABA concentrations, but interaction with GCR1 does not block G protein signaling [12]. We assume that unperturbed GCR1 is in the ON state.

**GTP**

GTP is the substrate for cGMP production. We assume that a sufficient amount of substrate is present, thus we assign this node the ON state.

**MRP5**

MRP5 encodes an ATP-binding cassette (ABC) transporter. The *mrp5* loss of function mutant shows partially impaired ABA-induced stomatal closure and assays of non-stretch-activated plasma membrane Ca^2+^ permeable channels show loss of activation in the *mrp5* mutant [13]. We assume that the undisrupted state of MRP5 is ON.

**NAD^+^**

NAD^+^ is a coenzyme that is required for cADPR production. We assume that a sufficient amount of co-enzyme is always present.

**NADPH**

NADPH is a coenzyme that is required for NO production. We assume that sufficient amount of co-enzyme is always present.

**Nitrite**

Nitrite is a substrate required for NO production. We assume that a sufficient amount of substrate is always present.

**NtSyp121**

Dominant-negative expression of the NtSyp121-Sp2 fragment inhibits opening of Ca^2+^ permeable channels [14] indicating that NtSyp121 is a positive regulator of CaIM. We assume that unperturbed NtSyp121 is in the ON state.

**PC**

PC is the substrate for PA production by both PLDα and PLDδ. We assume that a sufficient amount of substrate is always present.

**PtdInsP3, PtdInsP4**

LY294002 (inhibitor of PtdInsP3 biosynthesis) and wortmanin (inhibitor of PtdInsP4 biosynthesis) inhibit ABA-induced actin reorganization in guard cells, indicating that PtdInsP3 and PtdInsP4 are positive regulators of actin reorganization [15]. We assume that sufficient amounts of PtdInsP3 and PtdInsP4 are always present.

**RCN1**

RCN1 encodes the regulatory A subunit of a PP2A phosphatase. A T-DNA disruption mutant of *rcn1* showed hyposensitivity to ABA and lower ROS production [16]. We assume that undisrupted RCN1 is in the ON state. Because upstream regulators of RCN1 in guard cells have not been identified, it is modeled as a source node.

**SCAB1**

SCAB1 is an actin binding protein. The *scab1* loss-of-function mutant shows delayed stomatal closure and the mutant also shows slower actin reorganization in response to ABA in comparison to wild type plants [17]. We assume that undisrupted SCAB1 is in the ON state.

**Sph**

Sphingosine (Sph) is the substrate that is phosphorylated to produce S1P. SPHKs (1/2) use Sph for production of S1P. We assume that sufficient substrate is always present.

***Source nodes assumed to be OFF***

**GEF 1/4/10**

The *gef1gef4* double mutant (loss-of-function) and *gef1/4/10* triple mutant (loss-of-function) are hypersensitive in ABA-mediated stomatal responses. The *gef* double and triple mutants do not affect stomatal apertures in the absence of exogenous ABA [18-20]. We assume that the normal activity of GEF1/4/10 is below the threshold necessary to activate its downstream target ROP11, thus we assign it the OFF state. Assuming otherwise would not recapitulate the observed inhibitory effect of ABA on both ABI1 and ABI2 (see the rules for ABI1 and ABI2).

**SPP1**

Guard cells of the *atspp1* loss-of-function mutant show slightly enhanced stomatal closure over wild type in response to ABA. SPP1 encodes a long-chain base 1-phosphatase (LCBP) which has been implicated as a negative regulator of S1P accumulation in plants [21]. We assume that the normal activity of SPP1 is below the threshold required for efficient downregulation of S1P, and assign it the OFF state. Our model would capture the observed slight enhancement in stomatal closure in response to ABA in the *spp1* knockout [21] if we assumed that SPP1 is normally active (ON). However, this assumption, coupled with the knowledge that SPP1 is a negative regulator of S1P, would lead to the insufficiency of ABA to lead to S1P production, which contradicts observations [22, 23].

***Boolean regulatory functions of regulated nodes***

**RCARs*=ABA**

RCARs (alternatively known as PYR/PYLs) are soluble ABA receptors. These proteins directly bind ABA. Upon binding to ABA, these proteins inhibit ABI1, ABI2, HAB1 and PP2CA [1, 2].

**PEPC*= not ABA**

ABA inhibits PEP carboxylase (PEPC) activity in guard cells [24].

**PI3P5K*= ABA**

ABA indirectly promotes PI3P5K activity [25].

**ROP11*= GEF1/4/10**

ROP11 is a small GTPase that binds and hydrolyzes GTP. GEFs facilitate disassociation of bound GDP from ROP11, which in turn allows ROP11 to associate with GTP and achieve its active state [18, 26].

**ABI1*=not PA and (not RCARs or ROP11) and not ROS and pH_c_**

RCARs inhibit ABI1 through physical binding [1]. ROP11 interacts with ABI1 and protects it from inhibition by RCARs [20]. Upon binding PA inhibits phosphatase activity of ABI1 [27]. PA also tethers ABI1 to the plasma membrane, which in turn negatively regulates ABI1 function [28]. ROS inhibit ABI1 activity [29]. Cytosolic pH increase activates enzyme activity of ABI1 [30].

**ABI2*= (not RCARS or ROP11) and not ROS**

ROP11 physically interacts with ABI2 and promotes its phosphatase activity [18]. RCARs inhibit phosphatase activity of ABI2 by physical binding [1]. ABI2 has been shown to be negatively regulated by ROS [31]; we assume that the absence or low level of ROS is a necessary condition for ABI2 activity. In addition to this requirement, we assume that in order for ABI2 to be active, its positive regulator ROP11 must be active or its other negative regulators, RCARs, must be off.

**HAB1*= not RCARs and not ROS**

Upon ABA binding, RCARs interact with the PP2C-type phosphatases ABI1/ABI2/HAB1/PP2CA and inhibit their phosphatase activity. Thus, RCARs are negative regulators of these protein phosphatases [1, 2]. ROS oxidize H_2_O_2_-sensitive thiols and inhibit HAB1’s catalytic activity [32]. We assume that either RCARs or ROS suffices to inhibit HAB1 phosphatase activity and combine these inhibitors with an “and” function.

**PP2CA*= not RCARS and not ROS**

In an *in vitro* study, it has been shown that soluble ABA receptors (RCARs, alternatively known as PYR/PYLs) PYR1, PYL1, PYL2, PYL4, PYL5, PYL6, PYL8 inhibit the phosphatase activity of PP2CA in the presence of ABA [33]. ROS-mediated inhibition has been reported for three PP2C-type protein phosphatases that play negative regulatory roles in guard cell ABA signaling: ABI1, ABI2 and HAB1 [29, 31, 32]. We assume that ROS would similarly inhibit the phosphatase activity of PP2CA.

**OST1*= (not ABI1 and not HAB1) or (not PP2CA and not ABI2) or (not ABI1 and not ABI2) or (not HAB1 and not PP2CA) or (not HABI1 and not ABI2) or (not ABI1 and not PP2CA)**

The indicated PP2C type protein phosphatases inhibit OST1 [2, 34, 35]. The recessive double knockout mutant *abi1* *abi2* shows higher stomatal responsiveness to ABA than single recessive mutants of *abi1* and *abi2* [36]. Similarly, the *abi1* *hab1* double knockout mutant shows greater stomatal closure in response to ABA than the corresponding single knockout mutants [37]. Another study indicates that *hab1 pp2ca1* and *abi1 pp2ca1* double knockout mutants show stronger ABA-mediated stomatal response than the corresponding single knockout mutants [38]. These findings suggest that the indicated protein phosphatases have overlapping and cooperative roles in guard cell ABA signaling. Also, these findings suggest that in an *in planta* scenario, for OST1 to be active (in response to ABA), at least two of the indicated protein phosphatases must be inactive.

**RBOH*= pH_c_ and not ABI1 and PtdInsP3 and OST1 and GPA1 and PA and RCN1**

RBOH enzymes catalyze ROS production in guard cells in response to ABA-activated signaling [39]. ABA-induced cytosolic alkalization (pH_c_ increase) is necessary for ROS production [40]. In comparison to wild-type, guard cells of *ost1* loss-of-function mutants show less ABA-induced ROS production whereas guard cells of OST1 overexpression plants accumulate more ROS [41, 42]. Also, OST1 phosphorylates RBOH [41, 43]. We assume that OST1 activates RBOH via phosphorylation. Guard cells of the *abi1-1* mutant (dominant negative) do not show ABA induced ROS production, indicating that ABI1 is a negative regulator of RBOH [44]. *gpa1* loss-of-function mutants are hyposensitive to ABA-mediated ROS production [45]. PA is shown to activate RBOH oxidase activity [46]; we assume that the effect of PA is not independent of the other positive regulators and thus use an “and” rule. Inhibition of PI3K, the enzyme responsible for PtdInsP3 production, causes decreased ROS production and stomatal closure [47] indicating the importance of PtdInsP3 in ABA-induced ROS production. PtdInsP3-mediated activation of RBOH has not been shown in plants yet but in neutrophils, PtdInsP3 binding activates the NADPH oxidase complex [48]. We assume PtdInsP3 is a required positive regulator of RBOH. *rcn1* knockout mutants showed no ROS production during ABA signaling. We assume that RCN1 is required for the activity of RBOH [16].

**ROS*= NADPH and RBOH**

RBOH and NADPH have an enzyme and coenzyme relationship that is explained by an AND rule. There is evidence that CuAO enhances ROS production in response to ABA [49] but this is a weaker and less documented effect than RBOH; thus CuAO is not included in the ROS rule.

**GHR1*= not ABI2 and ROS**

ABI2 inhibits GHR1 and GHR1-mediated ROS signal propagation. The *ghr1* loss-of-function mutant does not show ROS-mediated stomatal closure [50]. For GHR1 to activate, ABI2 must not be activated whilst ROS is activated.

**NO*= Nitrite and NIA1/2 and NADPH**

Nitrite, NIA1/2, and NADPH comprise a substrate, enzyme, and coenzyme relationship respectively [51]; all three are required for NO production.

**NIA1/2*= ROS**

NIA1 and NIA2 are nitrate reductases. In the NIA1/2 double mutant (loss-of-function), *nia1* *nia2*, ROS fails to induce NO production [52], thus we assume that ROS is the sole, positive, regulator of NIA1/2.

**NOGC1*= NO**

NO promotes the enzyme activity of the guanylate cyclase NOGC1 [53].

**cGMP*= NOGC1 and GTP**

NOGC1 (enzyme) uses GTP (substrate) for the production of cGMP [53].

**8-nitro-cGMP*= cGMP and ROS and NO**

 8-nitro-cGMP is a point where the ROS and NO signaling pathways converge: ROS and NO activate together and are both required for 8-nitro-cGMP synthesis [54]. cGMP is the substrate in this reaction and is required to be present. Thus all three upstream components are connected by “and” relationships.

**ADPRc*= 8-nitro-cGMP**

cADPR is a product of the enzyme ADPRc and functions as a second messenger for modulation of intracellular calcium levels. Both nicotinamide [55] and 8-bromo-cADPR [56, 57] are antagonists of cADPR production. These two inhibitors themselves do not show any effect on stomatal aperture but both inhibit 8-nitro-cGMP mediated stomatal closure. These findings suggest that 8-nitro-cGMP indirectly activates ADPRc [54].

**cADPR*= NAD^+^  and ADPRc**

ADPRc (enzyme) uses NAD^+^ (substrate) for production of cADPR.

**CIS*= InsP3 or InsP6 or cADPR**

cADPR is an important signaling molecule leading to calcium release from internal stores [58]. InsP3 and InsP6 can each independently release calcium from internal stores [59, 60]. Hence we use an “or” function between InsP3 and InsP6. We assume that activation of calcium influx (to the cytosol) from stores (CIS) can happen independently via the inositol phosphate or via the cADPR pathway, and thus use “or” functions between all three regulators.

**CaIM*= Actin Reorganization or (NtSyp121 and GHR1 and MRP5) or not ABH1 or not ERA1**

Ca^2+^ influx across the plasma membrane is mediated by two types of plasma membrane Ca^2+^ permeable channels: stretch-activated channels [61] and those not related to stretch. Many experiments focus on the second type. While much less is known about the regulation of the stretch-activated channels, it is an independent mechanism activated by actin reorganization; we assume that it can also lead to a sufficient amount of Ca^2+^ influx.

NtSyp121-sp2, a dominant negative protein fragment of NtSyp121, inhibits Ca^2+^ influx across the plasma membrane through non-stretch-activated Ca^2+^ channels, indicating that NtSyp121 is a positive regulator of CaIM [14]. *ghr1* loss-of-function mutants show impaired Ca^2+^ influx through non-stretch-activated Ca^2+^ channels and is shown to be part of ROS signaling [50]. The *mrp5* loss of function mutant shows no ABA activation of non-stretch-activated plasma membrane Ca^2+^ permeable channels [13]. At low ABA concentrations, greater increases in cytosolic Ca^2+^ and stomatal closure activation were observed in the *era1-2* loss-of-function mutant in comparison to the wild type. These observations suggest that ERA1 inhibits cytosolic Ca^2+^ influx. The *abh1* mutant shows greater increases in cytosolic Ca^2+^ in response to ABA compared to the wild type [3].

**Ca­­^2+^_c_ *= (CIS or CaIM) and not Ca^2+^ ATPase**

Both CIS and CaIM (Ca­­^2+^ Influx across the plasma Membrane) cause increase of cytosolic Ca^2+^ (Ca­­_c_^2+^_c_). The Ca­­^2+^ ATPase pumps Ca­­^2+^ from the cytosol to the apoplast.

**Ca­­^2+^ ATPase*= Ca­­^2+^ _c_**

The mechanisms that mediate Ca^2+^ efflux from the cytosolic compartment are incorporated by including Ca^2+^ ATPases and Ca^2+^/H^+^ antiporters as the node “Ca^2+^ ATPase” in our model. Calcium oscillations occur in response to ABA. This rule is the simplest way to incorporate calcium concentration oscillations in our model [62].

**CPK3/21*= Ca^2+^­­_c_ or CPK3/21**

Cytosolic calcium activates CPK3 and CPK21 by binding to their EF hand calcium binding motifs. ABI1 has been implicated as a negative regulator of CPK3/21 mediated activation of slow anion channels [6, 8]. A recent report indicates that PP2Cs (which act as negative regulators in guard cell ABA signaling) do not downregulate the activity of different CPKs (e.g. CPK6) directly [7]. ABI1 has been shown to downregulate SLAC1 by direct dephosphorylation which inhibits SLAC1 activity [7]. We assume that ABI1 does not directly regulate the kinase activity of CPK3/21. Thus we do not include ABI1 as a negative regulator of CPK3/21 in our model. We assume that once activated by Ca^2+^_c,_ CPK3 and CPK21 remain in the active state through autophosphorylation [63], and implement this assumption by using the “or” operator.

**MPK9/12*=Ca^2+^­­_c_ or MPK9/12**

MAP kinases MPK9 and MPK12 play positive regulatory roles in Ca^2+^ activation of S-type anion channels. The *mpk9 mpk12* double mutant (loss-of-function) does not show Ca^2+^ activation of S-type anion channels [64]. We assume from these results that Ca^2+^_c_ is a positive regulator of MPK9/12. We assume that once activated, MPK9 and MPK12 remain active due to autophosphorylation [65], and implement this assumption by using the “or” operator.

**PtdIns(4,5)P2*= PtdInsP4**

PtdInsP4 is a known precursor of PtdIns(4,5)P2 [66].

**PLC^*^= Ca^2+^_c_**

InsP3 production by the enzyme phospholipase C (PLC) requires calcium [67]. We accordingly assume that Ca^2+^_c_ activates PLC.

**DAG*= PtdIns(4,5)P2 and PLC**

PLC (enzyme) uses PtdIns(4,5)P2 (substrate) for production of DAG.

**InsP3*= PLC and PtdIns(4,5)P2**

PLC (enzyme) uses substrate PtdIns(4,5)P2 for production of InsP3.

**InsP6*= InsP3**

Triple phosphorylation of InsP3 can yield InsP6 [68]

**PLDα*= GPA1 and Ca^2+^­­_c_**

Cytosolic calcium promotes PLDα translocation to the plasma membrane and tonoplast, where its substrate phospholipids reside. PLDα and GPA1 bind to each other through the DRY motif [69]. The GDP bound form of GPA1 inhibits PLDα activity. The GTP bound form of GPA1 is required for PLDα to be active [28, 69]. Hence an “and” function is used between GPA1 and Ca­^2+^_c_.

**PLDδ*= NO or ROS and GAPC1/2**

Loss-of-function mutants of *pldδ* do not show stomatal closure in response to NO, indicating that PLDδ is a player in NO signaling, although details of the interaction between NO and PLDδ are unknown [70]. ROS promotes the interaction between GAPC1/2 and PLDδ, which in turn promotes PLDδ activity [11]. We used an “or” function as either NO, or ROS and GAPC1/2 together, could activate PLDδ independently.

**PA*= PC and (PLDδ or PLDα) or DAG and DAGK**

PC is the substrate needed by PLDα or PLDδ for PA production. DAG, a product of PLC, can be converted into PA by DAGK-mediated phosphorylation [71, 72].

**SPHK1/ 2*= PA or ABA**

SPHK1 and SPHK2 are sphingosine kinases. Guard cell sphingosine kinase activity is stimulated by ABA in Arabidopsis [22]. Upon binding, PA increases SPHK1/2 activity [73]. An “or” function is used since there are no available data that SPHK1/2 require both PA and ABA to activate [74].

**S1P/PhytoS1P*= SPHK1/2 and Sph and not SPP1**

SPHK1/2 and Sph are the enzymes and substrate needed for S1P production. SPP1 encodes a long-chain base 1-phosphatase (LCBP) which has been implicated as a negative regulator of S1P accumulation in plants [21].

Time course data indicate that in response to ABA S1P accumulation increases quickly and then decreases gradually, subsequently stabilizing above the resting level. It was suggested that this reflects negative feedback regulation of S1P/PhytoS1P [22, 62, 75]. In the absence of any identified mechanism, and since the stabilized level is above the initial level, we do not include this putative negative regulation into the regulatory function.

**GPA1*=  S1P/phytoS1P or not GCR1**

GCR1 is a candidate GPCR that is hypothesized to be a negative regulator of GPA1, given ABA hyposensitivity of *gpa1* guard cells and ABA hypersensitivity of *gcr1* guard cells [12, 76]. GPA1 is required for S1P-mediated stomatal response, so it is assumed that S1P functions through GPA1 [12, 22, 62].

**PtdIns(3,5)P2*= PI3P5K**

The enzyme PI35PK produces PtdIns(3,5)P2 [25].

**V-PPase*= PtdIns(3,5)P2**

Single and double null mutants of PI3P5K (the enzyme that produces PtdIns(3,5) P2) exhibit slow stomatal closure in comparison to the wild type in response to ABA application. Inhibition of PI3P5K through pharmacological agents reduced vacuolar acidification and delayed stomatal closure. These findings indicate that the product of the PI3P5K-catalyzed reaction, PtdIns(3,5)P2, is necessary for vacuolar acidification [25]. PtdIns(3,5)P2 binds to the V-PPase (vacuolar pyrophosphatase). It has been suggested (and we assume) that upon binding PtdIns(3,5)P2 activates the V-PPase [25].

**V-ATPase*= Ca^2+^_c_**

Ca^2+^_c_ has been implicated as a positive regulator of the V-ATPase [77].

**Vacuolar Acidification*= V-PPase or V-ATPase or Vacuolar Acidification**

In yeast, the vacuolar proton ATPase (V-ATPase) proton pump plays an important role in vacuolar acidification [78, 79]. The proton pumping vacuolar pyrophosphatase (V-PPase) uses the energy of PP_i_ hydrolysis to acidify the vacuole [80]. An Arabidopsis V-PPase loss-of-function mutant, *vhp1,* shows delayed vacuolar acidification and slower stomatal closure in response to ABA [25, 81]. An Arabidopsis double knockout mutant of V-ATPase, *vha1 vha2*, exhibits a vacuolar pH of 6.4 rather than 5.9 [82]. The double knockout mutant of the V-ATPase also shows delayed stomatal closure in response to ABA [25]. We used an “or” function between V-PPase and V-ATPase as the V-PPase and V-ATPase play independent roles in vacuolar acidification [25]. We assume that the vacuolar acidification state is sustained for a longer period, and implement this assumption as a positive self-regulation. This assumption is necessary in order to allow the possibility of closure in response to internal closure signals (e.g. supply of S1P or Ca^2+^ [83]).

**TCTP*= Ca­­^2+^ _c_**

Ca^2+^_c_ promotes the interaction between AtTCTP and microtubules [84]. We incorporate this effect by assuming that Ca^2+^_c_ is a positive regulator of TCTP.

**Microtubule Depolymerization*= TCTP or Microtubule Depolymerization**

Interaction between AtTCTP and microtubules promotes microtubule depolymerization [84]. Microtubule depolymerization continues forward once initiated by a process termed catastrophe [85]; we assume that it is not reversed during the ABA signaling process and implement this assumption by using the “or” operator.

**pH_c_*= (OST1 and not ABI2 and not ABI1 or Ca­­^2+^_c_) and Vacuolar acidification**

Guard cells of *abi1-1* (dominant negative)*, abi2-1* (dominant negative)*,* and *ost1-2* loss of function mutants show impaired cytosolic alkalization in response to ABA [86]. Exogenous calcium application is assumed to increase Ca­­^2+^_c_ concentration and can induce cytosolic alkalization in *ost1-2* (loss-of-function)*, abi1-1* (dominant negative)*, and abi2-1* (dominant negative) mutants [86], indicating that Ca^2+^_c_-triggered pH_c_ increase in guard cells does not require functional OST1 or the inactivation of ABI1 or ABI2; hence the OR relationship between Ca^2^ **_c_** and these three proteins. External application of a calcium chelator, EGTA, reduces ABA-induced cytosolic alkalization (pH_c_ increase) [87], indicating that Ca^2+^_c_ is a positive regulator in ABA-induced cytosolic alkalization. Vacuolar acidification is a necessary condition for maintenance of ABA induced cytosolic alkalization state in guard cells [25]. Thus we use an “and” function between Vacuolar acidification and other indicated positive and negative regulators of pH_c_ increase.

Time course data indicate that in response to ABA, the pH_c_ level increases quickly and then decreases gradually, subsequently stabilizing above the resting level. It has been suggested that this reflects negative feedback regulation [40, 62, 75], but no mediators have been identified. pH_c_ forms a negative feedback loop with ABI1, but this would yield a delay in ABA-induced pH_c_ increase and not the observed decrease. Since the stabilized pH_c_ level is well above the resting level, we do not include this suggested negative feedback regulation into the regulatory function.

**H^+^ ATPase*= not pH_c_ and not Ca^2+^­­_c_ and not ROS**

The H^+^ ATPase has been shown to be inhibited independently by reactive oxygen species [88], cytosolic calcium concentration increase [89], or cytosolic H^+^ concentration decrease [90]. Any one of these inhibitors can suffice to inhibit the H^+^ ATPase. Accordingly, the “and” function is used between these negative regulators [62].

**AtRAC1*= not ABA or ABI1**

ABA treatment causes inactivation of AtRAC1 and promotion of actin reorganization in guard cells. The *abi1-1* dominant negative mutant shows impaired actin reorganization and stomatal closure in response to ABA [91]. The dominant negative form of AtRAC1 was able to recover stomatal closure in *abi1-1.*  These findings suggest that ABI1 is a positive regulator of AtRAC1.

**Actin Reorganization*= (PtdInsP4 or PtdInsP3) and not AtRAC1 and ARP Complex and SCAB1**

Expression of a dominant-positive mutant of AtRAC1 inhibits ABA-induced actin reorganization whereas expression of a dominant-negative mutant of AtRAC1 promotes actin reorganization in the absence of ABA [91]. Inhibition of AtRAC1 is necessary for actin reorganization in response to ABA. ARP2 knockouts did not exhibit actin reorganization during ABA signaling. The *arp2* phenotype was rescued upon application of an actin depolymerizing agent which illustrates that ARP2 is a positive regulator of the actin reorganization process [4]. In this particular case, the behavior of one subunit is assumed to describe the behavior of the protein complex [4].

In Arabidopsis, SCAB1 encodes a plant specific actin binding protein. The *scab1* loss-of-function mutant shows a delayed ABA response that is associated with delayed actin reorganization [17]. Both PtdInsP4 and PtdInsP3 are implicated as positive regulators of actin reorganization in response to ABA [15]. In summary, inhibition of AtRAC1, as well as activation of PtdInsP3, PtdInsP4 and ARP2 are necessary for actin reorganization.

**SLAC1*= (CPK6 or CPK23 or CPK3/21) and MPK9/12 and OST1 and GHR1 and not ABI1 and not PP2CA and not ABI2 and pH_c_**

OST1 activates SLAC1 via phosphorylation [92]. Guard cells of the *ost1* mutant show impaired ABA activation of slow anion channels, thus OST1 is a required activator of SLAC1 [41, 92]. All indicated CPKs activate SLAC1 activity by phosphorylation [5, 6, 8]. A CPK23 loss of function mutant impairs SLAC1 activation by Ca^2+^ [8]. Guard cells of the *cpk3 cpk6* double knockout mutant show reduced sensitivity to ABA activation of slow anion channels [93]. We assume that each CPK is independently sufficient to activate slow anion channels, if other requirements are met. MPK9 and MPK12 (MPK9/12) play an important role in the activation of S-type anion channels by ABA and Ca^2+^_c_, although no physical interaction between MPK9/12 and anion channels has been shown. Guard cells of the *mpk9 mpk12* double mutant do not show ABA or calcium activation of slow anion channel activity [64], thus we used an “and” function for MPK9/12. GHR1-mediated phosphorylation activates SLAC1 activity. The *ghr1* knockout mutant shows impaired ABA and ROS activation of slow anion channels [50]. The *ghr1 ost1* double knockout mutant shows greater sensitivity to drought stress in comparison to single knockout mutants [50]. Thus we used an “and” function for GHR1. Clamping cytosolic pH inhibits ABA activation of slow anion channel activity [76]. ABI1, ABI2, and PP2CA have been shown to inhibit OST1-, CPK23-, CPK21- and CPK6-mediated activation of SLAC1 in Xenopus oocytes. A recent report indicates that PP2Cs dephosphorylate SLAC1 phosphosites that are essential for SLAC1 function, thus we assume that the inactivation of ABI1, ABI2 and PP2CA is necessary for SLAC1 activity [7].

**QUAC1*= OST1 and Ca^2+^­­_c_**

OST1 physically interacts with QUAC1 and activates this R-type anion channel [94]. The *ost1-2* loss of function mutant shows hyposensitivity for ABA activation of QUAC1 [94]. The QUAC1 loss-of-function mutant shows hyposensitivity to Ca^2+^-induced stomatal closure [95] indicating that QUAC1 is an effector in Ca­­_c_^2+^**-**mediated stomatal closure. These findings suggest that both OST1 and Ca­­_c_^2+^ are required for ABA activation of QUAC1.

S**LAH3*= (CPK6 or CPK23) and CPK3/21 and not ABI1**

All listed CPKs activate SLAH3 by physical interaction [6, 96]. All indicated CPKs have an independent positive effect on SLAH3. ABI1 inhibits CPK21-mediated activation of SLAH3 in oocytes indicating that ABI1 is a negative regulator of SLAH3 [96]. Since CPK6 and CPK23 are weakly dependent on ABA [7], we implement the dependence of SLAH3 activation on ABA [6, 96] by assuming that only the simultaneous activity of CPK6 and CPK3/21, or CPK23 and CPK3/21, is sufficient for SLAH3 activation.

**AnionEM*= SLAC1 or QUAC1 and SLAH3**

SLAC1 facilitates transport of a larger variety of anionic species (Cl^-^ and NO_3_^-^) than QUAC1 (malate^2-^) and SLAH3 (NO_3_^-^) [94, 97]. *slac1* loss-of-function mutant plants are insensitive to ABA-mediated stomatal closure [98, 99]. *quac1* loss-of-function mutants show partially impaired stomatal closure in response to ABA [95, 100]. *slah3* loss-of-function mutants have not shown any change in stomatal phenotype [96]. These findings suggest that SLAC1 is a major player in comparison to SLAH3 and QUAC1 in ABA-mediated stomatal response, which is why SLAC1 is given greater influence in this equation for Anion Efflux across the Membrane (AnionEM).

**Malate*= PEPC and not ABA and not AnionEM**

PEPC is an upstream enzyme of malate biosynthesis. ABA inhibits PEPC activity in guard cells [24]. ABA also negatively regulates malate concentration by inducing malate breakdown [101]^.^  Anion efflux (AnionEM) negatively regulates intracellular malate concentration by releasing malate from the cytosol [62].

**KEV*= Vacuolar Acidification or Ca­­^2+^ _c_**

Calcium induces K^+^ release through K^+^-permeable channels in the tonoplast [102]. Vacuolar acidification also induces K^+^ efflux from the vacuole [102].

**Depolarization*= (AnionEM or Ca­­^2+^ _c_ or KEV) and (not H^+^ ATPase or not K^+^ efflux)**

Efflux of cations or anions will hyperpolarize or depolarize the cell membrane, respectively. Anion efflux and Ca­­^2+^ influx across the plasma membrane, and release of K^+^ from the vacuole, are all events promoting plasma membrane depolarization, thus we connect them with “or” rules. KOUT (K^+^ efflux across the membrane) and H^+^ ATPase activity are events that negatively regulate plasma membrane depolarization [62]. We assume that the absence of either of these events, coupled with the presence of AnionEM, Ca^2+^­­_c_ or KEV, leads to plasma membrane depolarization.

**KOUT*= (not NO or not ROS or pH_c_) and Depolarization**

Membrane depolarization drives K^+^ efflux from the guard cell. Outwardly rectifying K^+^ channels are activated by cytosolic pH increase [103] and inhibited by ROS [104] and nitric oxide [105]. K^+^ efflux through outwardly rectifying K^+^ channels requires membrane depolarization, thus we use an “and” function between “Depolarization” and other indicated positive or negative regulators of KOUT. In the absence of documented synergy we use an “or” function between NO, ROS and pH_c_.

**K^+^ efflux*= KEV and KOUT**

Sustained efflux of K^+^ from the guard cell requires K^+^ efflux from the vacuole to the cytosol (KEV) followed by K^+^ efflux from the cytosol to the apoplast, mediated by outwardly rectifying K^+^ channels (KOUT). Hence we use an “and” function between KEV and KOUT.

**Aquaporin (PIP2;1)*= OST1**

OST1-mediated phosphorylation activates aquaporin Plasma membrane Intrinsic Protein 2;1 (PIP2;1) in guard cells in response to ABA [106].

**H_2_O Efflux*= (AnionEM and PIP2;1 and K^+^ efflux) and not Malate**

Both anion and K^+^ efflux are required for H_2_O efflux, which drives stomatal closure. Malate is an osmoticum that inhibits this process by decreasing water potential in the cell. Aquaporins (PIP2;1) facilitate water efflux during stomatal closure. Guard cells of a knockout mutant lacking PIP2;1 have a defect in ABA induced stomatal closure [106].

**Closure*= Microtubule Depolymerization and H_2_O efflux**

Microtubule depolymerization and H_2_O efflux are both needed for stomatal closure [107].

References

1. Ma Y, Szostkiewicz I, Korte A, Moes D, Yang Y, Christmann A, et al. Regulators of PP2C phosphatase activity function as abscisic acid sensors. Science. 2009;324(5930):1064-8. Epub 2009/05/02. doi: 10.1126/science.1172408. PubMed PMID: 19407143.

2. Park SY, Fung P, Nishimura N, Jensen DR, Fujii H, Zhao Y, et al. Abscisic acid inhibits type 2C protein phosphatases via the PYR/PYL family of START proteins. Science. 2009;324(5930):1068-71. Epub 2009/05/02. doi: 10.1126/science.1173041. PubMed PMID: 19407142; PubMed Central PMCID: PMC2827199.

3. Hugouvieux V, Kwak JM, Schroeder JI. An mRNA cap binding protein, ABH1, modulates early abscisic acid signal transduction in Arabidopsis. Cell. 2001;106(4):477-87. Epub 2001/08/30. PubMed PMID: 11525733.

4. Jiang K, Sorefan K, Deeks MJ, Bevan MW, Hussey PJ, Hetherington AM. The ARP2/3 complex mediates guard cell actin reorganization and stomatal movement in Arabidopsis. Plant Cell. 2012;24(5):2031-40. Epub 2012/05/10. doi: 10.1105/tpc.112.096263. PubMed PMID: 22570440; PubMed Central PMCID: PMC3442585.

5. Brandt B, Brodsky DE, Xue S, Negi J, Iba K, Kangasjarvi J, et al. Reconstitution of abscisic acid activation of SLAC1 anion channel by CPK6 and OST1 kinases and branched ABI1 PP2C phosphatase action. Proc Natl Acad Sci U S A. 2012;109(26):10593-8. Epub 2012/06/13. doi: 10.1073/pnas.1116590109. PubMed PMID: 22689970; PubMed Central PMCID: PMC3387046.

6. Scherzer S, Maierhofer T, Al-Rasheid KA, Geiger D, Hedrich R. Multiple calcium-dependent kinases modulate ABA-activated guard cell anion channels. Mol Plant. 2012;5(6):1409-12. Epub 2012/08/31. doi: 10.1093/mp/sss084. PubMed PMID: 22933711.

7. Brandt B, Munemasa S, Wang C, Nguyen D, Yong T, Yang PG, et al. Calcium specificity signaling mechanisms in abscisic acid signal transduction in Arabidopsis guard cells. eLife. 2015;4. Epub 2015/07/21. doi: 10.7554/eLife.03599. PubMed PMID: 26192964; PubMed Central PMCID: PMC4507714.

8. Geiger D, Scherzer S, Mumm P, Marten I, Ache P, Matschi S, et al. Guard cell anion channel SLAC1 is regulated by CDPK protein kinases with distinct Ca2+ affinities. Proc Natl Acad Sci U S A. 2010;107(17):8023-8. Epub 2010/04/14. doi: 10.1073/pnas.0912030107. PubMed PMID: 20385816; PubMed Central PMCID: PMC2867891.

9. Allen GJ, Murata Y, Chu SP, Nafisi M, Schroeder JI. Hypersensitivity of abscisic acid-induced cytosolic calcium increases in the Arabidopsis farnesyltransferase mutant era1-2. Plant Cell. 2002;14(7):1649-62. Epub 2002/07/18. PubMed PMID: 12119381; PubMed Central PMCID: PMC150713.

10. Pei ZM, Ghassemian M, Kwak CM, McCourt P, Schroeder JI. Role of farnesyltransferase in ABA regulation of guard cell anion channels and plant water loss. Science. 1998;282(5387):287-90. PubMed PMID: 9765153.

11. Guo L, Devaiah SP, Narasimhan R, Pan X, Zhang Y, Zhang W, et al. Cytosolic glyceraldehyde-3-phosphate dehydrogenases interact with phospholipase Ddelta to transduce hydrogen peroxide signals in the Arabidopsis response to stress. Plant Cell. 2012;24(5):2200-12. Epub 2012/05/17. doi: 10.1105/tpc.111.094946. PubMed PMID: 22589465; PubMed Central PMCID: PMC3442596.

12. Pandey S, Assmann SM. The Arabidopsis putative G protein-coupled receptor GCR1 interacts with the G protein alpha subunit GPA1 and regulates abscisic acid signaling. Plant Cell. 2004;16(6):1616-32. doi: 10.1105/tpc.020321. PubMed PMID: 15155892; PubMed Central PMCID: PMC490050.

13. Suh SJ, Wang YF, Frelet A, Leonhardt N, Klein M, Forestier C, et al. The ATP binding cassette transporter AtMRP5 modulates anion and calcium channel activities in Arabidopsis guard cells. J Biol Chem. 2007;282(3):1916-24. Epub 2006/11/14. doi: 10.1074/jbc.M607926200. PubMed PMID: 17098742.

14. Sokolovski S, Hills A, Gay RA, Blatt MR. Functional interaction of the SNARE protein NtSyp121 in Ca2+ channel gating, Ca2+ transients and ABA signalling of stomatal guard cells. Mol Plant. 2008;1(2):347-58. Epub 2008/03/01. doi: 10.1093/mp/ssm029. PubMed PMID: 19825544.

15. Choi Y, Lee Y, Jeon BW, Staiger CJ, Lee Y. Phosphatidylinositol 3- and 4-phosphate modulate actin filament reorganization in guard cells of day flower. Plant Cell Environ. 2008;31(3):366-77. Epub 2007/12/20. doi: 10.1111/j.1365-3040.2007.01769.x. PubMed PMID: 18088331.

16. Saito N, Munemasa S, Nakamura Y, Shimoishi Y, Mori IC, Murata Y. Roles of RCN1, regulatory A subunit of protein phosphatase 2A, in methyl jasmonate signaling and signal crosstalk between methyl jasmonate and abscisic acid. Plant Cell Physiol. 2008;49(9):1396-401. Epub 2008/07/25. doi: 10.1093/pcp/pcn106. PubMed PMID: 18650210.

17. Zhao Y, Zhao S, Mao T, Qu X, Cao W, Zhang L, et al. The plant-specific actin binding protein SCAB1 stabilizes actin filaments and regulates stomatal movement in Arabidopsis. Plant Cell. 2011;23(6):2314-30. Epub 2011/07/02. doi: 10.1105/tpc.111.086546. PubMed PMID: 21719691; PubMed Central PMCID: PMC3160031.

18. Yu F, Qian L, Nibau C, Duan Q, Kita D, Levasseur K, et al. FERONIA receptor kinase pathway suppresses abscisic acid signaling in Arabidopsis by activating ABI2 phosphatase. Proc Natl Acad Sci U S A. 2012;109(36):14693-8. Epub 2012/08/22. doi: 10.1073/pnas.1212547109. PubMed PMID: 22908257; PubMed Central PMCID: PMC3437822.

19. Li Z, Liu D. ROPGEF1 and ROPGEF4 are functional regulators of ROP11 GTPase in ABA-mediated stomatal closure in Arabidopsis. FEBS Lett. 2012;586(9):1253-8. Epub 2012/04/17. doi: 10.1016/j.febslet.2012.03.040. PubMed PMID: 22500990.

20. Li Z, Gao X, Chinnusamy V, Bressan R, Wang ZX, Zhu JK, et al. ROP11 GTPase negatively regulates ABA signaling by protecting ABI1 phosphatase activity from inhibition by the ABA receptor RCAR1/PYL9 in Arabidopsis. J Integr Plant Biol. 2012;54(3):180-8. Epub 2012/01/19. doi: 10.1111/j.1744-7909.2012.01101.x. PubMed PMID: 22251383; PubMed Central PMCID: PMC3586988.

21. Nakagawa N, Kato M, Takahashi Y, Shimazaki K, Tamura K, Tokuji Y, et al. Degradation of long-chain base 1-phosphate (LCBP) in Arabidopsis: functional characterization of LCBP phosphatase involved in the dehydration stress response. J Plant Res. 2012;125(3):439-49. Epub 2011/09/13. doi: 10.1007/s10265-011-0451-9. PubMed PMID: 21910031.

22. Coursol S, Fan LM, Le Stunff H, Spiegel S, Gilroy S, Assmann SM. Sphingolipid signalling in Arabidopsis guard cells involves heterotrimeric G proteins. Nature. 2003;423(6940):651-4. Epub 2003/06/06. doi: 10.1038/nature01643. PubMed PMID: 12789341.

23. Worrall D, Liang YK, Alvarez S, Holroyd GH, Spiegel S, Panagopulos M, et al. Involvement of sphingosine kinase in plant cell signalling. Plant J. 2008;56(1):64-72. Epub 2008/06/19. doi: 10.1111/j.1365-313X.2008.03579.x. PubMed PMID: 18557834; PubMed Central PMCID: PMC2752831.

24. Du Z, Aghoram K, Outlaw WH, Jr. In vivo phosphorylation of phosphoenolpyruvate carboxylase in guard cells of Vicia faba L. is enhanced by fusicoccin and suppressed by abscisic acid. Arch Biochem Biophys. 1997;337(2):345-50. Epub 1997/01/15. PubMed PMID: 9016832.

25. Bak G, Lee EJ, Lee Y, Kato M, Segami S, Sze H, et al. Rapid structural changes and acidification of guard cell vacuoles during stomatal closure require phosphatidylinositol 3,5-bisphosphate. Plant Cell. 2013;25(6):2202-16. Epub 2013/06/13. doi: 10.1105/tpc.113.110411. PubMed PMID: 23757398; PubMed Central PMCID: PMCPMC3723621.

26. Li Z, Kang J, Sui N, Liu D. ROP11 GTPase is a negative regulator of multiple ABA responses in Arabidopsis. J Integr Plant Biol. 2012;54(3):169-79. Epub 2012/01/12. doi: 10.1111/j.1744-7909.2012.01100.x. PubMed PMID: 22233300.

27. Zhang W, Qin C, Zhao J, Wang X. Phospholipase D alpha 1-derived phosphatidic acid interacts with ABI1 phosphatase 2C and regulates abscisic acid signaling. Proc Natl Acad Sci U S A. 2004;101(25):9508-13. Epub 2004/06/16. doi: 10.1073/pnas.0402112101. PubMed PMID: 15197253; PubMed Central PMCID: PMC439007.

28. Mishra G, Zhang W, Deng F, Zhao J, Wang X. A bifurcating pathway directs abscisic acid effects on stomatal closure and opening in Arabidopsis. Science. 2006;312(5771):264-6. Epub 2006/04/15. doi: 10.1126/science.1123769. PubMed PMID: 16614222.

29. Meinhard M, Grill E. Hydrogen peroxide is a regulator of ABI1, a protein phosphatase 2C from Arabidopsis. FEBS Lett. 2001;508(3):443-6. PubMed PMID: 11728469.

30. Leube MP, Grill E, Amrhein N. ABI1 of Arabidopsis is a protein serine/threonine phosphatase highly regulated by the proton and magnesium ion concentration. FEBS Lett. 1998;424(1-2):100-4. Epub 1998/04/16. PubMed PMID: 9537523.

31. Meinhard M, Rodriguez PL, Grill E. The sensitivity of ABI2 to hydrogen peroxide links the abscisic acid-response regulator to redox signalling. Planta. 2002;214(5):775-82. doi: 10.1007/s00425-001-0675-3. PubMed PMID: 11882947.

32. Sridharamurthy M, Kovach A, Zhao Y, Zhu JK, Xu HE, Swaminathan K, et al. H2O2 inhibits ABA-signaling protein phosphatase HAB1. PLoS One. 2014;9(12):e113643. doi: 10.1371/journal.pone.0113643. PubMed PMID: 25460914; PubMed Central PMCID: PMC4252038.

33. Antoni R, Gonzalez-Guzman M, Rodriguez L, Rodrigues A, Pizzio GA, Rodriguez PL. Selective inhibition of clade A phosphatases type 2C by PYR/PYL/RCAR abscisic acid receptors. Plant Physiol. 2012;158(2):970-80. Epub 2011/12/27. doi: 10.1104/pp.111.188623. PubMed PMID: 22198272; PubMed Central PMCID: PMC3271782.

34. Umezawa T, Sugiyama N, Mizoguchi M, Hayashi S, Myouga F, Yamaguchi-Shinozaki K, et al. Type 2C protein phosphatases directly regulate abscisic acid-activated protein kinases in Arabidopsis. Proc Natl Acad Sci U S A. 2009;106(41):17588-93. doi: 10.1073/pnas.0907095106. PubMed PMID: 19805022; PubMed Central PMCID: PMC2754379.

35. Nishimura N, Sarkeshik A, Nito K, Park SY, Wang A, Carvalho PC, et al. PYR/PYL/RCAR family members are major in-vivo ABI1 protein phosphatase 2C-interacting proteins in Arabidopsis. Plant J. 2010;61(2):290-9. Epub 2009/10/31. doi: 10.1111/j.1365-313X.2009.04054.x. PubMed PMID: 19874541; PubMed Central PMCID: PMC2807913.

36. Merlot S, Gosti F, Guerrier D, Vavasseur A, Giraudat J. The ABI1 and ABI2 protein phosphatases 2C act in a negative feedback regulatory loop of the abscisic acid signalling pathway. Plant J. 2001;25(3):295-303. PubMed PMID: 11208021.

37. Saez A, Robert N, Maktabi MH, Schroeder JI, Serrano R, Rodriguez PL. Enhancement of abscisic acid sensitivity and reduction of water consumption in Arabidopsis by combined inactivation of the protein phosphatases type 2C ABI1 and HAB1. Plant Physiol. 2006;141(4):1389-99. doi: 10.1104/pp.106.081018. PubMed PMID: 16798945; PubMed Central PMCID: PMC1533955.

38. Rubio S, Rodrigues A, Saez A, Dizon MB, Galle A, Kim TH, et al. Triple loss of function of protein phosphatases type 2C leads to partial constitutive response to endogenous abscisic acid. Plant Physiol. 2009;150(3):1345-55. doi: 10.1104/pp.109.137174. PubMed PMID: 19458118; PubMed Central PMCID: PMC2705020.

39. Kwak JM, Mori IC, Pei ZM, Leonhardt N, Torres MA, Dangl JL, et al. NADPH oxidase AtrbohD and AtrbohF genes function in ROS-dependent ABA signaling in Arabidopsis. EMBO J. 2003;22(11):2623-33. Epub 2003/05/30. doi: 10.1093/emboj/cdg277. PubMed PMID: 12773379; PubMed Central PMCID: PMC156772.

40. Suhita D, Raghavendra AS, Kwak JM, Vavasseur A. Cytoplasmic alkalization precedes reactive oxygen species production during methyl jasmonate- and abscisic acid-induced stomatal closure. Plant Physiol. 2004;134(4):1536-45. doi: 10.1104/pp.103.032250. PubMed PMID: 15064385; PubMed Central PMCID: PMC419829.

41. Acharya BR, Jeon BW, Zhang W, Assmann SM. Open Stomata 1 (OST1) is limiting in abscisic acid responses of Arabidopsis guard cells. New Phytol. 2013;200(4):1049-63. Epub 2013/09/17. doi: 10.1111/nph.12469. PubMed PMID: 24033256.

42. Mustilli AC, Merlot S, Vavasseur A, Fenzi F, Giraudat J. Arabidopsis OST1 protein kinase mediates the regulation of stomatal aperture by abscisic acid and acts upstream of reactive oxygen species production. Plant Cell. 2002;14(12):3089-99. Epub 2002/12/07. PubMed PMID: 12468729; PubMed Central PMCID: PMC151204.

43. Sirichandra C, Gu D, Hu HC, Davanture M, Lee S, Djaoui M, et al. Phosphorylation of the Arabidopsis AtrbohF NADPH oxidase by OST1 protein kinase. FEBS Lett. 2009;583(18):2982-6. Epub 2009/09/01. doi: 10.1016/j.febslet.2009.08.033. PubMed PMID: 19716822.

44. Murata Y, Pei ZM, Mori IC, Schroeder J. Abscisic acid activation of plasma membrane Ca(2+) channels in guard cells requires cytosolic NAD(P)H and is differentially disrupted upstream and downstream of reactive oxygen species production in abi1-1 and abi2-1 protein phosphatase 2C mutants. Plant Cell. 2001;13(11):2513-23. PubMed PMID: 11701885; PubMed Central PMCID: PMC139468.

45. Zhang W, Jeon BW, Assmann SM. Heterotrimeric G-protein regulation of ROS signalling and calcium currents in Arabidopsis guard cells. J Exp Bot. 2011;62(7):2371-9. Epub 2011/01/26. doi: 10.1093/jxb/erq424. PubMed PMID: 21262908.

46. Zhang Y, Zhu H, Zhang Q, Li M, Yan M, Wang R, et al. Phospholipase dalpha1 and phosphatidic acid regulate NADPH oxidase activity and production of reactive oxygen species in ABA-mediated stomatal closure in Arabidopsis. Plant Cell. 2009;21(8):2357-77. Epub 2009/08/20. doi: 10.1105/tpc.108.062992. PubMed PMID: 19690149; PubMed Central PMCID: PMC2751945.

47. Park KY, Jung JY, Park J, Hwang JU, Kim YW, Hwang I, et al. A role for phosphatidylinositol 3-phosphate in abscisic acid-induced reactive oxygen species generation in guard cells. Plant Physiol. 2003;132(1):92-8. Epub 2003/05/15. doi: 10.1104/pp.102.016964. PubMed PMID: 12746515; PubMed Central PMCID: PMC166955.

48. Ellson CD, Gobert-Gosse S, Anderson KE, Davidson K, Erdjument-Bromage H, Tempst P, et al. PtdIns(3)P regulates the neutrophil oxidase complex by binding to the PX domain of p40(phox). Nat Cell Biol. 2001;3(7):679-82. Epub 2001/07/04. doi: 10.1038/35083076. PubMed PMID: 11433301.

49. An Z, Jing W, Liu Y, Zhang W. Hydrogen peroxide generated by copper amine oxidase is involved in abscisic acid-induced stomatal closure in Vicia faba. J Exp Bot. 2008;59(4):815-25. Epub 2008/02/15. doi: 10.1093/jxb/erm370. PubMed PMID: 18272918.

50. Hua D, Wang C, He J, Liao H, Duan Y, Zhu Z, et al. A plasma membrane receptor kinase, GHR1, mediates abscisic acid- and hydrogen peroxide-regulated stomatal movement in Arabidopsis. Plant Cell. 2012;24(6):2546-61. Epub 2012/06/26. doi: 10.1105/tpc.112.100107. PubMed PMID: 22730405; PubMed Central PMCID: PMCPMC3406912.

51. Desikan R, Griffiths R, Hancock J, Neill S. A new role for an old enzyme: nitrate reductase-mediated nitric oxide generation is required for abscisic acid-induced stomatal closure in Arabidopsis thaliana. Proc Natl Acad Sci U S A. 2002;99(25):16314-8. Epub 2002/11/26. doi: 10.1073/pnas.252461999. PubMed PMID: 12446847; PubMed Central PMCID: PMC138608.

52. Bright J, Desikan R, Hancock JT, Weir IS, Neill SJ. ABA-induced NO generation and stomatal closure in Arabidopsis are dependent on H2O2 synthesis. Plant J. 2006;45(1):113-22. Epub 2005/12/22. doi: 10.1111/j.1365-313X.2005.02615.x. PubMed PMID: 16367958.

53. Mulaudzi T, Ludidi N, Ruzvidzo O, Morse M, Hendricks N, Iwuoha E, et al. Identification of a novel Arabidopsis thaliana nitric oxide-binding molecule with guanylate cyclase activity in vitro. FEBS Lett. 2011;585(17):2693-7. Epub 2011/08/09. doi: 10.1016/j.febslet.2011.07.023. PubMed PMID: 21820435.

54. Joudoi T, Shichiri Y, Kamizono N, Akaike T, Sawa T, Yoshitake J, et al. Nitrated cyclic GMP modulates guard cell signaling in Arabidopsis. Plant Cell. 2013;25(2):558-71. Epub 2013/02/12. doi: 10.1105/tpc.112.105049. PubMed PMID: 23396828; PubMed Central PMCID: PMC3608778.

55. Sethi JK, Empson RM, Galione A. Nicotinamide inhibits cyclic ADP-ribose-mediated calcium signalling in sea urchin eggs. Biochem J. 1996;319 ( Pt 2):613-7. PubMed PMID: 8912702; PubMed Central PMCID: PMC1217811.

56. Walseth TF, Lee HC. Synthesis and characterization of antagonists of cyclic-ADP-ribose-induced Ca2+ release. Biochim Biophys Acta. 1993;1178(3):235-42. PubMed PMID: 8395888.

57. Rakovic S, Cui Y, Iino S, Galione A, Ashamu GA, Potter BV, et al. An antagonist of cADP-ribose inhibits arrhythmogenic oscillations of intracellular Ca2+ in heart cells. J Biol Chem. 1999;274(25):17820-7. PubMed PMID: 10364226.

58. Guse AH. Cyclic ADP-ribose: a novel Ca2+-mobilising second messenger. Cell Signal. 1999;11(5):309-16. Epub 1999/06/22. PubMed PMID: 10376802.

59. Staxen I, Pical C, Montgomery LT, Gray JE, Hetherington AM, McAinsh MR. Abscisic acid induces oscillations in guard-cell cytosolic free calcium that involve phosphoinositide-specific phospholipase C. Proc Natl Acad Sci U S A. 1999;96(4):1779-84. PubMed PMID: 9990101; PubMed Central PMCID: PMC15593.

60. Lemtiri-Chlieh F, MacRobbie EA, Webb AA, Manison NF, Brownlee C, Skepper JN, et al. Inositol hexakisphosphate mobilizes an endomembrane store of calcium in guard cells. Proc Natl Acad Sci U S A. 2003;100(17):10091-5. Epub 2003/08/13. doi: 10.1073/pnas.1133289100. PubMed PMID: 12913129; PubMed Central PMCID: PMC187775.

61. Zhang W, Fan LM, Wu WH. Osmo-sensitive and stretch-activated calcium-permeable channels in Vicia faba guard cells are regulated by actin dynamics. Plant Physiol. 2007;143(3):1140-51. Epub 2007/01/30. doi: 10.1104/pp.106.091405. PubMed PMID: 17259289; PubMed Central PMCID: PMC1820927.

62. Li S, Assmann SM, Albert R. Predicting essential components of signal transduction networks: a dynamic model of guard cell abscisic acid signaling. PLoS Biol. 2006;4(10):e312. Epub 2006/09/14. doi: 10.1371/journal.pbio.0040312. PubMed PMID: 16968132; PubMed Central PMCID: PMC1564158.

63. Swatek KN, Wilson RS, Ahsan N, Tritz RL, Thelen JJ. Multisite phosphorylation of 14-3-3 proteins by calcium-dependent protein kinases. Biochem J. 2014;459(1):15-25. Epub 2014/01/21. doi: 10.1042/BJ20130035. PubMed PMID: 24438037; PubMed Central PMCID: PMC4127189.

64. Jammes F, Song C, Shin D, Munemasa S, Takeda K, Gu D, et al. MAP kinases MPK9 and MPK12 are preferentially expressed in guard cells and positively regulate ROS-mediated ABA signaling. Proc Natl Acad Sci U S A. 2009;106(48):20520-5. Epub 2009/11/17. doi: 10.1073/pnas.0907205106. PubMed PMID: 19910530; PubMed Central PMCID: PMC2776606.

65. Nagy SK, Darula Z, Kallai BM, Bogre L, Banhegyi G, Medzihradszky KF, et al. Activation of AtMPK9 through autophosphorylation that makes it independent of the canonical MAPK cascades. Biochem J. 2015;467(1):167-75. Epub 2015/02/04. doi: 10.1042/BJ20141176. PubMed PMID: 25646663.

66. Jung JY, Kim YW, Kwak JM, Hwang JU, Young J, Schroeder JI, et al. Phosphatidylinositol 3- and 4-phosphate are required for normal stomatal movements. Plant Cell. 2002;14(10):2399-412. Epub 2002/10/09. PubMed PMID: 12368494; PubMed Central PMCID: PMC151225.

67. Otterhag L, Sommarin M, Pical C. N-terminal EF-hand-like domain is required for phosphoinositide-specific phospholipase C activity in Arabidopsis thaliana. FEBS Lett. 2001;497(2-3):165-70. Epub 2001/05/30. doi: S0014-5793(01)02453-X [pii]. PubMed PMID: 11377433.

68. Boss WF, Im YJ. Phosphoinositide signaling. Annu Rev Plant Biol. 2012;63:409-29. Epub 2012/03/13. doi: 10.1146/annurev-arplant-042110-103840. PubMed PMID: 22404474.

69. Zhao J, Wang X. Arabidopsis phospholipase Dalpha1 interacts with the heterotrimeric G-protein alpha-subunit through a motif analogous to the DRY motif in G-protein-coupled receptors. J Biol Chem. 2004;279(3):1794-800. doi: 10.1074/jbc.M309529200. PubMed PMID: 14594812.

70. Distefano AM, Scuffi D, Garcia-Mata C, Lamattina L, Laxalt AM. Phospholipase Ddelta is involved in nitric oxide-induced stomatal closure. Planta. 2012;236(6):1899-907. Epub 2012/08/31. doi: 10.1007/s00425-012-1745-4. PubMed PMID: 22932846.

71. Munnik T, Irvine RF, Musgrave A. Phospholipid signalling in plants. Biochim Biophys Acta. 1998;1389(3):222-72. PubMed PMID: 9512651.

72. Distefano AM, Garcia-Mata C, Lamattina L, Laxalt AM. Nitric oxide-induced phosphatidic acid accumulation: a role for phospholipases C and D in stomatal closure. Plant Cell Environ. 2008;31(2):187-94. Epub 2007/11/13. doi: 10.1111/j.1365-3040.2007.01756.x. PubMed PMID: 17996010.

73. Guo L, Mishra G, Taylor K, Wang X. Phosphatidic acid binds and stimulates Arabidopsis sphingosine kinases. J Biol Chem. 2011;286(15):13336-45. Epub 2011/02/19. doi: 10.1074/jbc.M110.190892. PubMed PMID: 21330371; PubMed Central PMCID: PMC3075680.

74. Guo L, Mishra G, Markham JE, Li M, Tawfall A, Welti R, et al. Connections between sphingosine kinase and phospholipase D in the abscisic acid signaling pathway in Arabidopsis. J Biol Chem. 2012;287(11):8286-96. Epub 2012/01/26. doi: 10.1074/jbc.M111.274274. PubMed PMID: 22275366; PubMed Central PMCID: PMCPMC3318714.

75. Puli MR, Rajsheel P, Aswani V, Agurla S, Kuchitsu K, Raghavendra AS. Stomatal closure induced by phytosphingosine-1-phosphate and sphingosine-1-phosphate depends on nitric oxide and pH of guard cells in Pisum sativum. Planta. 2016;244(4):831-41. Epub 2016/05/29. doi: 10.1007/s00425-016-2545-z. PubMed PMID: 27233507.

76. Wang XQ, Ullah H, Jones AM, Assmann SM. G protein regulation of ion channels and abscisic acid signaling in Arabidopsis guard cells. Science. 2001;292(5524):2070-2. Epub 2001/06/16. doi: 10.1126/science.1059046. PubMed PMID: 11408655.

77. Tang RJ, Liu H, Yang Y, Yang L, Gao XS, Garcia VJ, et al. Tonoplast calcium sensors CBL2 and CBL3 control plant growth and ion homeostasis through regulating V-ATPase activity in Arabidopsis. Cell Res. 2012;22(12):1650-65. doi: 10.1038/cr.2012.161. PubMed PMID: 23184060; PubMed Central PMCID: PMC3515760.

78. Baars TL, Petri S, Peters C, Mayer A. Role of the V-ATPase in regulation of the vacuolar fission-fusion equilibrium. Mol Biol Cell. 2007;18(10):3873-82. Epub 2007/07/27. doi: 10.1091/mbc.E07-03-0205. PubMed PMID: 17652457; PubMed Central PMCID: PMCPMC1995711.

79. Gary JD, Wurmser AE, Bonangelino CJ, Weisman LS, Emr SD. Fab1p is essential for PtdIns(3)P 5-kinase activity and the maintenance of vacuolar size and membrane homeostasis. J Cell Biol. 1998;143(1):65-79. Epub 1998/10/08. PubMed PMID: 9763421; PubMed Central PMCID: PMC2132800.

80. Martinoia E, Maeshima M, Neuhaus HE. Vacuolar transporters and their essential role in plant metabolism. J Exp Bot. 2007;58(1):83-102. doi: 10.1093/jxb/erl183. PubMed PMID: 17110589.

81. Ferjani A, Segami S, Horiguchi G, Muto Y, Maeshima M, Tsukaya H. Keep an eye on PPi: the vacuolar-type H+-pyrophosphatase regulates postgerminative development in Arabidopsis. Plant Cell. 2011;23(8):2895-908. Epub 2011/08/25. doi: 10.1105/tpc.111.085415. PubMed PMID: 21862707; PubMed Central PMCID: PMC3180799.

82. Krebs M, Beyhl D, Gorlich E, Al-Rasheid KA, Marten I, Stierhof YD, et al. Arabidopsis V-ATPase activity at the tonoplast is required for efficient nutrient storage but not for sodium accumulation. Proc Natl Acad Sci U S A. 2010;107(7):3251-6. Epub 2010/02/06. doi: 10.1073/pnas.0913035107. PubMed PMID: 20133698; PubMed Central PMCID: PMC2840351.

83. Schwartz A. Role of Ca-2+ and Egta on Stomatal Movements in Commelina-Communis L. Plant Physiol. 1985;79(4):1003-5. doi: Doi 10.1104/Pp.79.4.1003. PubMed PMID: ISI:A1985AXH0400016.

84. Kim YM, Han YJ, Hwang OJ, Lee SS, Shin AY, Kim SY, et al. Overexpression of Arabidopsis translationally controlled tumor protein gene AtTCTP enhances drought tolerance with rapid ABA-induced stomatal closure. Mol Cells. 2012;33(6):617-26. Epub 2012/05/23. doi: 10.1007/s10059-012-0080-8. PubMed PMID: 22610367; PubMed Central PMCID: PMCPMC3887759.

85. Gardner MK, Zanic M, Howard J. Microtubule catastrophe and rescue. Curr Opin Cell Biol. 2013;25(1):14-22. Epub 2012/10/25. doi: 10.1016/j.ceb.2012.09.006. PubMed PMID: 23092753; PubMed Central PMCID: PMCPMC3556214.

86. Islam MM, Hossain MA, Jannat R, Munemasa S, Nakamura Y, Mori IC, et al. Cytosolic alkalization and cytosolic calcium oscillation in Arabidopsis guard cells response to ABA and MeJA. Plant Cell Physiol. 2010;51(10):1721-30. Epub 2010/08/27. doi: 10.1093/pcp/pcq131. PubMed PMID: 20739306.

87. Gonugunta VK, Srivastava N, Puli MR, Raghavendra AS. Nitric oxide production occurs after cytosolic alkalinization during stomatal closure induced by abscisic acid. Plant Cell Environ. 2008;31(11):1717-24. Epub 2008/08/30. doi: 10.1111/j.1365-3040.2008.01872.x. PubMed PMID: 18721267.

88. Zhang X, Wang H, Takemiya A, Song CP, Kinoshita T, Shimazaki K. Inhibition of blue light-dependent H+ pumping by abscisic acid through hydrogen peroxide-induced dephosphorylation of the plasma membrane H+-ATPase in guard cell protoplasts. Plant Physiol. 2004;136(4):4150-8. doi: 10.1104/pp.104.046573. PubMed PMID: 15563626; PubMed Central PMCID: PMC535845.

89. Kinoshita T, Nishimura M, Shimazaki K. Cytosolic Concentration of Ca2+ Regulates the Plasma Membrane H+-ATPase in Guard Cells of Fava Bean. Plant Cell. 1995;7(8):1333-42. doi: 10.1105/tpc.7.8.1333. PubMed PMID: 12242406; PubMed Central PMCID: PMC160955.

90. Luo H, Morsomme P, Boutry M. The two major types of plant plasma membrane H+-ATPases show different enzymatic properties and confer differential pH sensitivity of yeast growth. Plant Physiol. 1999;119(2):627-34. PubMed PMID: 9952459; PubMed Central PMCID: PMC32140.

91. Lemichez E, Wu Y, Sanchez JP, Mettouchi A, Mathur J, Chua NH. Inactivation of AtRac1 by abscisic acid is essential for stomatal closure. Genes Dev. 2001;15(14):1808-16. Epub 2001/07/19. doi: 10.1101/gad.900401. PubMed PMID: 11459830; PubMed Central PMCID: PMCPMC312738.

92. Geiger D, Scherzer S, Mumm P, Stange A, Marten I, Bauer H, et al. Activity of guard cell anion channel SLAC1 is controlled by drought-stress signaling kinase-phosphatase pair. Proc Natl Acad Sci U S A. 2009;106(50):21425-30. Epub 2009/12/04. doi: 10.1073/pnas.0912021106. PubMed PMID: 19955405; PubMed Central PMCID: PMCPMC2795561.

93. Mori IC, Murata Y, Yang Y, Munemasa S, Wang YF, Andreoli S, et al. CDPKs CPK6 and CPK3 function in ABA regulation of guard cell S-type anion- and Ca(2+)-permeable channels and stomatal closure. PLoS Biol. 2006;4(10):e327. Epub 2006/10/13. doi: 10.1371/journal.pbio.0040327. PubMed PMID: 17032064; PubMed Central PMCID: PMC1592316.

94. Imes D, Mumm P, Bohm J, Al-Rasheid KA, Marten I, Geiger D, et al. Open stomata 1 (OST1) kinase controls R-type anion channel QUAC1 in Arabidopsis guard cells. Plant J. 2013;74(3):372-82. Epub 2013/03/05. doi: 10.1111/tpj.12133. PubMed PMID: 23452338.

95. Sasaki T, Mori IC, Furuichi T, Munemasa S, Toyooka K, Matsuoka K, et al. Closing plant stomata requires a homolog of an aluminum-activated malate transporter. Plant Cell Physiol. 2010;51(3):354-65. Epub 2010/02/16. doi: 10.1093/pcp/pcq016. PubMed PMID: 20154005; PubMed Central PMCID: PMC2835873.

96. Geiger D, Maierhofer T, Al-Rasheid KA, Scherzer S, Mumm P, Liese A, et al. Stomatal closure by fast abscisic acid signaling is mediated by the guard cell anion channel SLAH3 and the receptor RCAR1. Sci Signal. 2011;4(173):ra32. Epub 2011/05/19. doi: 10.1126/scisignal.2001346. PubMed PMID: 21586729.

97. Hedrich R. Ion channels in plants. Physiol Rev. 2012;92(4):1777-811. Epub 2012/10/18. doi: 10.1152/physrev.00038.2011. PubMed PMID: 23073631.

98. Negi J, Matsuda O, Nagasawa T, Oba Y, Takahashi H, Kawai-Yamada M, et al. CO2 regulator SLAC1 and its homologues are essential for anion homeostasis in plant cells. Nature. 2008;452(7186):483-6. doi: 10.1038/nature06720. PubMed PMID: 18305482.

99. Vahisalu T, Kollist H, Wang YF, Nishimura N, Chan WY, Valerio G, et al. SLAC1 is required for plant guard cell S-type anion channel function in stomatal signalling. Nature. 2008;452(7186):487-91. Epub 2008/02/29. doi: 10.1038/nature06608. PubMed PMID: 18305484; PubMed Central PMCID: PMC2858982.

100. Meyer S, Mumm P, Imes D, Endler A, Weder B, Al-Rasheid KA, et al. AtALMT12 represents an R-type anion channel required for stomatal movement in Arabidopsis guard cells. Plant J. 2010;63(6):1054-62. Epub 2010/07/16. doi: 10.1111/j.1365-313X.2010.04302.x. PubMed PMID: 20626656.

101. Dittrich P, Raschke K. Malate metabolism in isolated epidermis of Commelina communis L. in relation to stomatal functioning. Planta. 1977;134(1):77-81. Epub 1977/01/01. doi: 10.1007/BF00390098. PubMed PMID: 24419583.

102. Ward JM, Schroeder JI. Calcium-Activated K+ Channels and Calcium-Induced Calcium Release by Slow Vacuolar Ion Channels in Guard Cell Vacuoles Implicated in the Control of Stomatal Closure. Plant Cell. 1994;6(5):669-83. doi: 10.1105/tpc.6.5.669. PubMed PMID: 12244253; PubMed Central PMCID: PMC160467.

103. Miedema H, Assmann SM. A membrane-delimited effect of internal pH on the K+ outward rectifier of Vicia faba guard cells. J Membr Biol. 1996;154(3):227-37. PubMed PMID: 8952952.

104. Kohler B, Hills A, Blatt MR. Control of guard cell ion channels by hydrogen peroxide and abscisic acid indicates their action through alternate signaling pathways. Plant Physiol. 2003;131(2):385-8. doi: 10.1104/pp.016014. PubMed PMID: 12586862; PubMed Central PMCID: PMC1540280.

105. Sokolovski S, Blatt MR. Nitric oxide block of outward-rectifying K+ channels indicates direct control by protein nitrosylation in guard cells. Plant Physiol. 2004;136(4):4275-84. doi: 10.1104/pp.104.050344. PubMed PMID: 15563619; PubMed Central PMCID: PMC535857.

106. Grondin A, Rodrigues O, Verdoucq L, Merlot S, Leonhardt N, Maurel C. Aquaporins Contribute to ABA-Triggered Stomatal Closure through OST1-Mediated Phosphorylation. Plant Cell. 2015;27(7):1945-54. Epub 2015/07/15. doi: 10.1105/tpc.15.00421. PubMed PMID: 26163575; PubMed Central PMCID: PMCPMC4531361.

107. Jiang Y, Wu K, Lin F, Qu Y, Liu X, Zhang Q. Phosphatidic acid integrates calcium signaling and microtubule dynamics into regulating ABA-induced stomatal closure in Arabidopsis. Planta. 2014;239(3):565-75. doi: 10.1007/s00425-013-1999-5. PubMed PMID: 24271006.
