# Supplementary material for: A new discrete dynamic model of ABA-induced stomatal closure predicts key feedback loops
Source: PLoS Biol. 2017 Sep 22;15(9):e2003451. doi: 10.1371/journal.pbio.2003451 (PMC5627951; doi:10.1371/journal.pbio.2003451)
Supplement: S3 Text — (DOCX) [file pbio.2003451.s019.docx]

**S3 Text. Comparison of the predicted effect of node knockout in the presence of ABA in the Li et al. 2006 article and this work.**

The signal transduction network of ABA induced stomatal closure by Li et al. [[1](#_ENREF_1)] contained 50 nodes, of which 44 are maintained in our network model, two are refined (ABI1/2 is separated into ABI1 and ABI2, PLD is separated into PLDα and PLDδ), and four (Arg, NOS, KAP and ROP2) are eliminated due to insufficient follow-up verification. The present model contains 26 additional nodes documented after 2006, including RCARs, CPKs, Microtubule Depolymerization and Vacuolar Acidification. The dynamic model of Li et al. included 40 nodes, as a result of collapsing unregulated molecules such as Nitrite and disregarding the alternate sink node ROP10. Simulating the knockout of 39 nodes in the presence of ABA, Li et al. found 25 cases (65%) of close to wild type response to ABA, two cases of hypersensitivity (ABI1/2 and Ca^2+^ ATPase knockout), three cases of hyposensitivity (RBOH, ROS and Ca^2+^_c_ knockout), seven cases of reduced ABA sensitivity (PLD, PA, SphK, S1P, GPA1, KOUT, pH_c_ knockout) and three cases of insensitivity (disruption of Depolarization, AnionEM, and Actin Reorganization). Our present simulated knockouts of 79 nodes predict fewer close to wild type responses and more cases of differential sensitivity. Specifically, 17 cases (21.5%) yield close to wild type sensitivity to ABA, there are 10 cases of hypersensitivity, 18 cases of hyposensitivity, 21 cases of reduced sensitivity to ABA and 12 cases of ABA insensitivity. The examples of knockouts that yield decreased ABA sensitivity in our model versus close to wild type response in the Li et al. model include KEV and OST1 (which now lead to ABA insensitivity), RCN1 and CaIM (which now lead to reduced ABA sensitivity), ADPRc, cADPR, PLC, InsP6, CIS (which now lead to ABA hyposensitivity). Knockout of AtRAC1 and of H^+^ ATPase leads to ABA hypersensitivity in our model compared to close to wild type response in the Li et al. model. In addition, the knockout of ROS or of Ca^2+^_c_, which led to ABA hyposensitivity in the Li et al. model, yields ABA insensitivity in our model. The only case of a lesser effect in our model compared to the Li et al. model is the disruption of the reorganization of the actin cytoskeleton, which led to ABA insensitivity in the Li et al. model and reduced sensitivity in our model. Overall, our model maintains agreement with previous experimentally supported results and improves certain previous results in a manner consistent with the latest experimental evidence.

1. Li S, Assmann SM, Albert R. Predicting essential components of signal transduction networks: a dynamic model of guard cell abscisic acid signaling. PLoS Biol. 2006;4(10):e312. Epub 2006/09/14. doi: 10.1371/journal.pbio.0040312. PubMed PMID: 16968132; PubMed Central PMCID: PMC1564158.
